# Protein Lactylation Critically Regulates Energy Metabolism in the Protozoan Parasite *Trypanosoma brucei*

**DOI:** 10.3389/fcell.2021.719720

**Published:** 2021-10-14

**Authors:** Naiwen Zhang, Ning Jiang, Liying Yu, Tiandong Guan, Xiaoyu Sang, Ying Feng, Ran Chen, Qijun Chen

**Affiliations:** ^1^Key Laboratory of Livestock Infectious Diseases in Northeast China, Ministry of Education, Key Laboratory of Zoonosis, College of Animal Science and Veterinary Medicine, Shenyang Agricultural University, Shenyang, China; ^2^The Research Unit for Pathogenic Mechanisms of Zoonotic Parasites, Chinese Academy of Medical Sciences, Shenyang, China

**Keywords:** *Trypanosoma brucei*, post-translational modification, lactylation, lactate, metabolism

## Abstract

Lysine lactylation has been recognized as a novel post-translational modification occurring on histones. However, lactylation in non-histone proteins, especially in proteins of early branching organisms, is not well understood. Energy metabolism and the histone repertoire in the early diverging protozoan parasite *Trypanosoma brucei*, the causative agent of African trypanosomiasis, markedly diverge from those of conventional eukaryotes. Here, we present the first exhaustive proteome-wide investigation of lactylated sites in *T. brucei*. We identified 387 lysine-lactylated sites in 257 proteins of various cellular localizations and biological functions. Further, we revealed that glucose metabolism critically regulates protein lactylation in *T. brucei* although the parasite lacks lactate dehydrogenase. However, unlike mammals, increasing the glucose concentration reduced the level of lactate, and protein lactylation decreased in *T. brucei via* a unique lactate production pathway. In addition to providing a valuable resource, these foregoing data reveal the regulatory roles of protein lactylation of trypanosomes in energy metabolism and gene expression.

## Introduction

*Trypanosoma brucei* is a flagellated parasitic protozoan (Vickerman, 1985; Radwanska et al., 2018). It is the causative agent of African trypanosomiasis, known as sleeping sickness in humans and nagana in domestic animals. The prognosis of trypanosomiasis is generally poor, with a high mortality rate, as currently available treatments are inadequate and possess low therapeutic efficacy (Cullen and Mocerino, 2017). Hence, the search for new prophylactic and therapeutic options continues.

African trypanosomal parasites are transmitted between mammalian hosts by tsetse flies (*Glossina* sp.). They undergo morphological changes in response to the distinct physiological environments between mammalian blood and the tsetse fly midgut. The bloodstream form (BSF) of *T. brucei* is the main pathogenic stage in mammalian hosts. Its two developmental forms are proliferative long slender (LS), which has a very high metabolic energy demand, and arrested short stumpy, which is readily transmitted to the tsetse fly vector (Mony et al., 2014).

Cellular metabolism requires oxygen and various sugars as substrates. Anaerobic glycolysis produces a large amount of lactate, which is utilized as an energy source and is associated with the Warburg effect (Vander Heiden et al., 2009; Palsson-McDermott and O’Neill, 2013). Tumor cells opt for relatively inefficient anaerobic glycolysis to generate abundant lactate even under oxygen-rich conditions. In *T. brucei*, BSFs rely heavily on glycolysis for ATP biosynthesis even under aerobic conditions, rather than tricarboxylic acid cycle and oxidative phosphorylation, whereas PCFs rely primarily on oxidative phosphorylation (Bakker et al., 1997; Uzcátegui et al., 2018). During glycolysis, glucose consumed by cells is broken down to generate energy. Nevertheless, the glycolytic pathway in early branched trypanosomatid parasites substantially differs from those in other organisms (Opperdoes, 1987). In contrast to other eukaryotes, trypanosomes compartmentalize their glycolytic enzymes in peroxisome-derived organelles known as glycosomes (Bauer and Morris, 2017). Glycolysis generates several metabolic end-products that must be eliminated (Uzcátegui et al., 2018). Lactate is abundantly produced during glycolysis in conventional eukaryotes (Izzo and Wellen, 2019). However, as BSF *T. brucei* lacks lactate dehydrogenase (LDH), it converts glucose into pyruvate under aerobic conditions but forms pyruvate plus glycerol under anaerobic conditions (Haanstra et al., 2016; Smith et al., 2017). High rates of glycolysis yield methylglyoxal, which is a toxic by-product of the triosephosphate isomerase (TPI) reaction (Roberts et al., 2018). Dihydroxyacetone phosphate (DHAP) and glyceraldehyde 3-phosphate (GA-3P) generate methylglyoxal through an enediol intermediate (Wyllie and Fairlamb, 2011). As *T. brucei* expresses glyoxalase II (GLO2) but lacks glyoxalase I (GLO1), the end-product of methylglyoxal metabolism is mainly *L*-lactate, while other eukaryotic cells produce *D*-lactate (Greig et al., 2009; Sousa Silva et al., 2012).

A previous study showed that non-transcriptional mechanisms, such as metabolite-protein interactions and post-translational modification (PTM) of proteins, are highly correlated in terms of their metabolic control (Walsh et al., 2005). PTMs, such as protein acylation or phosphorylation, play critical roles in cellular metabolism and signal transduction, and can modulate protein conformation, stability, and function (Heinemann and Sauer, 2010). These modifications can alter the stability or function of the modified proteins, especially histones and their variants, and frequently work in concert to influence fundamental cellular processes, including gene expression (Kouzarides, 2007; Croken et al., 2012; Saha, 2020). Several studies have implicated PTMs in multiple aspects of trypanosome biology, especially in the modulation of parasite development and viability (Nett et al., 2009; Urbaniak et al., 2013; Moretti et al., 2018; Zhang et al., 2020).

Lysine lactylation (Kla) is a novel PTM identified in the core histones of humans and mice (Zhang et al., 2019; Irizarry-Caro et al., 2020), which markedly affects downstream gene expression and DNA replication. Histone lactylation activates genes associated with the process of clearing infections (Izzo and Wellen, 2019), and was therefore proposed as a mechanism for restoring tissue homeostasis. However, Kla in non-histone proteins has only been identified in plant fungal pathogens (Gao et al., 2020). The function of lactylation in non-histone proteins in other organisms, especially in the early branching organisms, is not well understood.

In addition to the different pathways of energy metabolism, the gene expression patterns of trypanosomes also have unique characteristics. The protein-coding genes of *T. brucei* are arranged in polycistronic transcription units (Jackson et al., 2008; Clayton, 2016, 2019). The primary transcript is processed into individual mRNAs by *trans*-splicing a capped splicing leader (Michaeli, 2011; Preußer et al., 2012), and by polyadenylation (Koch et al., 2016). mRNA processing, translation, and degradation compensate for the lack of individual gene transcription control (Clayton, 2019); thus, trypanosomes rely almost exclusively on post-transcriptional mechanisms to regulate the biosynthesis of their gene products. Moreover, RNA-binding proteins (RBPs) have vital functions. Further study on the lactylation of trypanosome proteins with unique biological characteristics is required to clarify the regulatory functions of lysine lactylation.

In this study, we elucidated the *T. brucei* lysine lactylome using an integrated proteome-wide method (Pandey and Mann, 2000). A total of 387 Kla sites were identified in 257 proteins. We performed an in-depth analysis of the modified proteins. The links between lactylation and glycolysis using multiple markers and at various levels were explored. The findings of this study provide a valuable resource that lays the foundation for the discovery of the specific functional roles of lactylation in *T. brucei*.

## Materials and Methods

### Ethics Approval and Consent to Participate

Six- to eight-week pathogen-free female BALB/c mice were maintained in an enriched environment. All animal experiments were performed according to the institutional guidelines on animal welfare and ethical permissions. The study was approved by the Ethical Committee of Shenyang Agricultural University, China (Clearance No. 2015-CAV-01).

### Parasite Cultivation and Purification

*Trypanosoma brucei* Lister 427 strain (Wirtz et al., 1999) were cultured *in vitro* in Hirumi’s modified Iscove’s medium (HMI-9) supplemented with 10% (v/v) fetal bovine serum (FBS) at 37∘C and 5% CO_2_ (Van Reet et al., 2011).

*Trypanosoma brucei in vivo* were purified from the blood of infected BALB/c mice using a previously described method (Lanham and Godfrey, 1970). *T. brucei* were intraperitoneally inoculated into healthy mice for serial passage. Trypanosomes from the blood of infected mice were purified over DEAE-cellulose (Sigma-Aldrich Corp., St. Louis, MO, United States) when parasitemia severity reached 1 × 10^8^/mL.

### Protein Extraction

Parasites were suspended in 4 volumes cold phenol extraction buffer containing 0.7 M sucrose; 0.1 M KCl; 0.5 M Tris–HCl; 1% TritonX-100; 10 mM dithiothreitol (DTT; Sigma-Aldrich Corp.), 1% protease inhibitors (Cocktail set V, Millipore, Billerica, MA, United States); 1% phosphatase inhibitor (Cocktail set III, Millipore); 50 μM deubiquitination inhibitor PR-619 (SelleckChem, Houston, TX, United States); 3 μM Trichostatin A (TSA, SelleckChem); 50 mM Nicotinamide (NAM, Sigma-Aldrich Corp.), 2 mM Ethylene Diamine Tetraacetic Acid (EDTA, Solarbio Life Sciences Inc., Beijing, China), and 2% β-mercaptoethanol. The mixture was ultrasonically for 3 min, pause for 5 s per 3 s for 25% intensity. An equal volume of Tris–balanced phenol (Solarbio Life Sciences Inc.) was added, and the mixture was centrifuged at 5,500 × *g* at 4∘C for 10 min. Five times the volume of 0.1 M ammonium acetate/methanol was added to the upper phenolic phase, and extraction was allowed to proceed overnight at −20∘C. The protein precipitate was then washed with methanol and acetone and redissolved with 8 M urea. The protein concentration was determined with a bicinchoninic acid kit (Beyotime Institute of Biotechnology, Jiangsu, China).

### Trypsin Digestion

Dithiothreitol was added to the protein solution to make up a final 5 mM concentration, and the volume was reduced by heating at 56∘C for 30 min. The mixture was alkylated with iodoacetamide (Sigma-Aldrich Corp.) and made up to a final 11 mM concentration. The mixture was incubated at room temperature in the dark for 15 min. The urea in the sample was diluted to <2 M concentration with 100 mM NH_4_HCO. Trypsin (Promega, Madison, WI, United States) was added at a 1:50 trypsin-to-protein mass ratio, and the first digestion was performed overnight at 37∘C. The second digestion was performed over 4 h using a 1:100 trypsin-to-protein mass ratio.

### High-Performance Liquid Chromatography Fractionation

Tryptic peptides (20 mg) were separated into fractions by high-pH, reverse-phase High-performance liquid chromatography (HPLC) on a Thermo Betasil C18 column (5 μm particles, 10 mm i.d., 250 mm length; Thermo Fisher Scientific, Waltham, MA, United States). Briefly, peptides were separated into 60 fractions using an acetonitrile gradient of 8–32% acetonitrile (pH 9.0) over 60 min. The peptides were then combined into four fractions and dried by vacuum centrifugation.

### Affinity Enrichment

Tryptic peptides dissolved in NETN buffer [100 mM NaCl, 1 mM EDTA, 50 mM Tris–HCl, and 0.5% (w/v) NP-40; pH 8.0] were incubated with pre-washed antibody beads (PTM Biolabs Inc., Chicago, IL, United States) overnight at 4∘C with gentle shaking. The beads were washed four times with NETN buffer and twice with H_2_O. The peptides were eluted from the beads with 0.1% (v/v) trifluoroacetic acid, and the eluted fractions were combined and vacuum-dried. For the LC-MS/MS analysis, the peptides were desalted with C18 ZipTips (EMD Millipore) according to the manufacturer’s instructions.

### LC-MS/MS Analysis

The tryptic peptides were dissolved in 0.1% (v/v) formic acid (solvent A) and loaded onto an in-house reversed-phase analytical column (15 cm length, 75 μm i.d.). The solvent gradient consisted of an increase in the range of 6–23% solvent B [0.1% (v/v) formic acid in 98% (v/v) acetonitrile] over 26 min, an increase in the range of 23–35% over 8 min, 80% over 3 min, and a final 80% over 3 min at a constant 400 nL/min flow rate in an EASY-nLC 1000 UPLC system (Thermo Fisher Scientific).

The peptides were subjected to the NSI source followed by tandem mass spectrometry (MS/MS) in a Q Exactive^TM^ Plus (Thermo Fisher Scientific) coupled online to the UPLC. The electrospray voltage was 2.0 kV, the m/z full scan range was 350–1,800, and the intact peptides were detected in an Orbitrap at 70,000 resolutions. The peptides were selected for MS/MS using NCE = 28, and the fragments were detected in the Orbitrap at 17,500 resolutions. The data-dependent procedure alternated between one MS scan followed by 20 MS/MS scans with 15.0 s dynamic exclusion. The automatic gain control and the fixed first mass were set to 5E4 and 100 m/z, respectively.

### Database Search

The resulting MS/MS data were processed using the Maxquant v. 1.5.2.8 search engine. Tandem mass spectra were searched against the UniProtKB database *T. brucei brucei* (strain 927/4 GUTat10.1; 8,587 sequences; release date 2017.10) concatenated with a reverse decoy database. Trypsin/P was the cleavage enzyme that allowed ≤4 missing cleavages. The mass tolerances for the precursor ions were set to 20 ppm in the first search and 5 ppm in the main search. The mass tolerance for the fragment ions was set to 0.02 Da. Carbamidomethyl on Cys was the fixed modification, and lactylation and oxidation on Met were the variable modifications. The false discovery rate (FDR) was adjusted to <1%, and the minimum score for the modified peptides was set to >40.

### Protein Annotation

Gene ontology (GO) annotation was derived from the UniProt-GOA database. For the proteins not annotated by the UniProt-GOA database, InterProScan software annotated the GO function of each protein based on the sequence alignment. The domain functional description was annotated by InterProScan, and the InterPro domain database was used. The KEGG online service tool KAAS annotated the KEGG database protein description. The annotation was mapped on the KEGG pathway database using the KEGG service tool KEGG Mapper. WoLF PSORT predicted the subcellular localizations.

### Statistical Analysis

#### Motif Analysis

Soft MoMo (motif-x algorithm) analyzed the sequence model consisting of amino acids in specific modify-21-mers positions in all protein sequences. There were 10 amino acids upstream and downstream of the site and phosphorylation with modify-13-mers at six amino acids upstream and downstream of the site. All database protein sequences served as background database parameters. The minimum number of occurrences was set to 20. The emulate original motif-x was ticked, and other parameters were used at their default values.

#### Functional Enrichment

All functional enrichments were performed with a two-tailed Fisher’s exact test. For further hierarchical clustering based on various functional classifications, all categories generated after the enrichment and their *P* values were collated. Categories enriched in ≥1 of the clusters at *P* < 0.05 were filtered. The filtered *P* value matrix was transformed by the function *x* = −log10 (*P*). The *x* values were *z*-transformed for each functional category. The *z* scores were clustered by one-way hierarchical clustering (Euclidean distance; average linkage) in Genesis. Cluster membership was visualized by a heatmap using the “heatmap.2” function in the “gplots” package of R (R Core Team, Vienna, Austria).

#### Enrichment-Based Clustering

Further hierarchical clustering was based on differentially modified protein functional classifications, including GO, domains, pathways, and complexes. All categories obtained were collated after enrichment with their *P* values. They were then filtered for categories enriched in ≥1 cluster with *P* < 0.05. The filtered *P* value matrix was transformed by the function *x* = −log10 (*P*), the *x* values were *z*-transformed for each functional category, and the *z* scores were clustered by one-way hierarchical clustering (Euclidean distance; average linkage) in Genesis. Cluster membership was visualized with a heatmap *via* the “heatmap.2” function in the “gplots” package of R (R Core Team).

#### Protein-Protein Interaction Network

All differentially expressed modified protein database accessions and sequences were searched for protein-protein interactions against the STRING v. 11.0 database. Only interactions between proteins in the searched dataset were selected, and external candidates were thereby excluded. STRING defines the metric “confidence score,” which is the interaction confidence. All interactions had a confidence score >0.7. The interaction network from STRING was visualized in the R package “networkD3” (R Core Team).

#### Data Statistics of the Functional Experiments

All experiments are set to more than 3 biological replicates. Statistical significance was determined by one-way ANOVA followed by Sidak’s multiple comparisons test. *P* values were determined using the Student’s *t*-test. ^∗^*P* < 0.05, ^∗∗^*P* < 0.01, and ^∗∗∗^*P* < 0.001 versus control.

### Viability Assays

*Trypanosoma brucei* BSF (100 μL; 2 × 10^5^/mL) was plated in 96-well plates and combined with 100 μL modulators to various concentrations in HMI-9 medium supplemented with 10% (v/v) FBS. Untreated parasites were plated as controls. After 24 h incubation, 20 μL PrestoBlue (Thermo Fisher Scientific) was added, and the assays were developed for 10 min. Fluorescence measurements were recorded with a SpectraMax M3 microplate reader (Molecular Devices LLC, San Jose, CA, United States) at 544 nm excitation and 590 nm emission. Data were analyzed in GraphPad Prism v. 8.0 (GraphPad Software Inc., La Jolla, CA, United States).

### Glycolytic Rate Assay

Parasites were incubated in Seahorse XF glycolytic rate assay medium (Agilent Technologies, Inc., Santa Clara, CA, United States). It is a Roswell Park Memorial Institute-based medium and does not contain phenol red, bicarbonate, glucose, pyruvate, or glutamine. It contains 1 mM HEPES. Then add glucose (up to 10 mM), glutamine (up to 2 mM) and sodium pyruvate (up to 1 mM) to this basal medium. Basal rates were recorded over three measurement periods. Rot/AA (mitochondrial electron transport chain inhibitors) were injected to minimize mitochondrial oxygen consumption and, by extension, CO_2_-derived protons. The second injection comprised 2-deoxy-*D*-glucose (2-DG), which inhibits glycolysis by competitively binding the initial glycolytic enzyme glucose hexokinase. The resulting decrease in PER qualitatively confirmed that the PER produced before the injection was produced mainly by glycolysis. The glycolytic rate was measured with the Seahorse XF glycolytic rate assay kit (Agilent Technologies, Inc.).

### Lactate Assay

Trypanosomes were plated at a density of 2 × 10^5^/mL on complete medium, were treated with glucose (0, 1, 5, and 25 mM), 2-DG (0, 1, 5, and 10 mM), oxamate (0, 5, 10, 20, and 20 mM) in HMI-9 medium containing 10% FBS at 37∘C for 24 h. After 24 h, *T. brucei* cells were harvested and washed 3 times with phosphate-buffered saline (PBS). The mixture was ultrasonically for 3 min, pause for 5 s per 3 s for 25% intensity, and deproteinized with a 10 KD filter (EMD Millipore) by centrifuging at 10,000 × *g* at room temperature for 20 min. *L*-lactate was quantitated with an assay kit (MAK064; Sigma-Aldrich Corp.) according to the manufacturer’s protocol. Data were analyzed in GraphPad Prism v. 8.0 (GraphPad Software Inc.).

### SDS-PAGE and Western Blot Analysis

The parasites were dissolved in SDS-PAGE loading buffer [250 mM Tris, 1.92 M glycine, and 1% (v/v) SDS], electrophoresed on SDS-PAGE gel, and transferred to a 0.2-μm nitrocellulose membrane (Bio-Rad Laboratories, Hercules, CA, United States). After blocking with Tris–buffered saline containing Tween 20 (TBST) and 5% skim milk (Sigma-Aldrich Corp.) at 37∘C for 1 h, the membrane was incubated in TBST containing 5% skim milk and anti-lactyllysine antibody (1:1,000 dilution; PTM Biolabs Inc.) at 4∘C for 12 h. The membrane was washed five times with TBST buffer and incubated with horseradish peroxidase (HRP)-conjugated goat anti-mouse IgG (H + L; 1:10,000; Thermo Fisher Scientific). The membrane was incubated with fluorogenic substrate (EMD Millipore) for 5 min, and the exposure time was adjusted according to the signal strength.

### Immunofluorescence Assay

*Trypanosoma brucei* smears were fixed with cold methanol at −80∘C for 10 s, air-dried, washed thrice with sterile PBS, and blocked in 3% (v/v) bovine serum albumin at 37∘C for 1 h. The slides were incubated in PBS containing monoclonal pan anti-lactyllysine antibody (PTM Biolabs Inc.) at 4∘C for 12 h. The smears were washed with PBS and incubated with Alexa Fluor 488-conjugated goat anti-rabbit IgG (Thermo Fisher Scientific) at 37∘C for 1 h. The nuclei were stained with 4′,6-diamidino-2-phenylindole (DAPI; Invitrogen, Carlsbad, CA, United States) for 5 min before image capturing under a fluorescence microscope (Leica Microsystems, Wetzlar, Germany). Mean fluorescence intensity was analyzed by ImageJ (NIH, Bethesda, MD, United States), and there were 10 replicates per group.

### Polyclonal Antibody Preparation and GADPH Immunoprecipitation

Polyclonal antibodies against glyceraldehyde 3-phosphate dehydrogenase (GAPDH) were prepared by immunizing New Zealand White rabbits with His-tagged recombinant proteins emulsified with Freund’s adjuvant (Sigma-Aldrich Corp.). The rabbits were subcutaneously immunized four times at two intervals. Antisera were collected 10 day after the fourth immunization. IgG was purified from the immune sera using Protein A Sepharose 4 Fast Flow (GE Healthcare, Little Chalfont, United Kingdom). Specificity and quality of the antibodies against the natural protein were verified by western blotting. For GAPDH immunoprecipitation, the parasites were lysed by sonication (3 s on; 7 s off; total 6 min) in 500 μL PBS with protease. The lysed cells were microcentrifuged (Eppendorf; Hamburg, Germany) at 12,000 rpm and 4∘C for 5 min. The cleared cell lysate was incubated overnight with 1.0 μL antibody at 4∘C and then with 40 μL Protein A-Sepharose beads (GE Healthcare).

### Reactive Oxygen Species Level, Apoptosis, and Cell Cycle Arrest of *T. brucei* After Treatment With Oxamate

*Trypanosoma brucei* cells were treated with oxamate (0, 5, 10, and 20 mM) in HMI-9 medium containing 10% FBS at 37∘C for 24 h. Next, *T. brucei* cells were harvested and washed 3 times with FBS-free HMI-9 medium, and analyzed by a BD FACSAria III flow cytometer (BD Biosciences, United States). The apoptosis assay was performed using the Annexin V-APC Apoptosis Detection Kit (Beyotime, China). Cell cycle was analyzed by flow cytometry with PI (Propidium Iodide) staining using a Cell Cycle and Apoptosis Analysis Kit (Beyotime, China). The reactive oxygen species (ROS) levels were monitored by measuring the oxidative conversion of the fluorescent molecular probe Dihydroethidium (DHE; BestBio, China).

## Results

### Proteome-Wide Analysis and Characterization of Lysine Lactylome in *T. brucei*

*Trypanosoma brucei* in various growth environments were collected from mouse blood at the peak of parasitemia and from *in vitro* culture media. The protein lactylation levels were compared by pan-antibody-based Western blotting and several lactylated proteins widely ranging in molecular masses were detected (Supplementary Figure 1A). Kla occurred relatively more frequently in parasites proliferating *in vivo*. To localize the lactylated lysine sites in *T. brucei*, parasite proteins were trypsinized and fractionated by liquid chromatography affinity enrichment. The lactylated peptides were detected by high-resolution MS/MS. The output data were processed, and the spectra were searched against the UniProtKB database *T. brucei* (strain 927/4 GUTat10.1; 8,587 sequences; and release date 2017.10). Thereafter, lactylation-specific proteomic analysis was performed (Supplementary Figure 1B).

We obtained 147,086 secondary spectra and 3,149 available spectra from three eligible biological replicates (Supplementary Figures 1B, 2A,B and Supplementary Table 1). A total of 2,523 peptides and 665 lactylated peptides were identified in BSF *T. brucei.* The peptide score was >40 (Supplementary Figure 3). In total, 257 lactylated proteins and 387 Kla sites were identified with a localization probability >0.75 and a 1% FDR. Of the 257 lactylated proteins, 76% (195/257) had one Kla site, 14% (35/257) had two Kla sites, and 5% (14/257) had three Kla sites (Supplementary Figure 1C). There were also 13 proteins with ≥4 Kla sites. The most extensively lactylated protein was heat shock protein 90 (HSP90, Q389P1), it has 14 independent lactylated lysine residues (Supplementary Table 1). HSP90 is also referred to as HSP83 due to the molecular weight of this HSP varies among orthologs. It has been proposed as a potential target for future drug discovery campaigns (Pizarro et al., 2013).

The characteristics of the Kla sites flanking sequences were discovered by searching the sequence motifs in all identified lactylated lysines, and identifying amino acid sequence enrichment in the region where lactylation occurred. The heatmap of amino acid enrichment surrounding each lactylated Lys (−10 − + 10; Supplementary Figure 4A) and the WebLogo consensus diagram (Supplementary Figure 4B) disclosed that protein lactylation in *T. brucei* has a conserved motif (XXXXXXXXXX_Kla_XXXXXXXKXX, where X indicates a random amino acid residue) based on the criteria of 10 amino acids upstream and 10 amino acids downstream of a lactylated lysine. Strikingly, the sequence logo showed a strong lysine (K) bias both upstream and downstream of the lactylated residue. Glycine (G) occurred most frequently at the −1 and +1 positions of the Kla sites.

**FIGURE 1:**
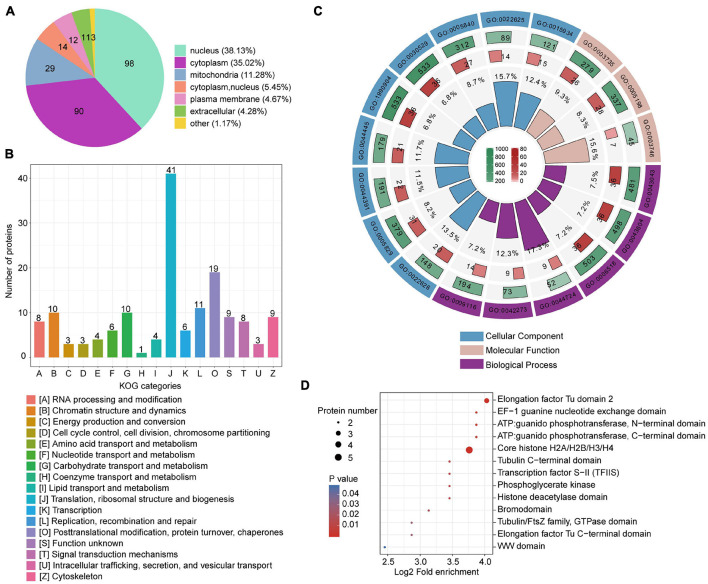
Compartmental Kla protein distribution and enrichment analysis. **(A)** Pie chart indicating subcellular lactylated protein localization. **(B)** KOG functional classification of identified lactylated proteins. **(C)** Enrichment analysis of GO items. From outside to inside: items, number of background proteins, number of identified proteins, and degree of enrichment. **(D)** Bubble plot displaying domain enrichment of lactylated proteins.

Lactylated *T. brucei* proteins were annotated using predicted functions and localizations (Figure 1 and Supplementary Table 2). These proteins have various subcellular localizations and are distributed in different cellular compartments. There were 98 lactylated proteins (38%) in the nucleus, 90 lactylated proteins (35%) in the cytoplasm, and 29 lactylated proteins (11%) in the mitochondria (Figure 1A). Classification based on eukaryotic orthologous groups (KOG) revealed that lactylated proteins broadly participated in translation, ribosomal structure and biogenesis, PTM, protein turnover, and chaperones (Figure 1B). GO annotation revealed that the largest class of lactylated proteins were associated with biological process and involved in metabolic processes. Numerous lactylated proteins bind cyclic compounds (Supplementary Figure 5). Several biological processes related to single-organism carbohydrate catabolism (GO: 0044724) and ribosomal large subunit biogenesis (GO: 0042273) were substantially enriched (Figure 1C, Supplementary Figures 6–8, and Supplementary Table 3). The enrichment of these functional categories might be related to their high abundance. We also detected the enrichment of lactylated proteins involved in translation elongation factor activity (GO: 0003746) and comprising structural ribosome constituents (GO: 0003735). Compared to *T. brucei* protein backgrounds, the Kla proteome is statistically enriched in cytosolic ribosomes (GO: 0022626) and the cytosolic large ribosomal subunit (GO: 0022625). Furthermore, we performed a domain enrichment analysis to elucidate the metabolic processes involved (Figure 1D). Elongation factors, ATP guanido phosphotransferase, and core histones were highly enriched in the *T. brucei* lactylome.

**FIGURE 2:**
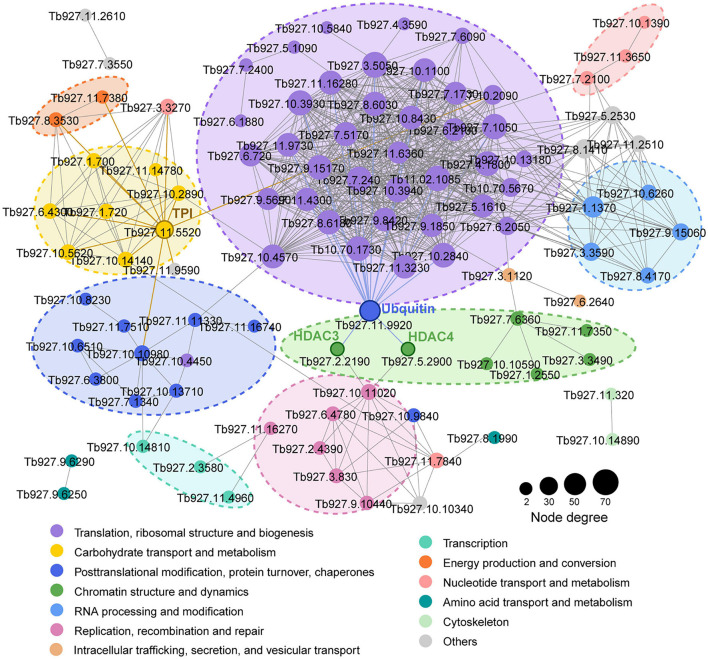
Kla protein interaction network. Protein-protein interaction networks for all Kla proteins. Interactive proteins are connected by lines. Different colors represent functional protein clusters based on KOG classification. Circle sizes are proportional to node degree.

We mapped the *T. brucei* lactylome onto protein-protein interactions predicted in the STRING database (v. 11.0) to explore the roles of lactylation in biological process regulation in *T. brucei* (Figure 2). A multitude of lactylated proteins were found to be involved in cellular processes such as translation, carbohydrate metabolism, chromatin dynamics, replication, recombination, and repair. A putative ubiquitin (Tb927.11.9920) strongly interacted with other proteins, including histone deacetylase 3 (HDAC3, Tb927.2.2190), which is essential and is specifically required for silencing of expression sites of variant surface glycoproteins (Wang et al., 2010) and histone deacetylase 4 (HDAC4, Tb927.5.2900), which regulates cell cycle progression (Ingram and Horn, 2002). Notably, TPI (Tb927.11.5520), an enzyme involved in carbohydrate metabolism, also showed strong interactions with proteins relating to energy production and the progression of translation. The substrate of the lactate production pathway, methylglyoxal, is mainly derived from the by-products of TPI. Moreover, these Kla proteins participate in different cellular functions such as metabolism and gene regulation, extensively characterizing the lactylome.

### Lysine Lactylation Occurred on Histones, Gene Regulators and Metabolic Enzymes

**FIGURE 3:**
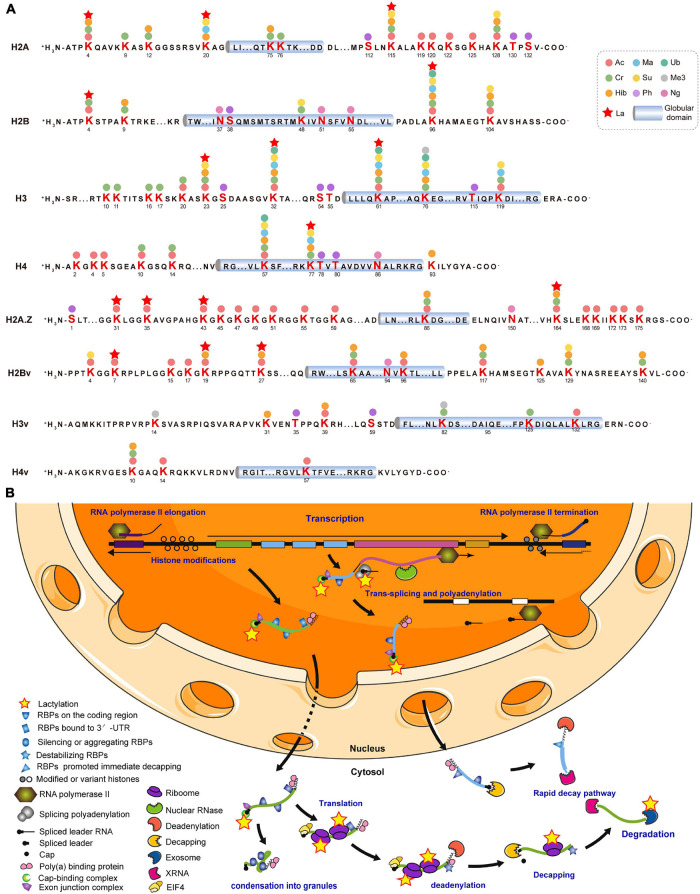
Lysine lactylation occurs on histones and gene regulators. **(A)** Cylinders indicate globular histone domains. Mature histone sequence is shown with modified residues in bold. Numbers represent amino acid positions after initial Met was removed. **(B)** This diagram is modified from Clayton (2016, 2019). Multiple ORFs lacking introns are arranged in a head-to-tail manner with some tandem repeats and occasional directional changes (Clayton, 2016). Most RNA Pol II transcription is initiated in DNA stretches several kilobases in length, separating gene arrays orientated in opposite, diverging directions. Initiation and termination regions are enriched with modified and variant histones. Pre-mRNA is processed into mature mRNAs by *trans*-splicing capped spliced leader and polyadenylation. Completed mRNA is exported to cytosol with bound poly (A) binding protein (PABP; Zoltner et al., 2018), exon junction complex (EJC; Bercovich et al., 2009), and nuclear cap-binding complex (CBC; Freire et al., 2017). Certain untranslated mRNAs bind and silence or induce the aggregation of RBPs, followed by condensation into granules. Mature mRNA binding by EIF4 is followed by translation (Dhalia et al., 2006). Stabilizing RBP is replaced by destabilizing RBP, followed by deadenylation (Schwede et al., 2009). After decapping, mRNA is degraded by XRNA and exosome (Fadda et al., 2013). A subset of mRNAs is immediately degraded. Kla proteins are represented by yellow pentagrams. RBPs are unlabeled but listed in Supplementary Table 5.

Various covalent histone PTMs regulate transcription and shape functional chromatin states (Sabari et al., 2017). We identified 16 Kla sites distributed across all *T. brucei* canonical and variant histones (Supplementary Table 4). The most comprehensive *T. brucei* histone modification map was generated by combining 10 others previously identified PTM types for a total of 178 PTM marks (Figure 3A; Zhang et al., 2020). All 16 Kla sites were associated with other PTMs. H2BK96, H3K23 (Supplementary Figure 3A), and H3K61 were modified by seven different PTMs. H2AK20 (Supplementary Figure 3B) can be lactylated and has the ability to undergo succinylation, malonylation, and 2-hydroxybutyrylation, which form active histone marks. Furthermore, the Kla sites on H3 and H4 were observed to be localized to the N- and C-terminal tails, which bear numerous PTM sites with various modifications such as hyperacetylation. H3K23 and H3K32 (Supplementary Figure 3C) in *T. brucei* may be synonymous with the universal H3K27 and H3K36 residues and might represent an ancient basal repertoire of histone modifications (Saha, 2020). H3K27La was previously identified in both human and mouse (Zhang et al., 2019), while H3K32La in *T. brucei* is probably a species-specific modification.

As *T. brucei* mainly regulates gene expression post-transcriptionally, 66 Kla sites were identified on 40 RBPs and most of the latter were elongation factors, including elongation factor 1 alpha 2 (EF1A2; P86939) with five Kla sites (Supplementary Table 5). Lactylated proteins widely participate in *trans*-splicing, cap-binding, and RNA export, translation, and degradation (Figure 3B). Additionally, Kla sites have been identified on the key enzymes associated with these processes, including eukaryotic translation initiation factor 3 subunit a (EIF3A; Q57VK2), importin alpha subunit (KAP60; Tb927.6.2640), and ribosomal RNA processing protein 45 (RRP45; Q583T6). The latter is a component of the major *T. brucei* cytosolic exosome complex (Estévez et al., 2001, 2003).

**FIGURE 4:**
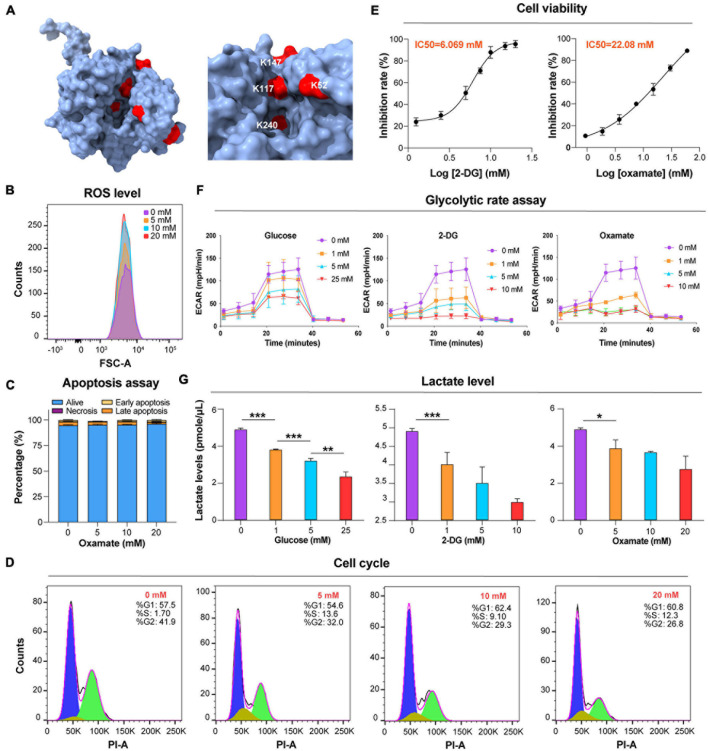
Glucose and glycolytic enzyme inhibitors mod ulate lactate levels. **(A)** Lactylated sites detected in ALDO are represented by the three-dimensional structure of protein. The lactylated sites are shown in red (PDB: 1F2J). **(B)** Flow cytometry determination of cellular ROS levels of *T. brucei* with DHE after exposure to the different concentrations of oxamate for 24 h. **(C)** Analysis of apoptosis/necrosis in *T. brucei* after exposure to different concentrations of oxamate for 24 h. **(D)** Cell cycle distribution analysis of *T. brucei* exposure to different concentrations of oxamate for 24 h based on the flow cytometry results. **(E)** *T. brucei* were incubated for 24 h in presence of variable amounts of indicated inhibitors. IC50 and inhibition calculated by PrestoBlue as described in Star Methods. **(F)** Extracellular acidification rate (ECAR) after sequential addition of rotenone and antimycin A (Rot/AA; mitochondrial ETC inhibitors) and 2-DG. **(G)** Lactate levels were measured with lactate fluorescence kit. *N* = 3 biological replicates. Statistical significance was determined by one-way ANOVA followed by Sidak’s multiple comparisons test. *P* values were determined using the Student’s *t*-test. *P < 0.05, **P < 0.01, and ***P < 0.001 versus control.

Glycolysis is the main energy metabolism pathway in BSF trypanosomes (Visser and Opperdoes, 1980). Twenty-five Kla sites were identified on seven lactylated glycolytic enzymes (Figure 1A and Supplementary Table 6). Aldolase (ALDO), enolase (ENO), and GAPDH each had >5 Kla sites. There were two Kla sites located in the active sites of ALDO, K240, one of which, is the catalytic residue of this protein (Figure 4A and Supplementary Figure 3D). Additionally, lactylated sites of phosphoglycerate kinase (PGK) and pyruvate kinase (PYK) were also identified, which may have roles in catalyzing the processes accompanying ATP production. High glycolytic throughput yields copious amounts of methylglyoxal from DHAP and glyceraldehyde 3-phosphate (GA-3-P) *via* an enediol intermediate (Wyllie and Fairlamb, 2011). Kla was detected on K217 of the enzyme TPI with a strong interaction with other proteins found in the network above. Lactylation of other enzymes of the methylglyoxal detoxification pathway was not observed.

### Glucose Metabolism Regulates Protein Lactylation and Gene Expression

Lactate is a critical determinant of histone Kla levels (Zhang et al., 2019). To examine whether changes in the lactate level affect trypanosomal histone and total protein lactylation, we evaluated whether variations of three different treatments altered lactate levels and, thereby, protein lactylation. The first group consisted of glucose added to the medium at different concentrations; in the second group, activity levels of the enzymes involved in glucose metabolism were altered using the glucose analog 2-DG, which inhibits hexokinase; and in the third group, parasites were cultured in distinct concentrations of oxamate, the LDH inhibitor. Although oxamate can slightly increase the generation of ROS in the mitochondria (Figure 4B), it was not observed to promote apoptosis as in previous studies due to the lack of LDH in *T. brucei* (Figure 4C; Zhai et al., 2013). However, it is worth noting that with an increase in the concentration of oxamate added to the culture medium, G2/M cell cycle arrest (Figure 4D) is induced, and oxamate may target proteins such as cyclin-dependent kinases. We further measured the effects of different concentrations of 2-DG and oxamate on parasite viability and found that the inhibition rate increased with inhibitor concentration (Figure 4E). The half-maximal inhibitory concentration (IC50) for 2-DG and oxamate at 24 h were 6.069 and 22.08 mM, respectively. Both modulators lowered the glycolytic rates by detecting the extracellular acidification rate (ECAR; Figure 4F). Further, the intracellular lactate levels of the parasite decreased with increasing treatment concentrations (Figure 4G).

**FIGURE 5:**
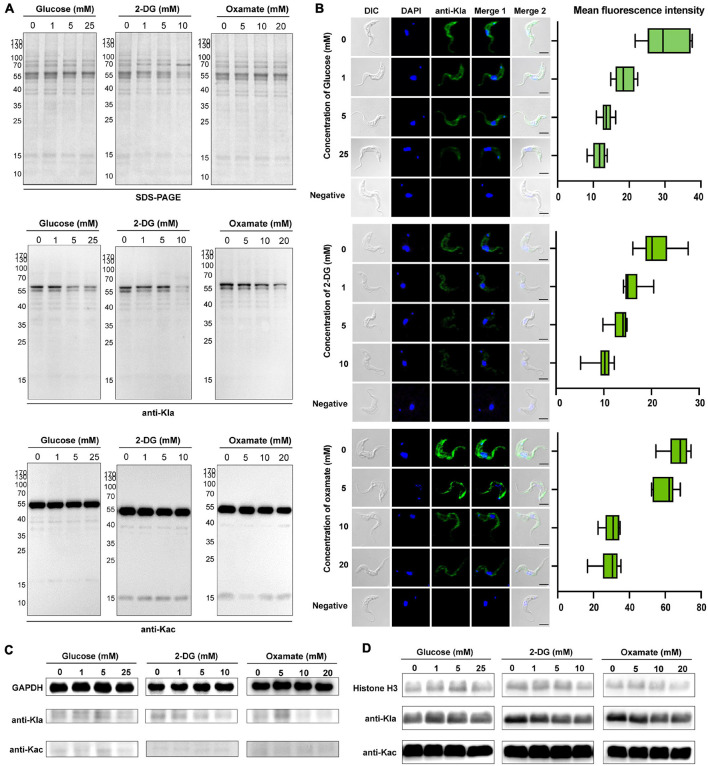
Kla is stimulated by lactate and contributes to gene expression. **(A)** SDS-PAGE and immunoblots of total *T. brucei* lysates in response to various glycolysis modulators. The upper panel shows equal loading amounts by SDS-PAGE. The middle and the lower panels show Western blotting assay probed with a monoclonal anti-lactyllysine antibody and anti-acetyl lysine antibody, respectively. **(B)** Immunofluorescence of lactylated *T. brucei* proteins with an anti-Kla antibody under the same exposure times, parameters, and statistics of mean fluorescence intensity. The fluorescence intensity decreases with the increases in glucose, 2-DG, and oxamate concentrations. Scale bar, 10 μm. **(C,D)** Immunoblots of GAPDH obtained by IP **(C)** and extracted histones **(D)** from *T. brucei* in response to various glycolysis modulators. The upper panel shows equal loading amounts with antibodies of target proteins. The middle and the lower panels show Western blotting assay probed with a monoclonal anti-lactyllysine antibody and anti-acetyl lysine antibody, respectively. The gray scale of the bands in the groups of 2-DG and oxamate incubated with the anti-KLa antibody decrease with the concentration.

Furthermore, lactylation levels were measured by Western blotting and immunofluorescence of total protein. As a result, lactylated proteins were reduced by these treatments, accompanied by a decrease in the lactate level, but the acetylation level remained unchanged (Figures 5A,B). For these reasons, glucose metabolism, cell division, and lactate generation have a targeted influence on lactylation. Similar results were obtained from GAPDH and histone H3 when 2-DG and oxamate were added (Figures 5C,D). Nevertheless, the glucose treatment group showed no marked change.

To establish whether this regulatory role on lactylation affects gene expression, we screened approximately 60 gene regulatory factors for RT-qPCR and found that most of the elongation factors and RBPs were substantially up-regulated (Supplementary Figure 9A), especially U3 small nucleolar ribonucleoprotein protein (MPP10, Q57W95) and ribosome biogenesis regulatory protein (RRS1, Q584V1; Supplementary Figure 9B). The expression levels of mRNA of ATP-dependent DEAD/H RNA helicase decreased with the increase in 2-DG concentration, while the transcription level of this protein may be closely related to the amount of hexokinase. To sum up, these different treatments affect glycolysis and are accompanied by changes in the degree of protein lactylation, especially in histones, and ultimately caused changes in gene expression.

### Crosstalk Between Lysine Lactylation and Other Post-Translational Modifications

**FIGURE 6:**
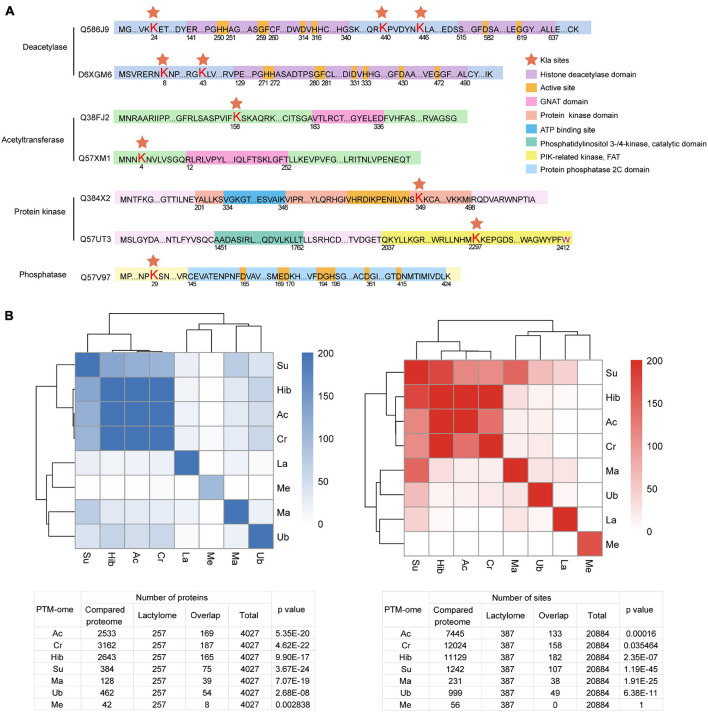
Overview of Kla sites identified on *T. brucei* “writers” and “erasers.” **(A)** Red pentagrams: Lys lactylation sites. Domains are marked with different colors. **(B)** Heatmaps and tables displaying the significance (−Log10 *p* value) of the overlaps of PTM datasets with one another.

Post-translational modifications are regulated by a dynamic balance between the enzymatic activities of “writers” and those of “erasers” (Zhao et al., 2018). Enzyme lactylation is summarized in Figure 6A. Kla sites were detected on two deacetylases and two acety ltransferases. However, none of them occurred in the critical catalytic or active domains. Moreover, lactylation occurs in the kinase domain while the phosphatase Kla site is localized to the N-terminal.

Crosstalk between PTM occurs in several organisms, including *T. brucei* (Zhang et al., 2020). PTM can promote or inhibit the occurrence of another PTM or act in a combinatorial manner. To explore crosstalk between lactylation and other PTMs, we analyzed the significance of overlaps between the lysine lactylome and proteins detected in previously published proteome-wide PTM datasets surveying lysine acetylation, crotonylation, 2-hydroxyisobutyrylation, succinylation, malonylation, and ubiquitination and the trymethylation proteome (Figure 6B). The lactylome is significantly enriched in succinylated (−log2 *p* value 77.9), crotonylated (−log2 *p* value 13.5), and acetylated proteins (−log2 *p* value 33.9).

## Discussion

Lactylation, a novel acylation was proposed in 2019, histone lactylation in mammalian cell lines were identified by mass spectrometry (Zhang et al., 2019). Initial evidence for lactylation came from a mass shift observed on lysine residues in HPLC–MS/MS analysis. The mass shift is the same as that caused by the addition of a lactyl group to the ε-amino group of a lysine residue. Furthermore, comparative the mass spectrum of chemical synthetic peptide and intracellular modified peptide, verifying the presence of lactylation. The study found that lysine lactylation is regulated by alterations in glucose metabolic dynamics of glucose and lactate levels. Then lactyl-CoA has been detected in mammalian cells and tissues (Varner et al., 2020). Elucidation of the biochemistry involved in both the transfer of lactyl moieties from lactyl-CoA to histones and the removal of lactyl groups in cellular physiology is an ongoing effort. Identification of p300 and Class I HDAC as regulatory enzymes suggests that histone lactylation is added and removed by enzymes as opposed to spontaneous chemical reactivity (Moreno-Yruela et al., 2021). As far, this is the first study in parasites, and enzymes that regulate lactylation in trypanosomes remain to be identified. Further investigation will clarify more details about how histone lactylation marks are written in a gene-specific manner and how they regulate transcription, as well as about the biochemical process that precisely regulates histone lactation, which may become an important future research topic (Chen et al., 2021).

Trypanosomes are early branched glycosome-containing organisms; even their extremely conservative histone sequences are markedly different from other organisms. Glycolysis occurs in their specific organelles, and the final product is not lactate, that is, the source of lactate is different from that in other organisms. We previously established the first comprehensive multiple PTM-omics atlas and quantitative proteomes of two phenotypically different representative African trypanosome species (*T. brucei* and *T. evansi*; Zhang et al., 2020). Here, we report a novel PTM-ome, namely, the BSF *T. brucei* lactylome. We found that lactylation exerted profound regulation in trypanosome gene expression and energy metabolism.

Comparison of lactylation levels in *T. brucei* from infected hosts and from *in vitro* cultivation revealed that the degree of modification was higher in the former than the latter, indicating protein lactylation was affected by the ambient environment of the parasite. To study the role of lactylation in the disease-causing phase of trypanosomiasis, we selected BSF *T. brucei* as the study sample and collected the parasites from infected mice.

A lysine residue at the + 8 position in a polypeptide is the preferred lysine lactyltransferase substrate. Lactylated proteins have various subcellular localizations and are distributed in different cellular compartments. They participate in a broad range of cellular functions with extensive lactylome characterization such as metabolism and gene regulation. Hence, protein lactylation could be vital to *T. brucei* biology. Lactylation of the lysine residues in proteins is related to carbohydrate catabolism and translation and especially ribosomal biogenesis and may play an important physiological role.

Relative to the plant fungal pathogen *Botrytis cinerea* (Gao et al., 2020), *T. brucei* had more lactylated proteins and sites. Of the 257 lactylated proteins detected here, 14 Kla sites were identified on HSP90, which is a heat shock protein and a cellular-level stress response marker. HSP90 is a molecular chaperone that helps peptides avoid misfolding and limits protein aggregation under heat stress (Sima and Richter, 2018). Key regulatory proteins in eukaryotes require HSP90 during maturation and are potential drug discovery targets (Pizarro et al., 2013; Schopf et al., 2017). The interactions between lactylated proteins can contribute to cooperation and coordination during the diverse biological processes of trypanosomes.

Histone modification is a secondary vehicle for heritable message transmission (Figueiredo et al., 2009). The PTM status of the core histones determines whether the chromatin is repressed or induced with respect to transcription and antigenic variation (Martínez-Calvillo et al., 2018; Stillman, 2018). We mapped the most current and complete histone modifications, including those previously described for trypanosomal proteins. Histones are widespread in the genome (Siegel et al., 2009), and their lactylation may vary with their specific functions at each genomic location. All 16 Kla sites were substrates of other PTMs. Thus, these PTMs have dynamic and complex roles in regulating histone function, and they provide an opportunity for regulation because dynamic chromatin structures can be locally and globally modified to influence DNA accessibility (Kraus et al., 2020). H3K23 and H3K32 in *T. brucei* may be synonymous with the universal H3K27 and H3K36 residues. Acetylation of H3K27 is associated with transcription in several organisms and is catalyzed by hGCN5/KAT2A in humans (Hyland et al., 2005). Human H3K36 can be methylated by SET2/KMT3 and might be involved in transcription elongation of Pol II (Shilatifard, 2006). However, H3K36 was not lactylated in the omics of humans and mice (Zhang et al., 2019). Hence, TbH3K32La is probably a *T. brucei*-specific PTM. The foregoing results furnish empirical evidence for the presence of Kla in the *T. brucei* chromatin and the possibility that it may have been conserved in *T. brucei*-specific regulatory functions.

In trypanosomes, there are no classical transcription factors, RNA polymerase II transcription is polycistronic, and individual mRNAs are excised by *trans*-splicing and polyadenylation (Clayton, 2019). The mRNA processing, translation, and degradation control mechanisms compensate for the aforementioned lack of individual gene transcription control. A putative mechanism for modulating *T. brucei* gene expression is the use of RBPs to process and stabilize mRNAs. Several RBPs such as elongation factors were detected in the lactylome reported here. We also found numerous lactylated enzymes and regulators involved in gene regulation and mRNA splicing, processing, export, translation, and degradation. Therefore, lactylation might play a role in gene expression as previously described (Izzo and Wellen, 2019; Zhang et al., 2019).

Cellular metabolism involves nutrient uptake, release, and interconversion to produce energy and synthesize complex biomolecules. Metabolic intermediates and end-products modulate cell signaling and gene expression in accordance with nutrient availability. Glucose is metabolized to pyruvate, which is canonically oxidized to acetyl-CoA for subsequent metabolism in the citric acid (Krebs; TCA) cycle and ATP biosynthesis (Liberti and Locasale, 2020). However, glucose may also be incompletely oxidized to lactate even in the presence of oxygen *via* the Warburg effect (Vander Heiden et al., 2009). The TCA cycle is absent in LS *T. brucei*, the glycolysis pathway is the main way that generates energy in a manner similar to the Warburg effect. A recent study demonstrated that the activity changes in ALDO depend on glucose availability in PCF trypanosomes, which is associated with the changes in the Kac levels of the protein (Barbosa Leite et al., 2020). We found that lactylation occurs at the catalytic site of the enzyme in BSF *T. brucei*, which may be related to activity changes. Further, PGK and PYK were also lactylated, but the roles of lactylation of these enzymes in ADP binding and their regulatory functions require further study.

**FIGURE 7:**
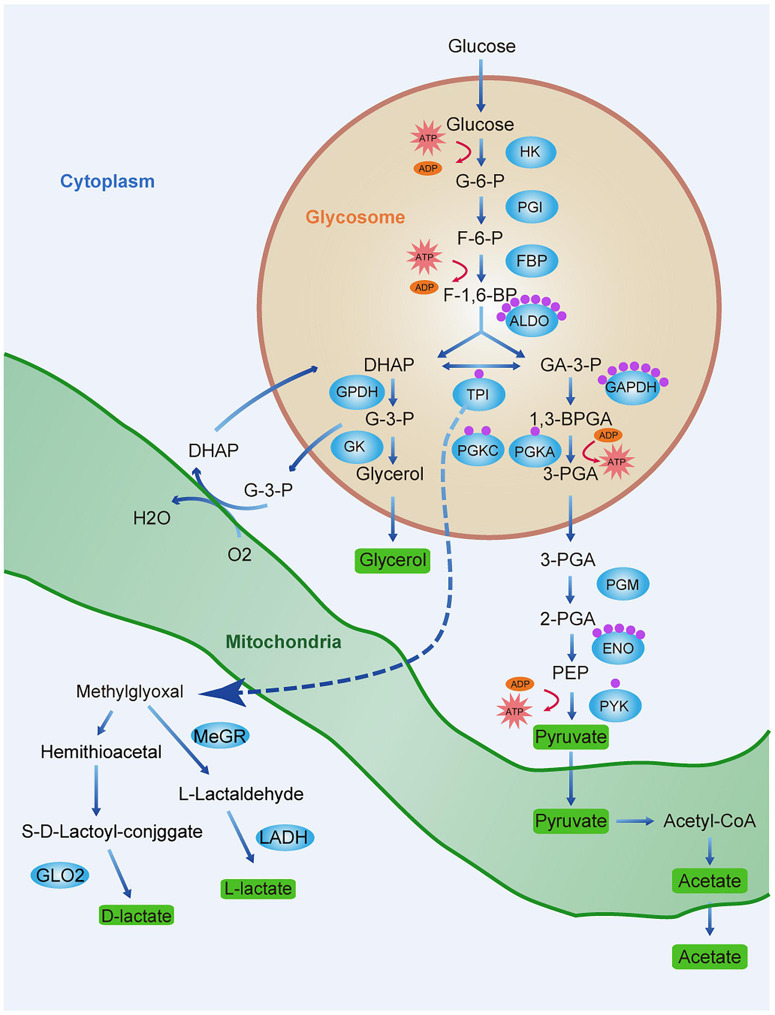
Lactate production and lactylation in the glycolysis of *T. brucei*. Schematic diagram of the glycolytic pathway and lactate production pathway in *T. brucei*. Glycerol 3-phosphate (G3P) is re-oxidized by mitochondrial glycerol phosphate complex and returned to the glycosome for further cycling. Green rectangles represent end-products which must be excreted by parasite to maintain homeostasis and ensure survival. Blue ellipses represent enzymes of the metabolic pathways. Each purple sphere represents a lactylated site on the protein. Abbreviations: G-6-P, glucose 6-phosphate; F-6-P, fructose 6-phosphate; F-1,6-BP, fructose 1,6-bisphosphate; DHAP, dihydroxyacetone phosphate; GA-3-P, glyceraldehyde 3-phosphate; G-3-P, glycerol-3-phosphate; 1,3-BPGA, 1,3-bisphosphoglycerate; 3-PGA, 3-phosphoglycerate; 2-PGA, 2-phosphoglycerate; PEP, phosphoenolpyruvate; HK, hexokinase; PGI, glucose phosphate isomerase; PFK, phosphofructokinase; TPI, triosephosphate isomerase; GPDH, glycerol-3-phosphate dehydrogenase; GK, glycerol kinase; GAPDH, glyceraldehyde-3-phosphate dehydrogenase; PGK, phosphoglycerate kinase; PGM, phosphoglycerate mutase; PYK, pyruvate kinase; G6PDH, glucose-6-phosphate dehydrogenase; MeGR, methylglyoxal reductase; LADH, lactaldehyde dehydrogenase; and GLO2, glyoxalase II.

However, as trypanosomes lack LDH, they cannot convert glucose to lactate. The latter is derived from the glyoxalase pathway (Figure 7). In mammalian cells, the principal detoxification route of this reactive metabolite is the glutathione-dependent glyoxalase pathway, which forms *D*-lactate and involves GLO1 and GLO2 (Al-Balas et al., 2019). The African trypanosome differs from *T. cruzi* and *Leishmania* sp. in that it lacks GLO1 and converts methylglyoxal to *L*-lactate rather than *D*-lactate (Sousa Silva et al., 2012). Trypanosome glyoxalase has been proposed as a drug target (Wyllie and Fairlamb, 2011; Uzcátegui et al., 2018). In the present study, lactylation was detected on K271 of TPI, which is a glycolytic enzyme in the glyoxalase pathway. Furthermore, TPI showed strong interactions with proteins relating to energy production. The significance of lactylation of TPI deserves further study.

As the lactylation substrate lactate is a glucose metabolism end-product, we explored the regulatory relationship between glycolysis and lactylation. We treated trypanosomes with three modulators: glucose, which is a glycolysis substrate; 2-DG targeting hexokinase; and oxamate targeting LDH. Glucose metabolism regulates protein lactylation in trypanosomes, but the regulation pattern differs from mammals. Due to its robust substrate saturable uptake activity, increasing the glucose concentration will reduce the lactate level in trypanosome thereby reducing lactylation. However, although it lacks LDH, increasing the concentration of oxamate in *T. brucei* culture arrested the G2/M phase of the cell cycle and reduced vitality, probably by downregulating the CDK1/cyclin B1 pathway as previously described (Zhai et al., 2013). The insignificant changes in ROS levels and apoptosis may be related to the mitochondrial inhibition of BSF trypanosomes; thus, oxamate mainly affects the changes in other indicators by inducing cell cycle arrest. The real-time ECAR reflected decreases in the glycolytic rates in response to various glycolysis modulator concentrations. In the absence of any change in the protein level, the lactate and lactylation levels decreased with increasing inhibitor concentration. In contrast to the results of a previous study on MCF-7 cells (Zhang et al., 2019), in the present study, the trypanosome lactate levels gradually decreased with increasing glucose concentration (Figure 4G). It is possible that the pyruvate concentration increases with glycolysis rate, and the former is eventually converted to acetate rather than lactate (Uzcátegui et al., 2018). *T. brucei* has a robust substrate saturable uptake activity with Km values in the low mM range, suggesting that BSF *T. bruce*i adapts its glucose transport in a manner that affords maximum yield at minimum expense (Ter Kuile and Opperdoes, 1991). Additionally, externally added glucose interfered with glycerol metabolism, which suggests that the rate-limiting step is at the level of glycerol kinase.

Moreover, by integrating our results, we found that reducing the level of lactate through the addition of modulators can affect the lactylation level of histone H3 and increase the expression of transcription elongation factor mRNA.

In conclusion, Kla in *T. brucei* is a critical modification with various cellular functions. Our data initiated the process of elucidating the roles and abundance of lactylation in *T. brucei.* This information will increase our understanding of lysine lactylation in the cellular physiology of *T. brucei* and other protozoan parasites. Further, this study provides theoretical and empirical bases for ongoing investigations that could build on this knowledge to discover potential drug targets for treatments against *T. brucei*.

## Data Availability Statement

The datasets presented in this study can be found in online repositories. The names of the repository/repositories and accession number(s) can be found below: ProteomeXchange consortium *via* the PRIDE archive (accession number PXD466466, http://proteomecentral.proteomexchange.org).

## Ethics Statement

The animal study was reviewed and approved by the Ethical Committee of Shenyang Agricultural University, China (Clearance No. 2015-CAV-01).

## Author Contributions

QC conceived the study and analyzed the data. NZ performed most of the experiments, analyzed the data, and wrote the first draft of the manuscript. NJ mentored the study. LY and TG assisted the glycolysis rate assay experiment. XS assisted WB experiment. YF and RC assisted the IFA experiment. QC finalized the manuscript. All authors contributed to the article and approved the submitted version.

## Conflict of Interest

The authors declare that the research was conducted in the absence of any commercial or financial relationships that could be construed as a potential conflict of interest.

## Publisher’s Note

All claims expressed in this article are solely those of the authors and do not necessarily represent those of their affiliated organizations, or those of the publisher, the editors and the reviewers. Any product that may be evaluated in this article, or claim that may be made by its manufacturer, is not guaranteed or endorsed by the publisher.
