# Supplementary figures and images for: Protein Lactylation Critically Regulates Energy Metabolism in the Protozoan Parasite Trypanosoma brucei (part 1 of 2)
Source: Front Cell Dev Biol. 2021 Oct 14;9:719720. doi: 10.3389/fcell.2021.719720 (PMC8551762; doi:10.3389/fcell.2021.719720)

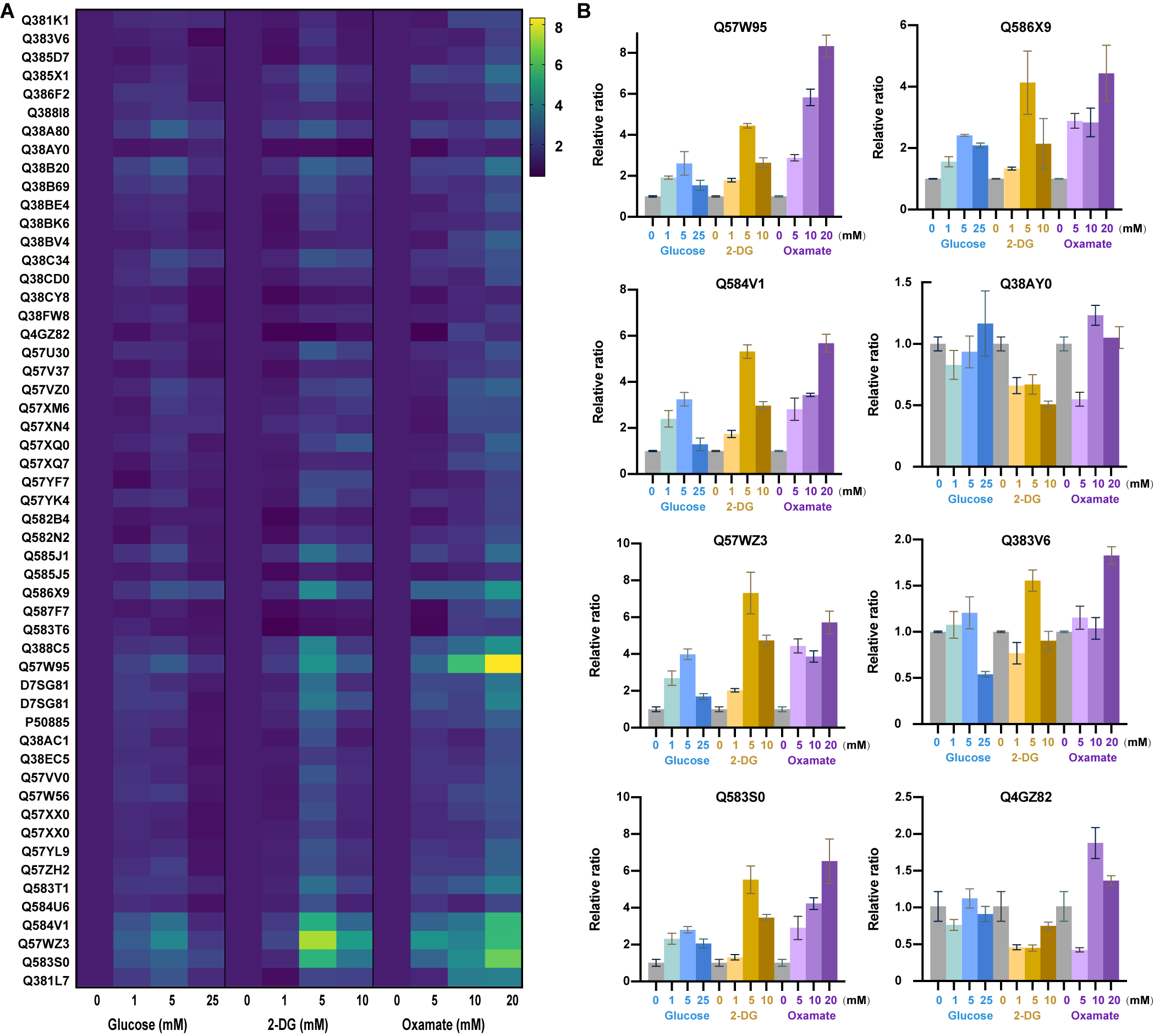

Supplement: Supplementary file 2 [file Data_Sheet_2.ZIP › Supplementary FIgures/Supplementary Figure 8.tif]

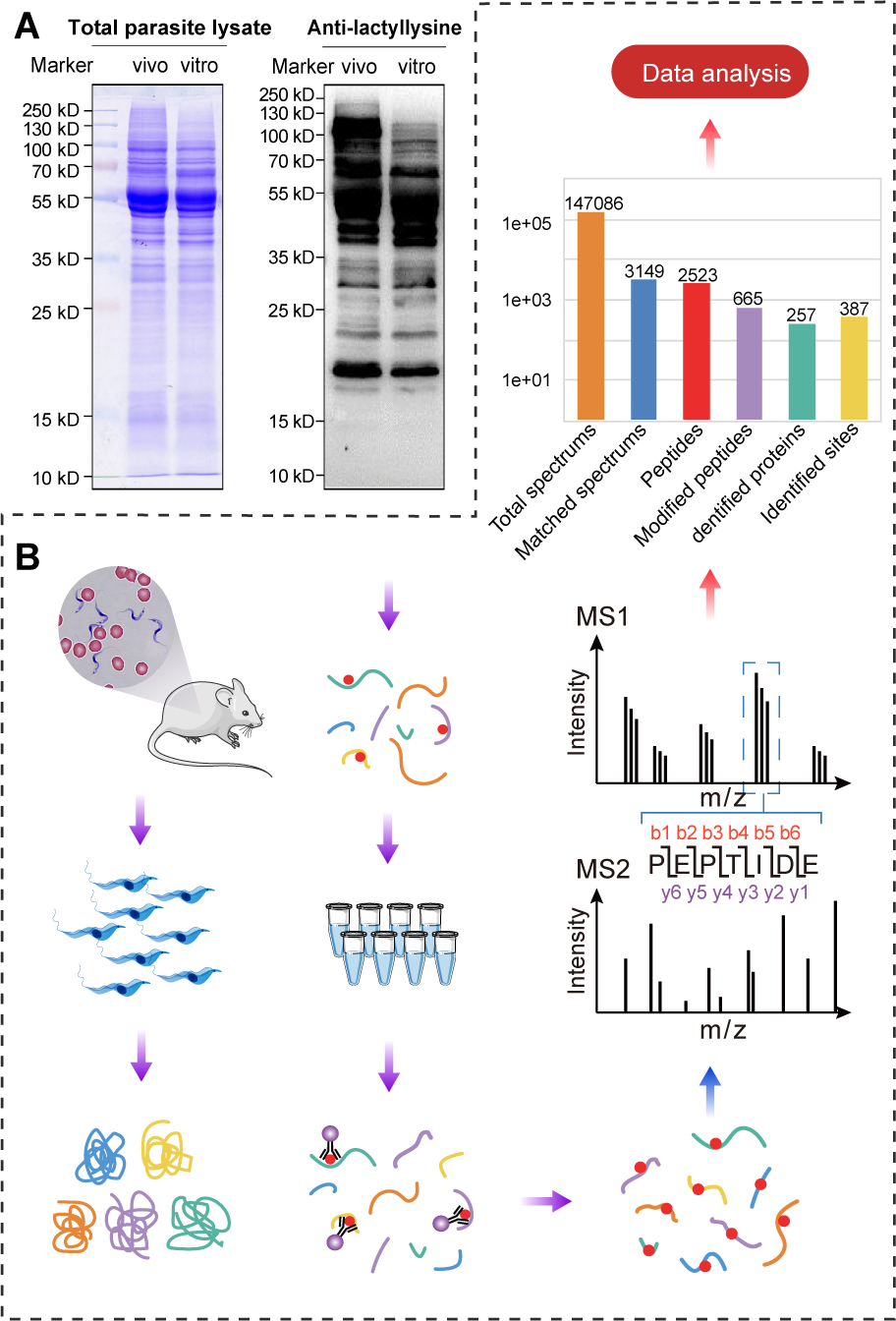

Supplement: Supplementary file 2 [file Data_Sheet_2.ZIP › Supplementary FIgures/Supplementary Figure 1.tif]

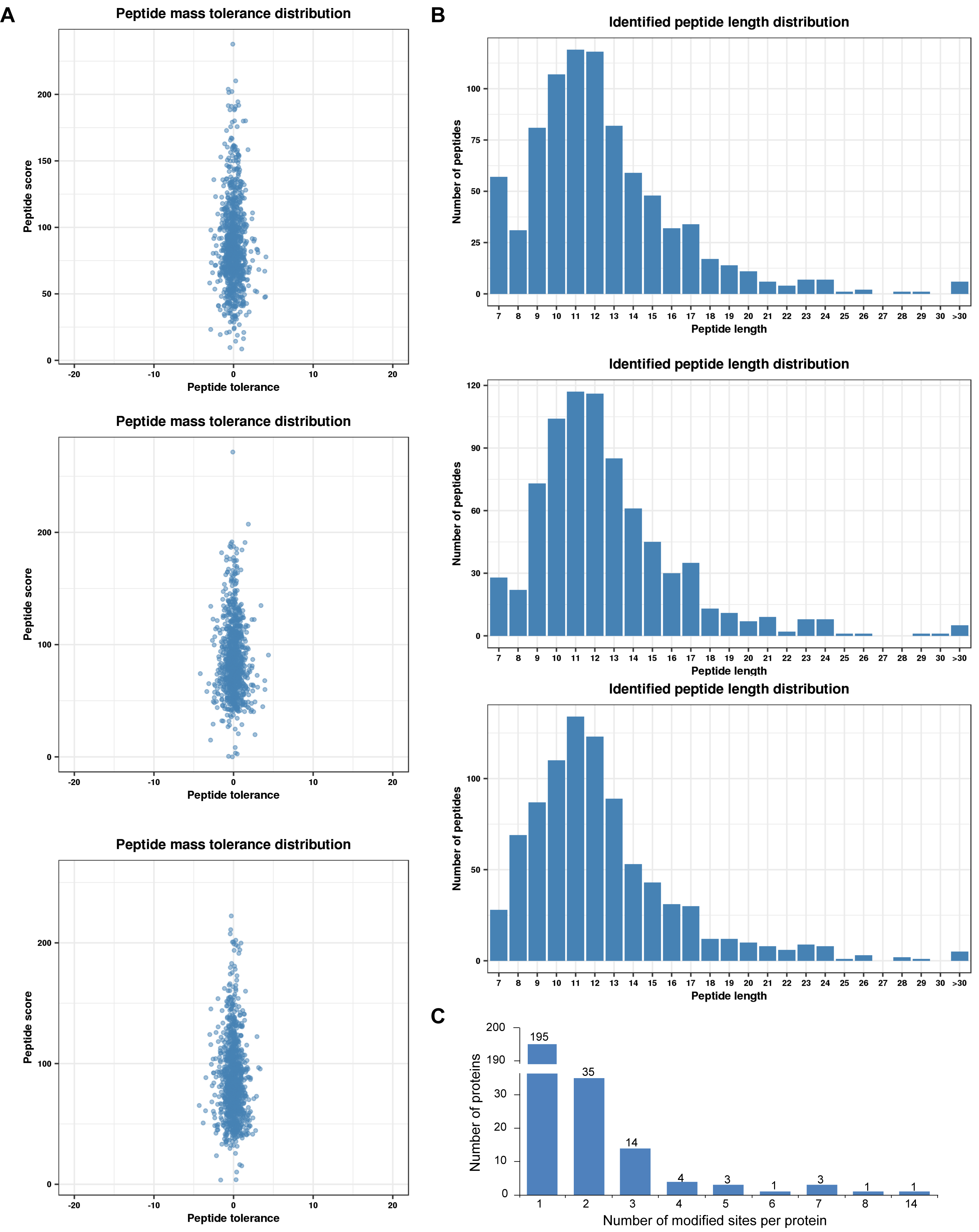

Supplement: Supplementary file 2 [file Data_Sheet_2.ZIP › Supplementary FIgures/Supplementary Figure 2.tif]

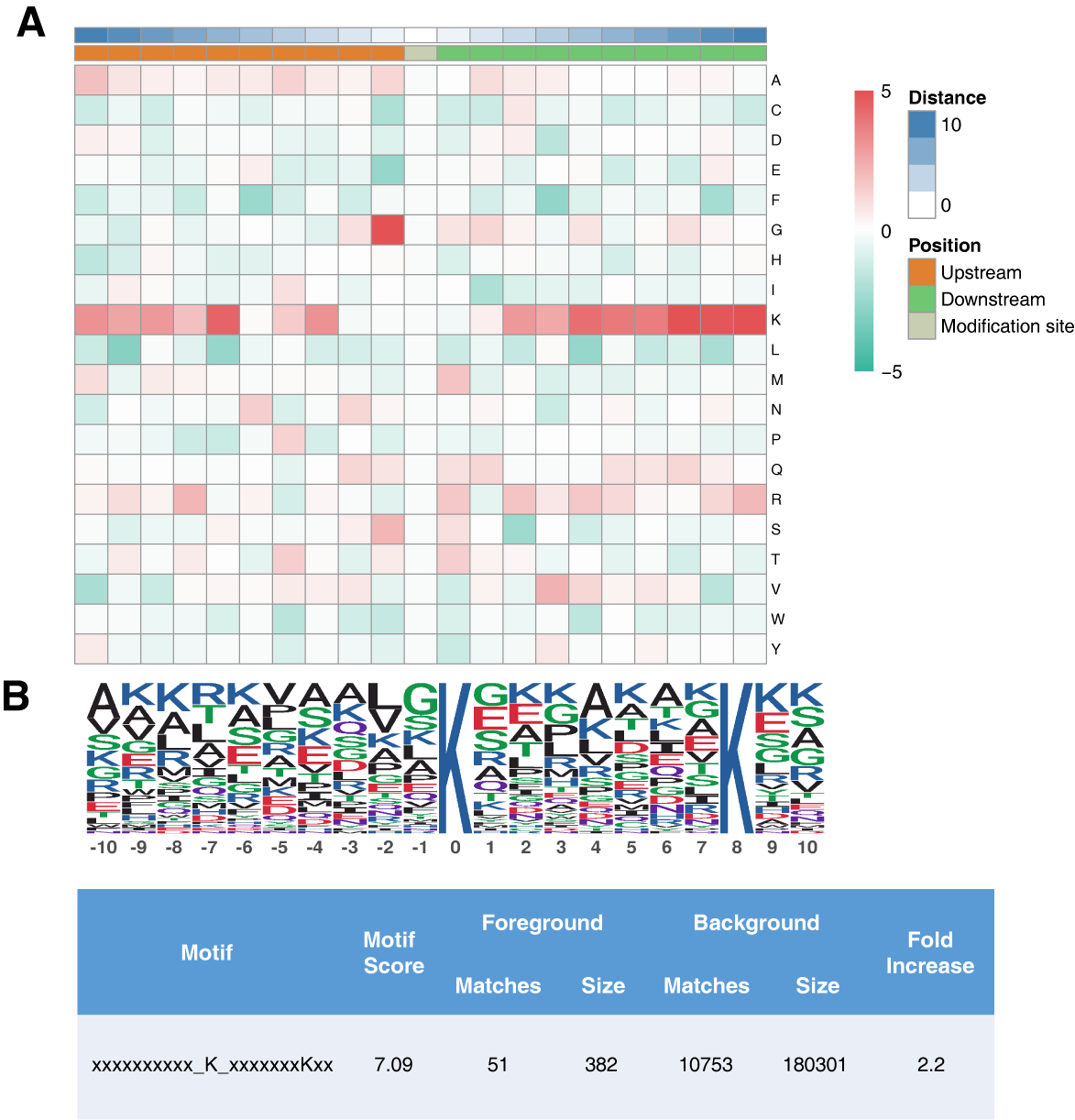

Supplement: Supplementary file 2 [file Data_Sheet_2.ZIP › Supplementary FIgures/Supplementary Figure 3.tif]

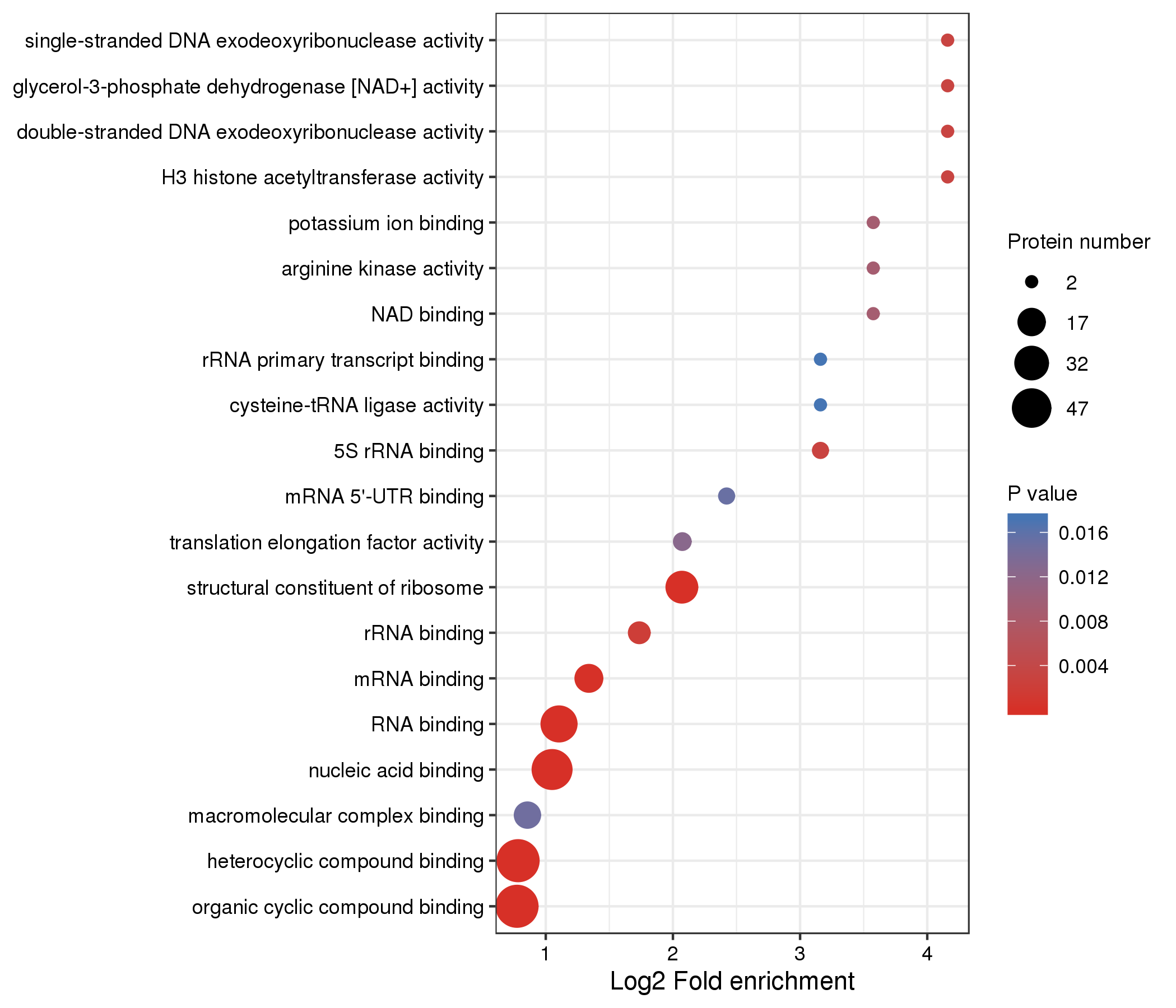

Supplement: Supplementary file 2 [file Data_Sheet_2.ZIP › Supplementary FIgures/Supplementary Figure 7.tif]

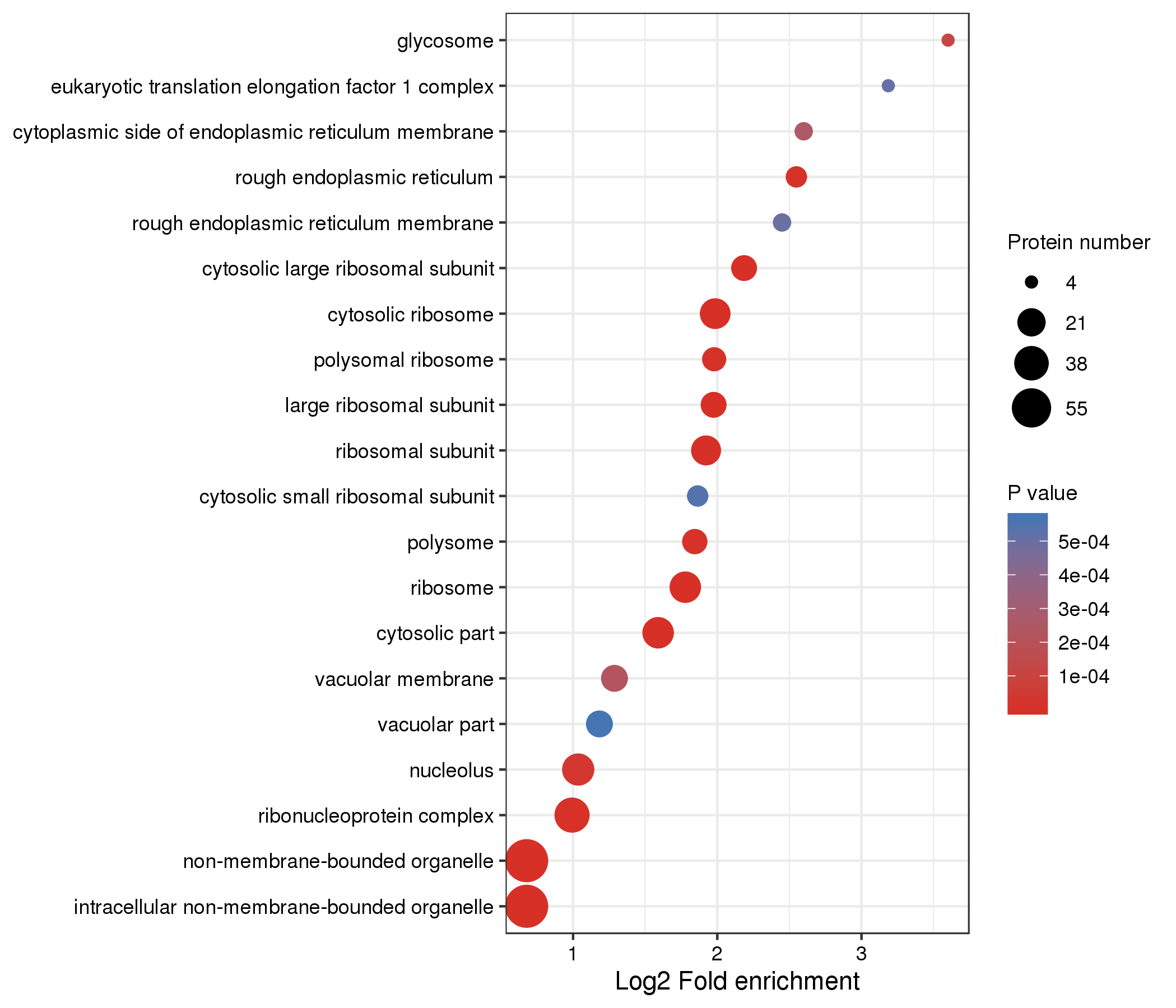

Supplement: Supplementary file 2 [file Data_Sheet_2.ZIP › Supplementary FIgures/Supplementary Figure 6.tif]

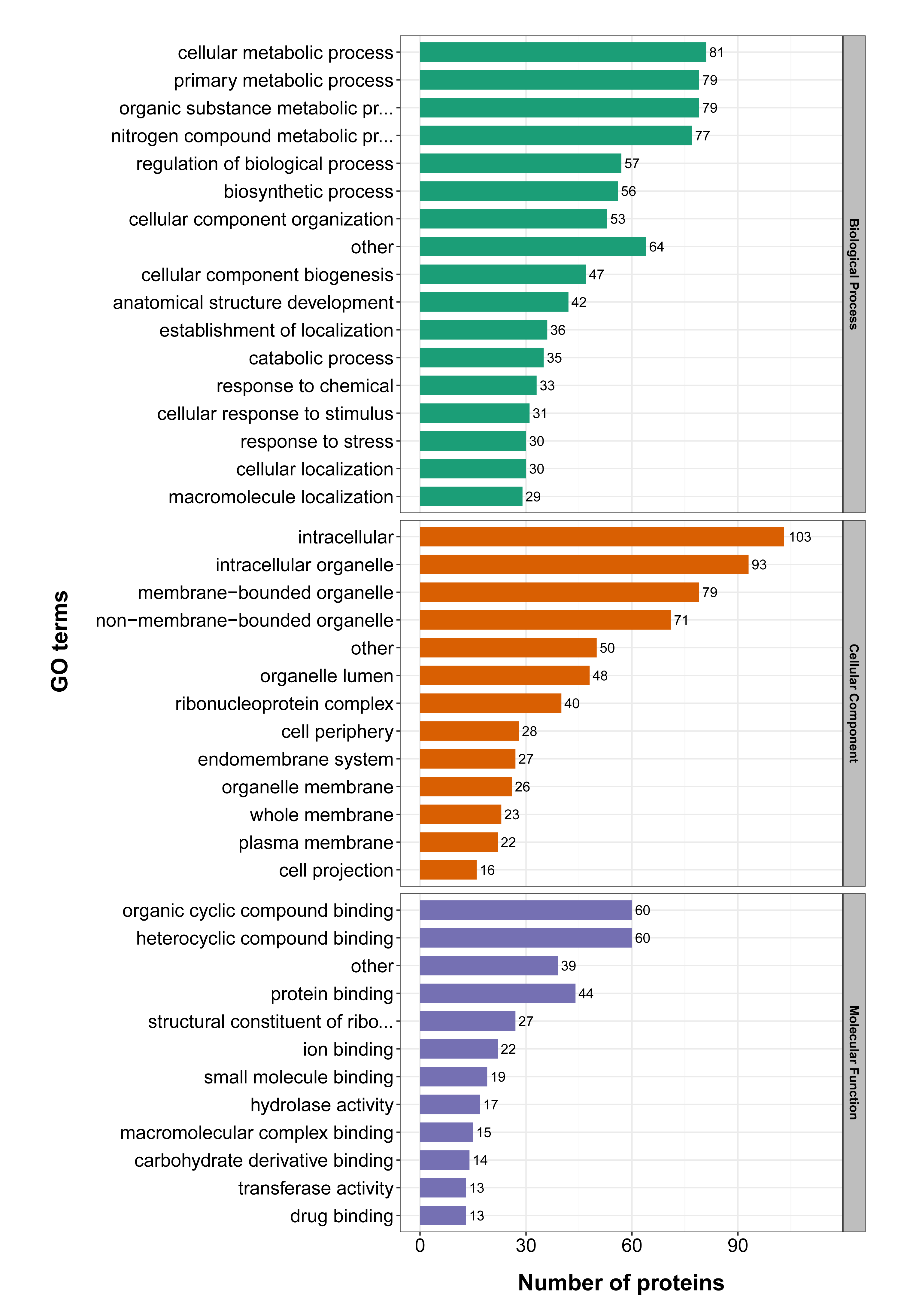

Supplement: Supplementary file 2 [file Data_Sheet_2.ZIP › Supplementary FIgures/Supplementary Figure 4.tif]

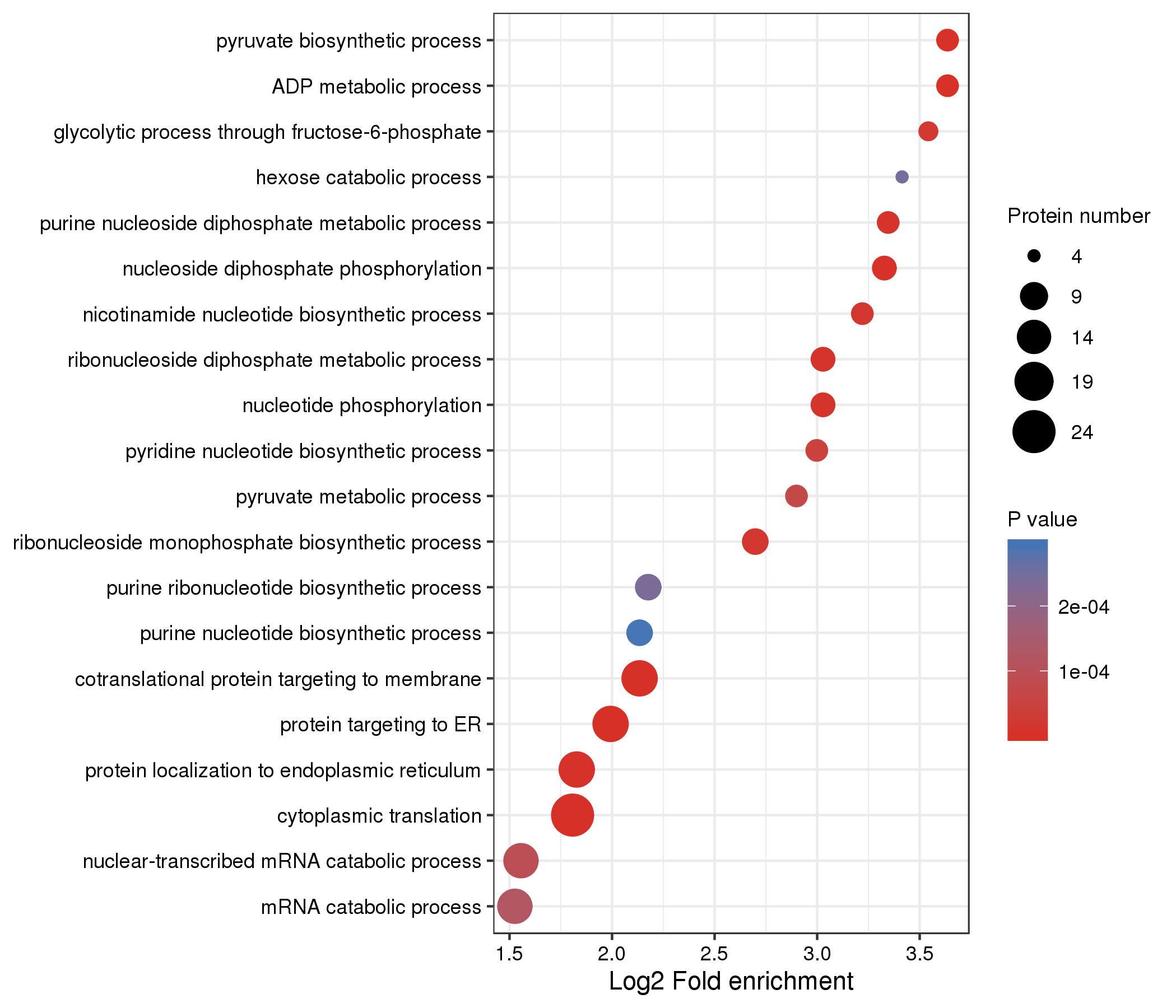

Supplement: Supplementary file 2 [file Data_Sheet_2.ZIP › Supplementary FIgures/Supplementary Figure 5.tif]

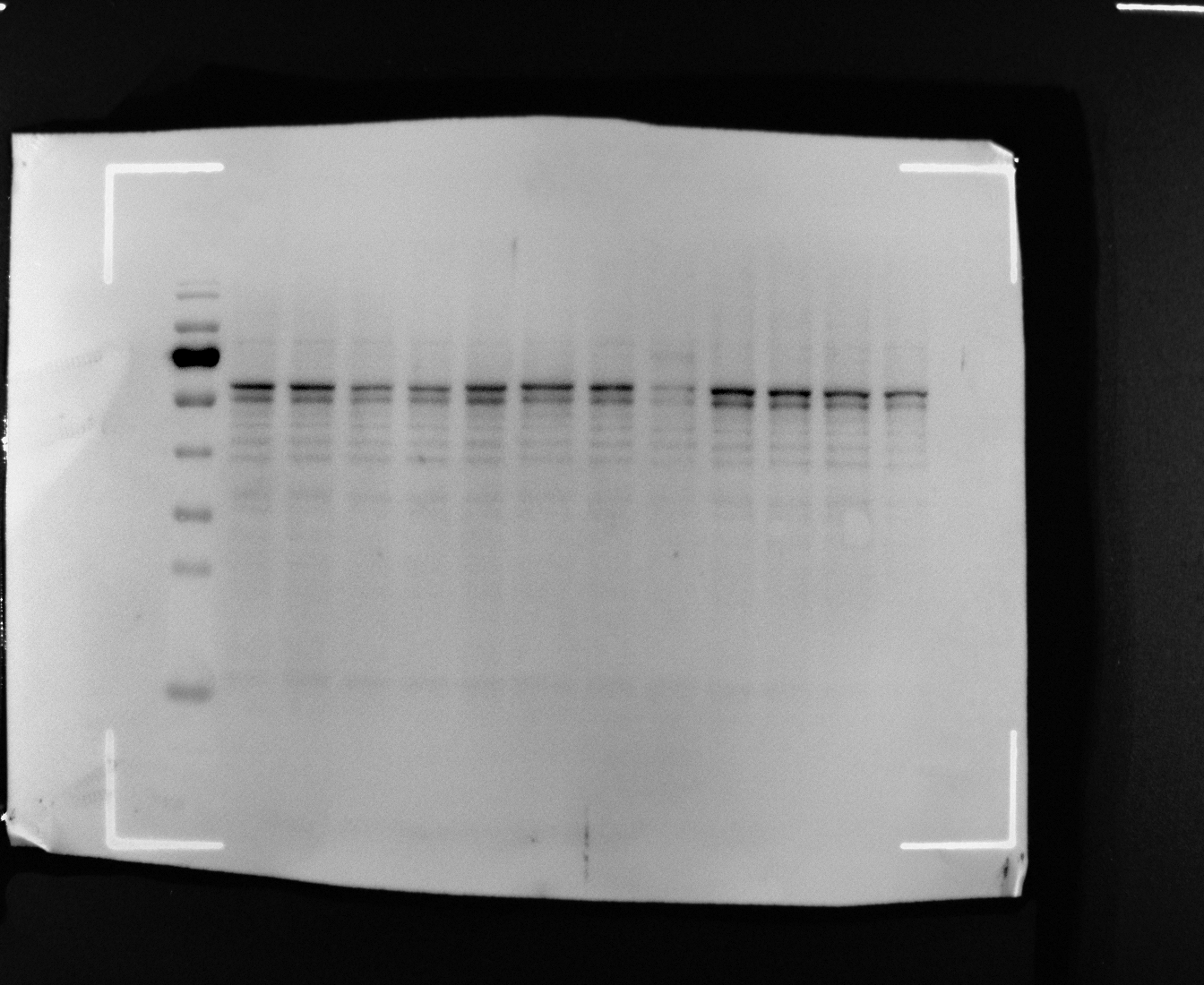

Supplement: Supplementary file 3 [file Data_Sheet_3.ZIP › Original Data 1/04 Western Blotting/01 Total T. brucei lysates/anti-Kla [Glucose (0,1,5,25) 2-DG (0,1,5,10) Oxamate (0,5,10,20)].tif]

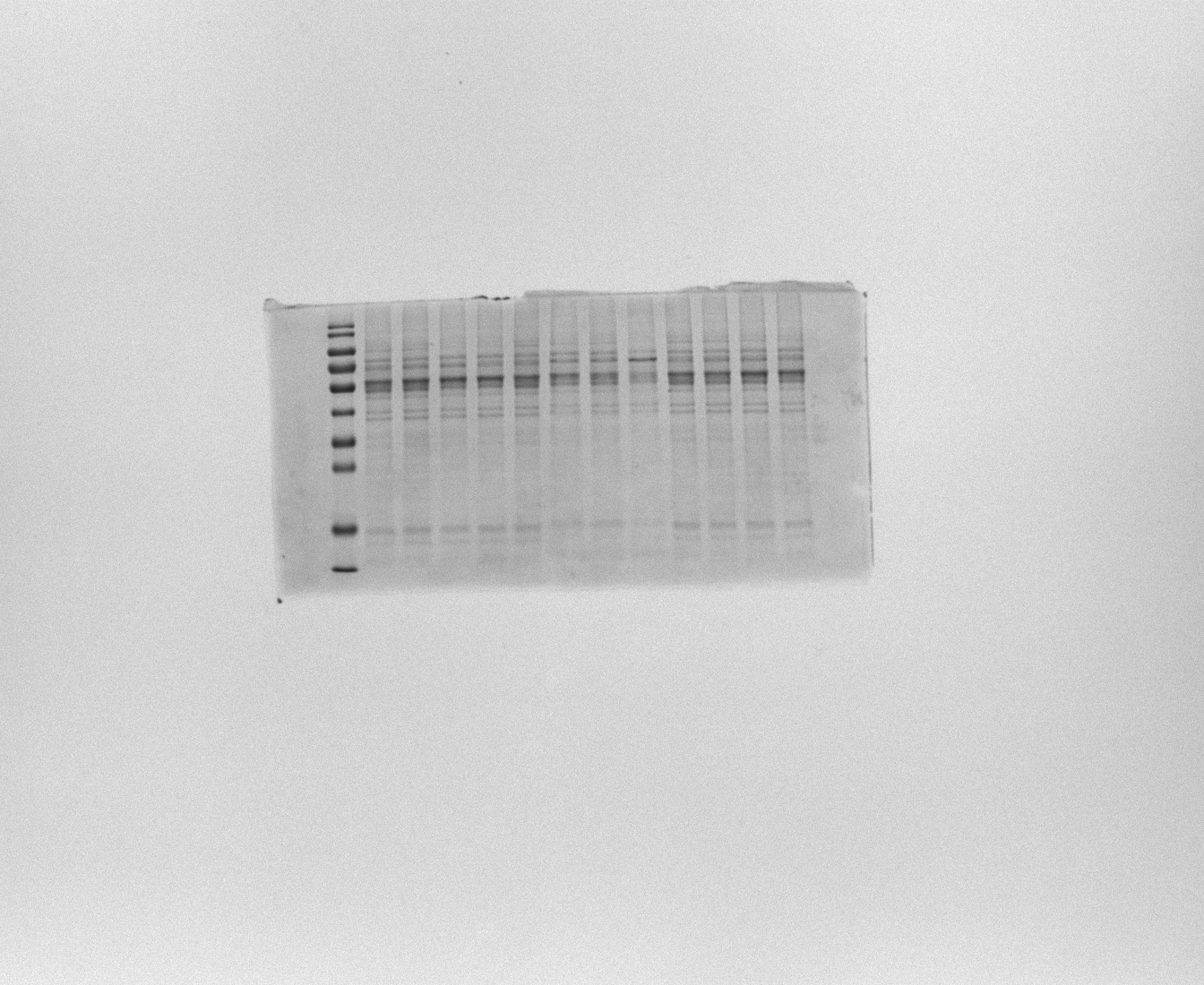

Supplement: Supplementary file 3 [file Data_Sheet_3.ZIP › Original Data 1/04 Western Blotting/01 Total T. brucei lysates/SDS-Page [Glucose (0,1,5,25)+2-DG (0,1,5,10)+Oxamate (0,5,10,20)].tif]

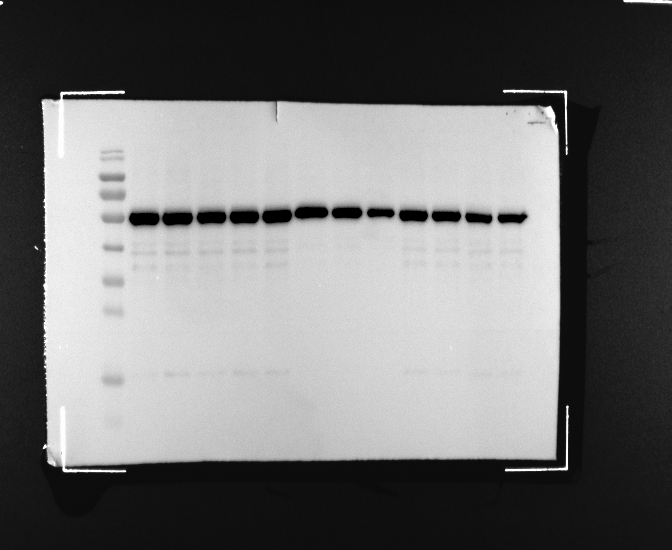

Supplement: Supplementary file 3 [file Data_Sheet_3.ZIP › Original Data 1/04 Western Blotting/01 Total T. brucei lysates/anti-Kac [Glucose(0,1,5,25), the last 8 lanes have been removed].tif]

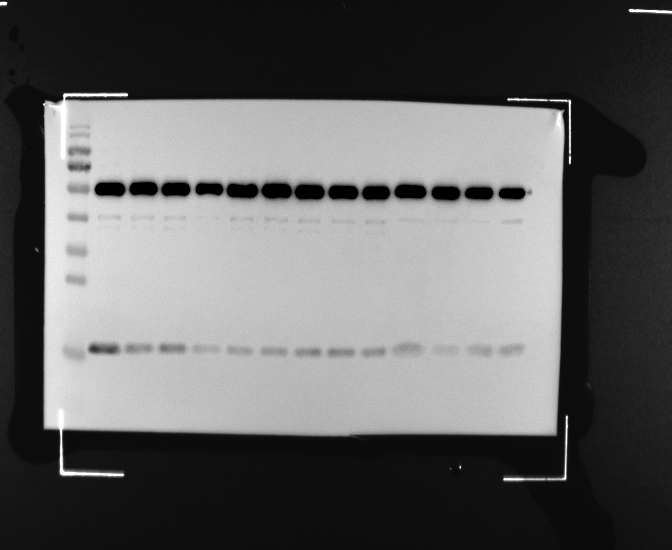

Supplement: Supplementary file 3 [file Data_Sheet_3.ZIP › Original Data 1/04 Western Blotting/01 Total T. brucei lysates/anti-Kac [The last 8 lanes are∩╝Ü2-DG(0,1,5,10)+Oxamate(0,5,10,20)].tif]

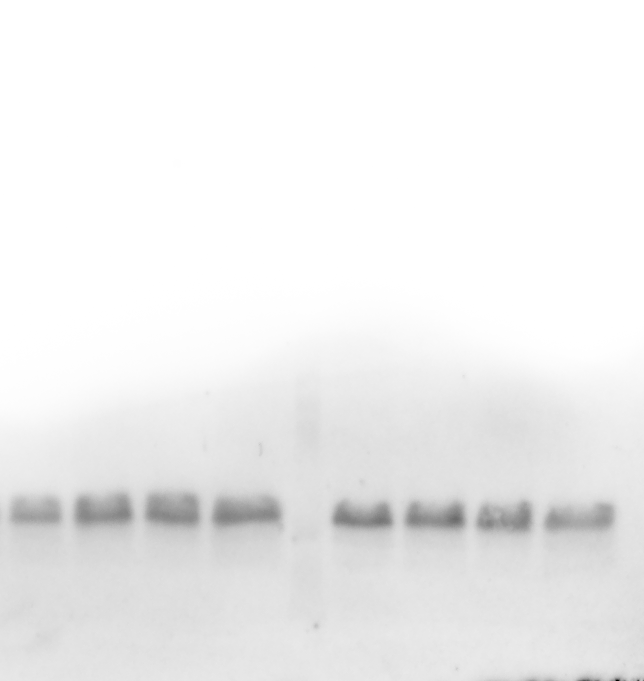

Supplement: Supplementary file 3 [file Data_Sheet_3.ZIP › Original Data 1/04 Western Blotting/03 Histone H3/anti-H3 [2-DG(10,5,1,0)+Oxamate(0,5,10,20)].tif]

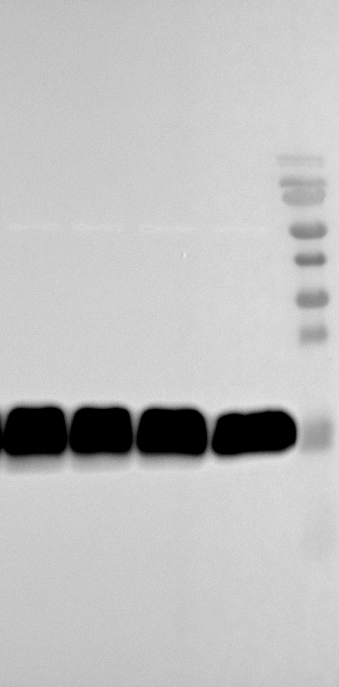

Supplement: Supplementary file 3 [file Data_Sheet_3.ZIP › Original Data 1/04 Western Blotting/03 Histone H3/anti-Kac [Glucose(0,1,5,25)].tif]

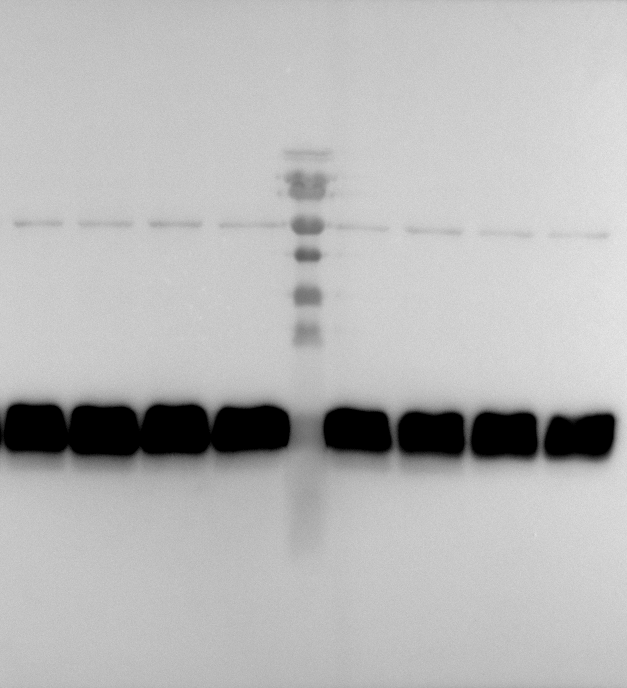

Supplement: Supplementary file 3 [file Data_Sheet_3.ZIP › Original Data 1/04 Western Blotting/03 Histone H3/anti-Kac [2-DG(10,5,1,0)+Oxamate(0,5,10,20)].tif]

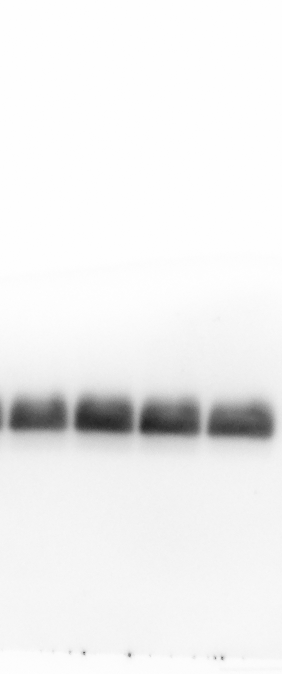

Supplement: Supplementary file 3 [file Data_Sheet_3.ZIP › Original Data 1/04 Western Blotting/03 Histone H3/anti-Kla [Glucose(0,1,5,25)].tif]

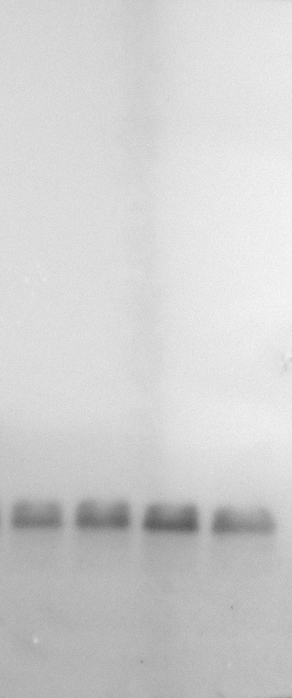

Supplement: Supplementary file 3 [file Data_Sheet_3.ZIP › Original Data 1/04 Western Blotting/03 Histone H3/anti-H3 [Glucose(0,1,5,25)].tif]

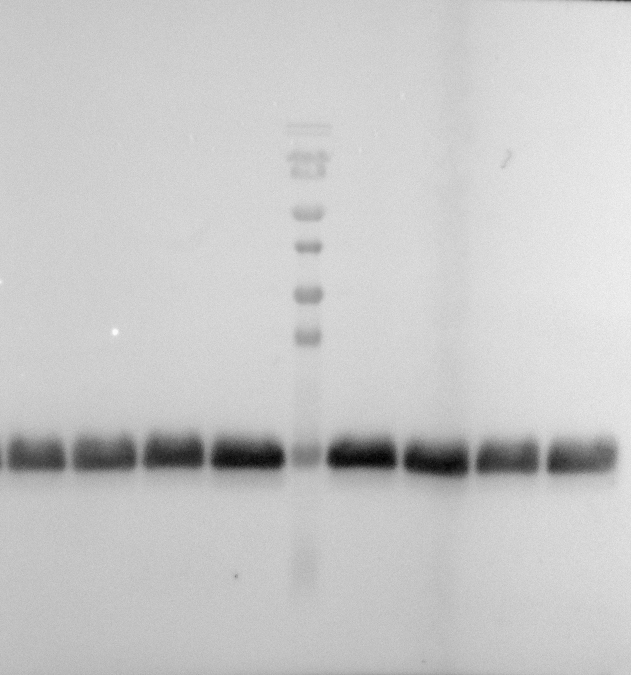

Supplement: Supplementary file 3 [file Data_Sheet_3.ZIP › Original Data 1/04 Western Blotting/03 Histone H3/anti-Kla [2-DG(10,5,1,0)+Oxamate(0,5,10,20)].tif]

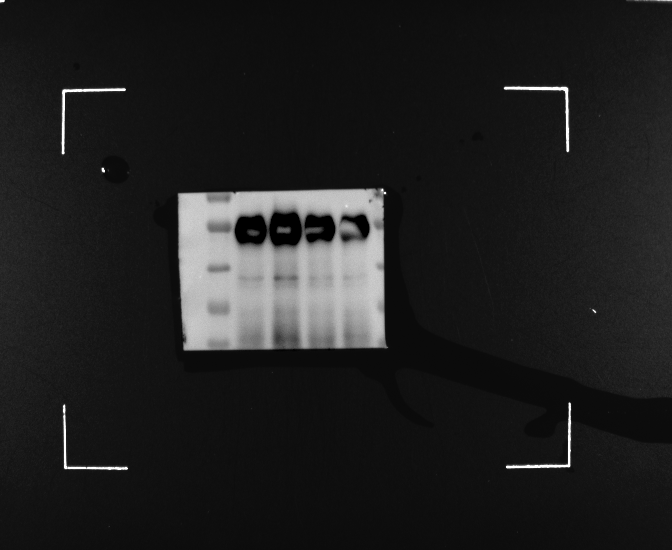

Supplement: Supplementary file 3 [file Data_Sheet_3.ZIP › Original Data 1/04 Western Blotting/02 GAPDH/anti-Kla [Oxamate(0,5,10,20),the right 4 lanes].tif]

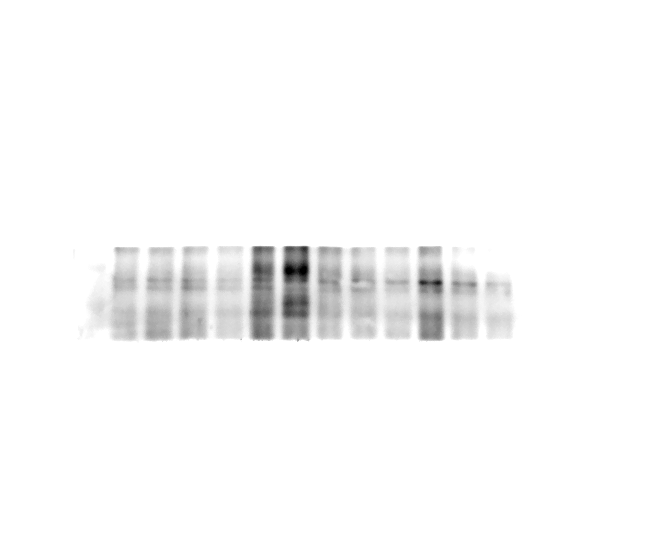

Supplement: Supplementary file 3 [file Data_Sheet_3.ZIP › Original Data 1/04 Western Blotting/02 GAPDH/anti-Kla [Glucose(0,1,5,25),the right 4 lanes].tif]

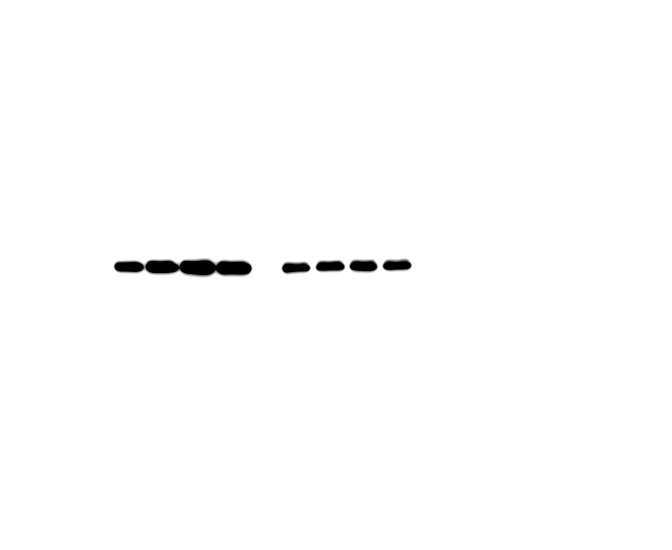

Supplement: Supplementary file 3 [file Data_Sheet_3.ZIP › Original Data 1/04 Western Blotting/02 GAPDH/anti-GAPDH [Glucose(0,1,5,25), the right 4 lanes].tif]

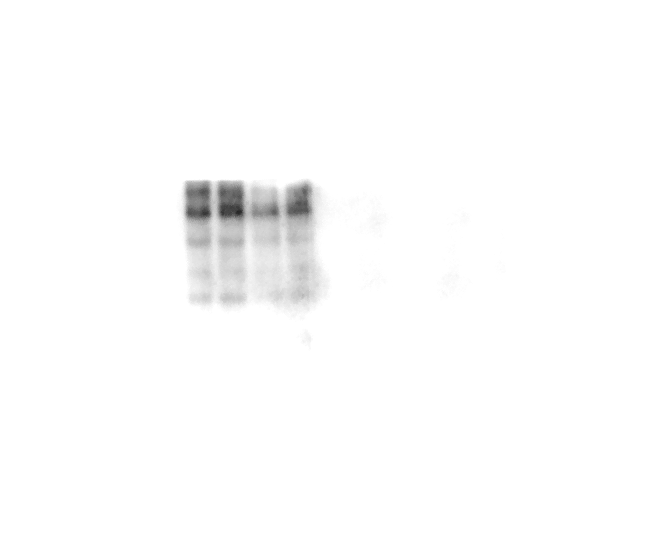

Supplement: Supplementary file 3 [file Data_Sheet_3.ZIP › Original Data 1/04 Western Blotting/02 GAPDH/anti-Kac [Oxamate(0,5,10,20)].tif]

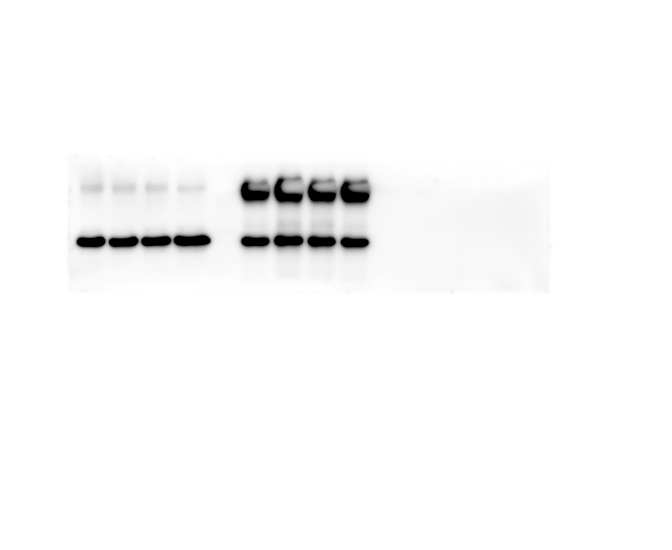

Supplement: Supplementary file 3 [file Data_Sheet_3.ZIP › Original Data 1/04 Western Blotting/02 GAPDH/anti-GAPDH [Oxamate(0,5,10,20),the right 4 lanes].tif]

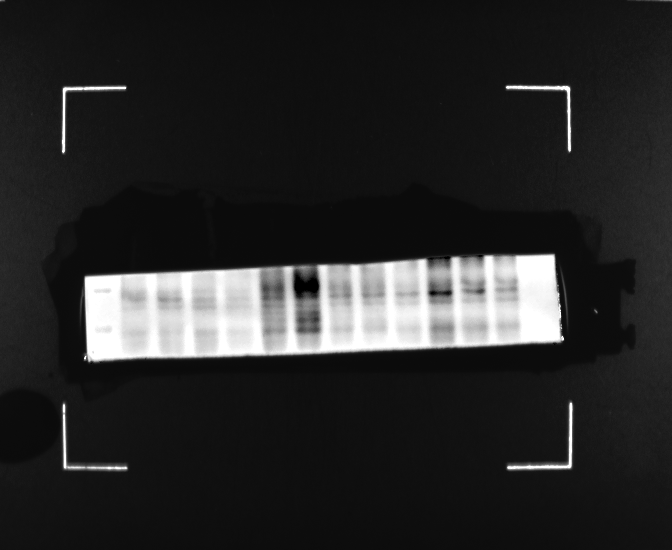

Supplement: Supplementary file 3 [file Data_Sheet_3.ZIP › Original Data 1/04 Western Blotting/02 GAPDH/anti-Kac [Glucose(0,1,5,25), the left 4 lanes].tif]

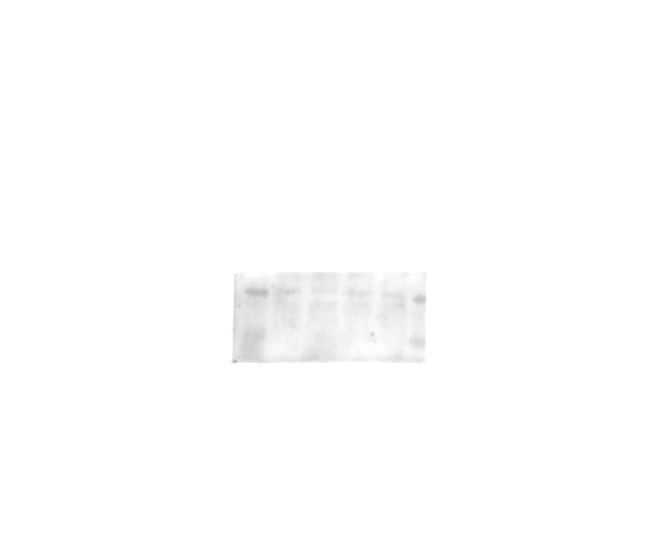

Supplement: Supplementary file 3 [file Data_Sheet_3.ZIP › Original Data 1/04 Western Blotting/02 GAPDH/anti-Kac [2-DG(0,1,5,25].tif]

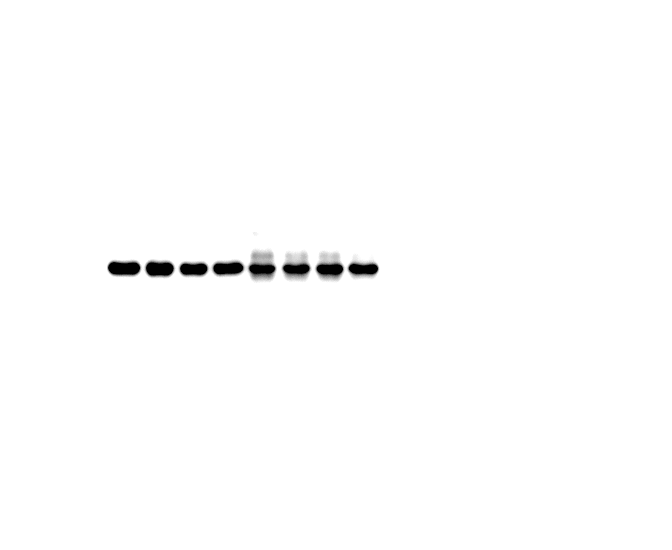

Supplement: Supplementary file 3 [file Data_Sheet_3.ZIP › Original Data 1/04 Western Blotting/02 GAPDH/anti-GAPDH [2-DG (0,1,5,10),the right 4 lanes].tif]

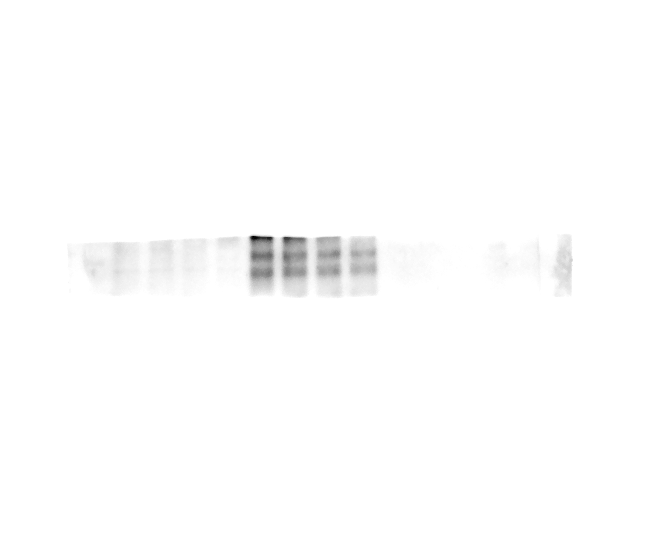

Supplement: Supplementary file 3 [file Data_Sheet_3.ZIP › Original Data 1/04 Western Blotting/02 GAPDH/anti-Kla [2-DG (0,1,5,10),the right 4 lanes].tif]

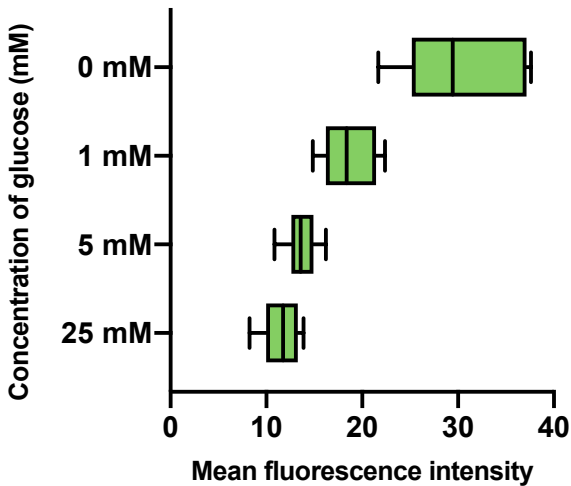

Supplement: Supplementary file 5 [file Data_Sheet_5.ZIP › Original Data 3-IFA/Fluorescence intensity statistics/Glucose.pdf]

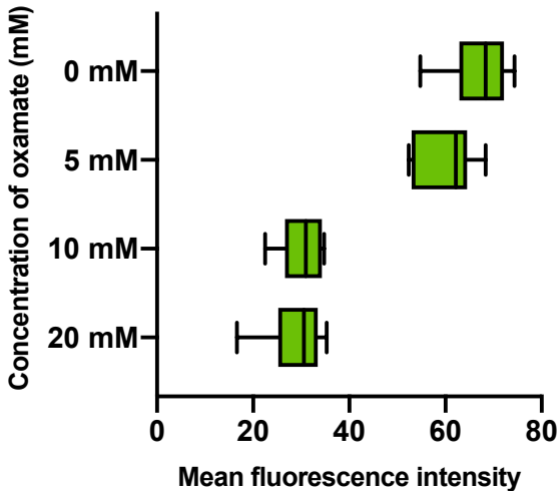

Supplement: Supplementary file 5 [file Data_Sheet_5.ZIP › Original Data 3-IFA/Fluorescence intensity statistics/Oxamate.pdf]

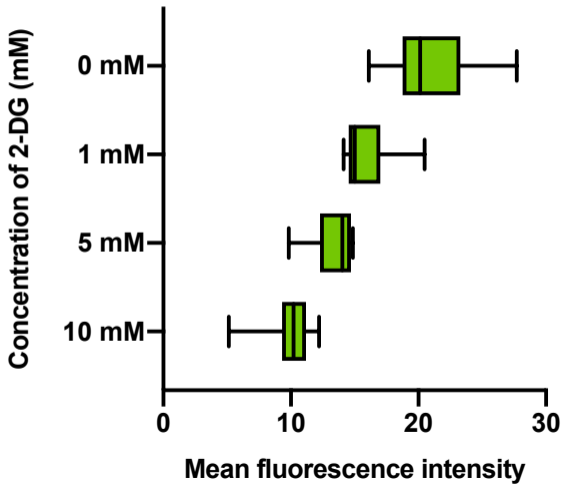

Supplement: Supplementary file 5 [file Data_Sheet_5.ZIP › Original Data 3-IFA/Fluorescence intensity statistics/2-DG.pdf]

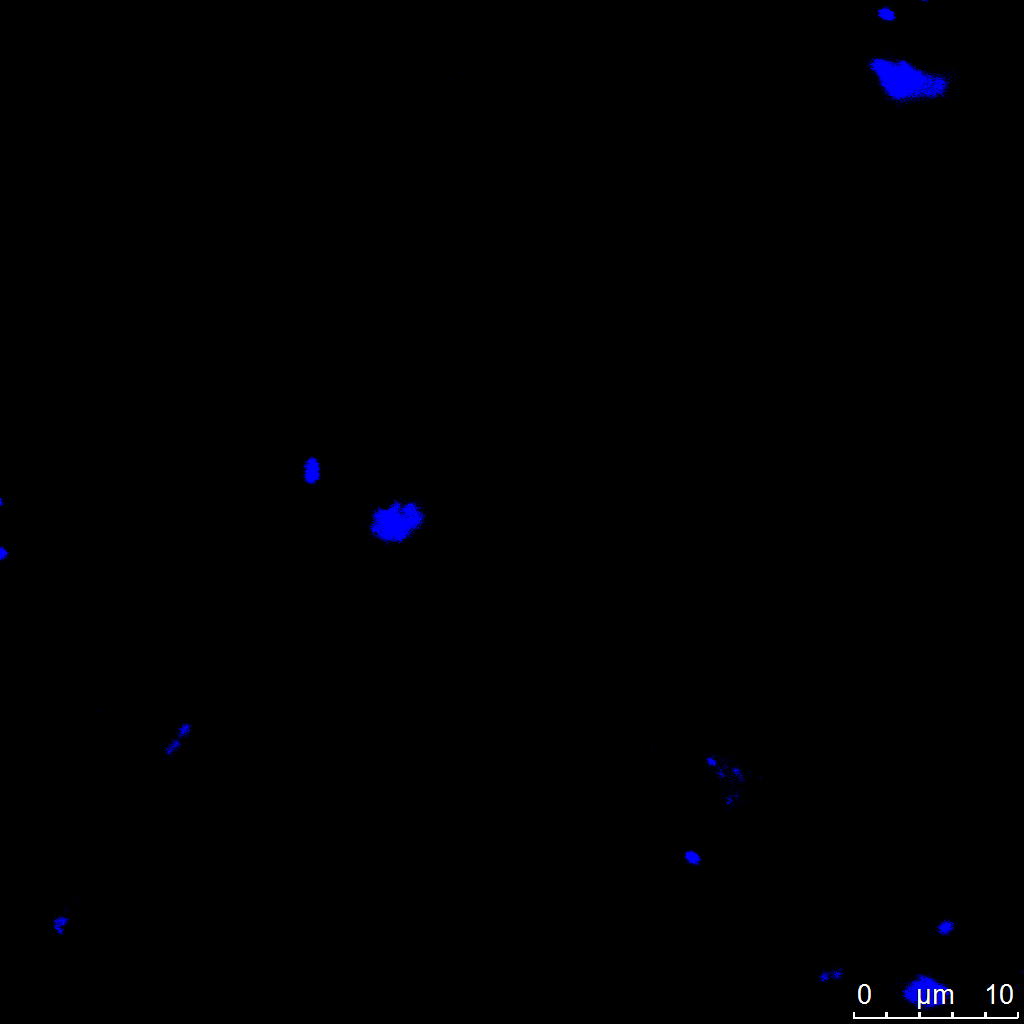

Supplement: Supplementary file 5 [file Data_Sheet_5.ZIP › Original Data 3-IFA/03 Oxamate/5 mM/Project20200710_0711-S5-2_z0_ch00.tif]

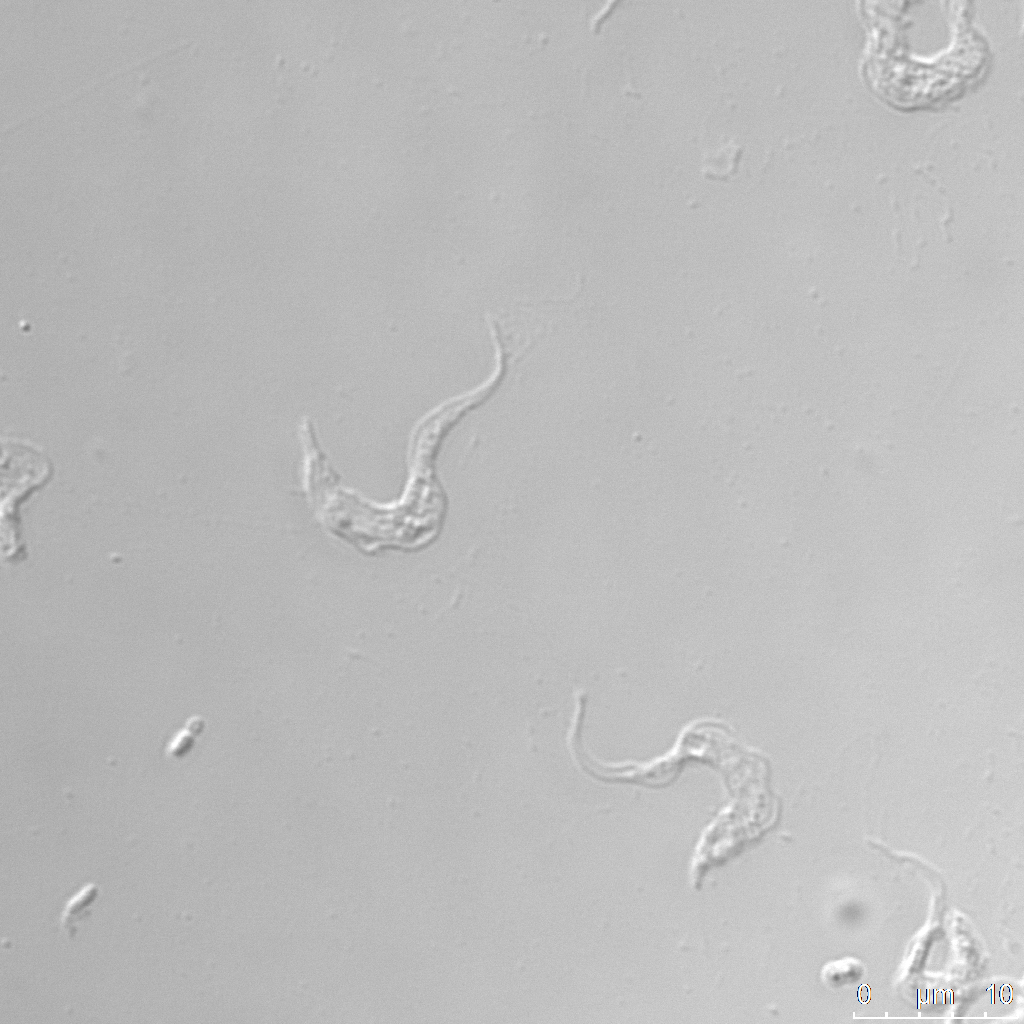

Supplement: Supplementary file 5 [file Data_Sheet_5.ZIP › Original Data 3-IFA/03 Oxamate/5 mM/Project20200710_0711-S5-2_z0_ch03.tif]

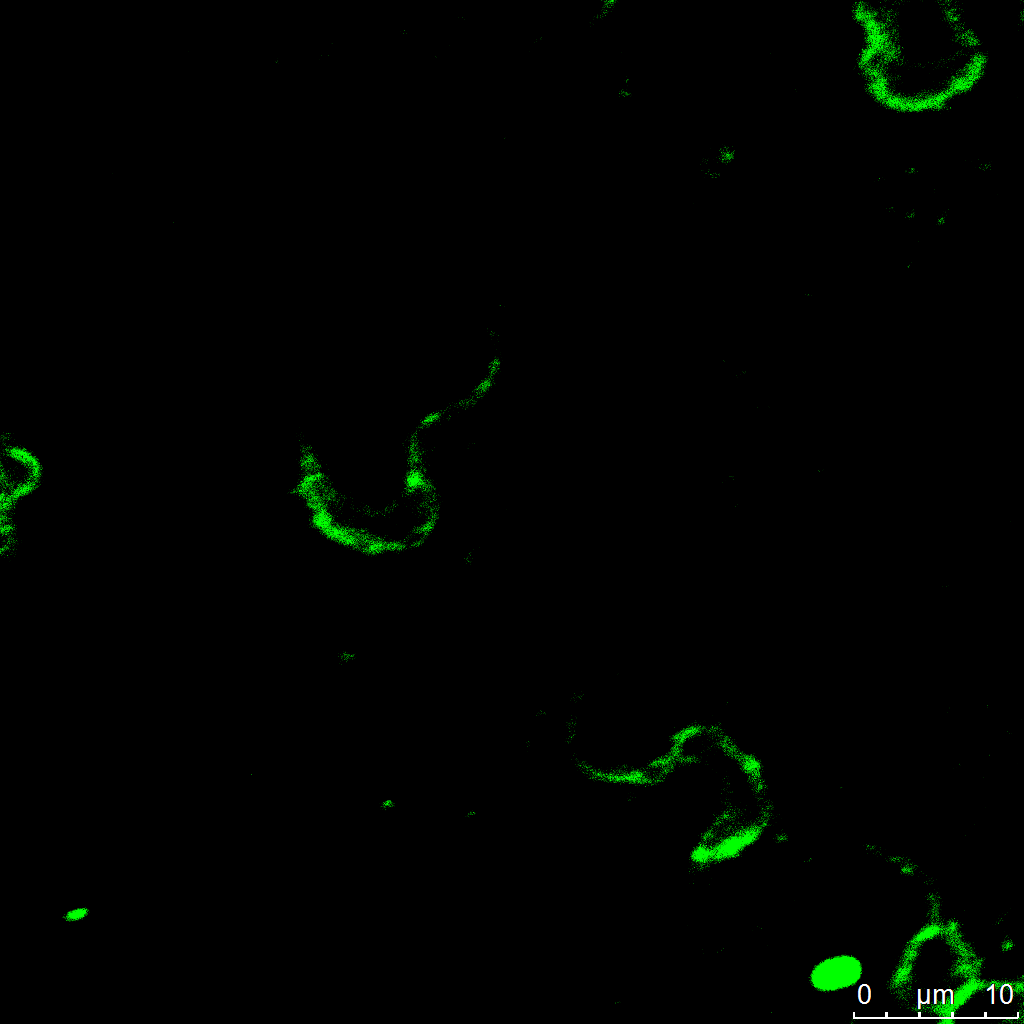

Supplement: Supplementary file 5 [file Data_Sheet_5.ZIP › Original Data 3-IFA/03 Oxamate/5 mM/Project20200710_0711-S5-2_z0_ch02.tif]

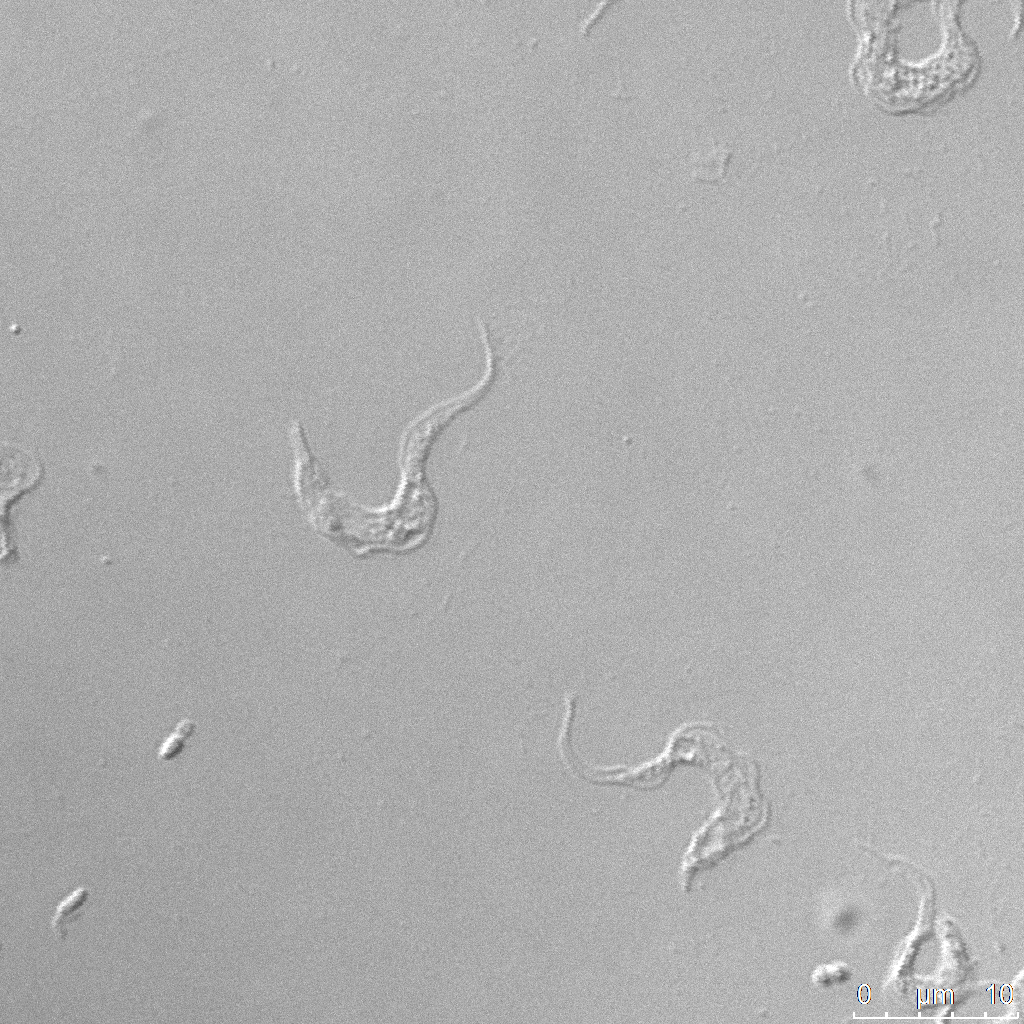

Supplement: Supplementary file 5 [file Data_Sheet_5.ZIP › Original Data 3-IFA/03 Oxamate/5 mM/Project20200710_0711-S0-2_z0_ch01.tif]

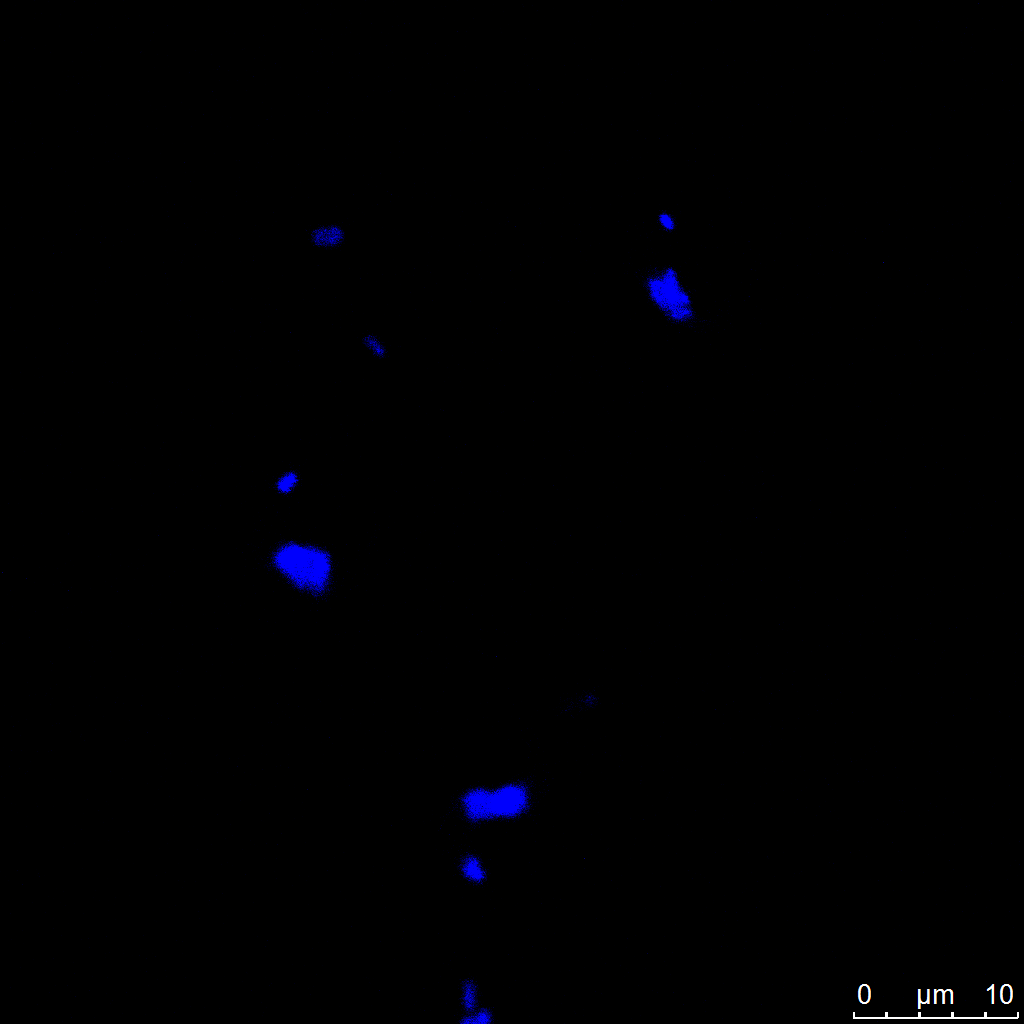

Supplement: Supplementary file 5 [file Data_Sheet_5.ZIP › Original Data 3-IFA/03 Oxamate/0 mM/Project20200710_0711-S0-2_z0_ch00.tif]

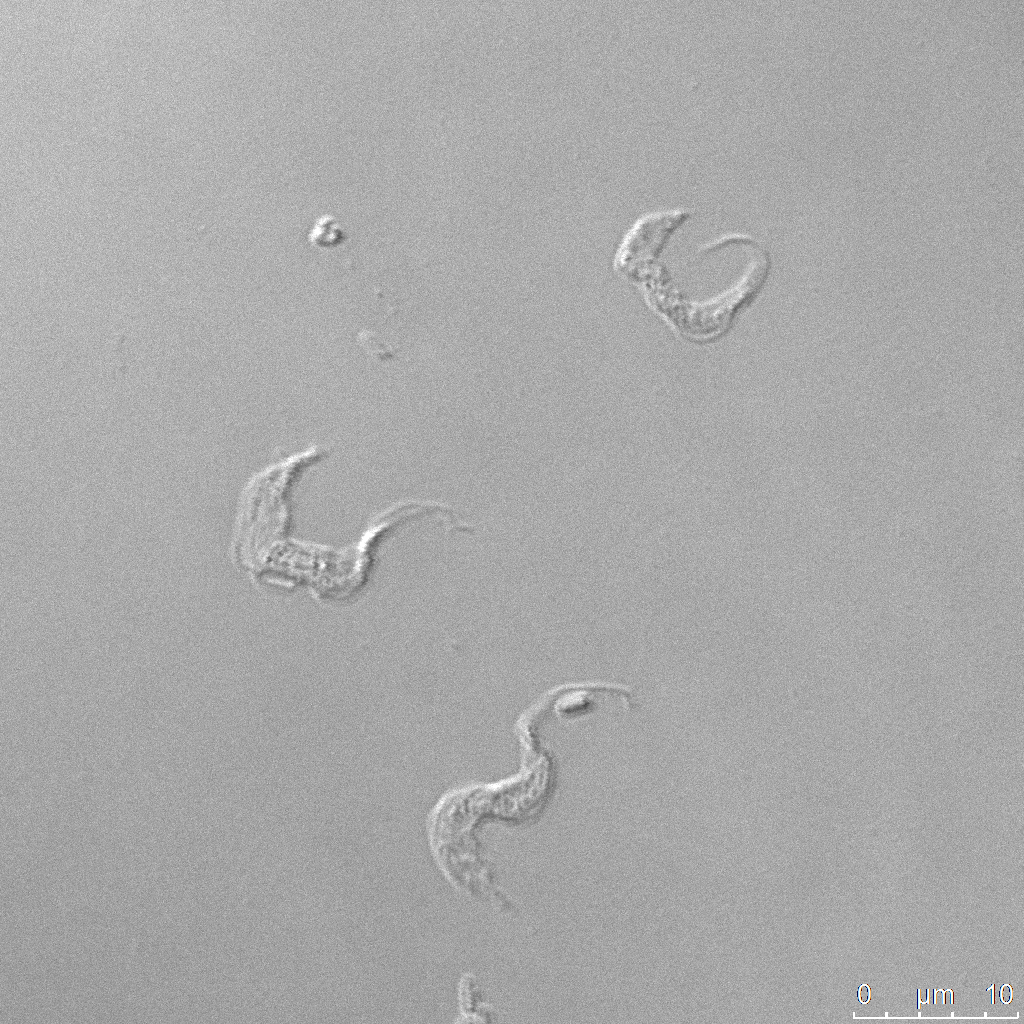

Supplement: Supplementary file 5 [file Data_Sheet_5.ZIP › Original Data 3-IFA/03 Oxamate/0 mM/Project20200710_0711-S0-2_z0_ch01.tif]

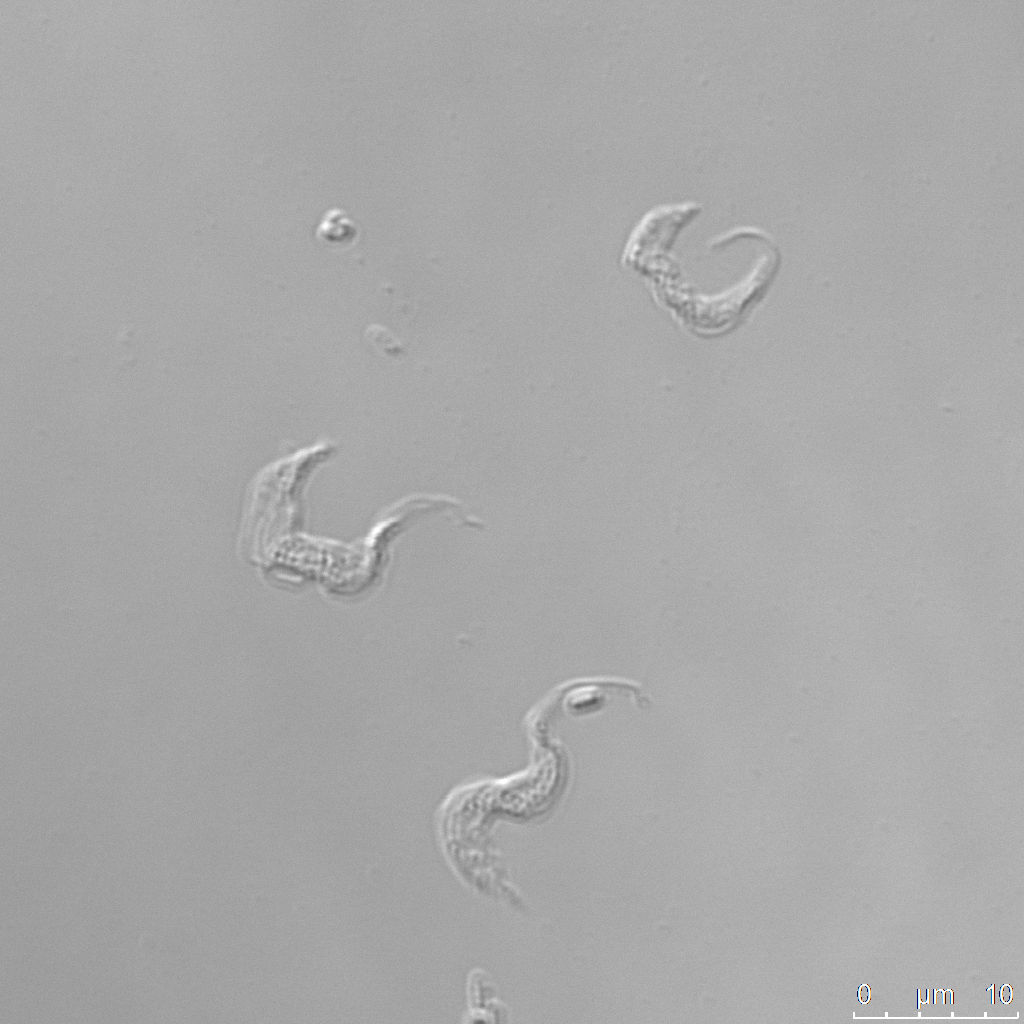

Supplement: Supplementary file 5 [file Data_Sheet_5.ZIP › Original Data 3-IFA/03 Oxamate/0 mM/Project20200710_0711-S0-2_z0_ch03.tif]

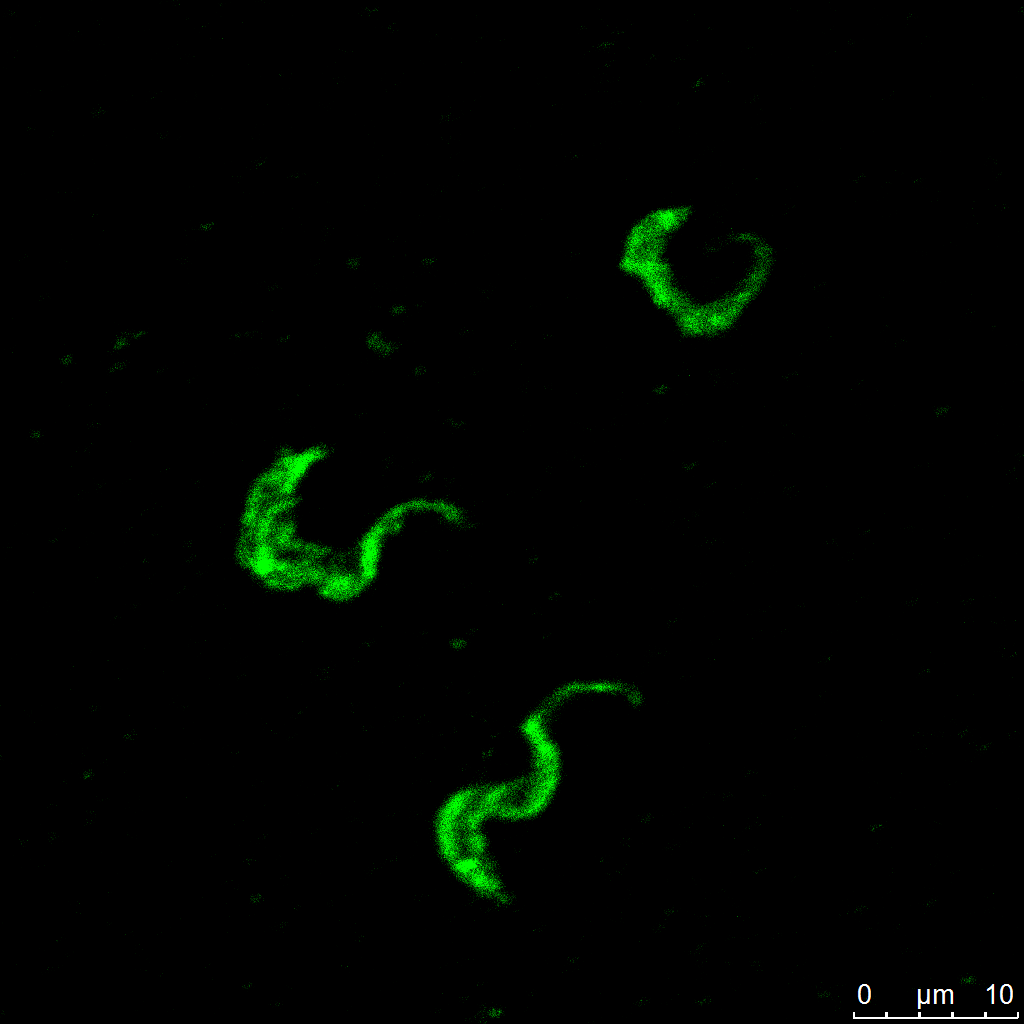

Supplement: Supplementary file 5 [file Data_Sheet_5.ZIP › Original Data 3-IFA/03 Oxamate/0 mM/Project20200710_0711-S0-2_z0_ch02.tif]

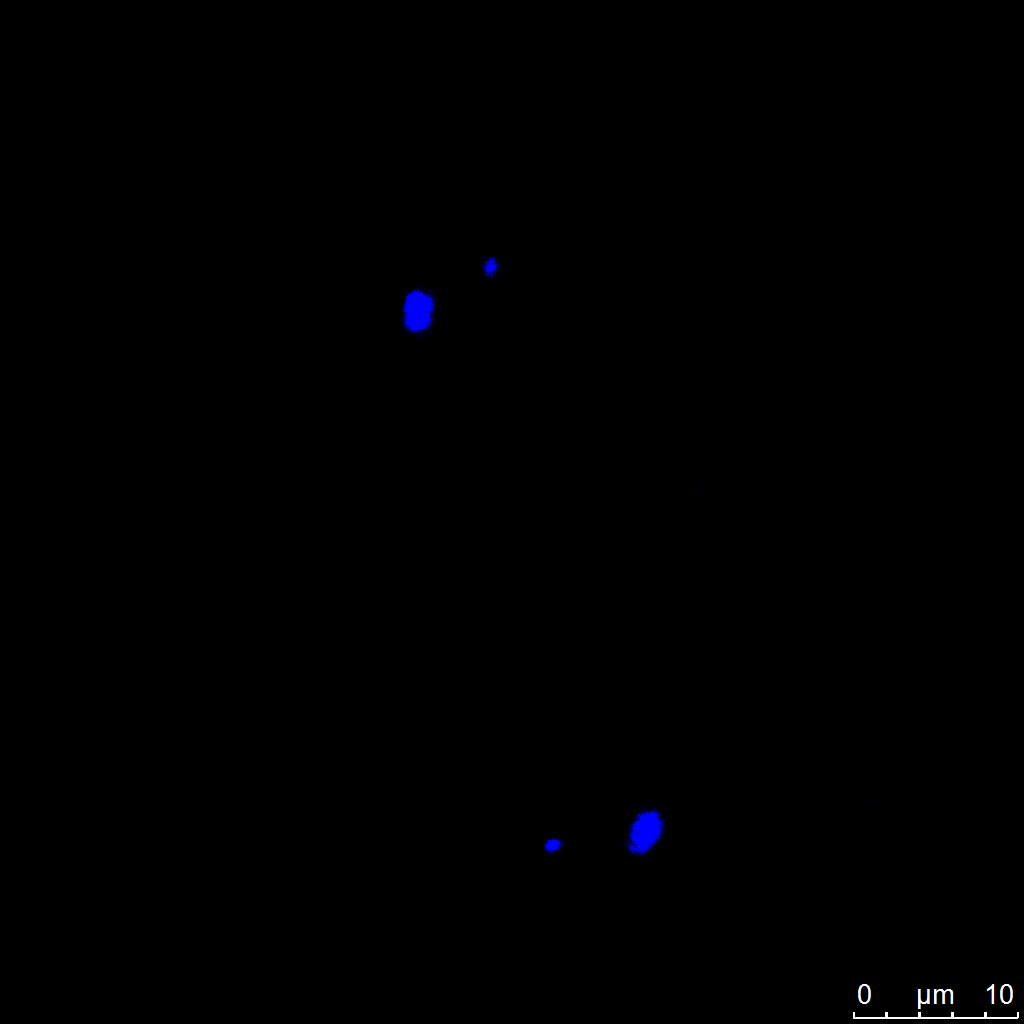

Supplement: Supplementary file 5 [file Data_Sheet_5.ZIP › Original Data 3-IFA/03 Oxamate/Negative/Project20200710_b-2_z0_ch00.tif]

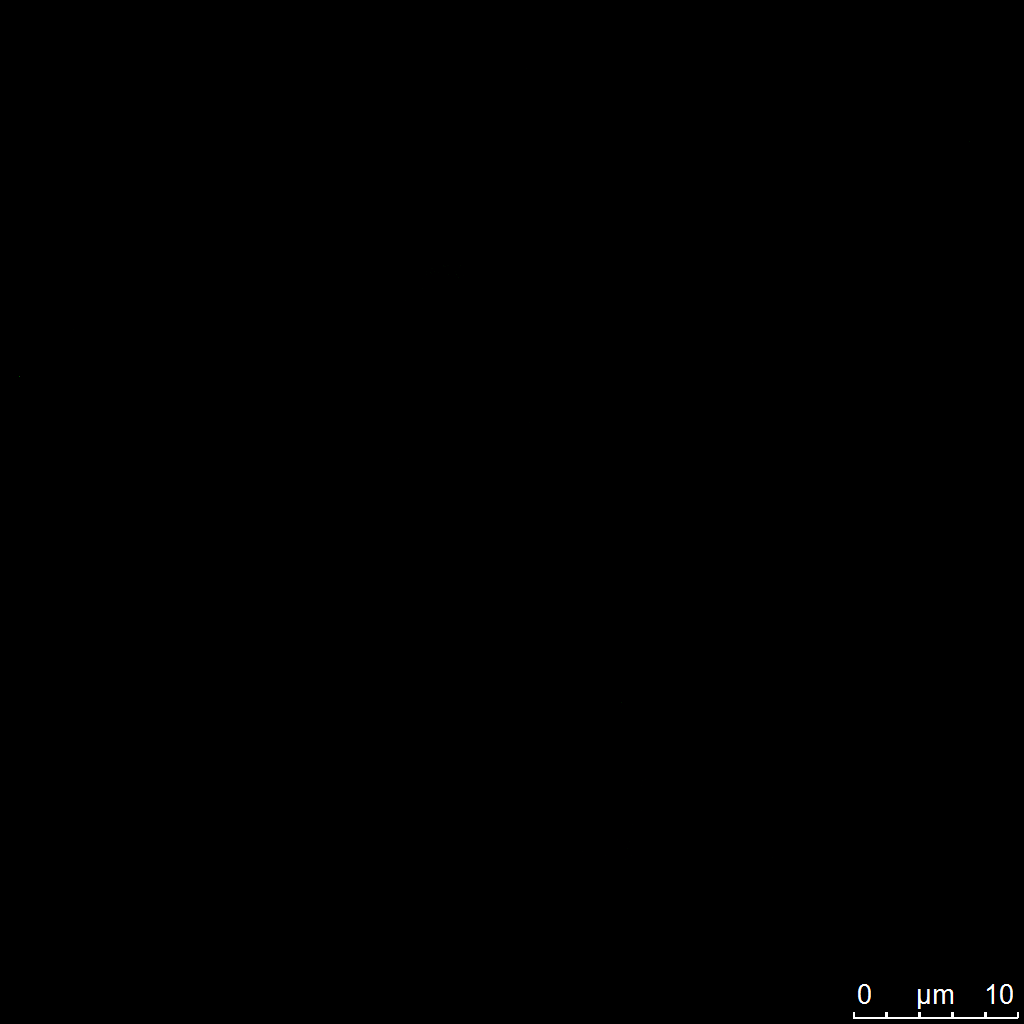

Supplement: Supplementary file 5 [file Data_Sheet_5.ZIP › Original Data 3-IFA/03 Oxamate/Negative/Project20200710_b-2_z0_ch01.tif]

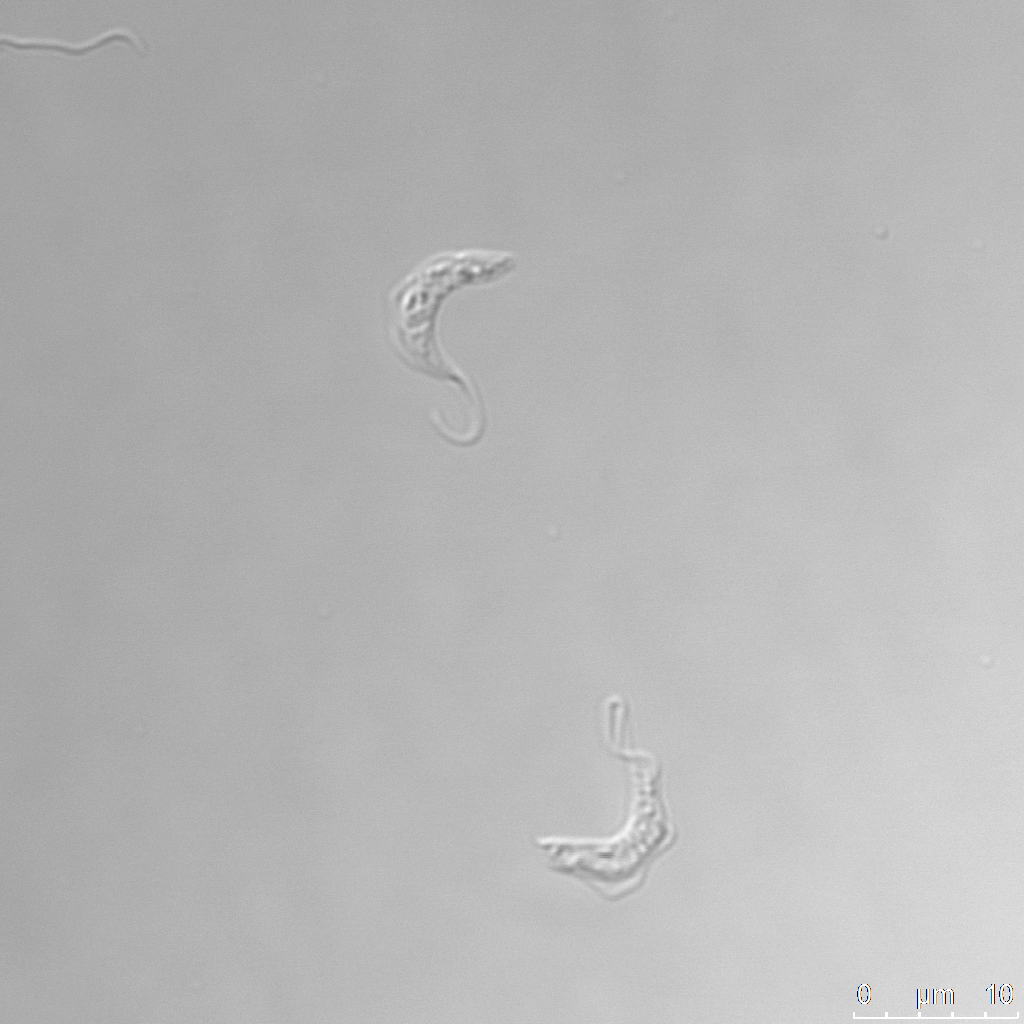

Supplement: Supplementary file 5 [file Data_Sheet_5.ZIP › Original Data 3-IFA/03 Oxamate/Negative/Project20200710_b-2_z0_ch02.tif]

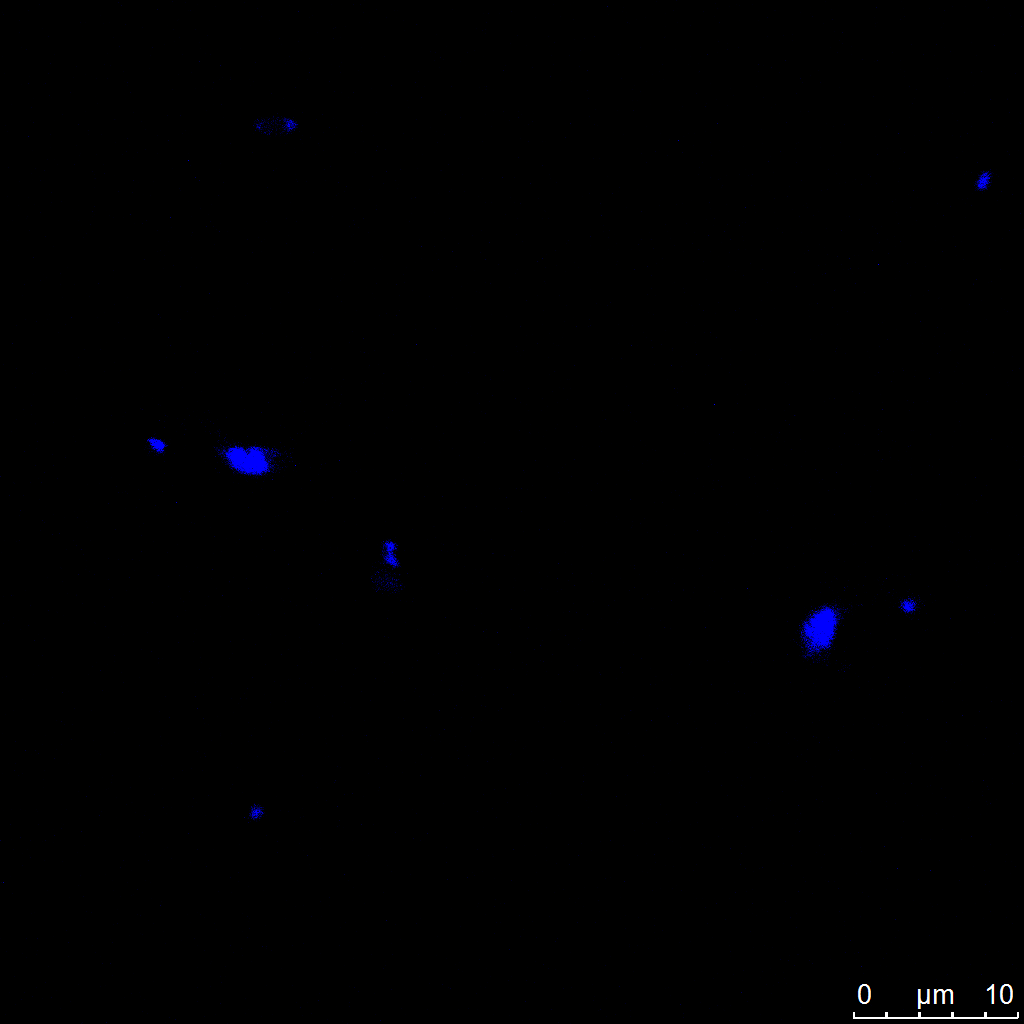

Supplement: Supplementary file 5 [file Data_Sheet_5.ZIP › Original Data 3-IFA/03 Oxamate/20 mM/Project20200710_0711-S20-6_z0_ch00.tif]

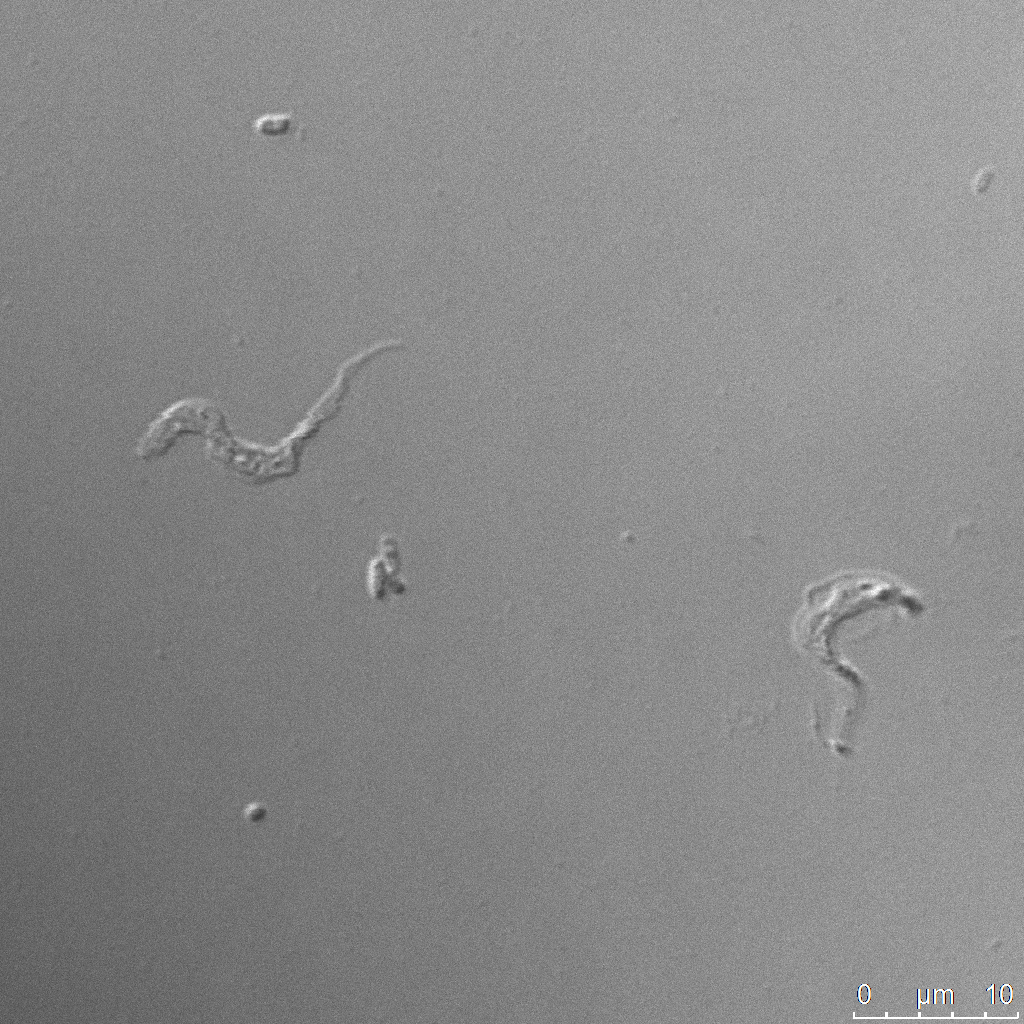

Supplement: Supplementary file 5 [file Data_Sheet_5.ZIP › Original Data 3-IFA/03 Oxamate/20 mM/Project20200710_0711-S20-6_z0_ch01.tif]

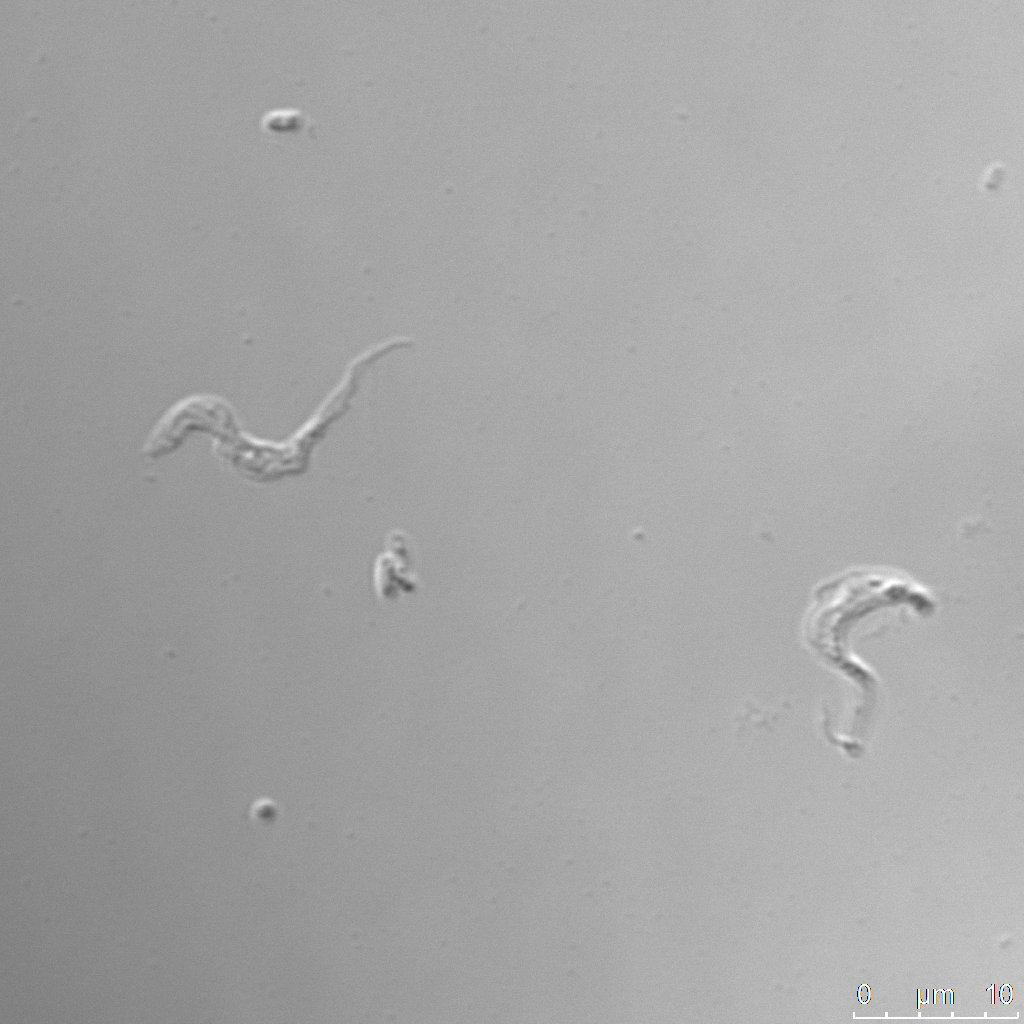

Supplement: Supplementary file 5 [file Data_Sheet_5.ZIP › Original Data 3-IFA/03 Oxamate/20 mM/Project20200710_0711-S20-6_z0_ch03.tif]

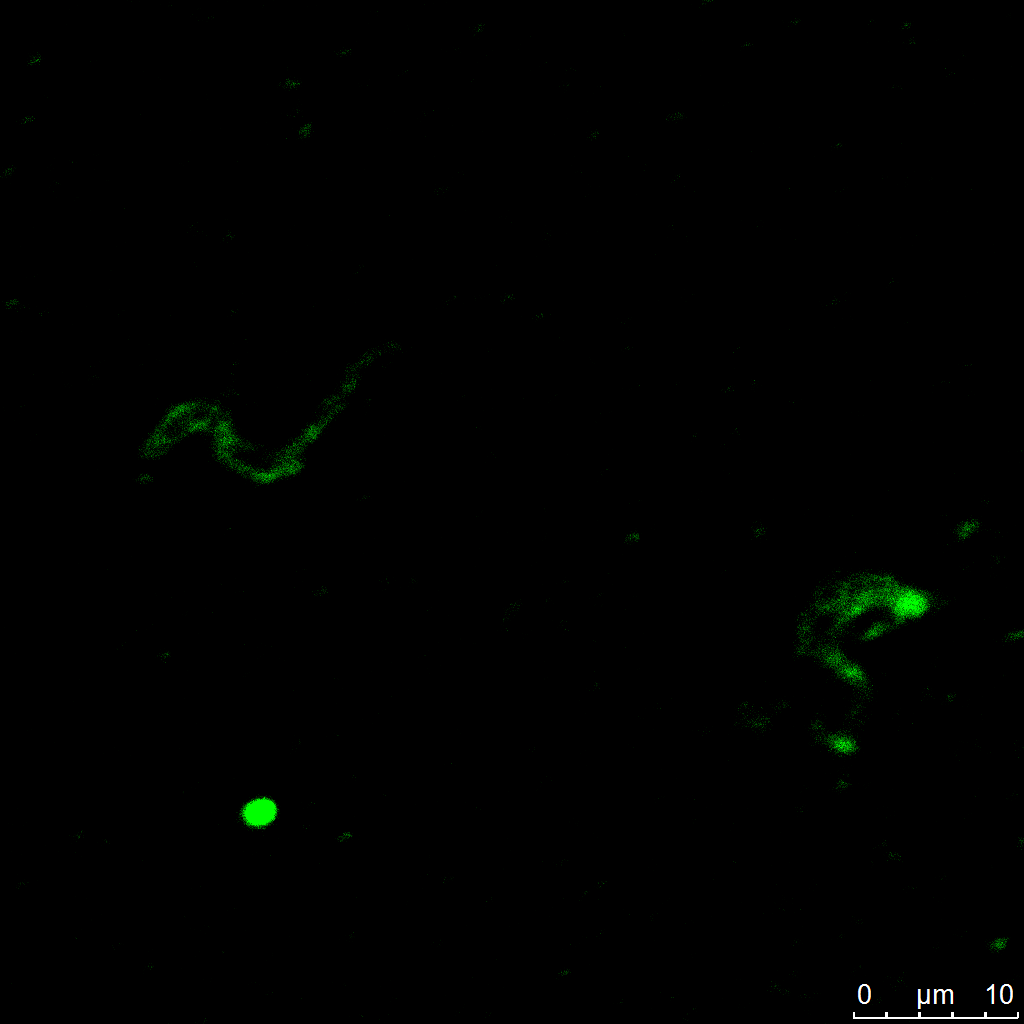

Supplement: Supplementary file 5 [file Data_Sheet_5.ZIP › Original Data 3-IFA/03 Oxamate/20 mM/Project20200710_0711-S20-6_z0_ch02.tif]

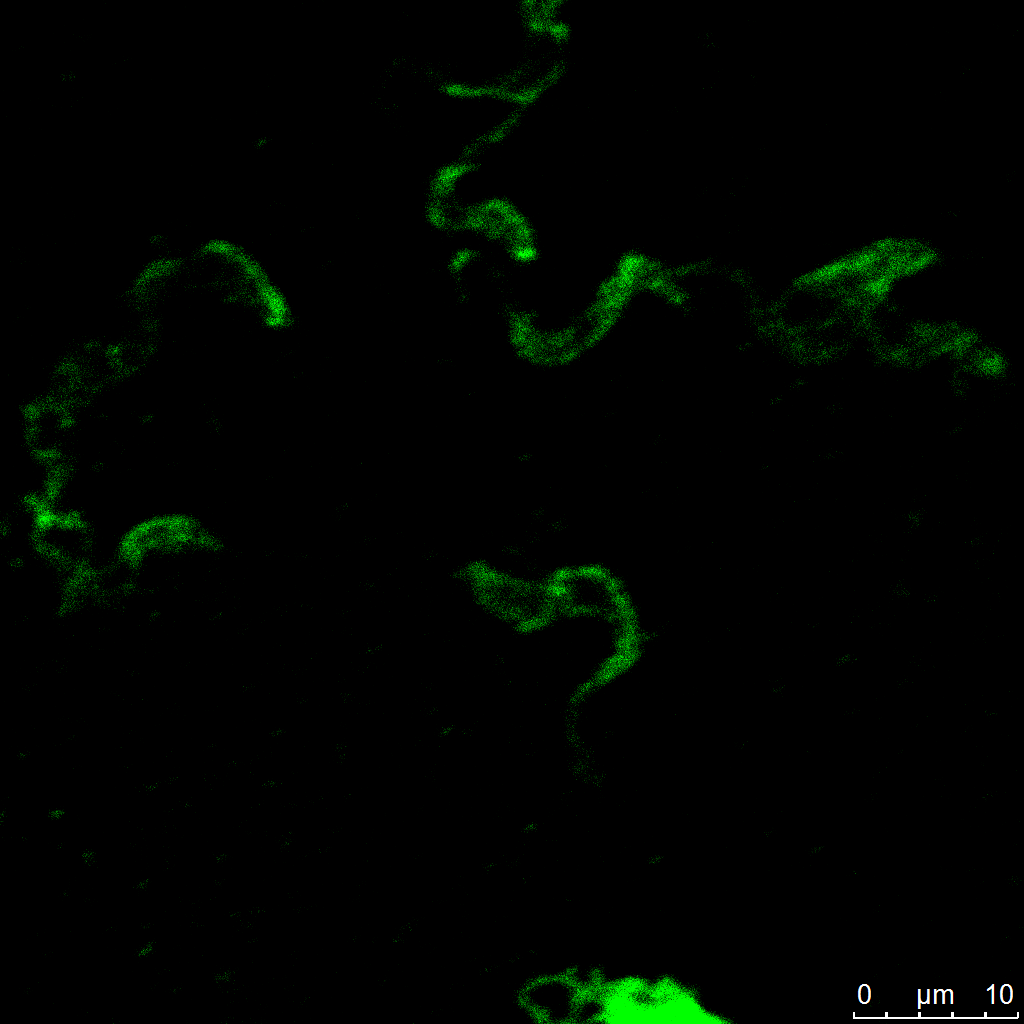

Supplement: Supplementary file 5 [file Data_Sheet_5.ZIP › Original Data 3-IFA/03 Oxamate/10 mM/Project20200710_0711-S10-3_z0_ch02.tif]

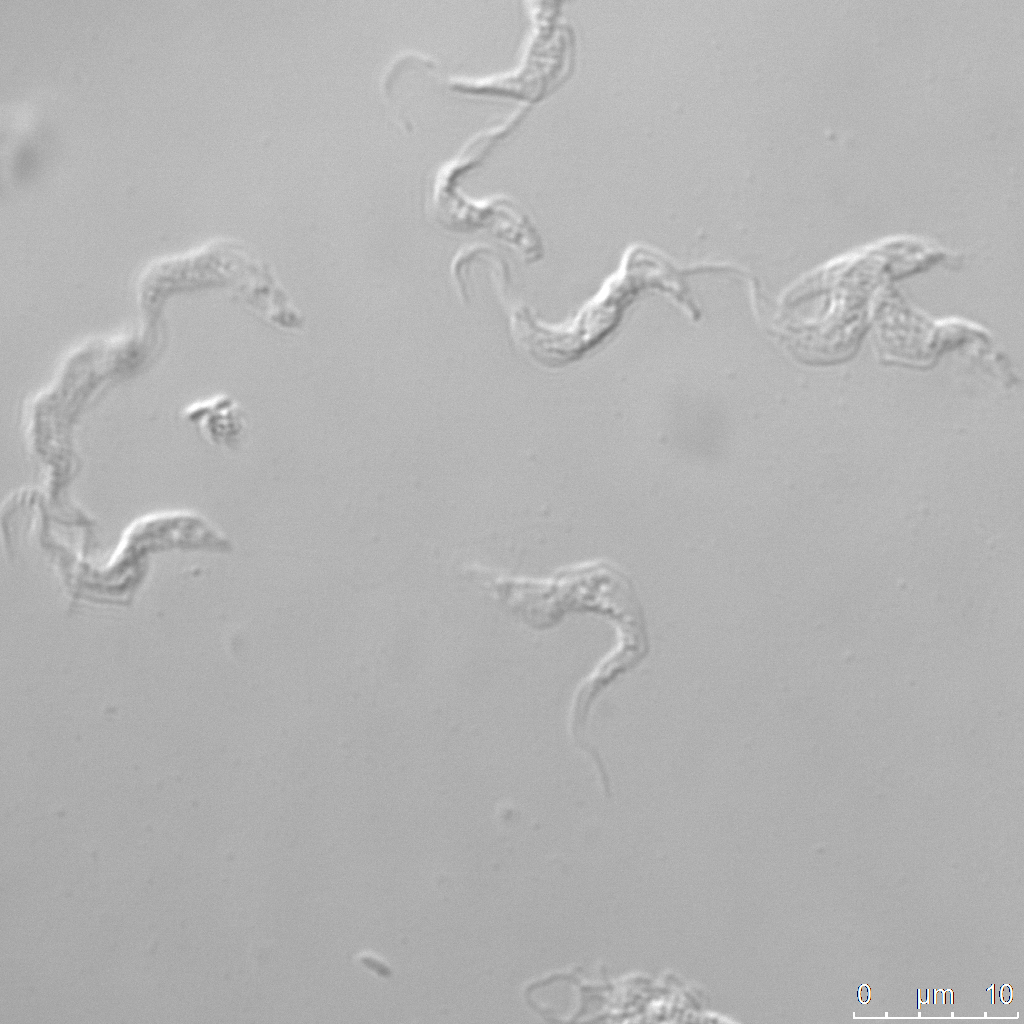

Supplement: Supplementary file 5 [file Data_Sheet_5.ZIP › Original Data 3-IFA/03 Oxamate/10 mM/Project20200710_0711-S10-3_z0_ch03.tif]

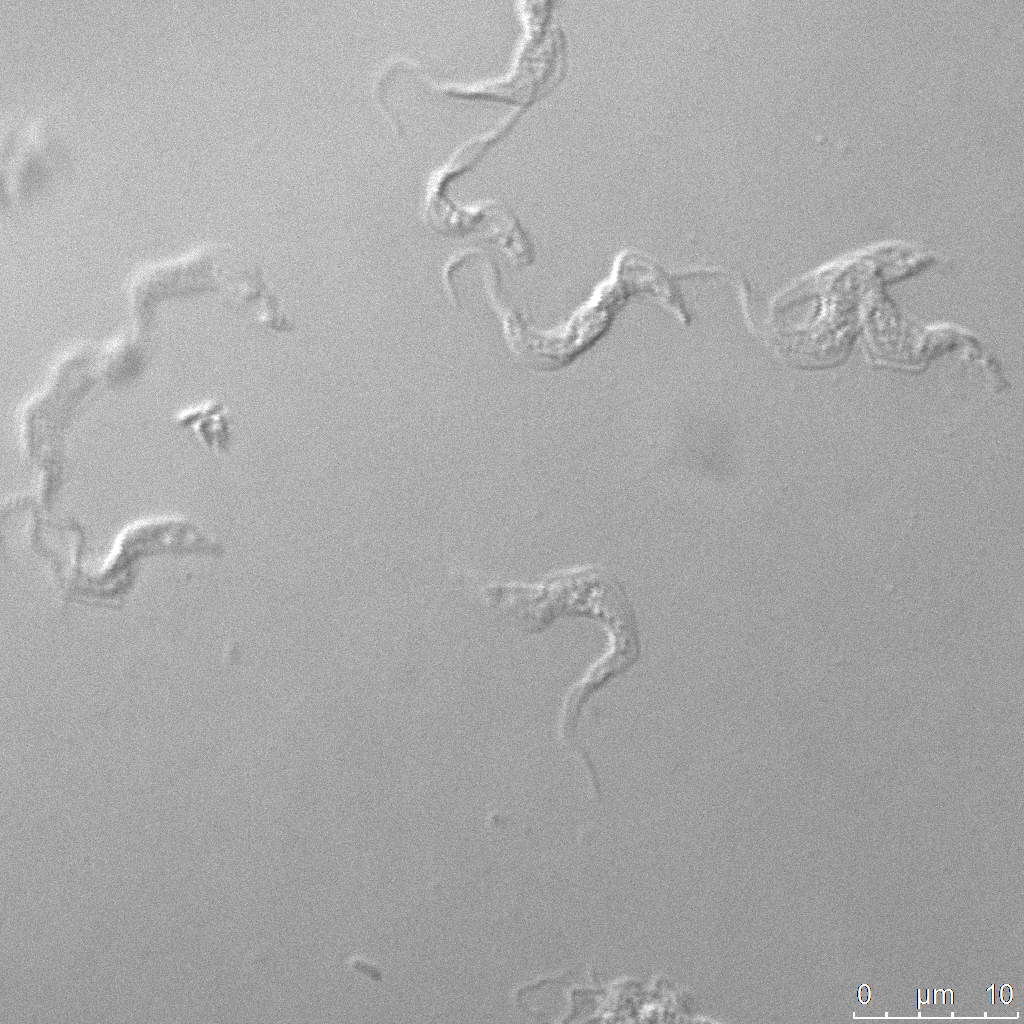

Supplement: Supplementary file 5 [file Data_Sheet_5.ZIP › Original Data 3-IFA/03 Oxamate/10 mM/Project20200710_0711-S10-3_z0_ch01.tif]

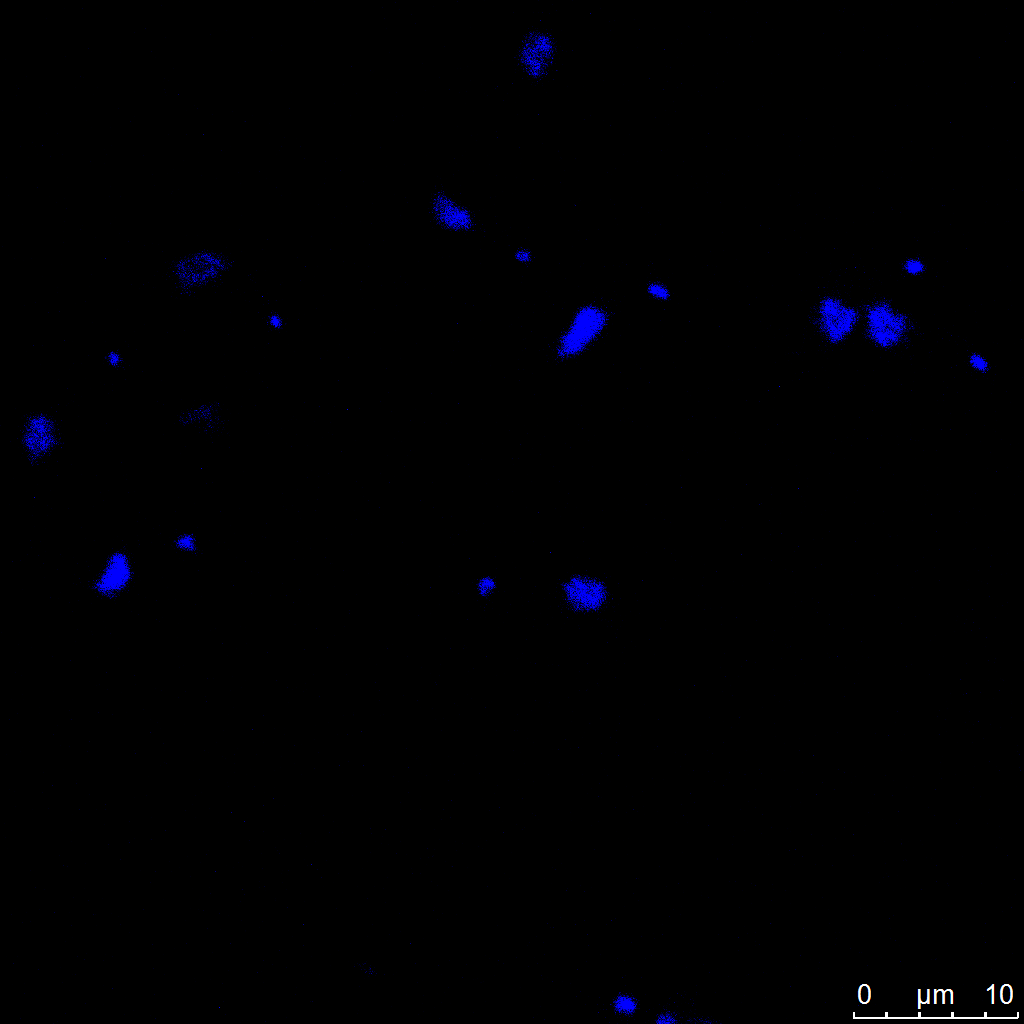

Supplement: Supplementary file 5 [file Data_Sheet_5.ZIP › Original Data 3-IFA/03 Oxamate/10 mM/Project20200710_0711-S10-3_z0_ch00.tif]

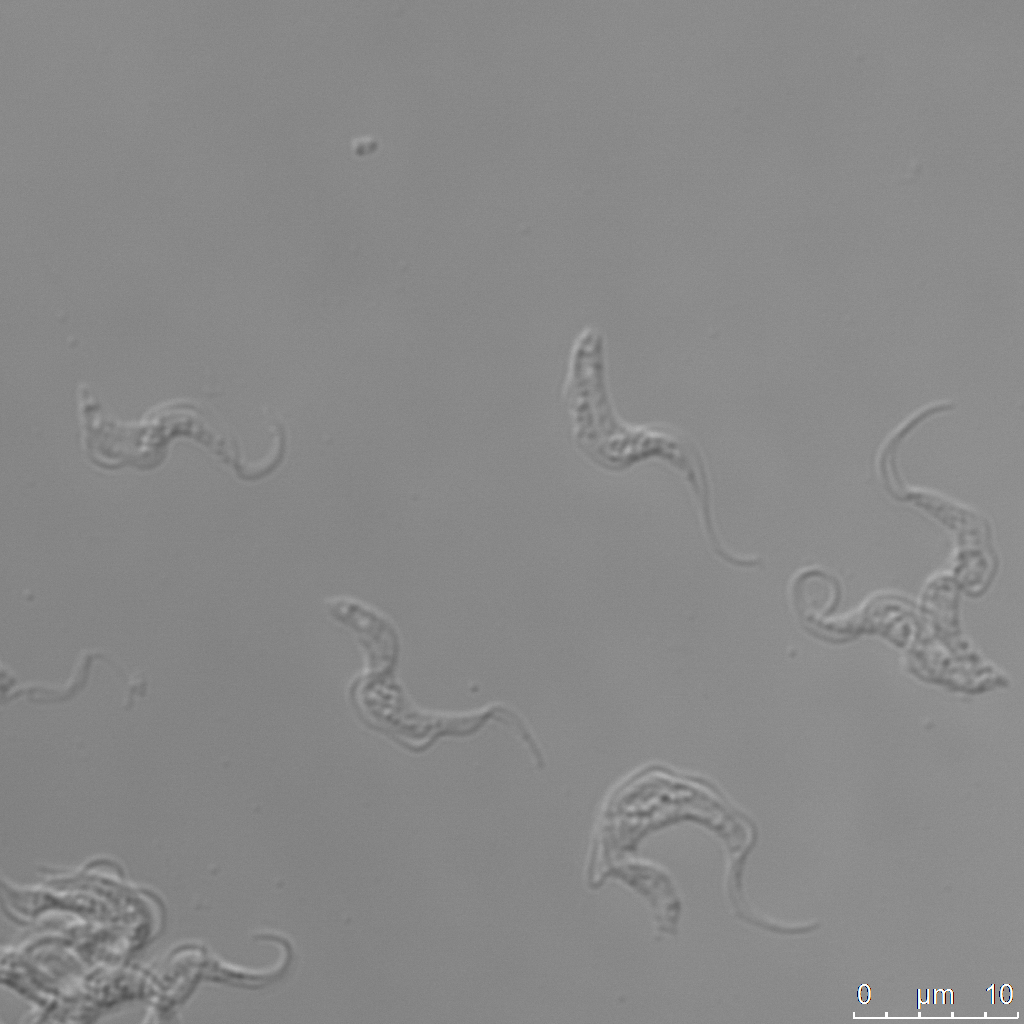

Supplement: Supplementary file 5 [file Data_Sheet_5.ZIP › Original Data 3-IFA/02 2-DG/1 mM/Project20200710_0715-D1-3_z0_ch03.tif]

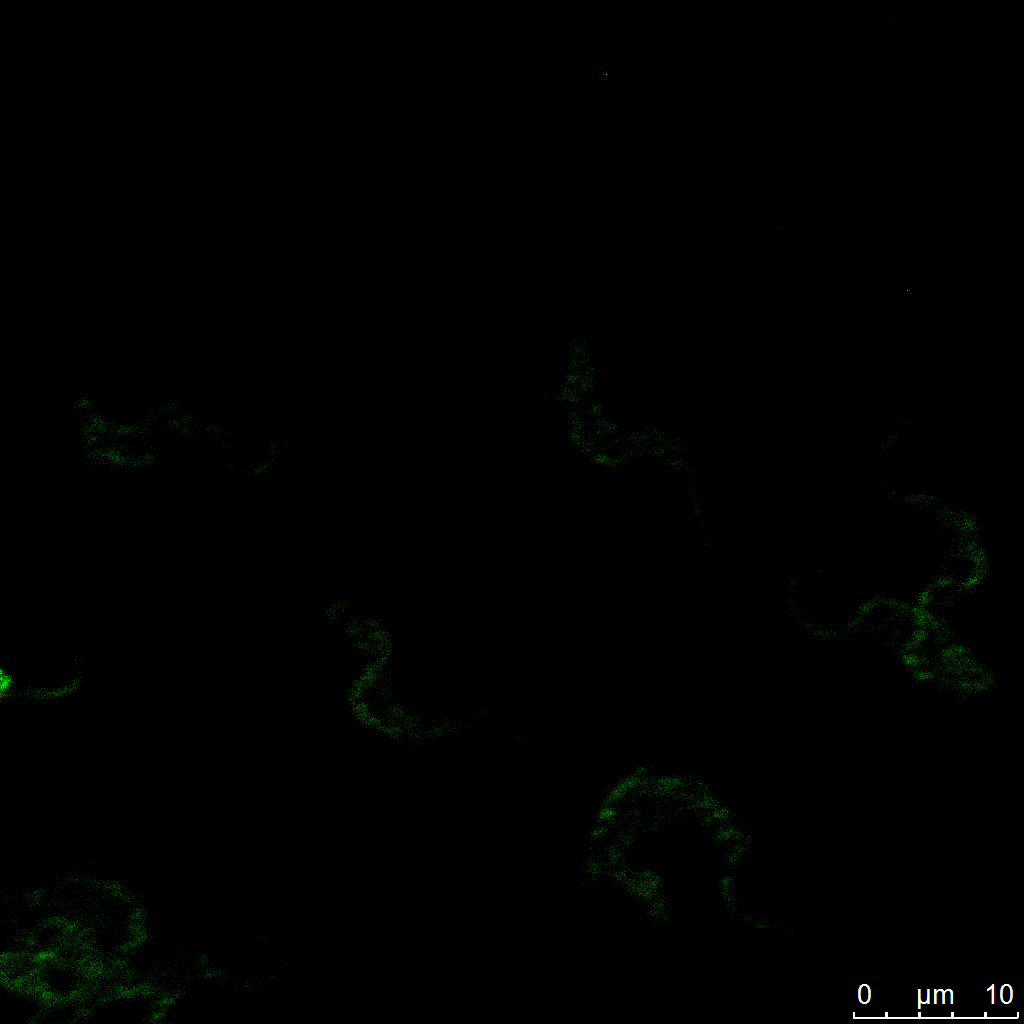

Supplement: Supplementary file 5 [file Data_Sheet_5.ZIP › Original Data 3-IFA/02 2-DG/1 mM/Project20200710_0715-D1-3_z0_ch02.tif]

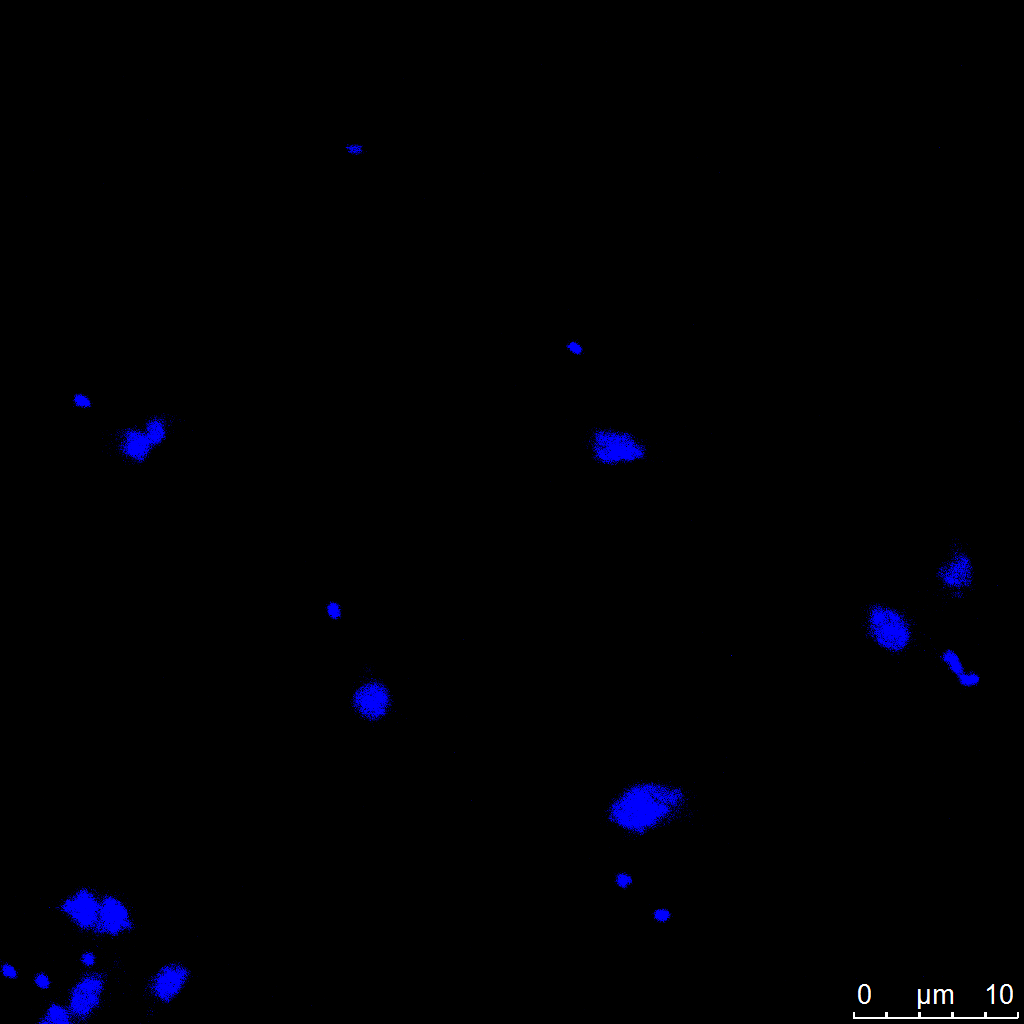

Supplement: Supplementary file 5 [file Data_Sheet_5.ZIP › Original Data 3-IFA/02 2-DG/1 mM/Project20200710_0715-D1-3_z0_ch00.tif]

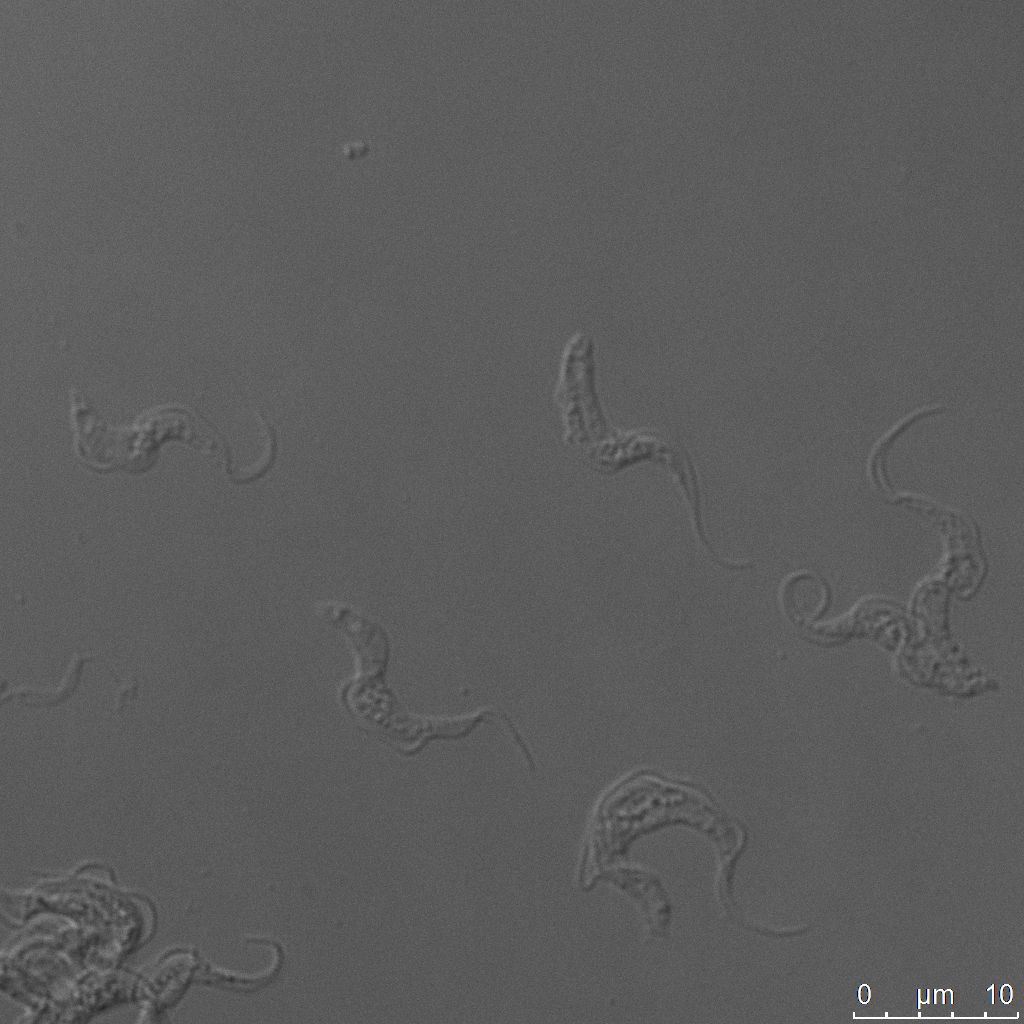

Supplement: Supplementary file 5 [file Data_Sheet_5.ZIP › Original Data 3-IFA/02 2-DG/1 mM/Project20200710_0715-D1-3_z0_ch01.tif]

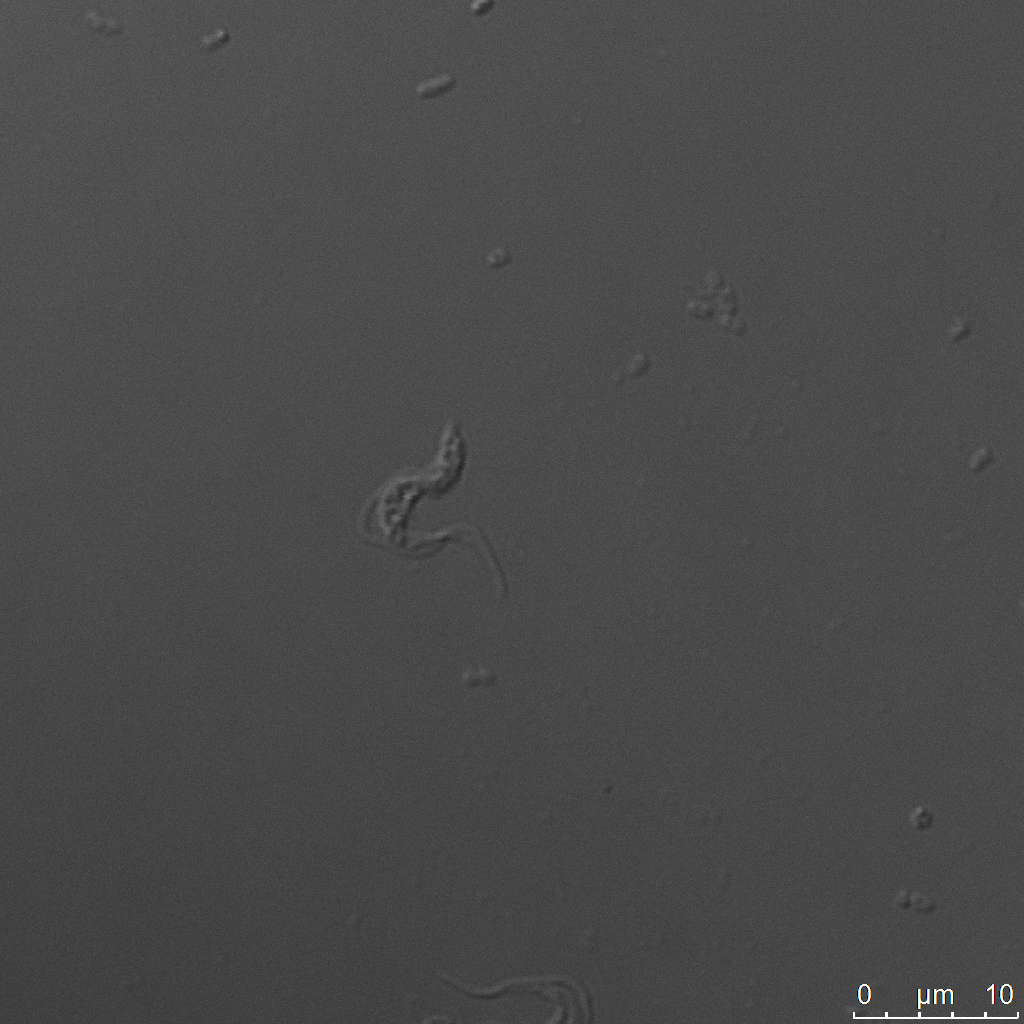

Supplement: Supplementary file 5 [file Data_Sheet_5.ZIP › Original Data 3-IFA/02 2-DG/5 mM/Project20200710_0715-D5-8_z0_ch01.tif]

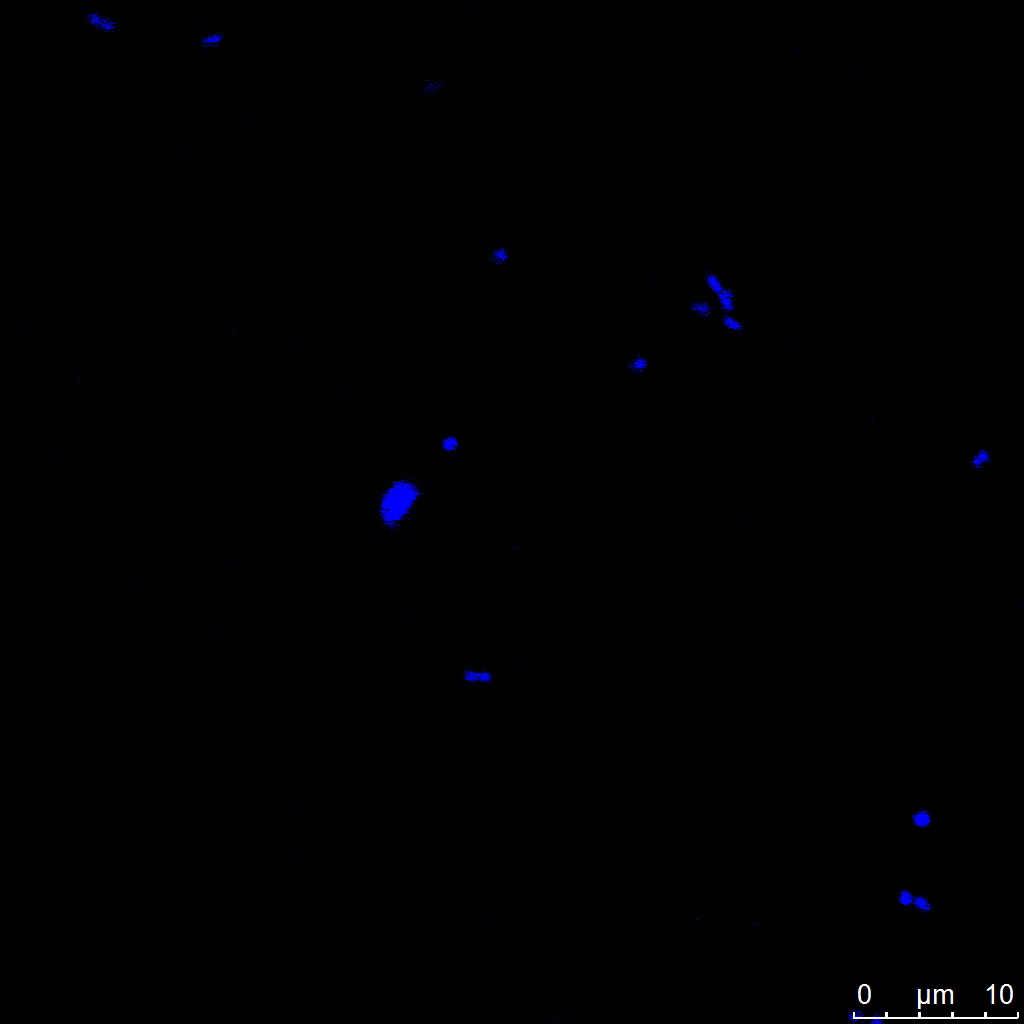

Supplement: Supplementary file 5 [file Data_Sheet_5.ZIP › Original Data 3-IFA/02 2-DG/5 mM/Project20200710_0715-D5-8_z0_ch00.tif]

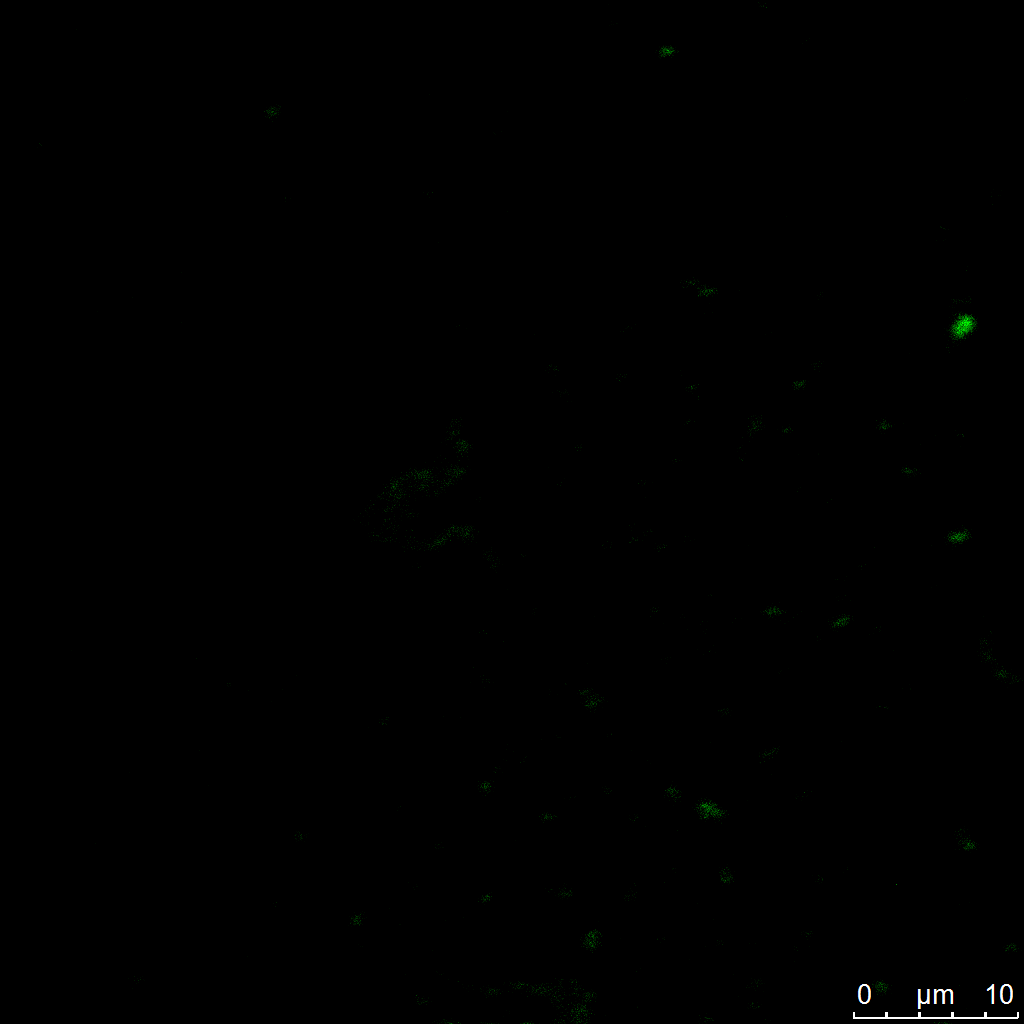

Supplement: Supplementary file 5 [file Data_Sheet_5.ZIP › Original Data 3-IFA/02 2-DG/5 mM/Project20200710_0715-D5-8_z0_ch02.tif]

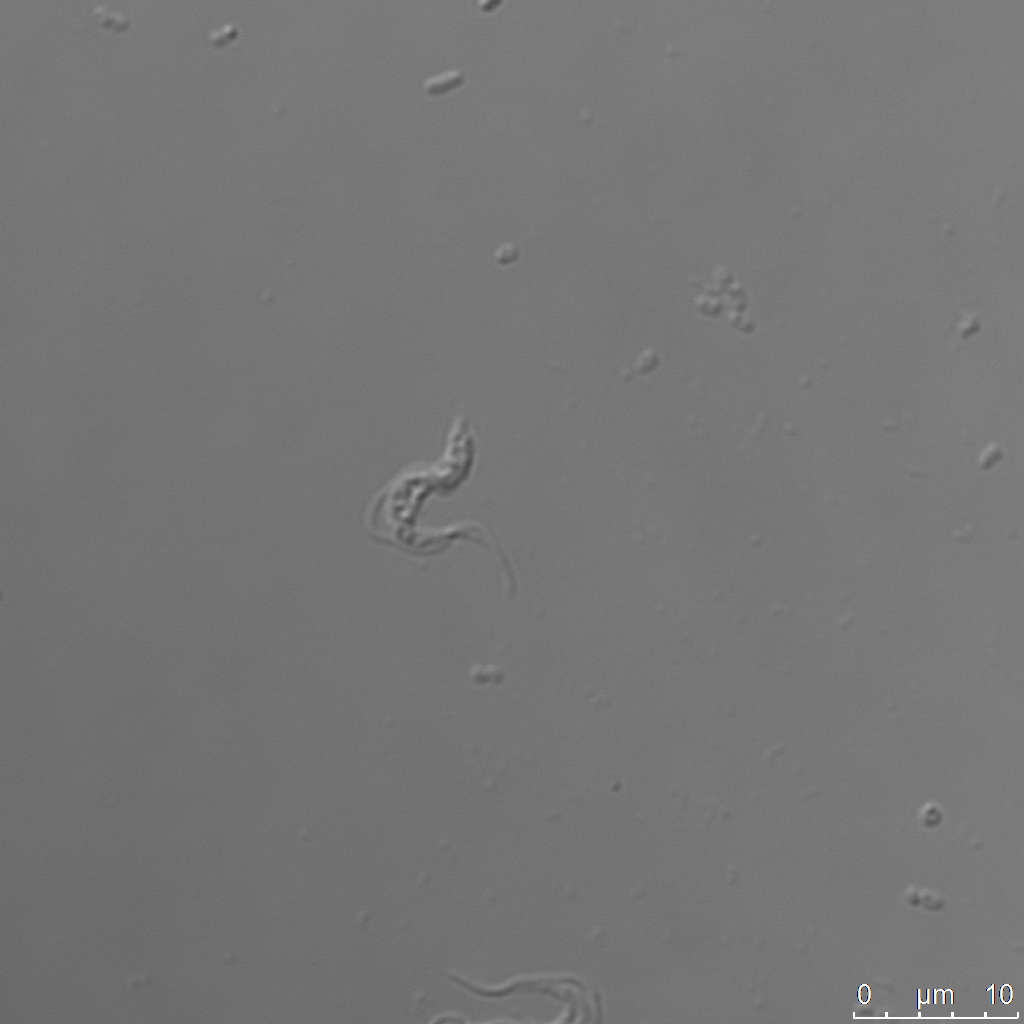

Supplement: Supplementary file 5 [file Data_Sheet_5.ZIP › Original Data 3-IFA/02 2-DG/5 mM/Project20200710_0715-D5-8_z0_ch03.tif]

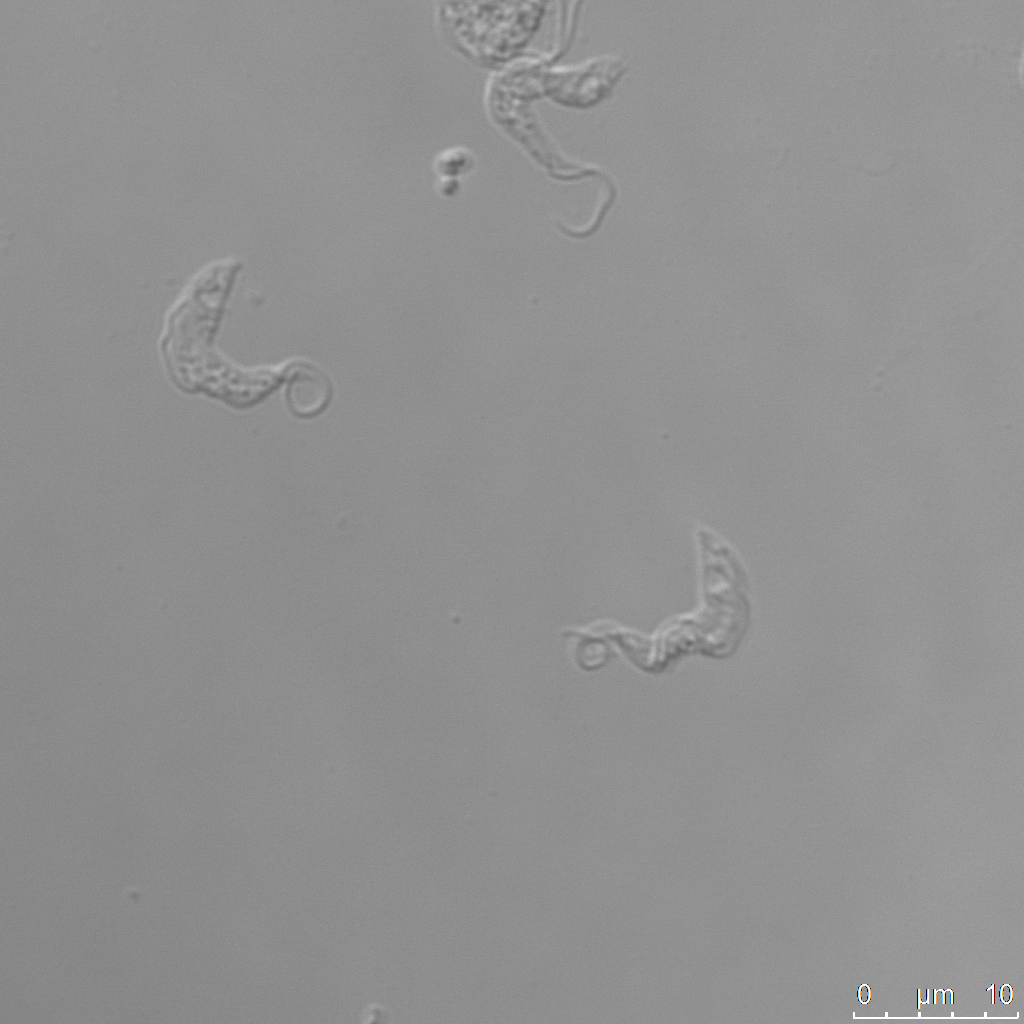

Supplement: Supplementary file 5 [file Data_Sheet_5.ZIP › Original Data 3-IFA/02 2-DG/0 mM/Project20200710_0715-D0-5_z0_ch03.tif]

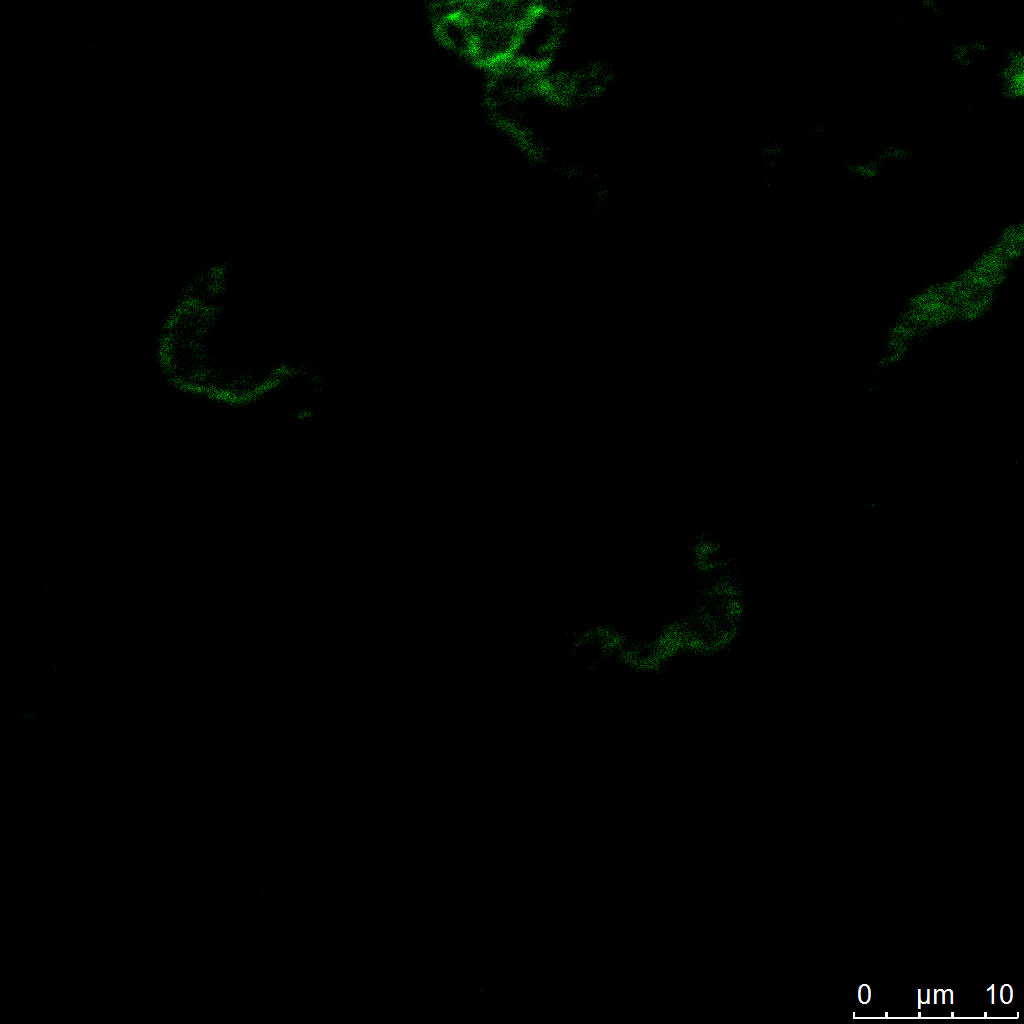

Supplement: Supplementary file 5 [file Data_Sheet_5.ZIP › Original Data 3-IFA/02 2-DG/0 mM/Project20200710_0715-D0-5_z0_ch02.tif]

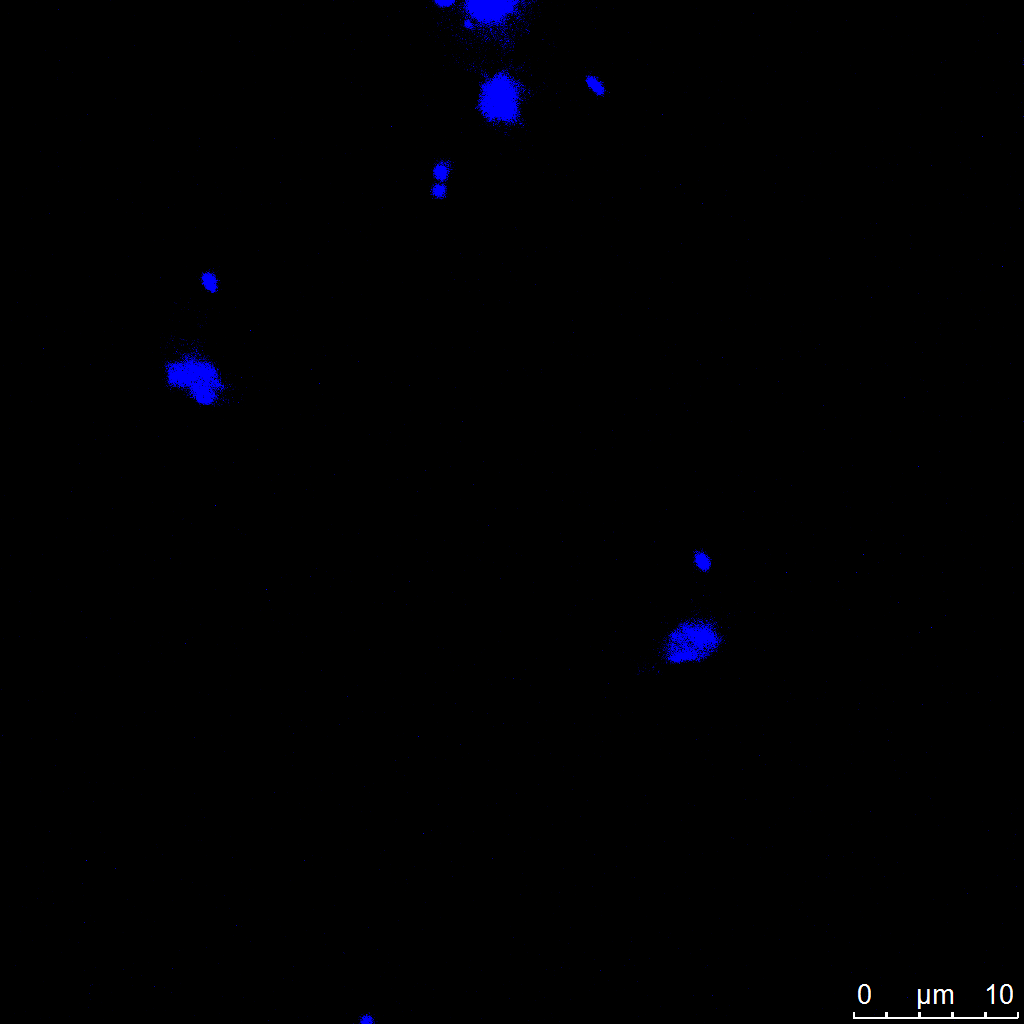

Supplement: Supplementary file 5 [file Data_Sheet_5.ZIP › Original Data 3-IFA/02 2-DG/0 mM/Project20200710_0715-D0-5_z0_ch00.tif]

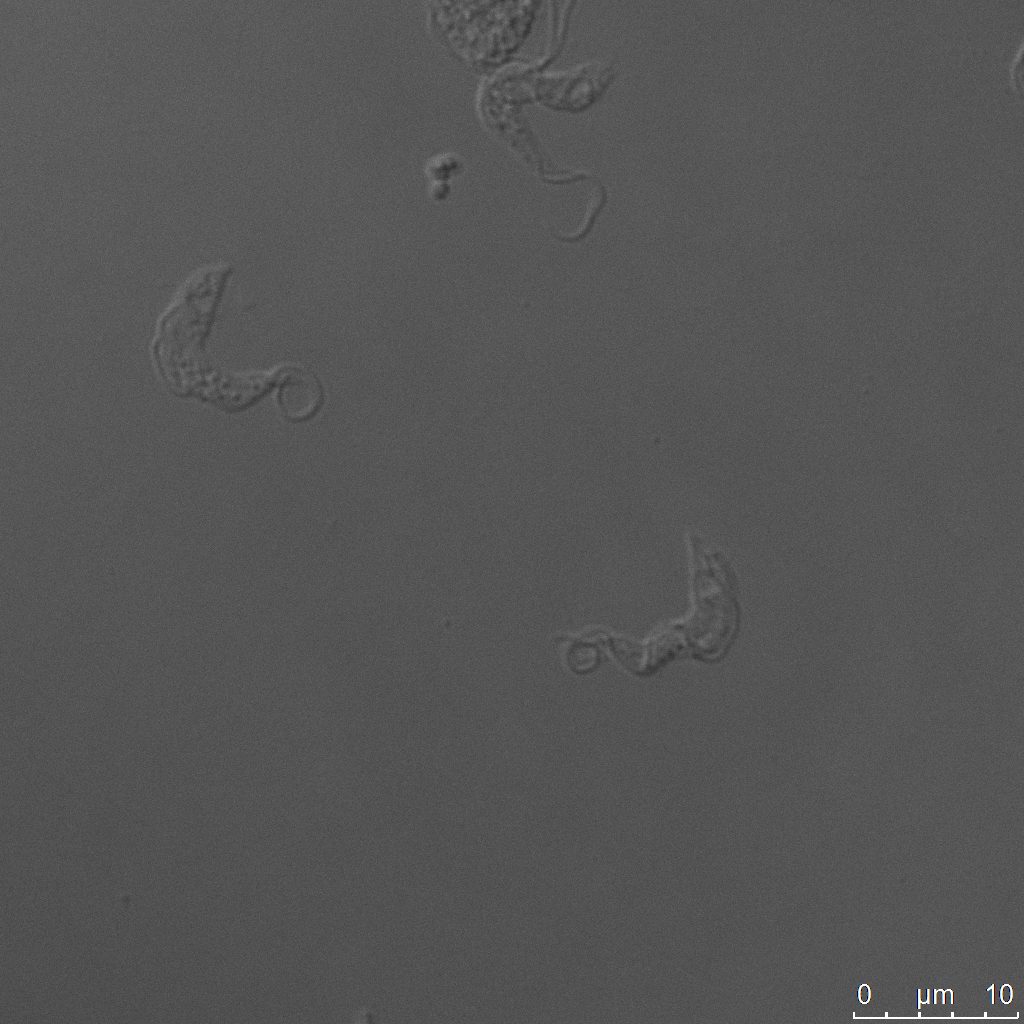

Supplement: Supplementary file 5 [file Data_Sheet_5.ZIP › Original Data 3-IFA/02 2-DG/0 mM/Project20200710_0715-D0-5_z0_ch01.tif]

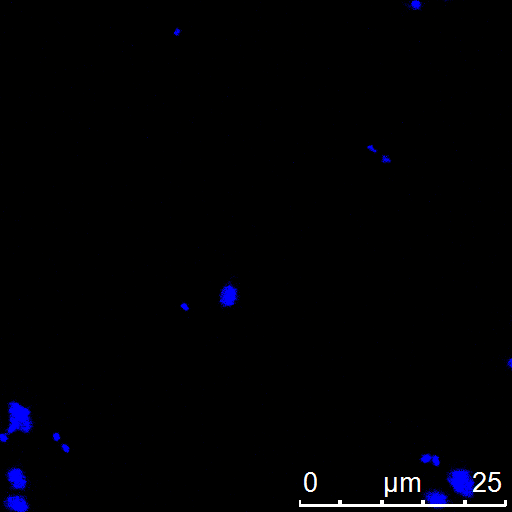

Supplement: Supplementary file 5 [file Data_Sheet_5.ZIP › Original Data 3-IFA/02 2-DG/Negative/Project20200710_0715-N-2_z0_ch00.tif]

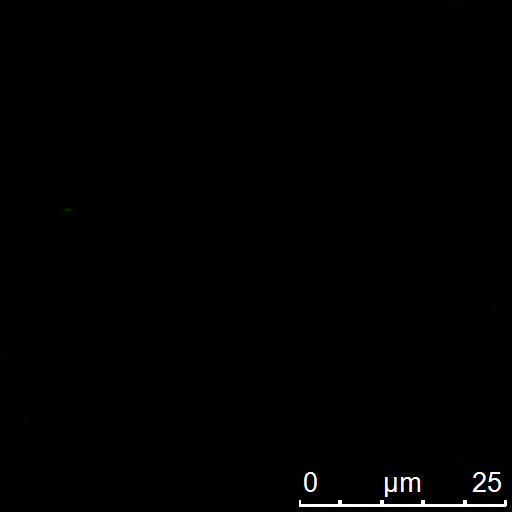

Supplement: Supplementary file 5 [file Data_Sheet_5.ZIP › Original Data 3-IFA/02 2-DG/Negative/Project20200710_0715-N-2_z0_ch02.tif]

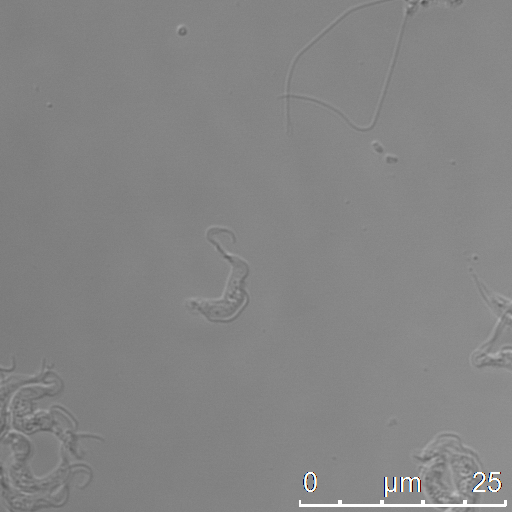

Supplement: Supplementary file 5 [file Data_Sheet_5.ZIP › Original Data 3-IFA/02 2-DG/Negative/Project20200710_0715-N-2_z0_ch03.tif]

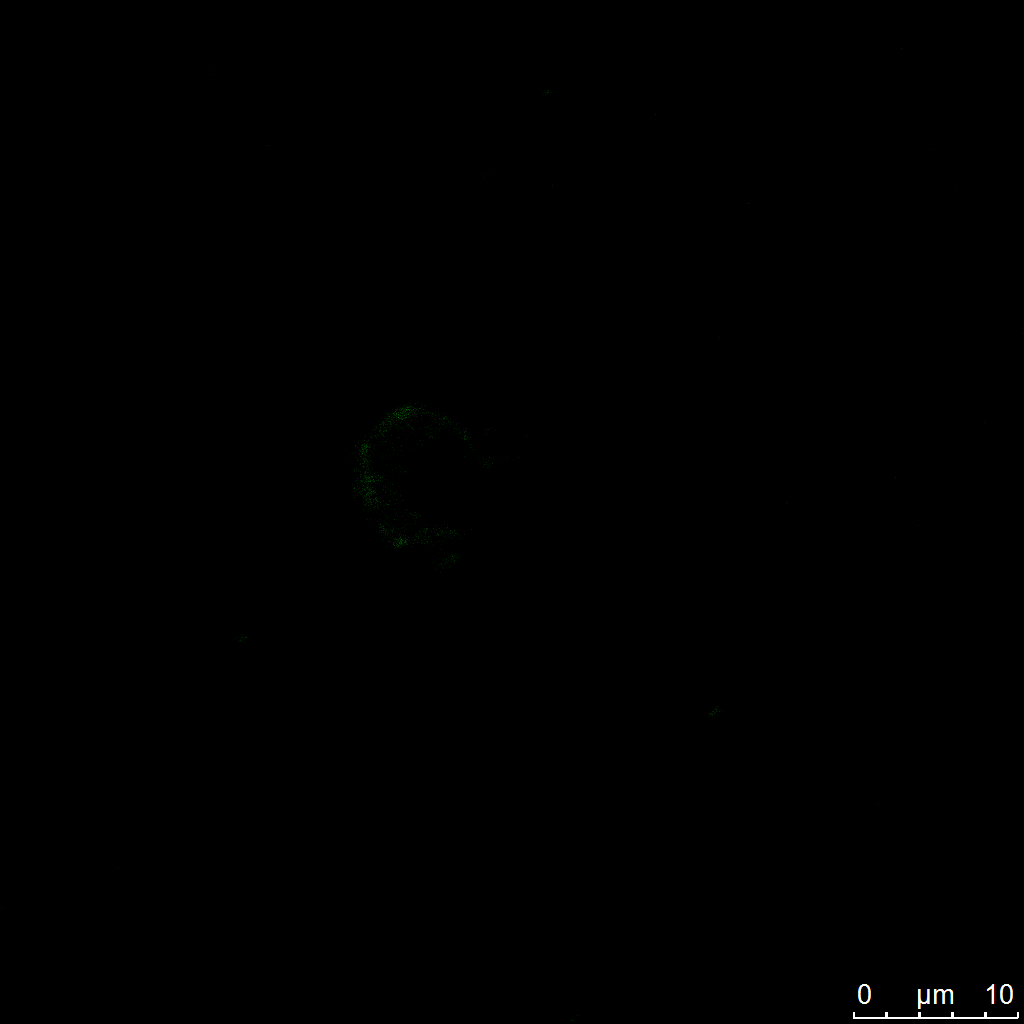

Supplement: Supplementary file 5 [file Data_Sheet_5.ZIP › Original Data 3-IFA/02 2-DG/10 mM/Project20200710_0715-D10-6_z0_ch02.tif]

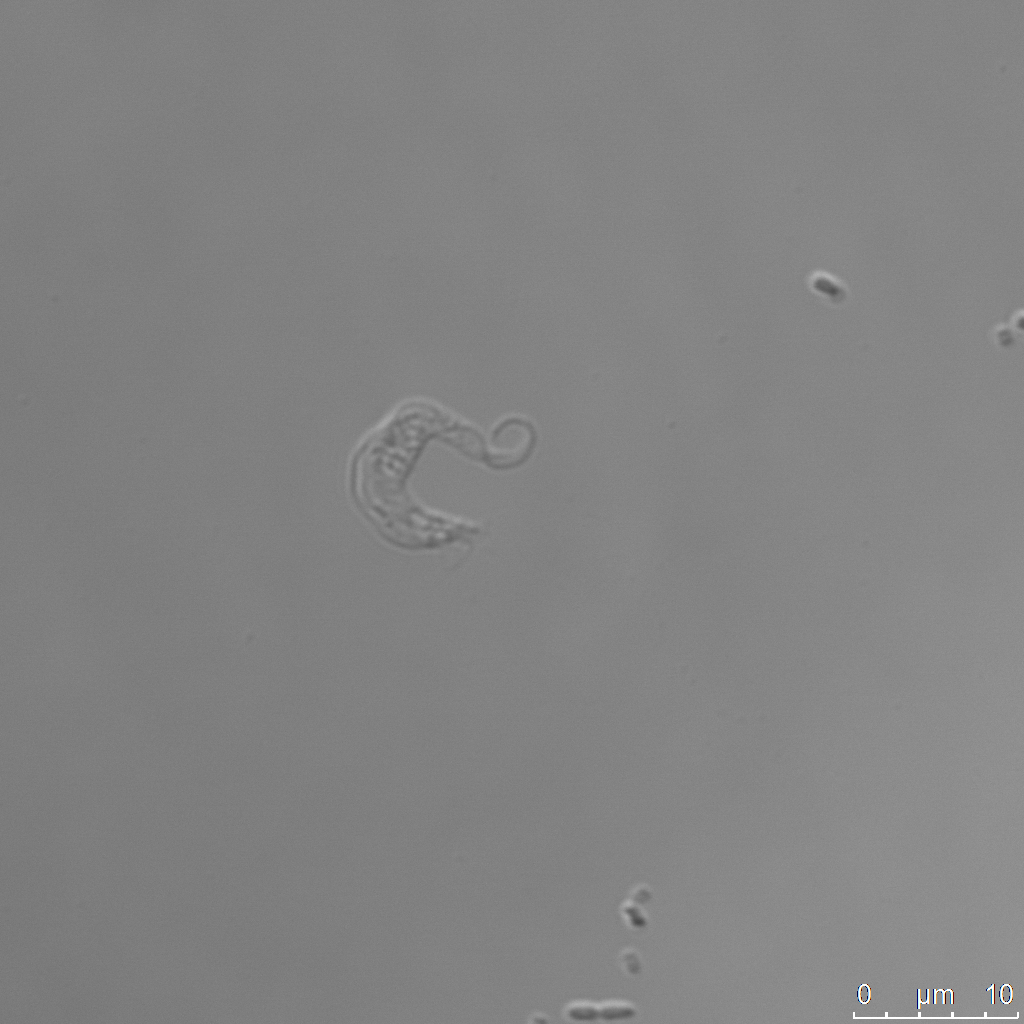

Supplement: Supplementary file 5 [file Data_Sheet_5.ZIP › Original Data 3-IFA/02 2-DG/10 mM/Project20200710_0715-D10-6_z0_ch03.tif]

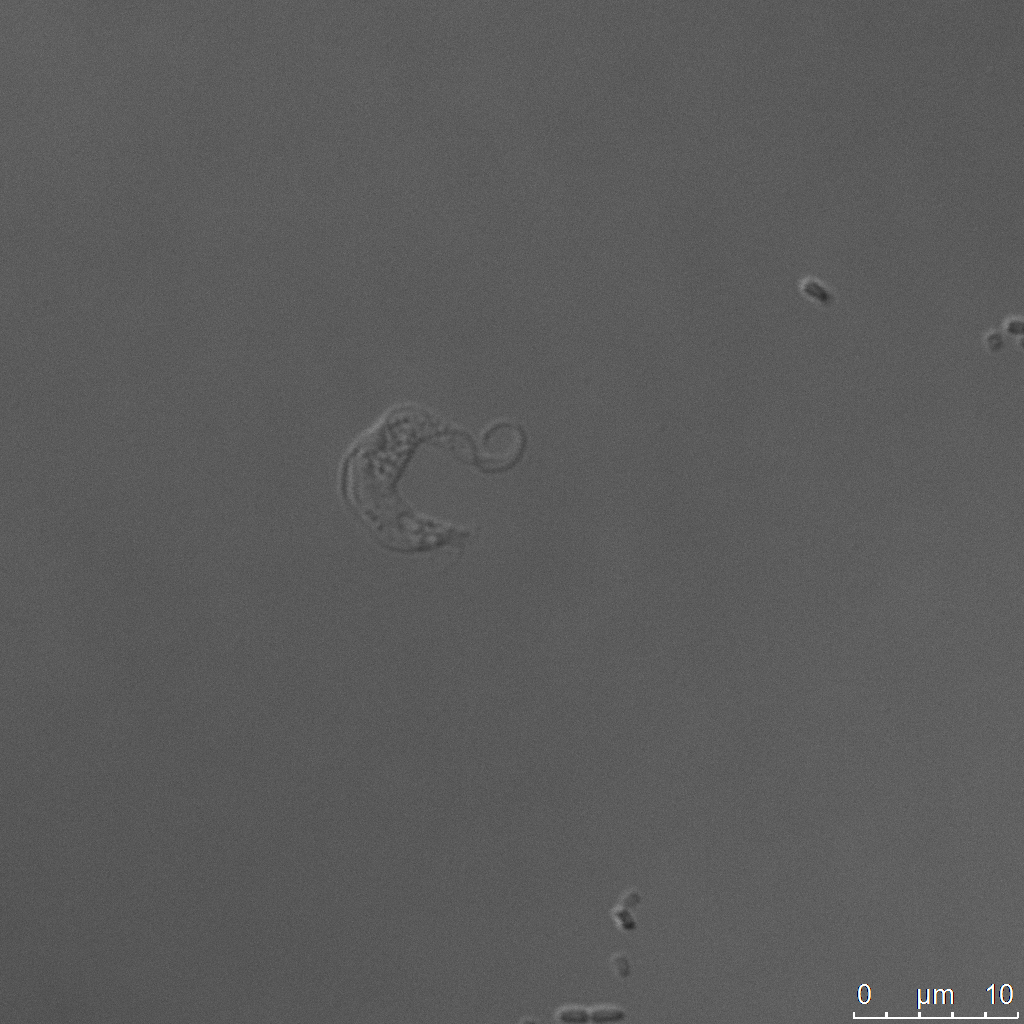

Supplement: Supplementary file 5 [file Data_Sheet_5.ZIP › Original Data 3-IFA/02 2-DG/10 mM/Project20200710_0715-D10-6_z0_ch01.tif]

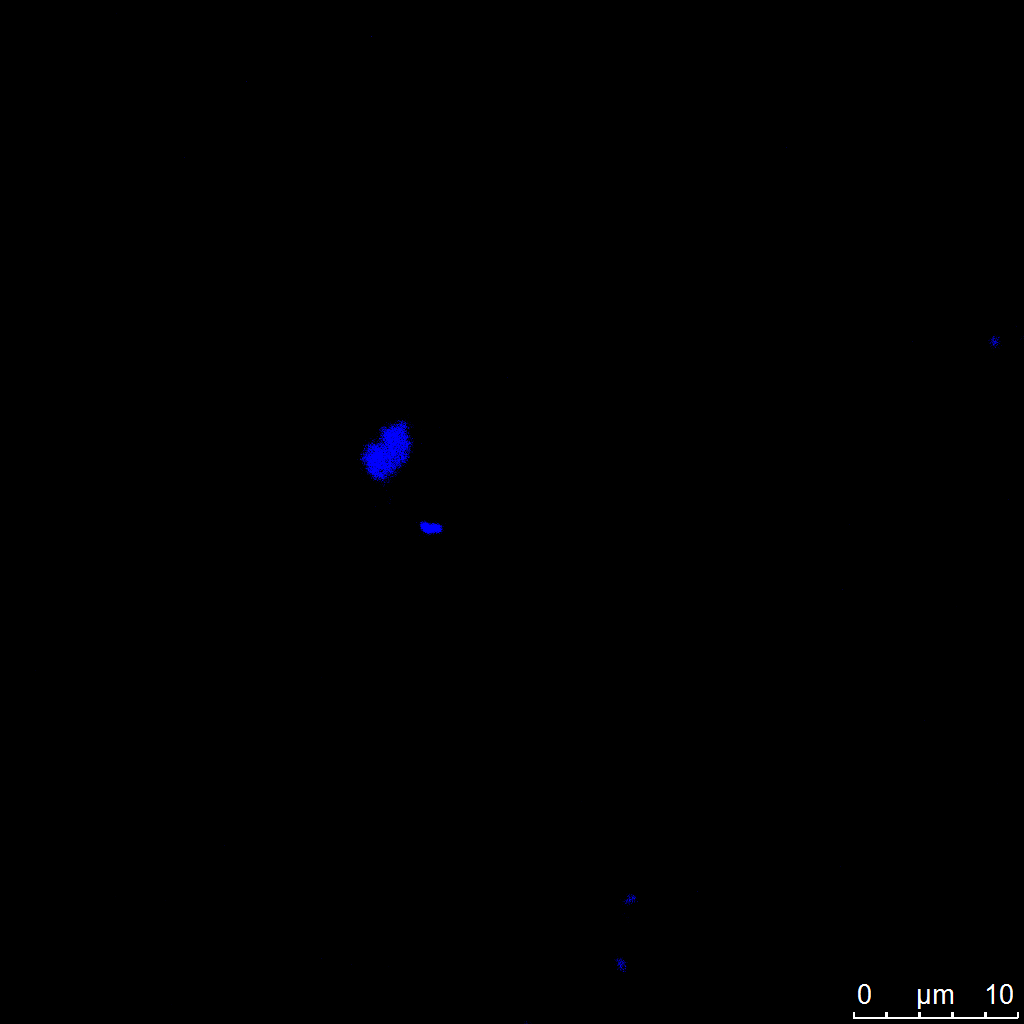

Supplement: Supplementary file 5 [file Data_Sheet_5.ZIP › Original Data 3-IFA/02 2-DG/10 mM/Project20200710_0715-D10-6_z0_ch00.tif]

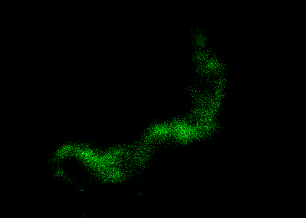

Supplement: Supplementary file 5 [file Data_Sheet_5.ZIP › Original Data 3-IFA/Fluorescence intensity statistics/Oxamate 10 mM/10.tif]

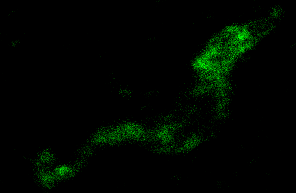

Supplement: Supplementary file 5 [file Data_Sheet_5.ZIP › Original Data 3-IFA/Fluorescence intensity statistics/Oxamate 10 mM/11.tif]

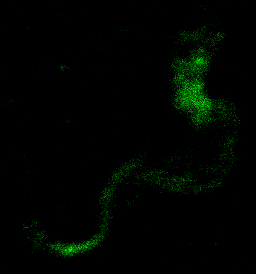

Supplement: Supplementary file 5 [file Data_Sheet_5.ZIP › Original Data 3-IFA/Fluorescence intensity statistics/Oxamate 10 mM/9.tif]

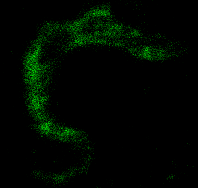

Supplement: Supplementary file 5 [file Data_Sheet_5.ZIP › Original Data 3-IFA/Fluorescence intensity statistics/Oxamate 10 mM/8.tif]

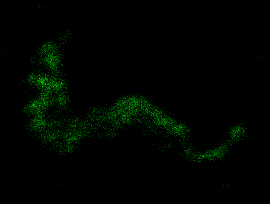

Supplement: Supplementary file 5 [file Data_Sheet_5.ZIP › Original Data 3-IFA/Fluorescence intensity statistics/Oxamate 10 mM/3.tif]

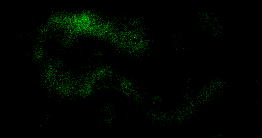

Supplement: Supplementary file 5 [file Data_Sheet_5.ZIP › Original Data 3-IFA/Fluorescence intensity statistics/Oxamate 10 mM/2.tif]

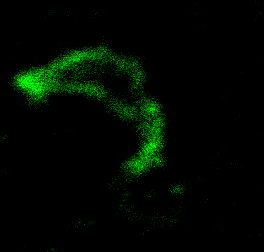

Supplement: Supplementary file 5 [file Data_Sheet_5.ZIP › Original Data 3-IFA/Fluorescence intensity statistics/Oxamate 10 mM/1.tif]

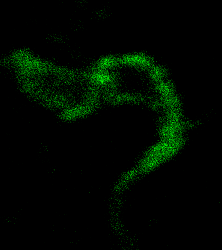

Supplement: Supplementary file 5 [file Data_Sheet_5.ZIP › Original Data 3-IFA/Fluorescence intensity statistics/Oxamate 10 mM/5.tif]

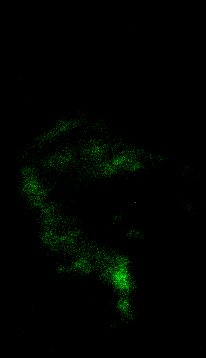

Supplement: Supplementary file 5 [file Data_Sheet_5.ZIP › Original Data 3-IFA/Fluorescence intensity statistics/Oxamate 10 mM/4.tif]

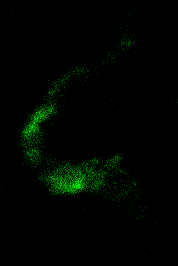

Supplement: Supplementary file 5 [file Data_Sheet_5.ZIP › Original Data 3-IFA/Fluorescence intensity statistics/Oxamate 10 mM/6.tif]

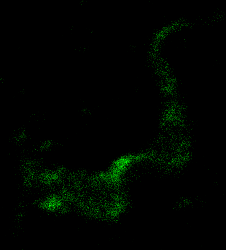

Supplement: Supplementary file 5 [file Data_Sheet_5.ZIP › Original Data 3-IFA/Fluorescence intensity statistics/Oxamate 10 mM/7.tif]

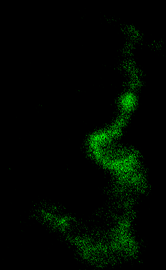

Supplement: Supplementary file 5 [file Data_Sheet_5.ZIP › Original Data 3-IFA/Fluorescence intensity statistics/Oxamate 20 mM/10.tif]

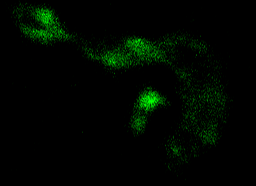

Supplement: Supplementary file 5 [file Data_Sheet_5.ZIP › Original Data 3-IFA/Fluorescence intensity statistics/Oxamate 20 mM/9.tif]

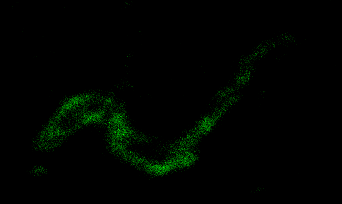

Supplement: Supplementary file 5 [file Data_Sheet_5.ZIP › Original Data 3-IFA/Fluorescence intensity statistics/Oxamate 20 mM/8.tif]

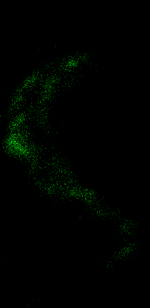

Supplement: Supplementary file 5 [file Data_Sheet_5.ZIP › Original Data 3-IFA/Fluorescence intensity statistics/Oxamate 20 mM/3.tif]

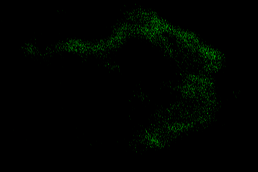

Supplement: Supplementary file 5 [file Data_Sheet_5.ZIP › Original Data 3-IFA/Fluorescence intensity statistics/Oxamate 20 mM/2.tif]

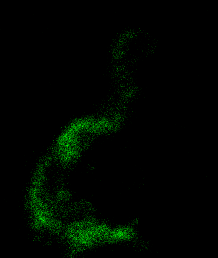

Supplement: Supplementary file 5 [file Data_Sheet_5.ZIP › Original Data 3-IFA/Fluorescence intensity statistics/Oxamate 20 mM/1.tif]

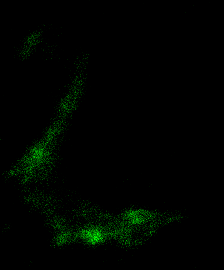

Supplement: Supplementary file 5 [file Data_Sheet_5.ZIP › Original Data 3-IFA/Fluorescence intensity statistics/Oxamate 20 mM/5.tif]

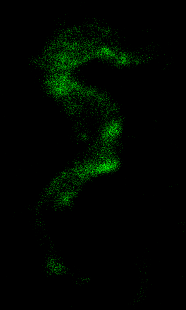

Supplement: Supplementary file 5 [file Data_Sheet_5.ZIP › Original Data 3-IFA/Fluorescence intensity statistics/Oxamate 20 mM/4.tif]

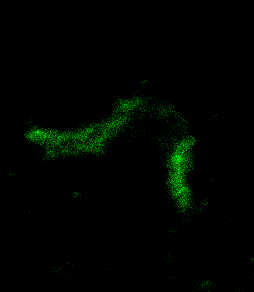

Supplement: Supplementary file 5 [file Data_Sheet_5.ZIP › Original Data 3-IFA/Fluorescence intensity statistics/Oxamate 20 mM/6.tif]

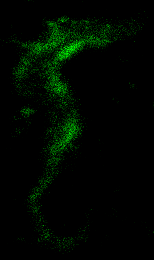

Supplement: Supplementary file 5 [file Data_Sheet_5.ZIP › Original Data 3-IFA/Fluorescence intensity statistics/Oxamate 20 mM/7.tif]

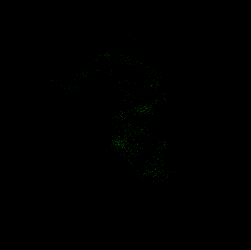

Supplement: Supplementary file 5 [file Data_Sheet_5.ZIP › Original Data 3-IFA/Fluorescence intensity statistics/2-DG 10 mM/10.tif]

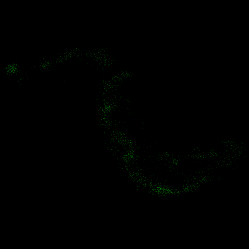

Supplement: Supplementary file 5 [file Data_Sheet_5.ZIP › Original Data 3-IFA/Fluorescence intensity statistics/2-DG 10 mM/9.tif]

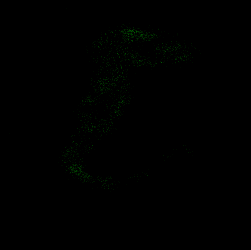

Supplement: Supplementary file 5 [file Data_Sheet_5.ZIP › Original Data 3-IFA/Fluorescence intensity statistics/2-DG 10 mM/8.tif]

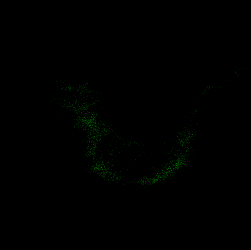

Supplement: Supplementary file 5 [file Data_Sheet_5.ZIP › Original Data 3-IFA/Fluorescence intensity statistics/2-DG 10 mM/3.tif]

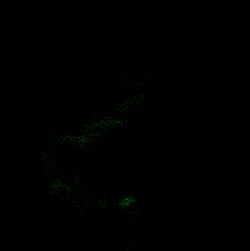

Supplement: Supplementary file 5 [file Data_Sheet_5.ZIP › Original Data 3-IFA/Fluorescence intensity statistics/2-DG 10 mM/2.tif]

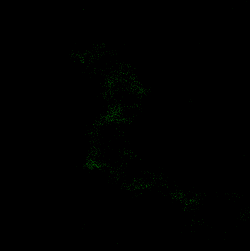

Supplement: Supplementary file 5 [file Data_Sheet_5.ZIP › Original Data 3-IFA/Fluorescence intensity statistics/2-DG 10 mM/1.tif]

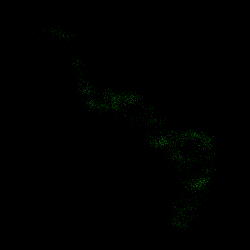

Supplement: Supplementary file 5 [file Data_Sheet_5.ZIP › Original Data 3-IFA/Fluorescence intensity statistics/2-DG 10 mM/5.tif]

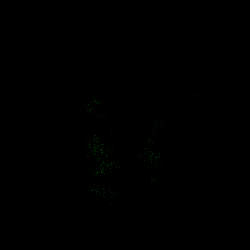

Supplement: Supplementary file 5 [file Data_Sheet_5.ZIP › Original Data 3-IFA/Fluorescence intensity statistics/2-DG 10 mM/4.tif]

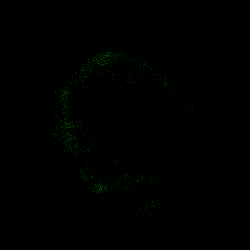

Supplement: Supplementary file 5 [file Data_Sheet_5.ZIP › Original Data 3-IFA/Fluorescence intensity statistics/2-DG 10 mM/6.tif]

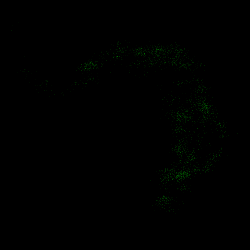

Supplement: Supplementary file 5 [file Data_Sheet_5.ZIP › Original Data 3-IFA/Fluorescence intensity statistics/2-DG 10 mM/7.tif]

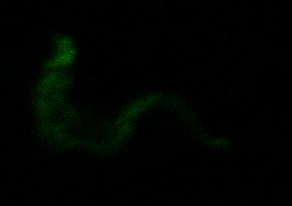

Supplement: Supplementary file 5 [file Data_Sheet_5.ZIP › Original Data 3-IFA/Fluorescence intensity statistics/Glucose 25 mM/3.tif]
