# Supplementary material for: Protein Lactylation Critically Regulates Energy Metabolism in the Protozoan Parasite Trypanosoma brucei
Source: Front Cell Dev Biol. 2021 Oct 14;9:719720. doi: 10.3389/fcell.2021.719720 (PMC8551762; doi:10.3389/fcell.2021.719720)
Supplement: Supplementary file 4 [file Data_Sheet_4.ZIP › Original Data 2-Flow Cytometry/Cell cycle.pdf]

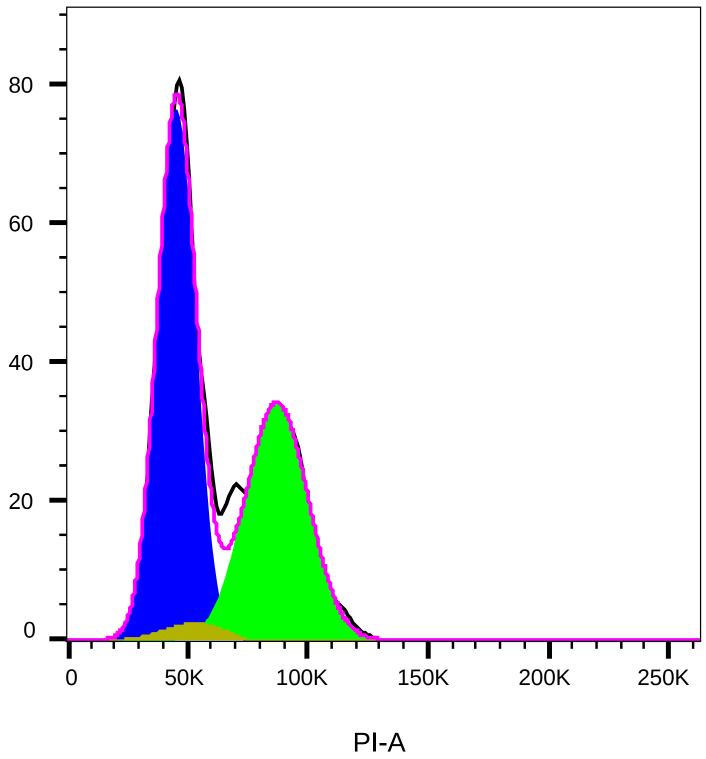

20210326_B1_002_004.fcs
Cell Cycle
2772

: 0.72
%G1 : 57.5
%S : 1.70
%G2 : 41.9
G1 Mean : 45769
G2 Mean : 87270
G1 CV : 24.4
G2 CV : 20.8
% less G1 : -1.43
% greater G2 : 0.16

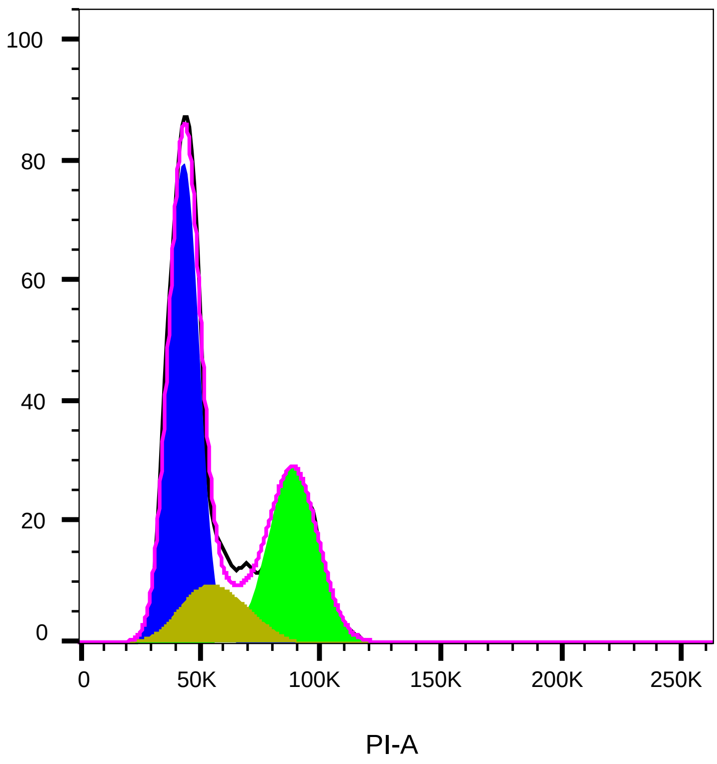

20210326_5_001_006.fcs
Cell Cycle
2316

: 0.75
%G1 : 54.6
%S : 13.6
%G2 : 32.0
G1 Mean : 43735
G2 Mean : 89084
G1 CV : 20.3
G2 CV : 16.1
% less G1 : -2.13
% greater G2 : -0.034

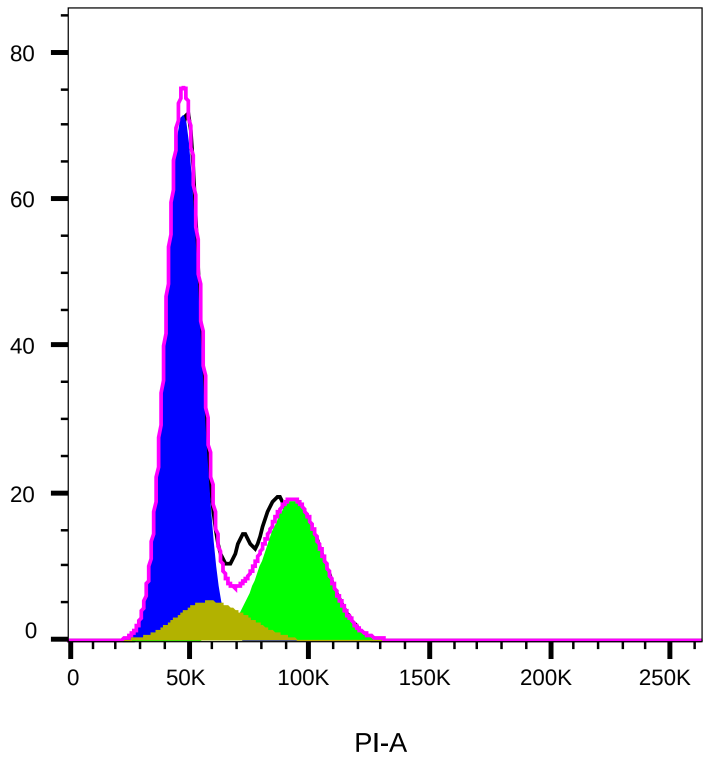

20210326_10_003_012.fcs
Cell Cycle
1982

: 0.75
%G1 : 62.4
%S : 9.10
%G2 : 29.3
G1 Mean : 47887
G2 Mean : 93699
G1 CV : 20.3
G2 CV : 18.4
% less G1 : -1.42
% greater G2 : -0.88

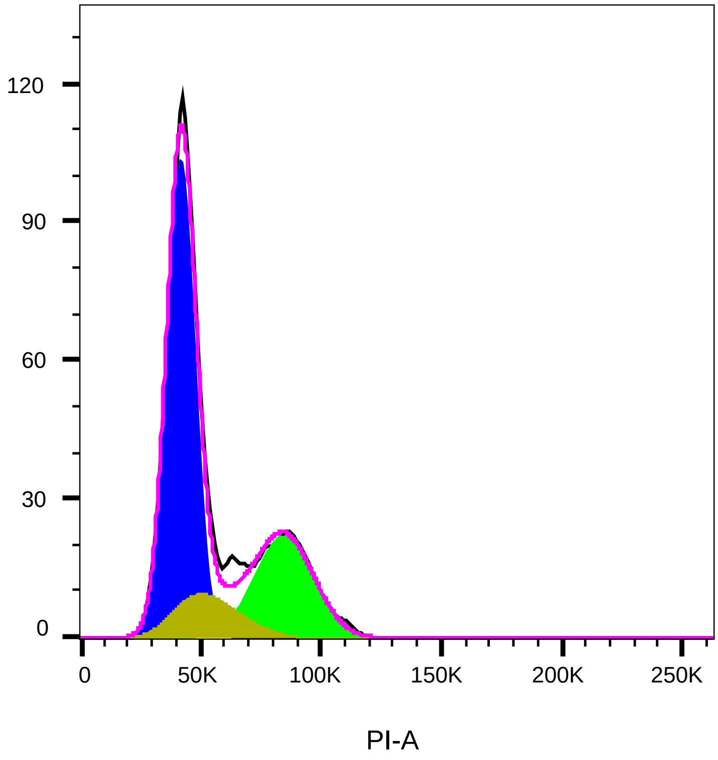

20210326_20_003_016.fcs
Cell Cycle
2656

: 0.71
%G1 : 60.8
%S : 12.3
%G2 : 26.8
G1 Mean : 42288
G2 Mean : 84988
G1 CV : 19.7
G2 CV : 20.3
% less G1 : -2.17
% greater G2 : -0.12
