# Supplementary material for: Protein Lactylation Critically Regulates Energy Metabolism in the Protozoan Parasite Trypanosoma brucei
Source: Front Cell Dev Biol. 2021 Oct 14;9:719720. doi: 10.3389/fcell.2021.719720 (PMC8551762; doi:10.3389/fcell.2021.719720)
Supplement: Supplementary file 4 [file Data_Sheet_4.ZIP › Original Data 2-Flow Cytometry/20210401-Apoptosis/Batch_Analysis_01042021173702.pdf]

BD FACSDiva 8.0.1

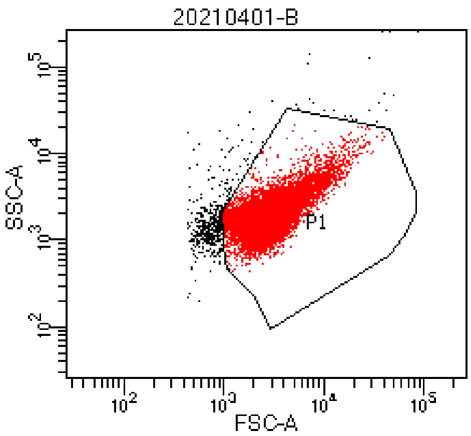

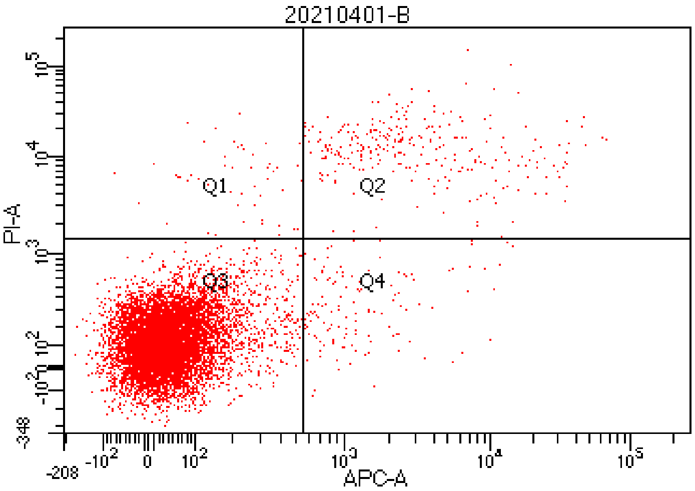

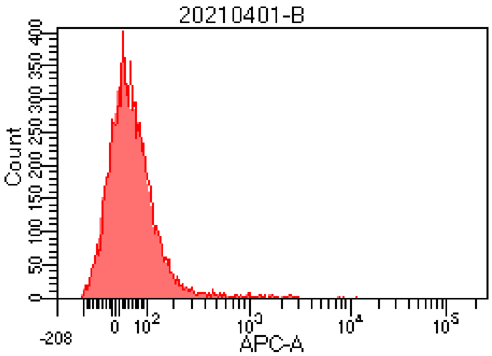

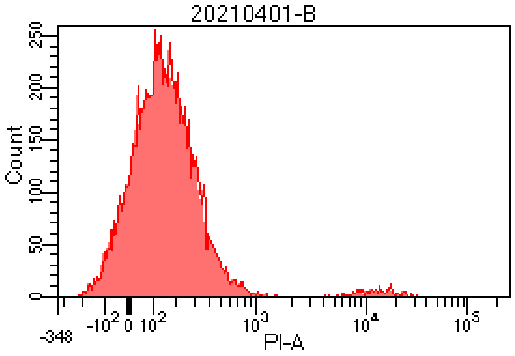

Tube: B

Population	#Events	%Parent	%Total
All Events	10,528	####	100.0
P1	10,000	95.0	95.0
Q1	52	0.5	0.5
Q2	281	2.8	2.7
Q3	9,538	95.4	90.6
Q4	129	1.3	1.2

Experiment Name: Experiment_049
Specimen Name: 20210401
Tube Name: B
Record Date: Apr 1, 2021 3:45:52 PM
$OP: Administrator
GUID: 58b3a1db-dd04-496a-9801-acc...

Population	#Events	%Parent	FSC-A Mean	SSC-A Mean
All Events	10,528	####	3,661	2,564
P1	10,000	95.0	3,755	2,409
Q1	52	0.5	5,239	4,879
Q2	281	2.8	5,228	4,927
Q3	9,538	95.4	3,657	2,294
Q4	129	1.3	7,205	4,444

BD FACSDiva 8.0.1

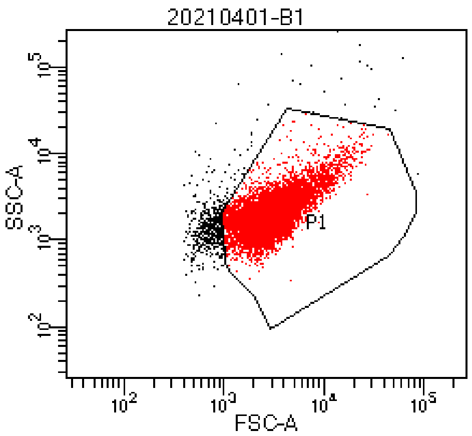

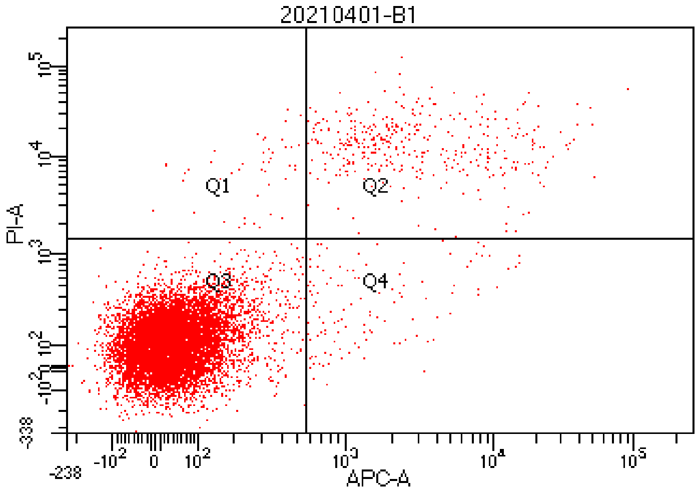

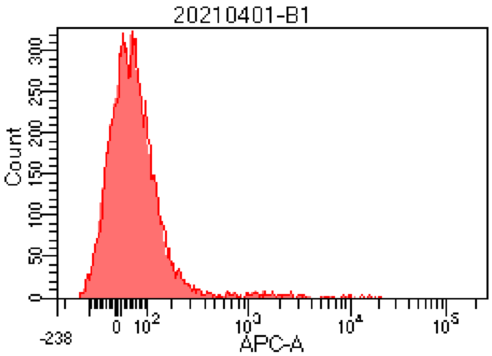

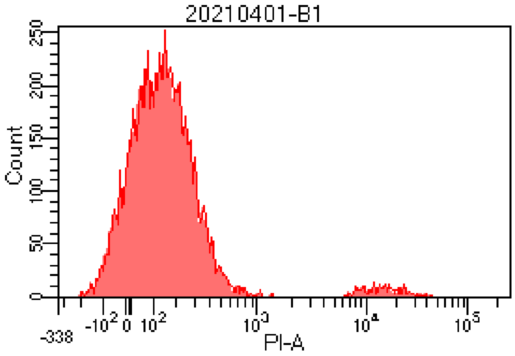

Tube: B1

Population	#Events	%Parent	%Total
All Events	10,597	####	100.0
P1	10,000	94.4	94.4
Q1	46	0.5	0.4
Q2	382	3.8	3.6
Q3	9,458	94.6	89.3
Q4	114	1.1	1.1

Experiment Name:	Experiment_049
Specimen Name:	20210401
Tube Name:	B1
Record Date:	Apr 1, 2021 3:49:13 PM
$OP:	Administrator
GUID:	8176fa3a-a5bf-4469-acac-36c3...

Population	#Events	%Parent	FSC-A Mean	SSC-A Mean
All Events	10,597	####	3,499	2,488
P1	10,000	94.4	3,617	2,357
Q1	46	0.5	2,829	3,907
Q2	382	3.8	4,038	4,427
Q3	9,458	94.6	3,562	2,239
Q4	114	1.1	7,056	4,642

BD FACSDiva 8.0.1

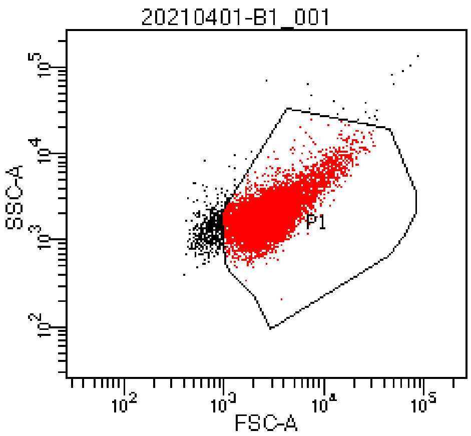

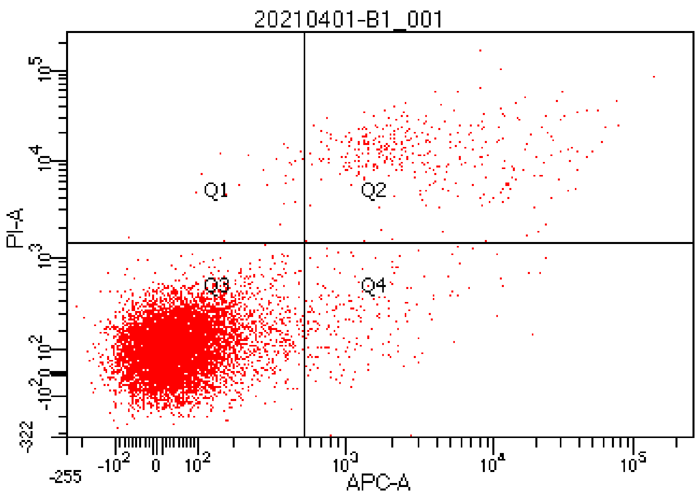

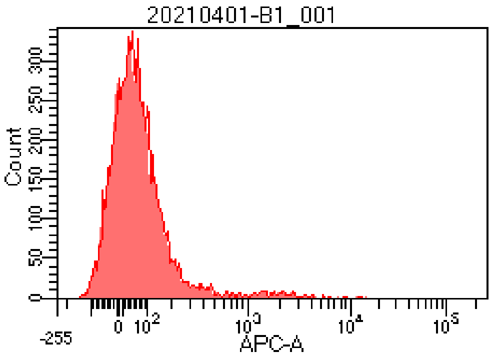

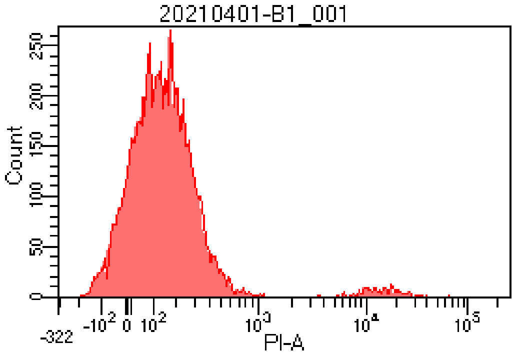

Tube: B1_001

Population	#Events	%Parent	%Total
All Events	10,547	####	100.0
P1	10,000	94.8	94.8
Q1	25	0.2	0.2
Q2	351	3.5	3.3
Q3	9,423	94.2	89.3
Q4	201	2.0	1.9

Experiment Name: Experiment_049
Specimen Name: 20210401
Tube Name: B1_001
Record Date: Apr 1, 2021 3:51:19 PM
$OP: Administrator
GUID: 789f599b-a40f-4f91-a44b-8e9ef...

Population	#Events	%Parent	FSC-A Mean	SSC-A Mean
All Events	10,547	####	3,543	2,440
P1	10,000	94.8	3,633	2,386
Q1	25	0.3	2,228	2,765
Q2	351	3.5	5,079	5,099
Q3	9,423	94.2	3,509	2,244
Q4	201	2.0	7,077	4,286

BD FACSDiva 8.0.1

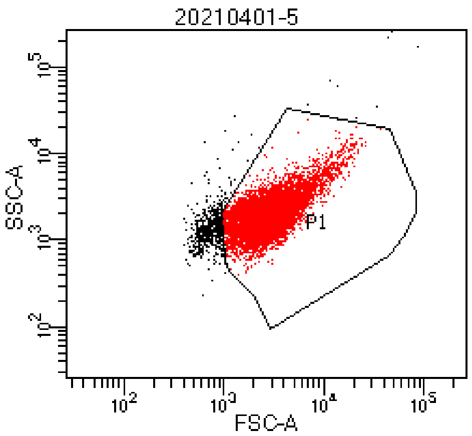

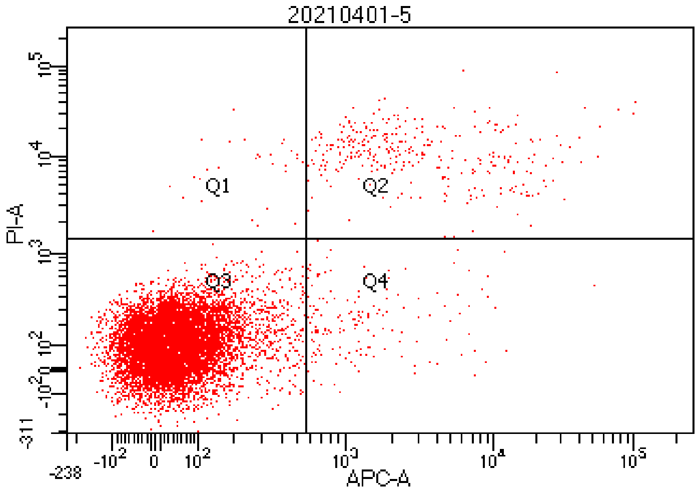

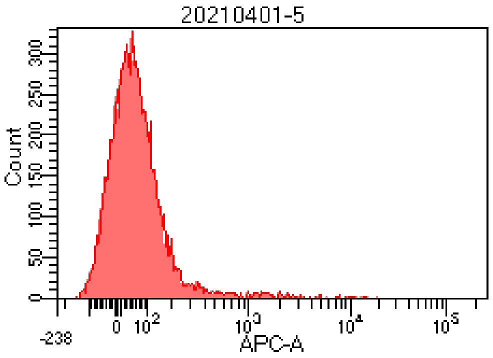

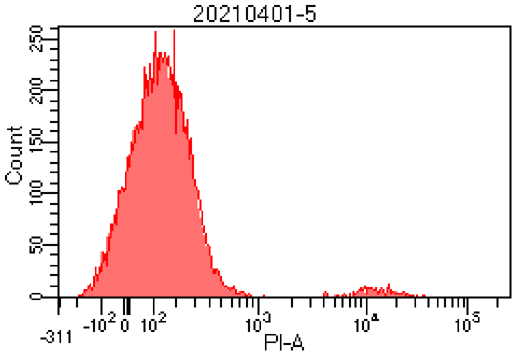

Tube: 5

Population	#Events	%Parent	%Total
All Events	10,621	####	100.0
P1	10,000	94.2	94.2
Q1	29	0.3	0.3
Q2	322	3.2	3.0
Q3	9,487	94.9	89.3
Q4	162	1.6	1.5

Experiment Name:	Experiment_049
Specimen Name:	20210401
Tube Name:	5
Record Date:	Apr 1, 2021 3:53:38 PM
$OP:	Administrator
GUID:	fcd43457-be20-405b-b8e8-3a9...

Population	#Events	%Parent	FSC-A Mean	SSC-A Mean
All Events	10,621	####	3,187	2,300
P1	10,000	94.2	3,312	2,247
Q1	29	0.3	2,858	4,247
Q2	322	3.2	3,910	3,948
Q3	9,487	94.9	3,249	2,158
Q4	162	1.6	5,898	3,754

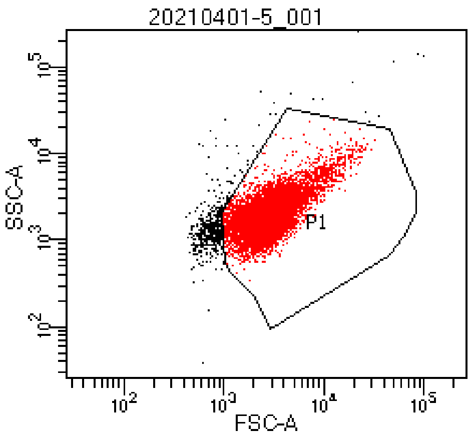

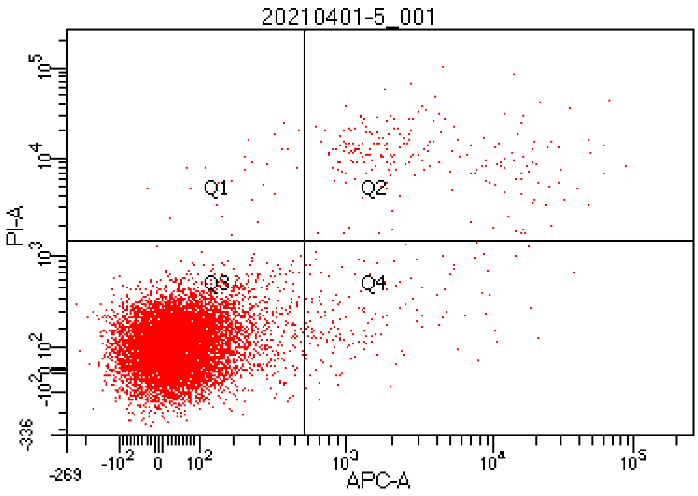

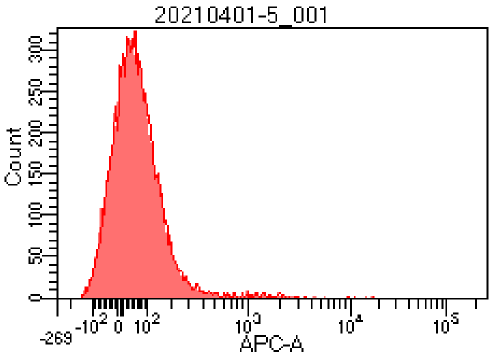

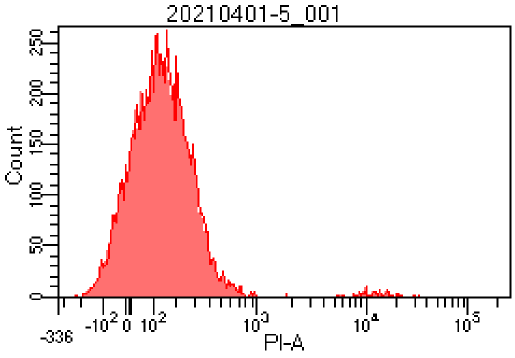

Tube: 5_001

Population	#Events	%Parent	%Total
All Events	10,518	####	100.0
P1	10,000	95.1	95.1
Q1	25	0.2	0.2
Q2	211	2.1	2.0
Q3	9,604	96.0	91.3
Q4	160	1.6	1.5

Experiment Name:	Experiment_049
Specimen Name:	20210401
Tube Name:	5_001
Record Date:	Apr 1, 2021 3:56:03 PM
$OP:	Administrator
GUID:	5c5d6cba-4959-4e1e-845c-488...

Population	#Events	%Parent	FSC-A Mean	SSC-A Mean
All Events	10,518	####	3,309	2,338
P1	10,000	95.1	3,401	2,256
Q1	25	0.3	2,149	3,122
Q2	211	2.1	4,466	4,564
Q3	9,604	96.0	3,339	2,175
Q4	160	1.6	5,923	3,980

BD FACSDiva 8.0.1

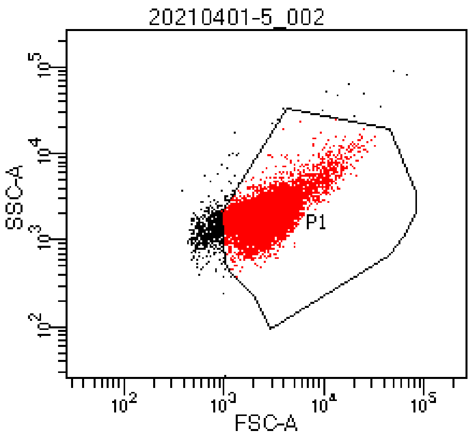

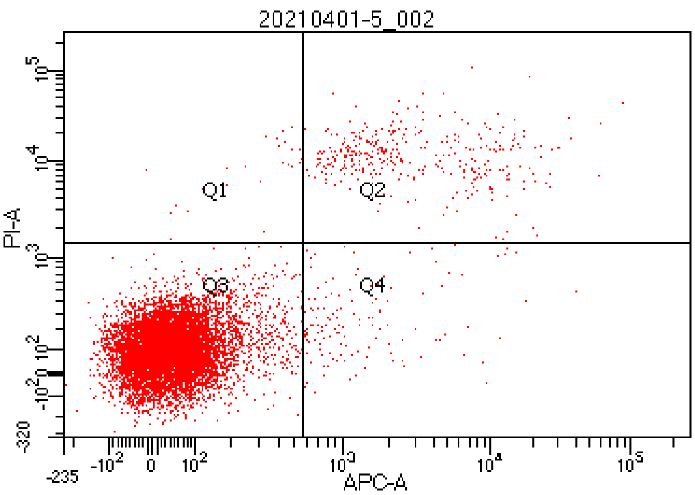

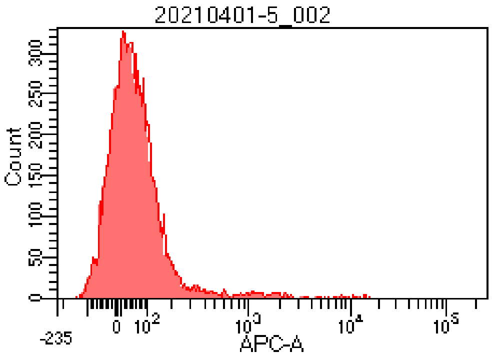

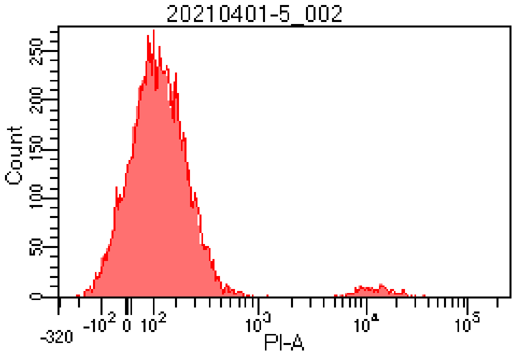

Tube: 5_002

Population	#Events	%Parent	%Total
All Events	10,669	####	100.0
P1	10,000	93.7	93.7
Q1	25	0.2	0.2
Q2	359	3.6	3.4
Q3	9,481	94.8	88.9
Q4	135	1.4	1.3

Experiment Name:	Experiment_049
Specimen Name:	20210401
Tube Name:	5_002
Record Date:	Apr 1, 2021 3:58:18 PM
$OP:	Administrator
GUID:	01bfff21-91b1-40f4-917c-da637...

Population	#Events	%Parent	FSC-A Mean	SSC-A Mean
All Events	10,669	####	3,207	2,256
P1	10,000	93.7	3,343	2,246
Q1	25	0.3	2,987	2,851
Q2	359	3.6	3,542	3,813
Q3	9,481	94.8	3,293	2,159
Q4	135	1.4	6,420	4,122

BD FACSDiva 8.0.1

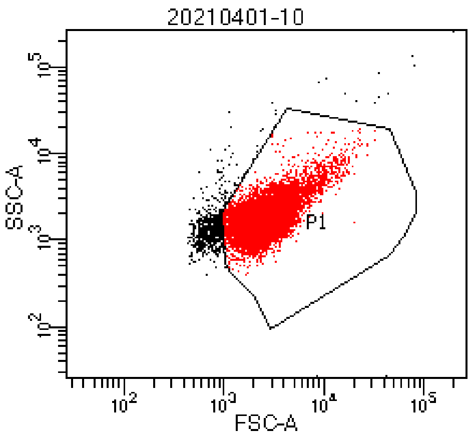

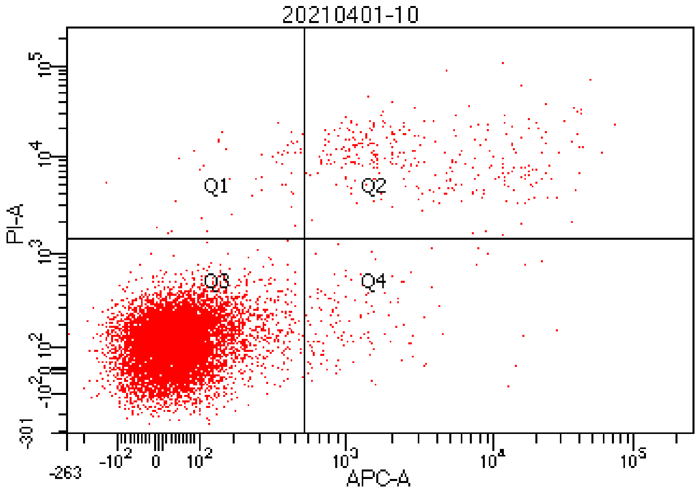

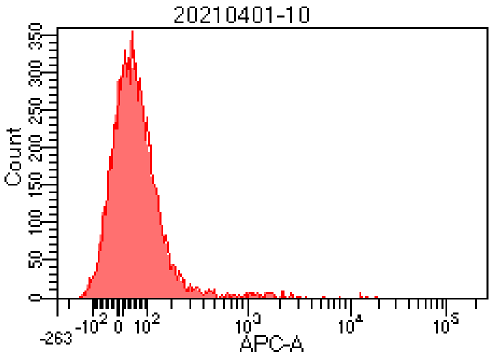

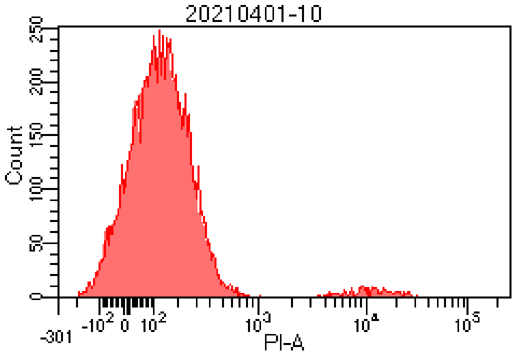

Tube: 10

Population	#Events	%Parent	%Total
All Events	10,742	####	100.0
P1	10,000	93.1	93.1
Q1	46	0.5	0.4
Q2	314	3.1	2.9
Q3	9,508	95.1	88.5
Q4	132	1.3	1.2

Experiment Name: Experiment_049
Specimen Name: 20210401
Tube Name: 10
Record Date: Apr 1, 2021 4:00:23 PM
$OP: Administrator
GUID: 1b250e3c-a696-4660-ae23-3c1...

Population	#Events	%Parent	FSC-A Mean	SSC-A Mean
All Events	10,742	####	3,057	2,257
P1	10,000	93.1	3,162	2,184
Q1	46	0.5	1,762	2,761
Q2	314	3.1	4,054	4,094
Q3	9,508	95.1	3,102	2,096
Q4	132	1.3	5,884	3,768

BD FACSDiva 8.0.1

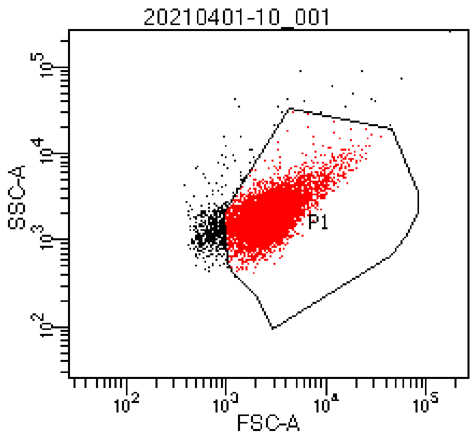

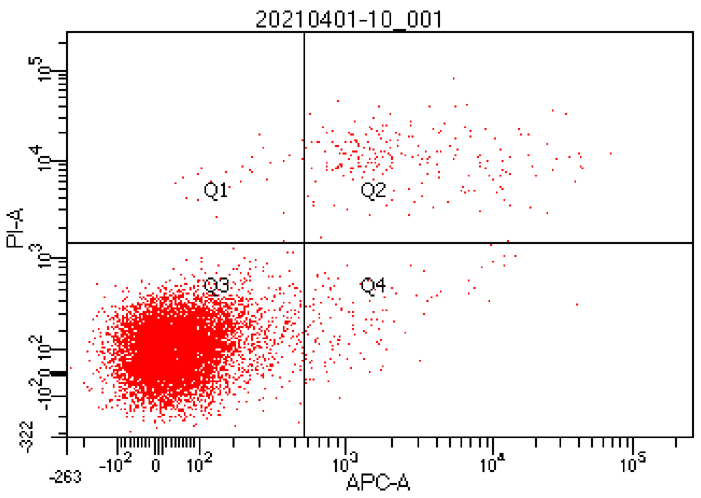

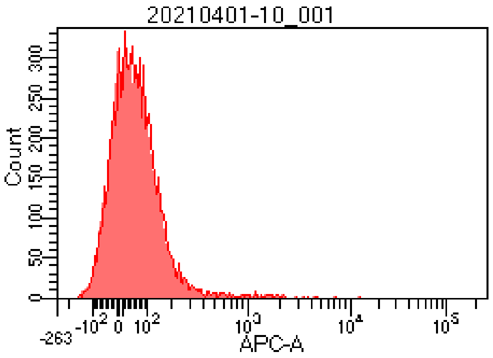

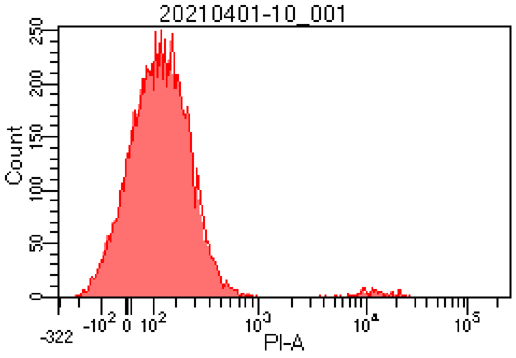

Tube: 10_001

Population	#Events	%Parent	%Total
All Events	10,598	####	100.0
P1	10,000	94.4	94.4
Q1	27	0.3	0.3
Q2	213	2.1	2.0
Q3	9,635	96.4	90.9
Q4	125	1.2	1.2

Experiment Name:	Experiment_049
Specimen Name:	20210401
Tube Name:	10_001
Record Date:	Apr 1, 2021 4:02:32 PM
$OP:	Administrator
GUID:	96558825-ac88-48a1-8e30-572...

Population	#Events	%Parent	FSC-A Mean	SSC-A Mean
All Events	10,598	####	3,053	2,232
P1	10,000	94.4	3,147	2,159
Q1	27	0.3	3,107	3,233
Q2	213	2.1	3,372	3,848
Q3	9,635	96.4	3,107	2,099
Q4	125	1.3	5,798	3,724

BD FACSDiva 8.0.1

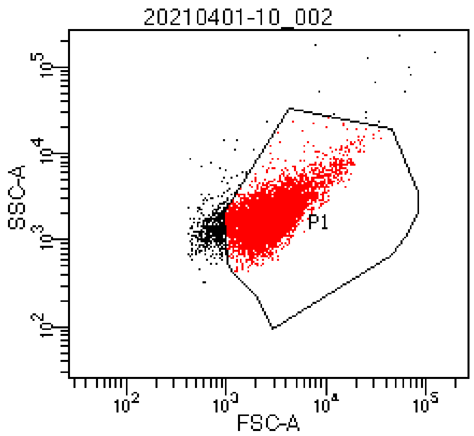

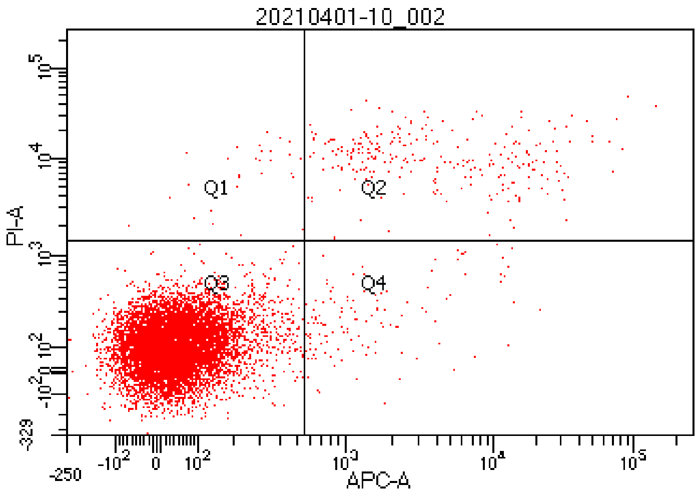

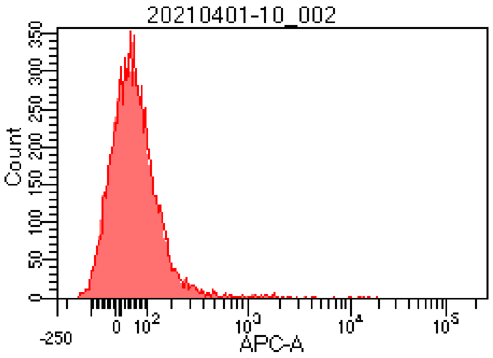

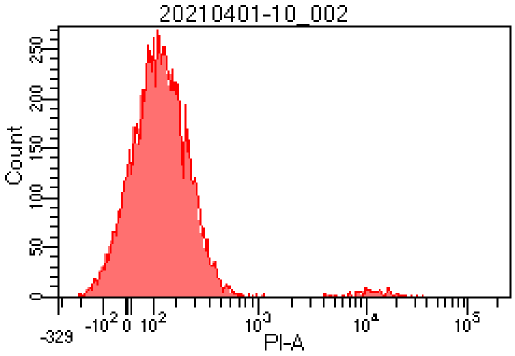

Tube: 10_002

Population	#Events	%Parent	%Total
All Events	10,563	####	100.0
P1	10,000	94.7	94.7
Q1	29	0.3	0.3
Q2	263	2.6	2.5
Q3	9,584	95.8	90.7
Q4	124	1.2	1.2

Experiment Name:	Experiment_049
Specimen Name:	20210401
Tube Name:	10_002
Record Date:	Apr 1, 2021 4:04:58 PM
$OP:	Administrator
GUID:	73e5e4f7-de8e-481a-9de7-9af5...

Population	#Events	%Parent	FSC-A Mean	SSC-A Mean
All Events	10,563	####	3,165	2,271
P1	10,000	94.7	3,247	2,192
Q1	29	0.3	3,021	3,086
Q2	263	2.6	4,907	5,021
Q3	9,584	95.8	3,165	2,091
Q4	124	1.2	6,098	3,770

BD FACSDiva 8.0.1

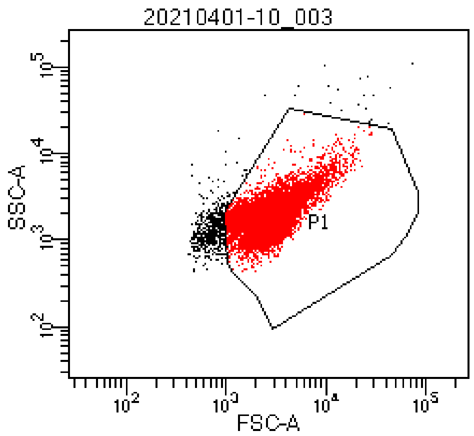

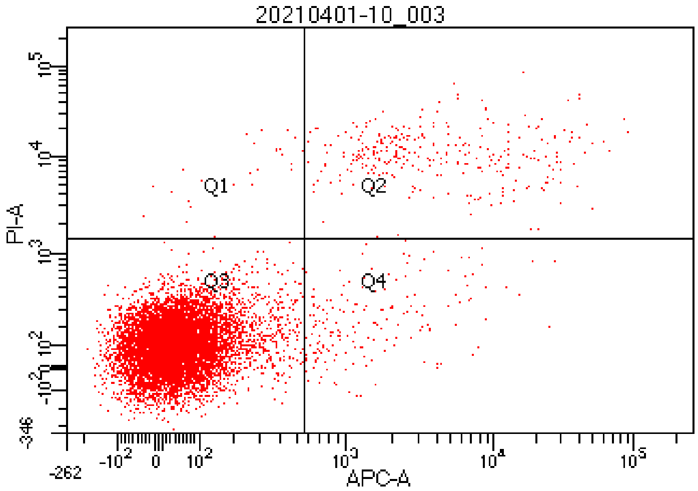

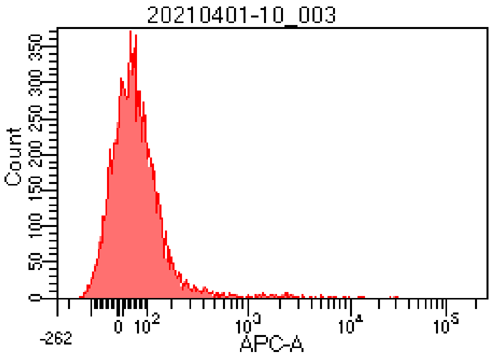

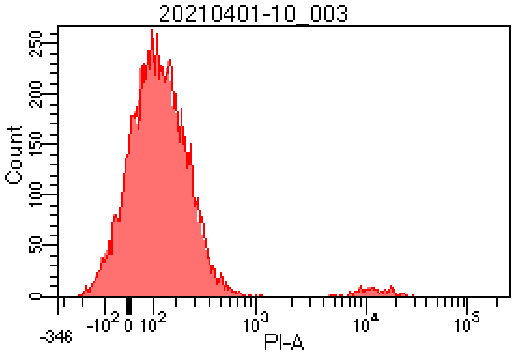

Tube: 10_003

Population	#Events	%Parent	%Total
All Events	10,670	####	100.0
P1	10,000	93.7	93.7
Q1	26	0.3	0.2
Q2	308	3.1	2.9
Q3	9,493	94.9	89.0
Q4	173	1.7	1.6

Experiment Name: Experiment_049
Specimen Name: 20210401
Tube Name: 10_003
Record Date: Apr 1, 2021 4:06:58 PM
$OP: Administrator
GUID: 19fb6c06-05e9-4a79-a723-6da...

Population	#Events	%Parent	FSC-A Mean	SSC-A Mean
All Events	10,670	####	3,160	2,272
P1	10,000	93.7	3,277	2,216
Q1	26	0.3	2,029	2,956
Q2	308	3.1	4,233	4,446
Q3	9,493	94.9	3,207	2,116
Q4	173	1.7	5,643	3,660

BD FACSDiva 8.0.1

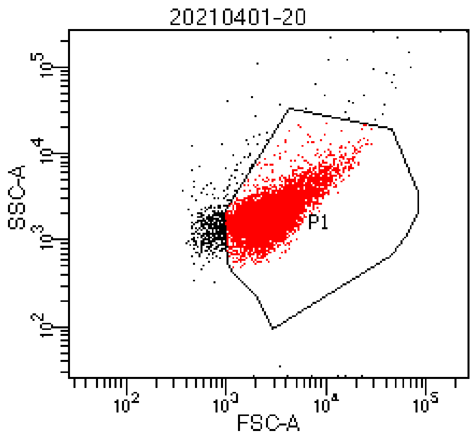

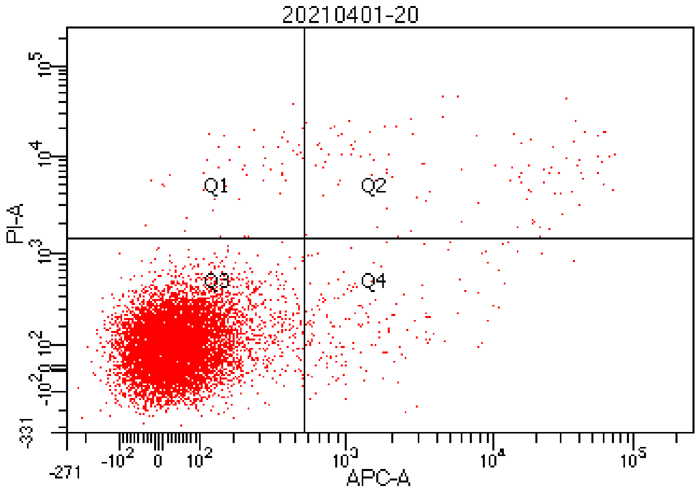

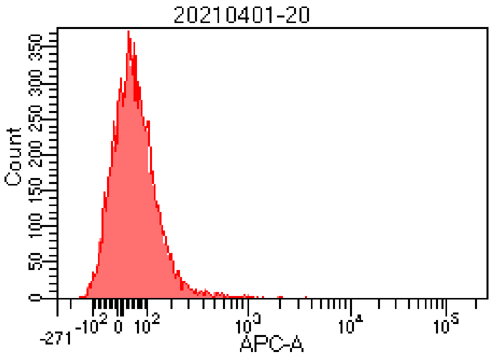

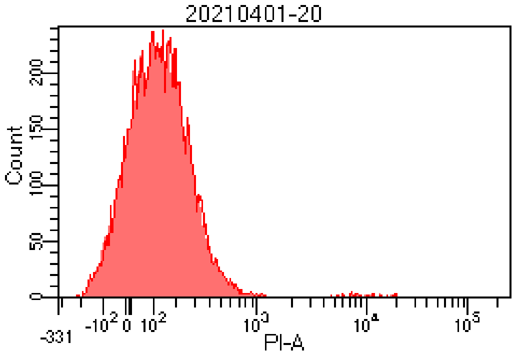

Tube: 20

Population	#Events	%Parent	%Total
All Events	10,522	####	100.0
P1	10,000	95.0	95.0
Q1	50	0.5	0.5
Q2	102	1.0	1.0
Q3	9,680	96.8	92.0
Q4	168	1.7	1.6

Experiment Name:	Experiment_049
Specimen Name:	20210401
Tube Name:	20
Record Date:	Apr 1, 2021 4:10:06 PM
$OP:	Administrator
GUID:	f9b52b0a-38b9-4fff-a55c-88f39...

Population	#Events	%Parent	FSC-A Mean	SSC-A Mean
All Events	10,522	####	3,322	2,482
P1	10,000	95.0	3,340	2,233
Q1	50	0.5	2,830	3,562
Q2	102	1.0	4,870	6,124
Q3	9,680	96.8	3,283	2,156
Q4	168	1.7	5,849	3,922

BD FACSDiva 8.0.1

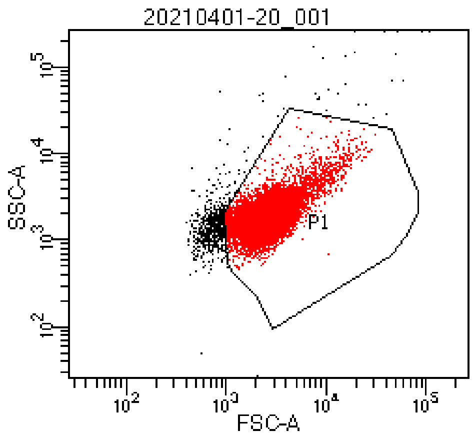

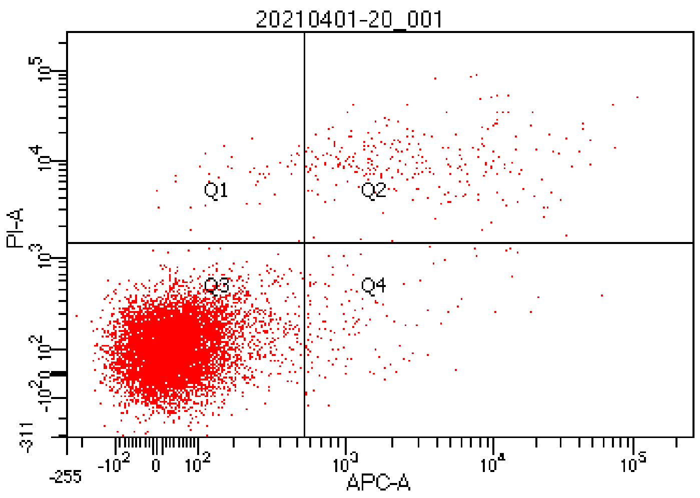

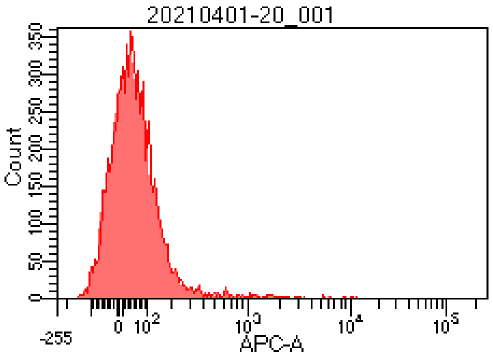

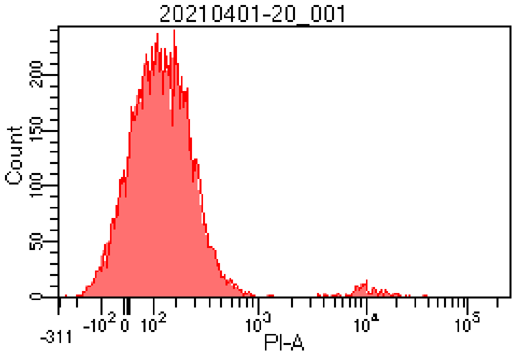

Tube: 20_001

Population	#Events	%Parent	%Total
All Events	10,694	####	100.0
P1	10,000	93.5	93.5
Q1	41	0.4	0.4
Q2	268	2.7	2.5
Q3	9,562	95.6	89.4
Q4	129	1.3	1.2

Experiment Name: Experiment_049
Specimen Name: 20210401
Tube Name: 20_001
Record Date: Apr 1, 2021 4:12:49 PM
$OP: Administrator
GUID: 07ef6132-8db4-47d9-94f7-7bd8...

Population	#Events	%Parent	FSC-A Mean	SSC-A Mean
All Events	10,694	####	3,168	2,504
P1	10,000	93.5	3,263	2,260
Q1	41	0.4	2,331	3,183
Q2	268	2.7	3,842	4,383
Q3	9,562	95.6	3,209	2,165
Q4	129	1.3	6,414	4,605

BD FACSDiva 8.0.1

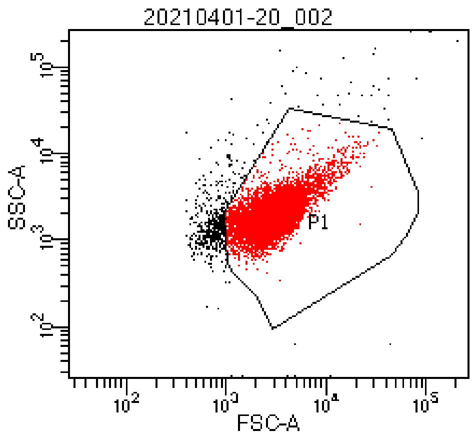

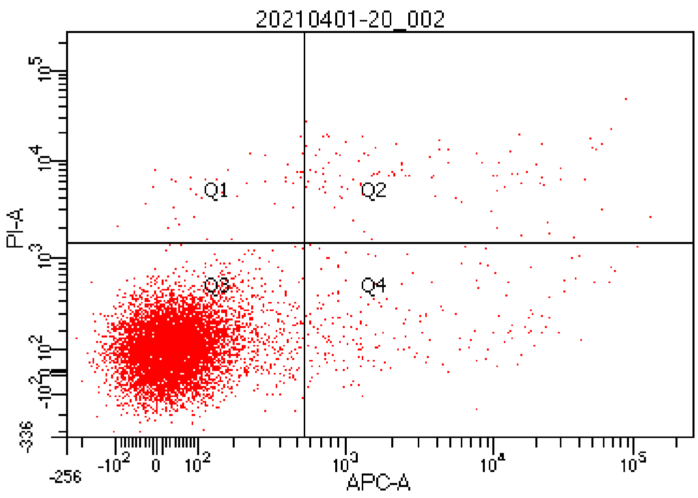

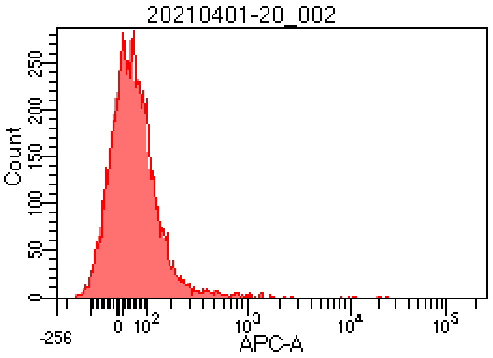

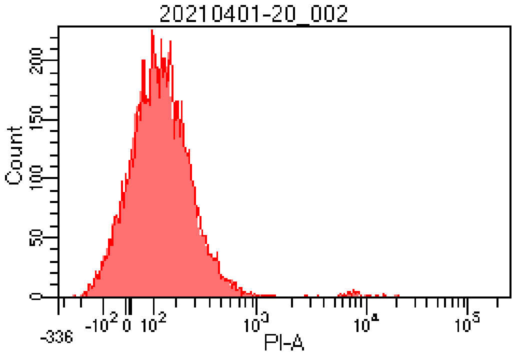

Tube: 20_002

Population	#Events	%Parent	%Total
All Events	8,811	####	100.0
P1	8,258	93.7	93.7
Q1	42	0.5	0.5
Q2	100	1.2	1.1
Q3	7,920	95.9	89.9
Q4	196	2.4	2.2

Experiment Name:	Experiment_049
Specimen Name:	20210401
Tube Name:	20_002
Record Date:	Apr 1, 2021 4:17:05 PM
$OP:	Administrator
GUID:	5cac6c70-2f44-4680-8e2e-519...

Population	#Events	%Parent	FSC-A Mean	SSC-A Mean
All Events	8,811	####	3,381	2,629
P1	8,258	93.7	3,381	2,267
Q1	42	0.5	2,875	3,867
Q2	100	1.2	3,878	4,843
Q3	7,920	95.9	3,325	2,177
Q4	196	2.4	5,522	4,253
