# Supplementary material for: Protein Lactylation Critically Regulates Energy Metabolism in the Protozoan Parasite Trypanosoma brucei
Source: Front Cell Dev Biol. 2021 Oct 14;9:719720. doi: 10.3389/fcell.2021.719720 (PMC8551762; doi:10.3389/fcell.2021.719720)
Supplement: Supplementary file 4 [file Data_Sheet_4.ZIP › Original Data 2-Flow Cytometry/20210326-Cell cycle/Batch_Analysis_26032021125211.pdf]

BD FACSDiva 8.0.1

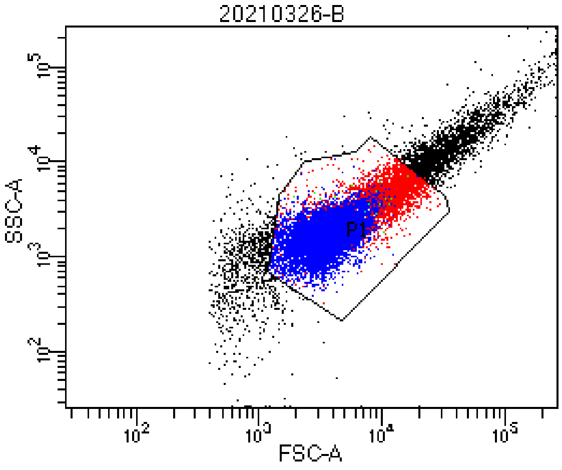

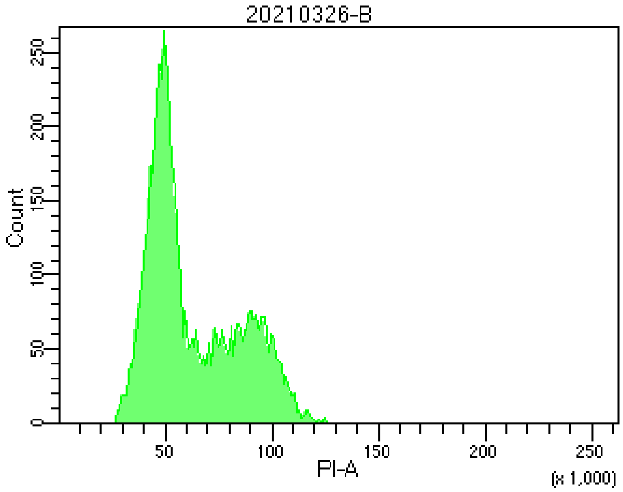

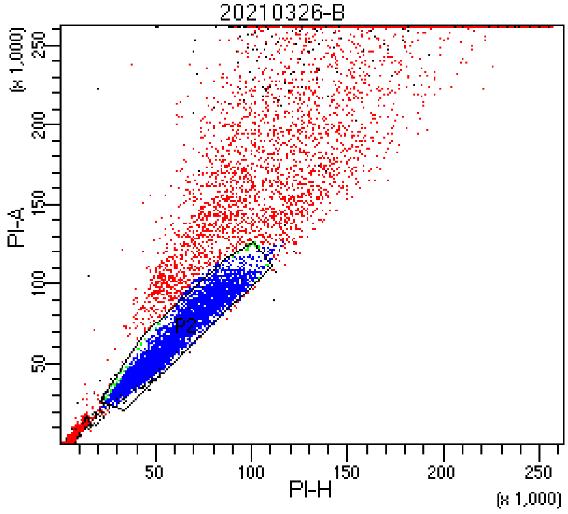

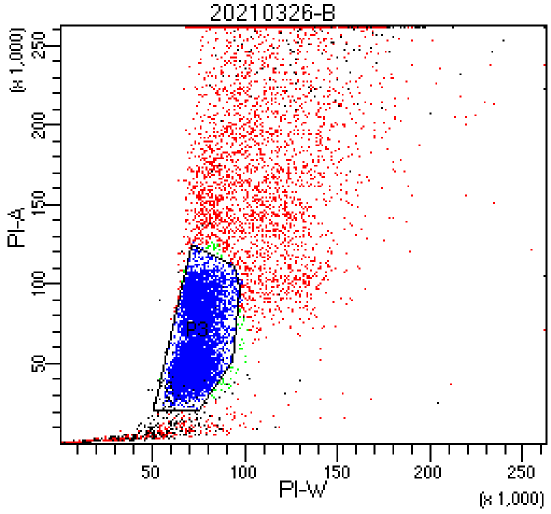

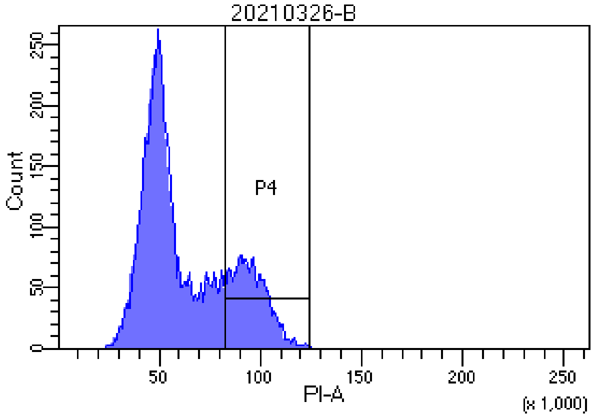

Tube: B

Population	#Events	%Parent	%Total
All Events	13,033	####	100.0
P1	10,000	76.7	76.7
P2	6,685	66.8	51.3
P3	6,696	67.0	51.4
P4	1,568	23.4	12.0

BD FACSDiva 8.0.1

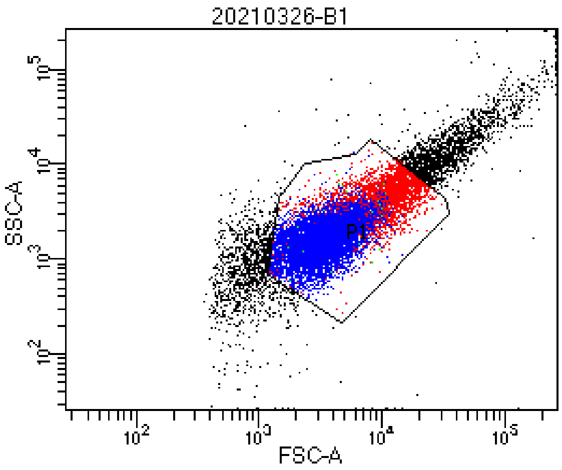

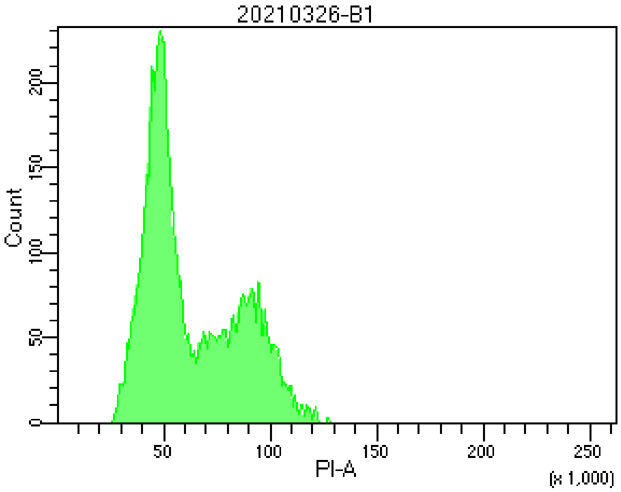

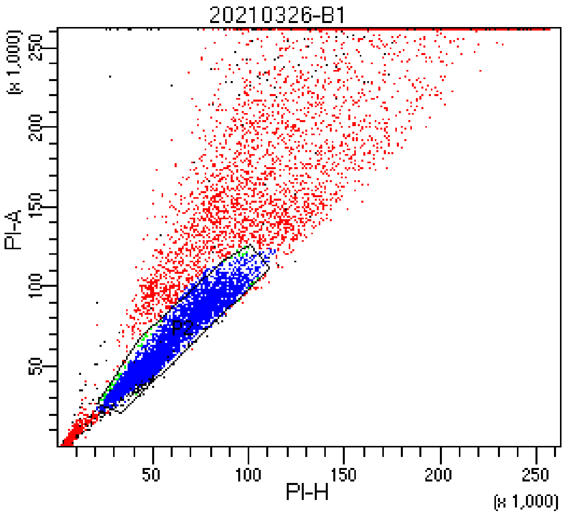

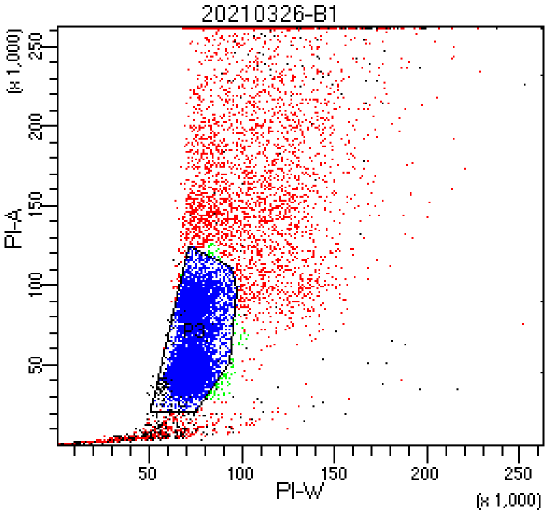

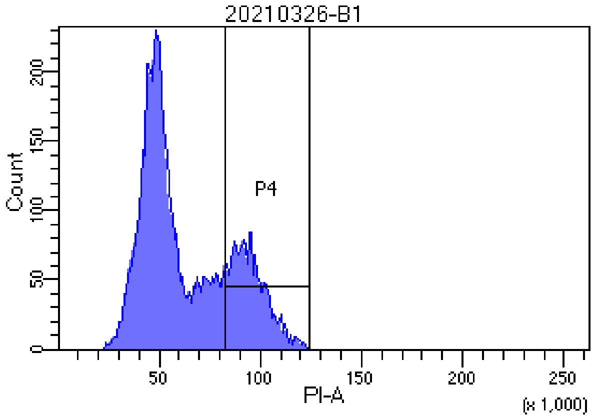

Tube: B1

Population	#Events	%Parent	%Total
All Events	12,886	####	100.0
P1	10,000	77.6	77.6
P2	6,372	63.7	49.4
P3	6,346	63.5	49.2
P4	1,572	24.8	12.2

BD FACSDiva 8.0.1

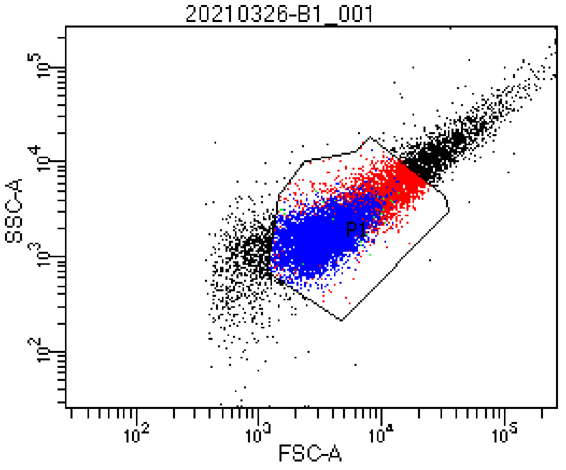

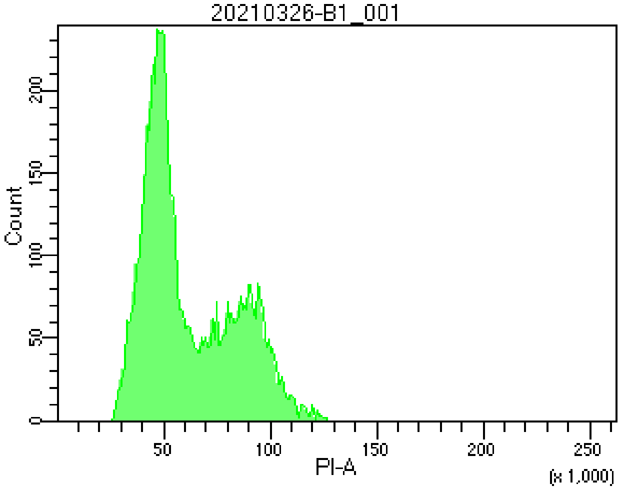

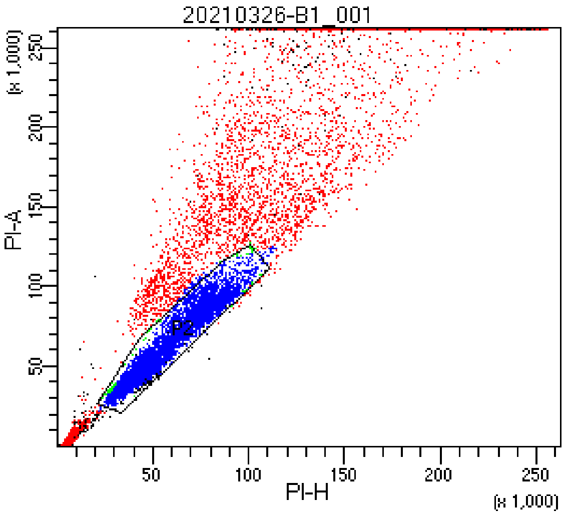

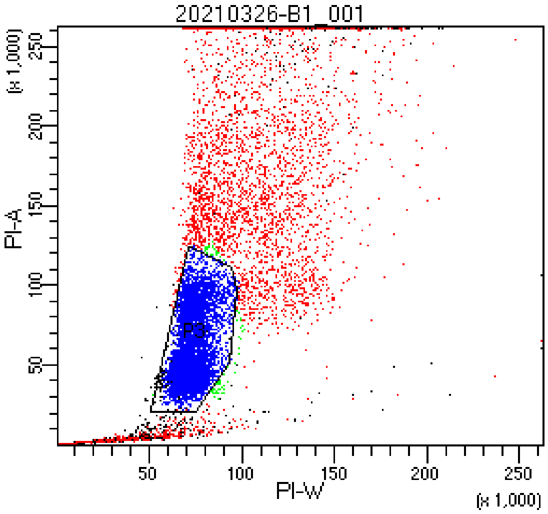

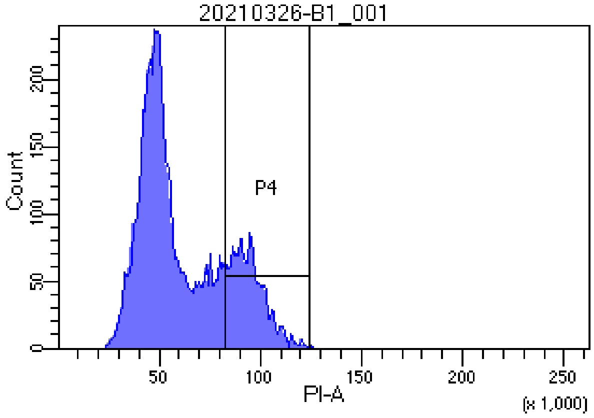

Tube: B1_001

Population	#Events	%Parent	%Total
All Events	12,602	####	100.0
P1	10,000	79.4	79.4
P2	6,661	66.6	52.9
P3	6,642	66.4	52.7
P4	1,475	22.2	11.7

BD FACSDiva 8.0.1

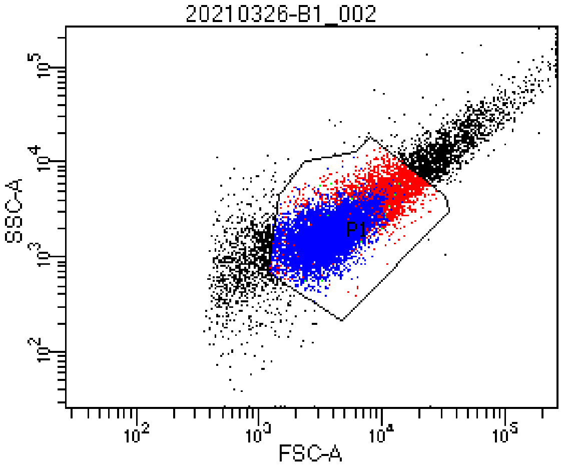

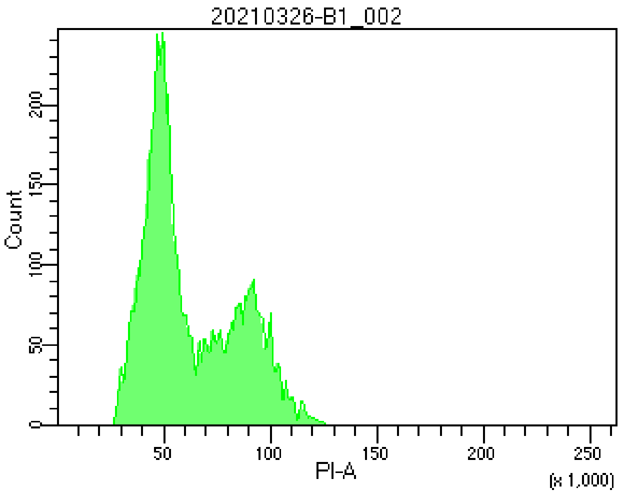

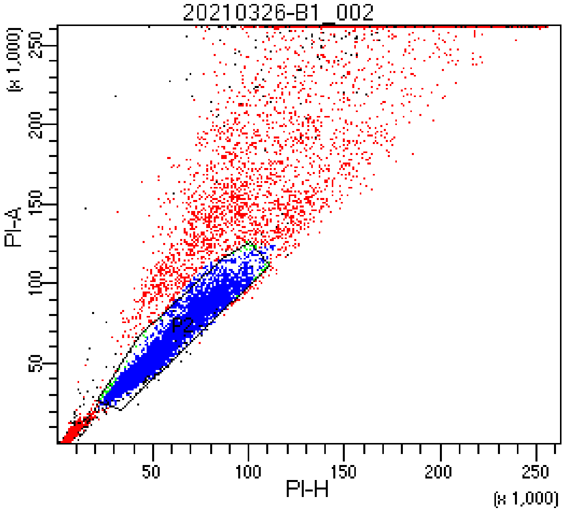

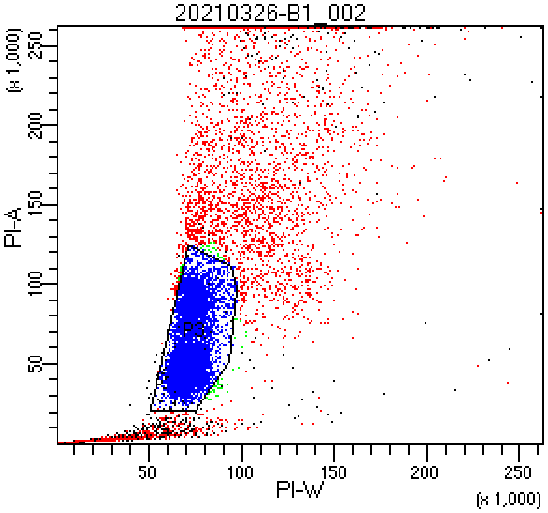

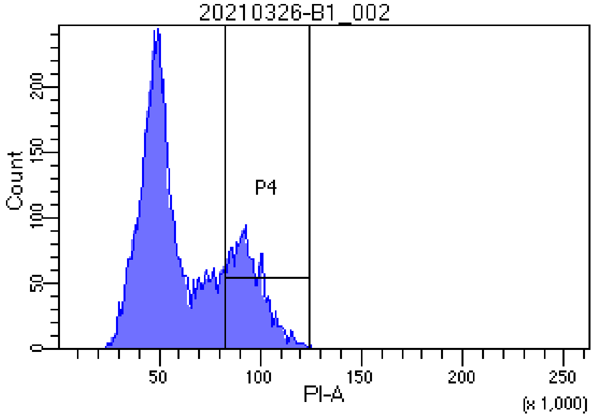

Tube: B1_002

Population	#Events	%Parent	%Total
All Events	12,796	####	100.0
P1	10,000	78.1	78.1
P2	6,727	67.3	52.6
P3	6,745	67.4	52.7
P4	1,629	24.2	12.7

BD FACSDiva 8.0.1

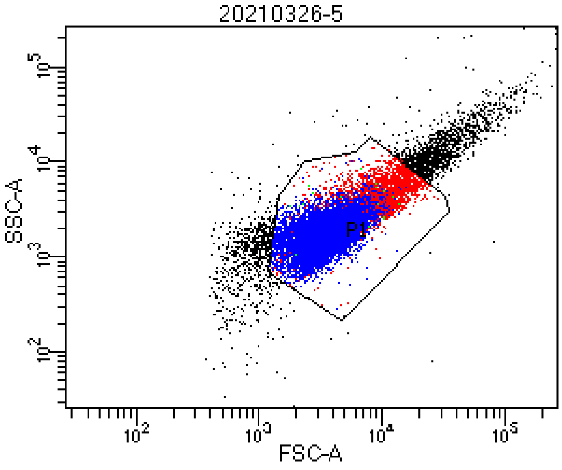

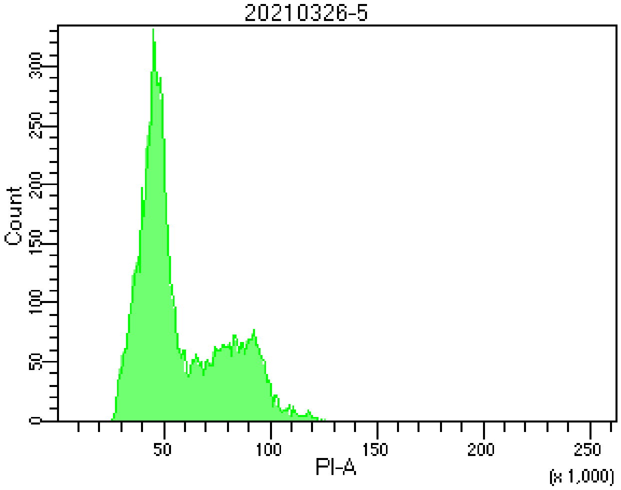

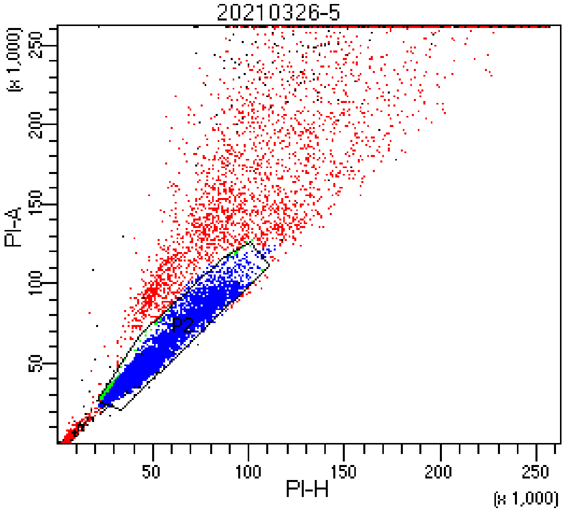

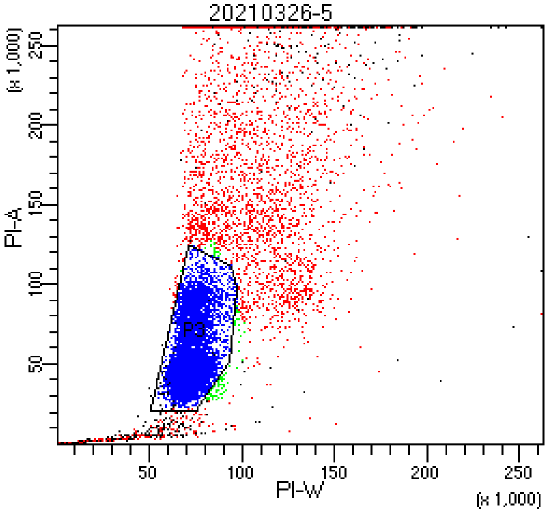

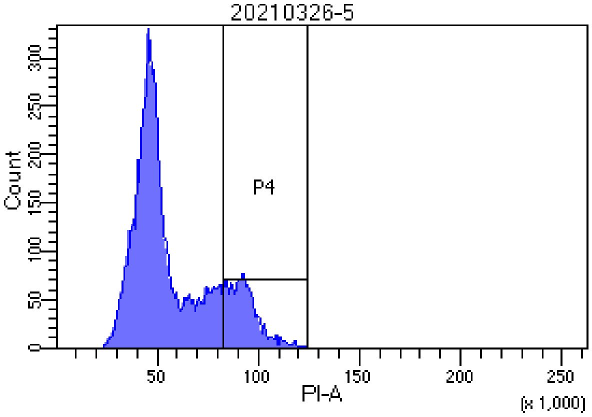

Tube: 5

Population	#Events	%Parent	%Total
All Events	12,097	####	100.0
P1	10,000	82.7	82.7
P2	7,209	72.1	59.6
P3	7,194	71.9	59.5
P4	1,236	17.2	10.2

BD FACSDiva 8.0.1

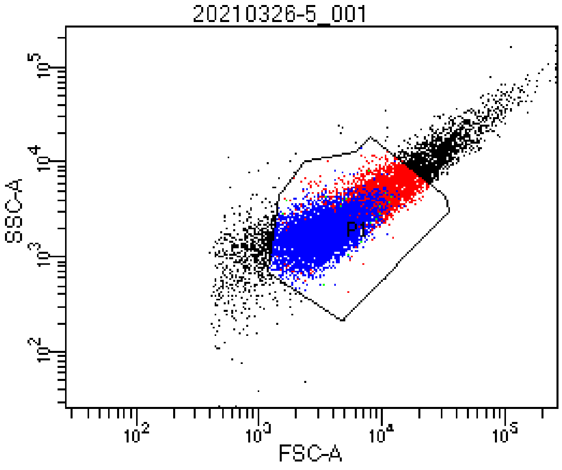

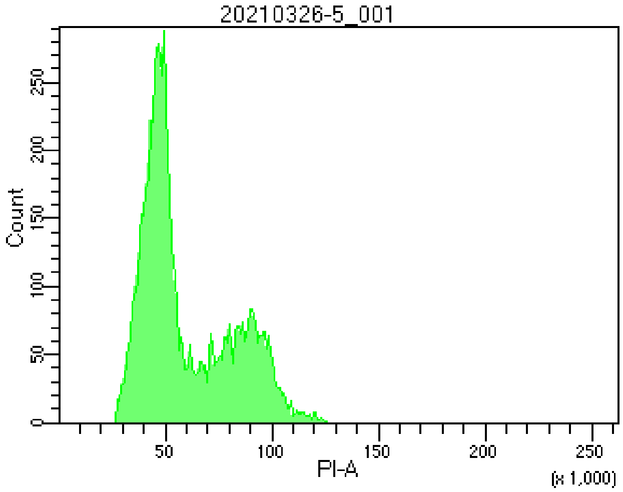

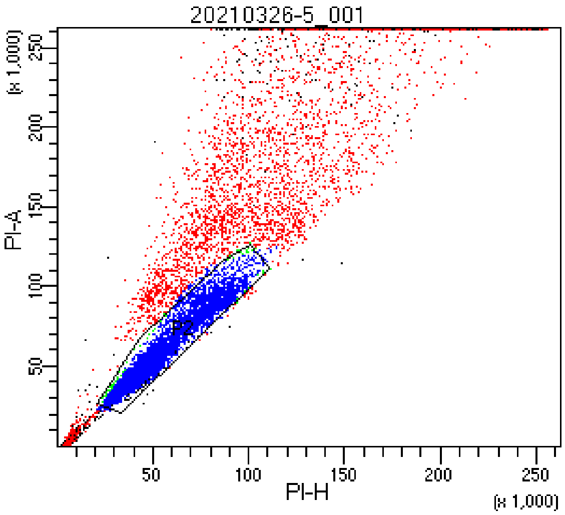

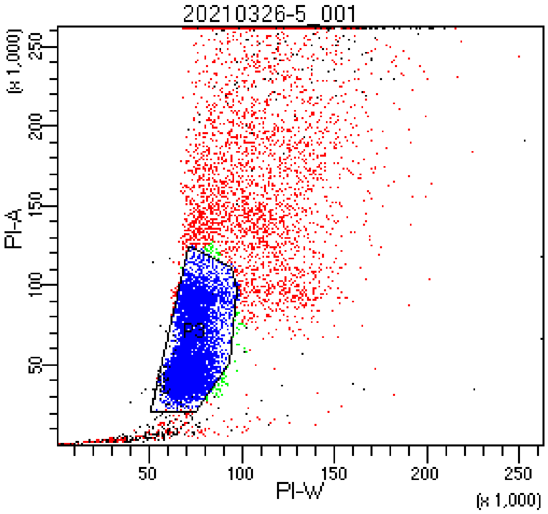

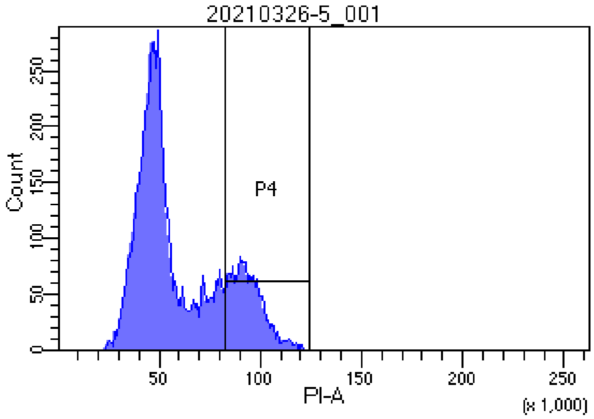

Tube: 5_001

Population	#Events	%Parent	%Total
All Events	12,199	####	100.0
P1	10,000	82.0	82.0
P2	6,932	69.3	56.8
P3	6,915	69.2	56.7
P4	1,437	20.8	11.8

BD FACSDiva 8.0.1

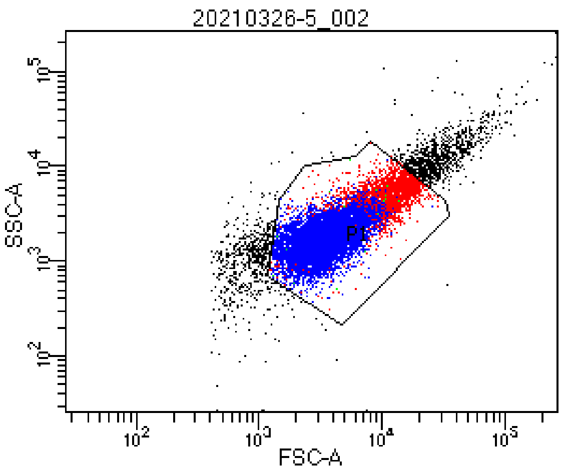

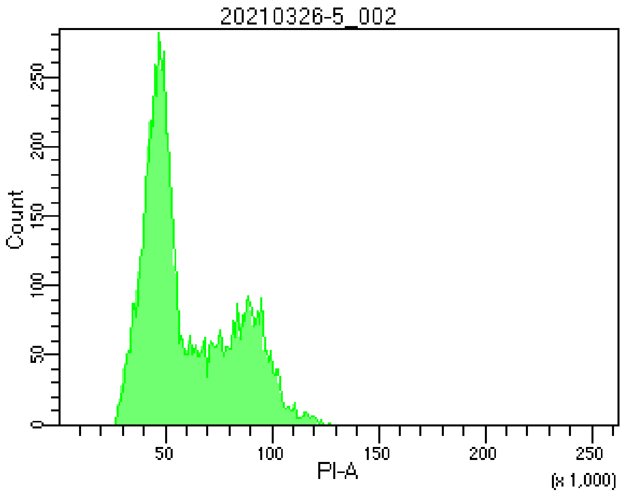

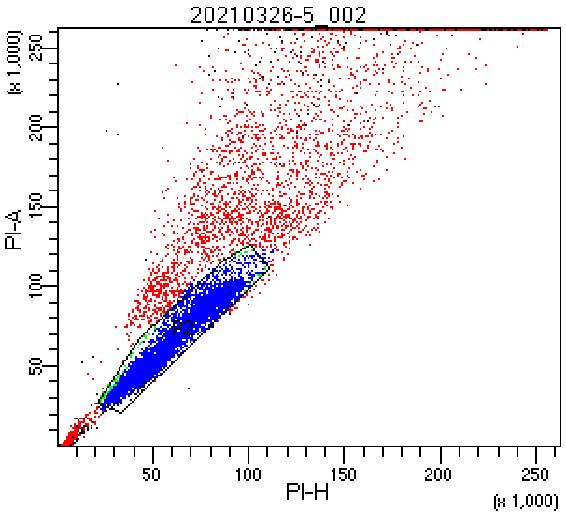

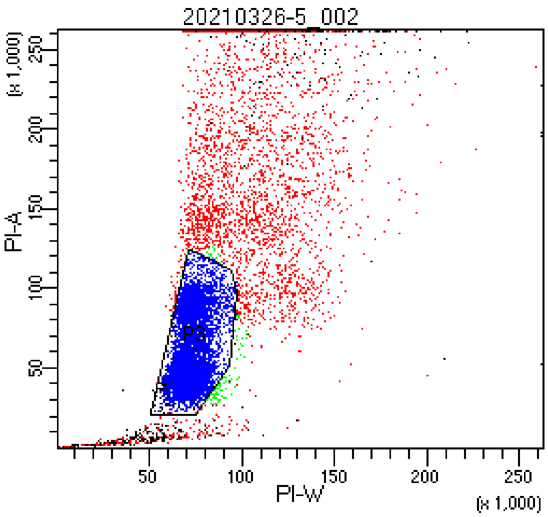

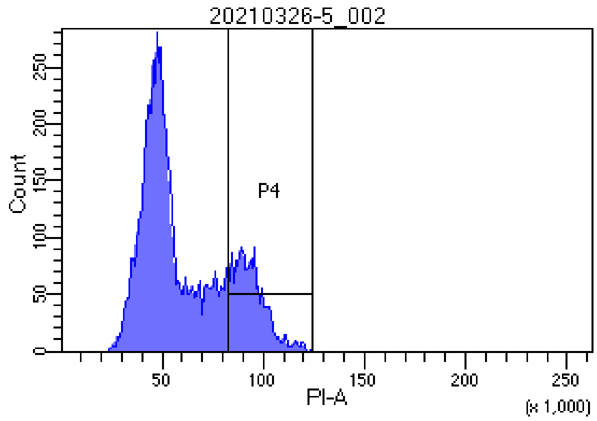

Tube: 5_002

Population	#Events	%Parent	%Total
All Events	11,644	####	100.0
P1	10,000	85.9	85.9
P2	7,135	71.4	61.3
P3	7,093	70.9	60.9
P4	1,525	21.5	13.1

BD FACSDiva 8.0.1

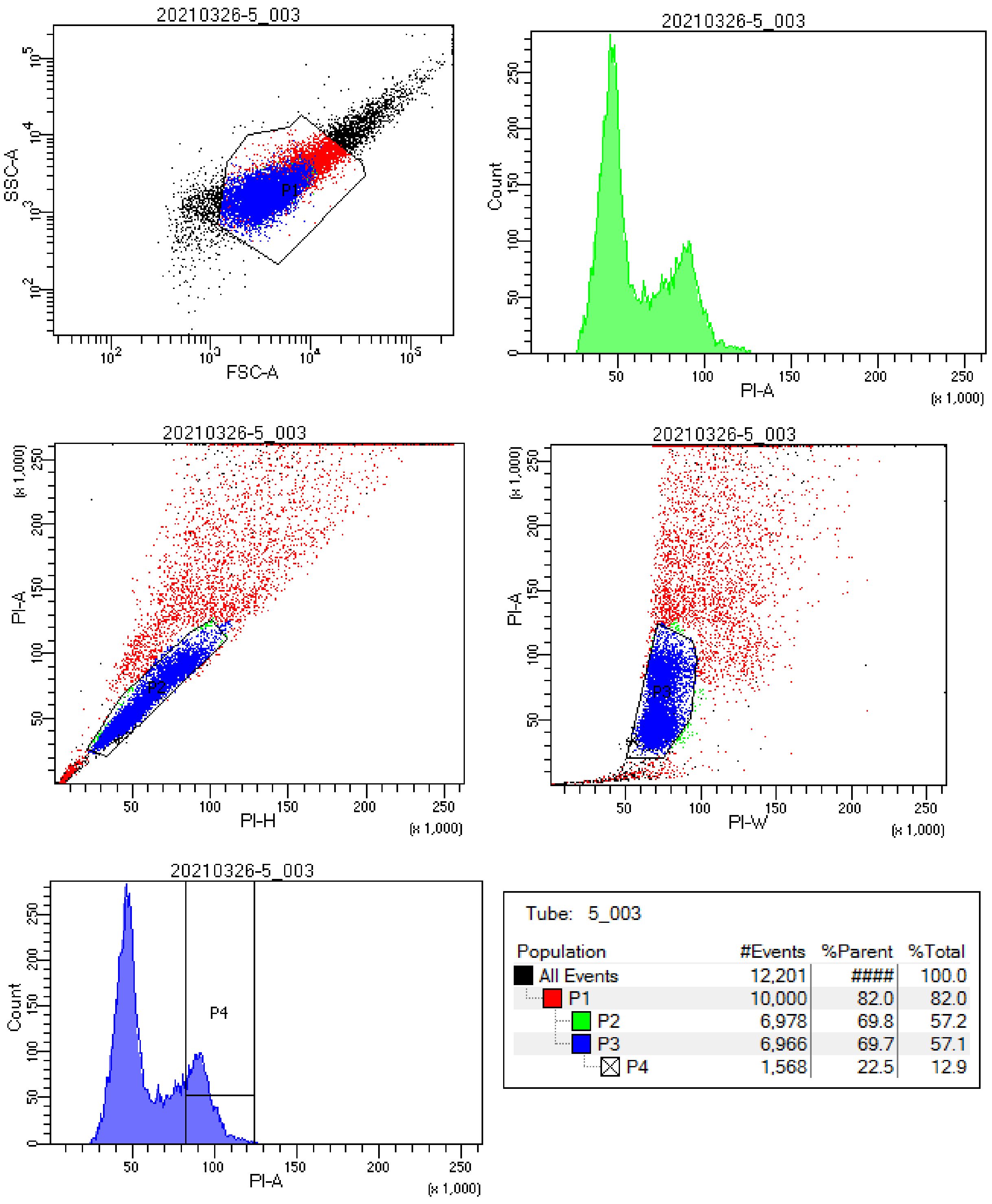

Tube: 5_003

Population	#Events	%Parent	%Total
All Events	12,201	####	100.0
P1	10,000	82.0	82.0
P2	6,978	69.8	57.2
P3	6,966	69.7	57.1
P4	1,568	22.5	12.9

BD FACSDiva 8.0.1

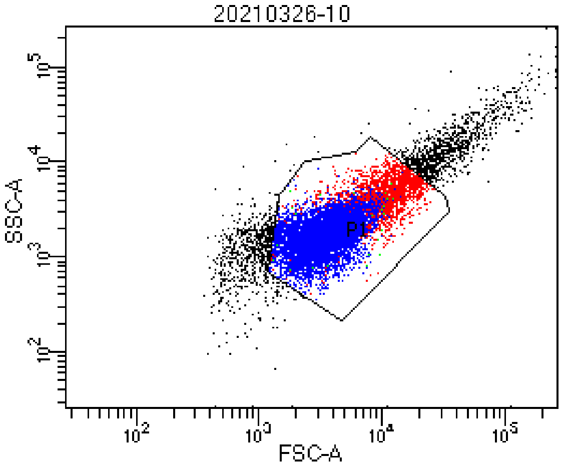

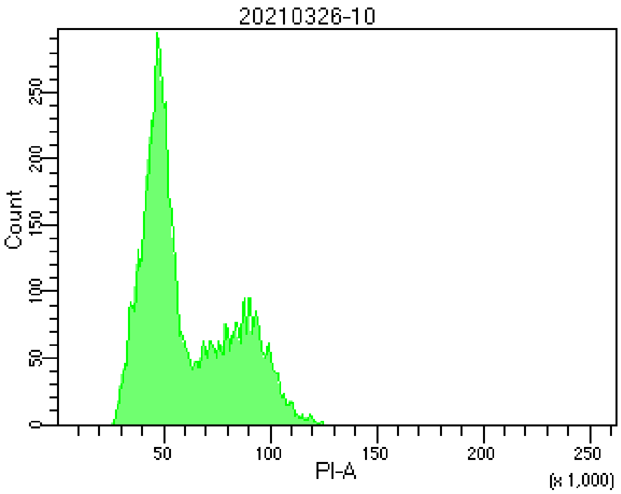

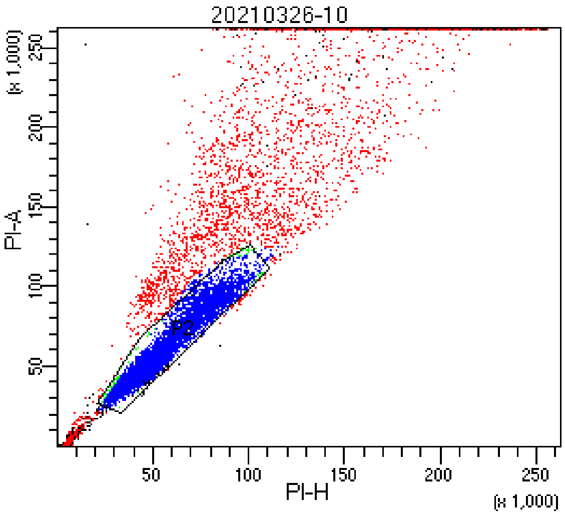

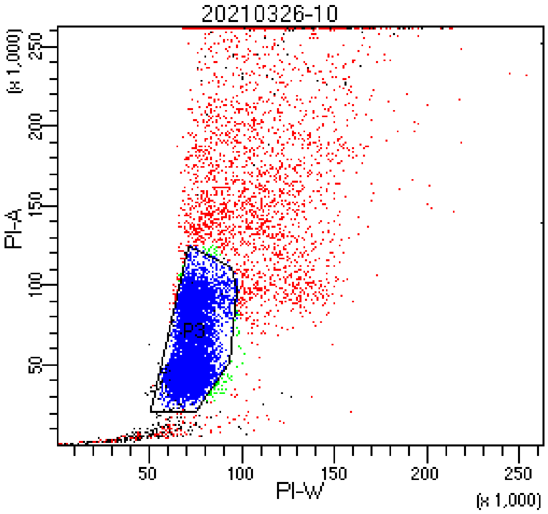

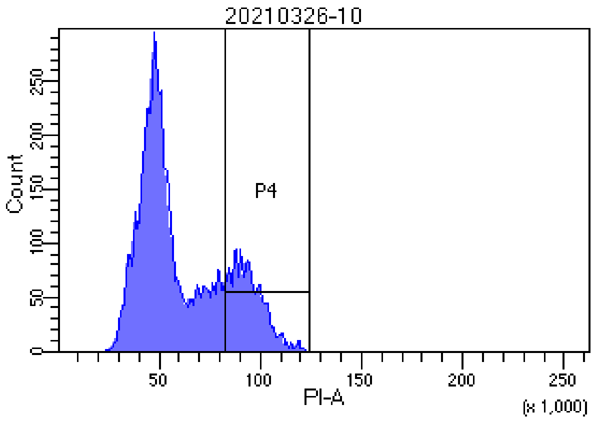

Tube: 10

Population	#Events	%Parent	%Total
All Events	11,723	####	100.0
P1	10,000	85.3	85.3
P2	7,361	73.6	62.8
P3	7,344	73.4	62.6
P4	1,603	21.8	13.7

BD FACSDiva 8.0.1

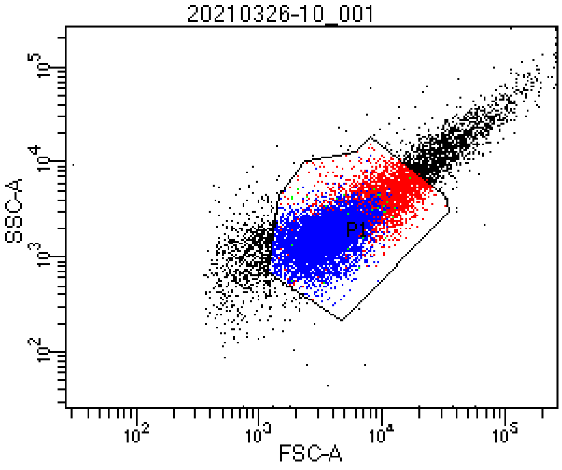

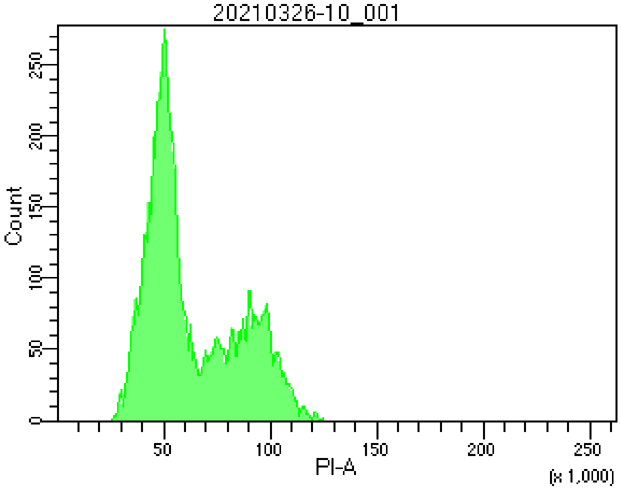

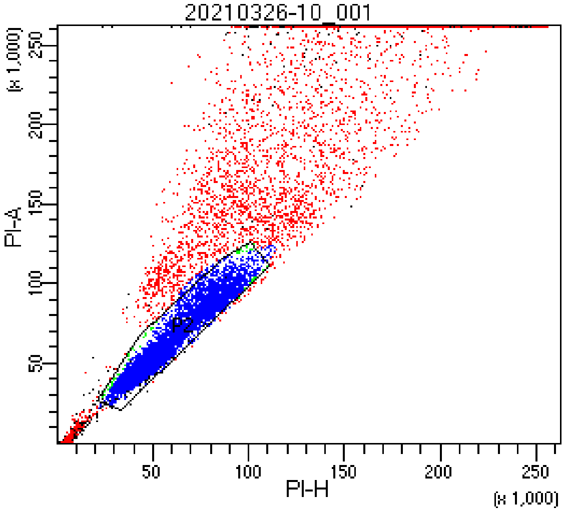

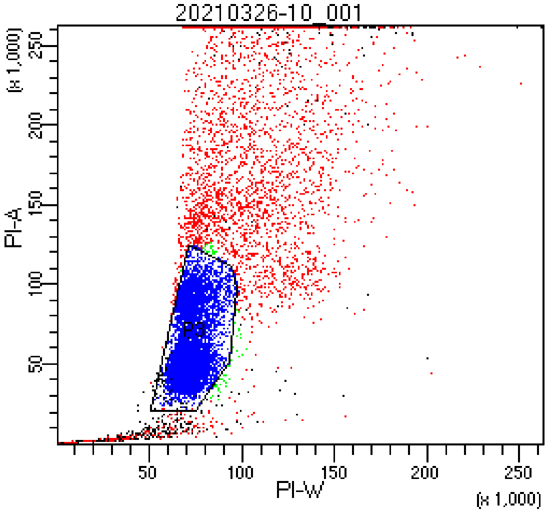

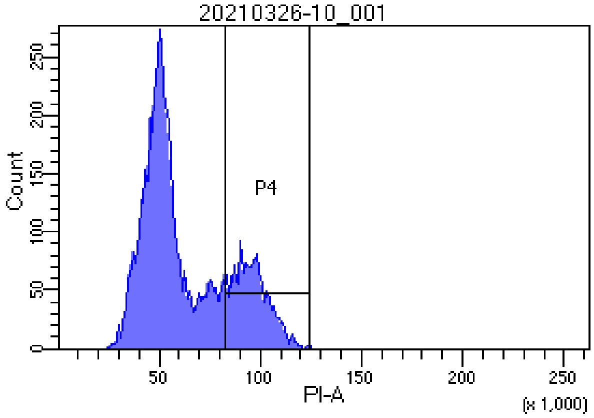

Tube: 10_001

Population	#Events	%Parent	%Total
All Events	12,400	####	100.0
P1	10,000	80.6	80.6
P2	6,872	68.7	55.4
P3	6,886	68.9	55.5
P4	1,646	23.9	13.3

BD FACSDiva 8.0.1

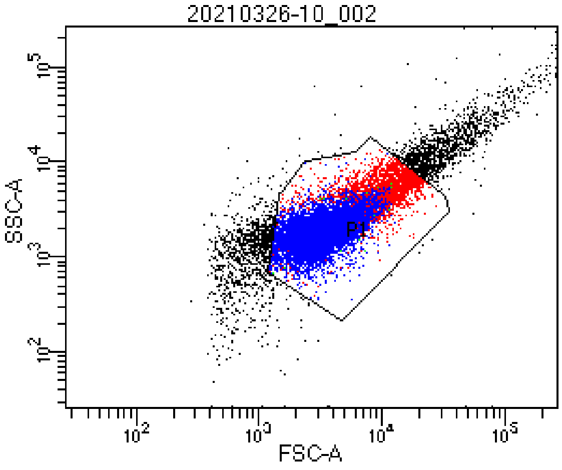

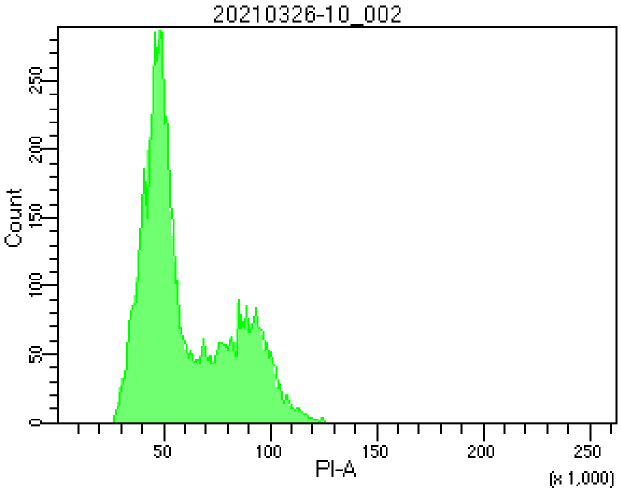

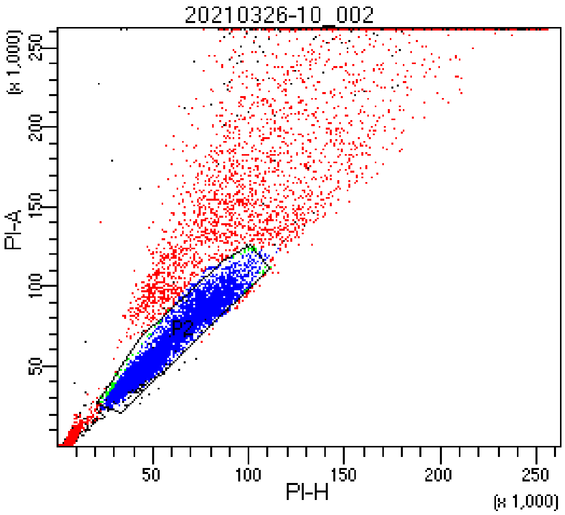

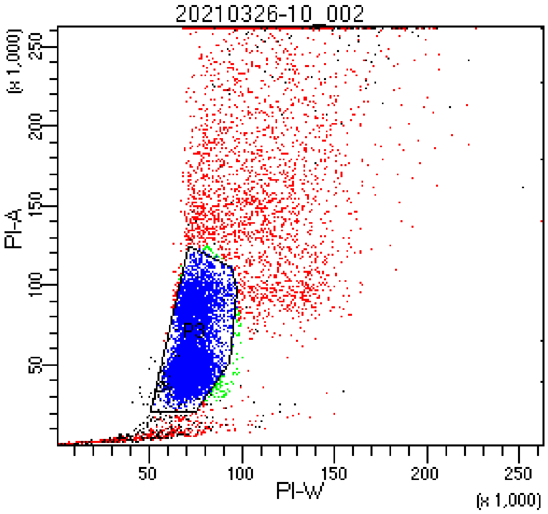

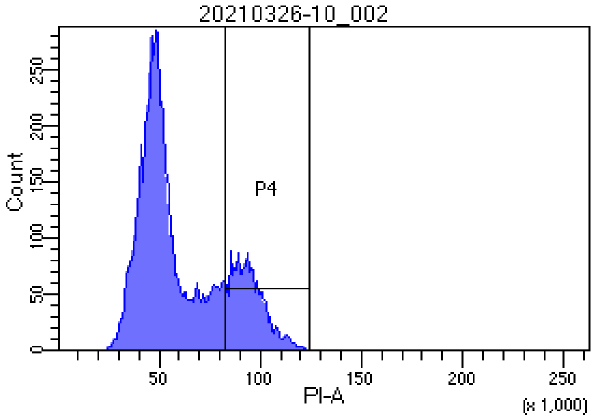

Tube: 10_002

Population	#Events	%Parent	%Total
All Events	12,269	####	100.0
P1	10,000	81.5	81.5
P2	7,186	71.9	58.6
P3	7,136	71.4	58.2
P4	1,536	21.5	12.5

BD FACSDiva 8.0.1

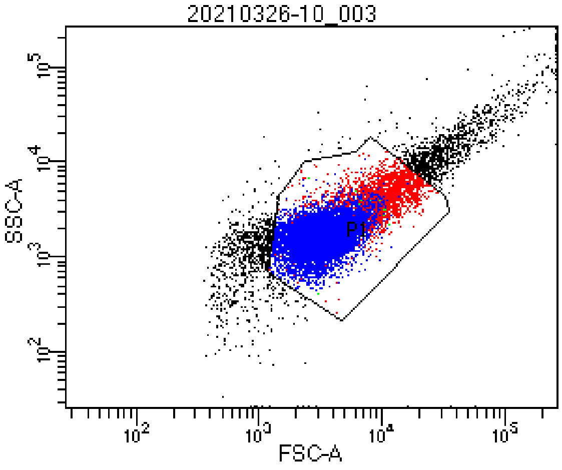

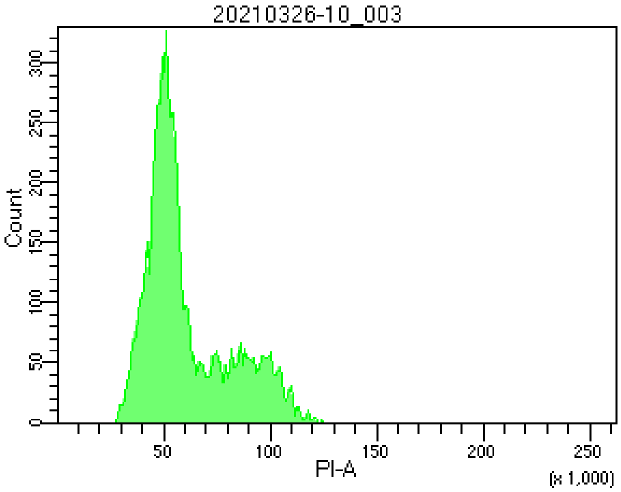

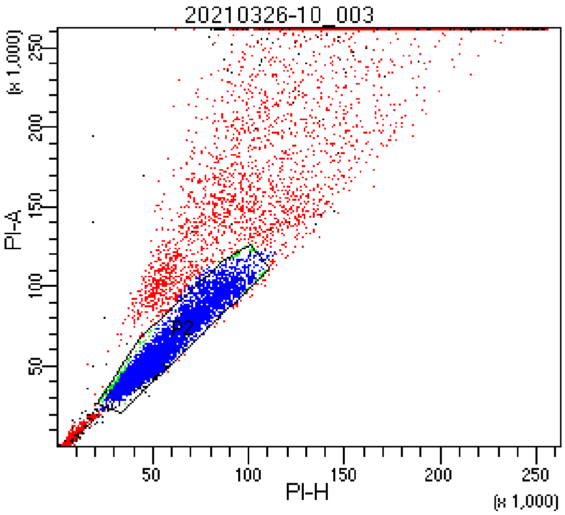

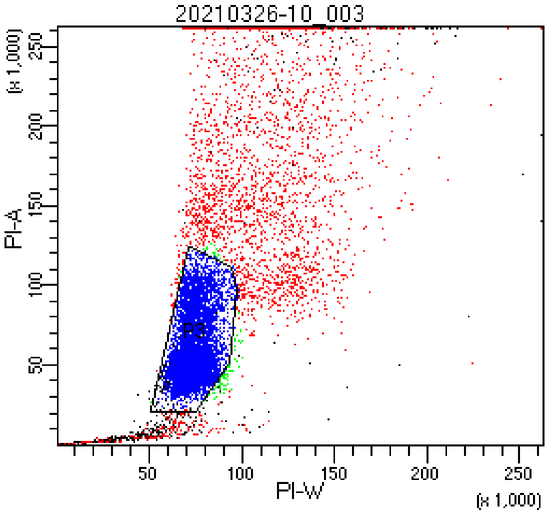

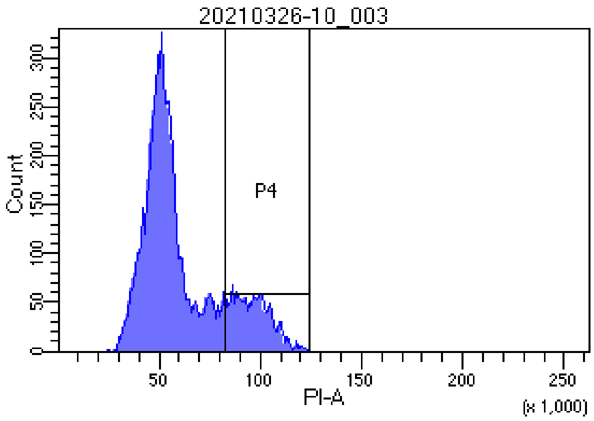

Tube: 10_003

Population	#Events	%Parent	%Total
All Events	11,982	####	100.0
P1	10,000	83.5	83.5
P2	7,389	73.9	61.7
P3	7,354	73.5	61.4
P4	1,406	19.1	11.7

BD FACSDiva 8.0.1

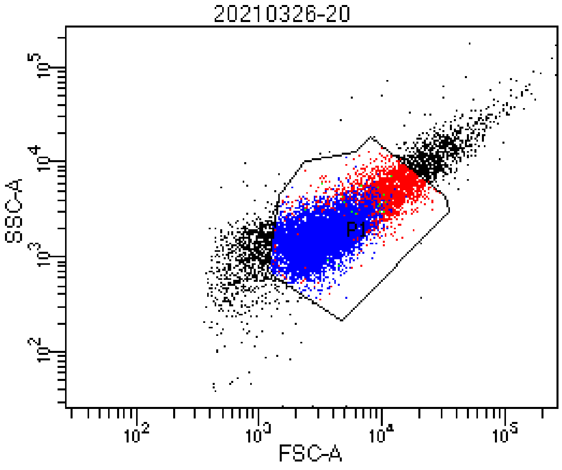

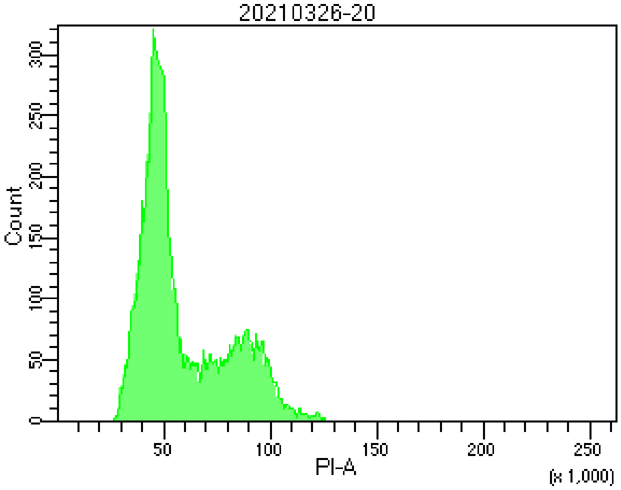

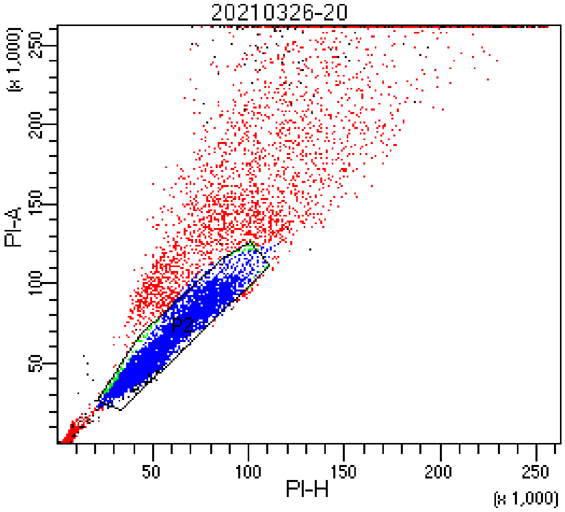

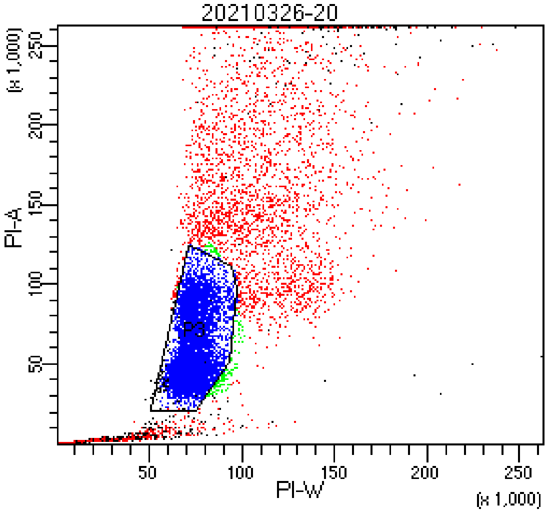

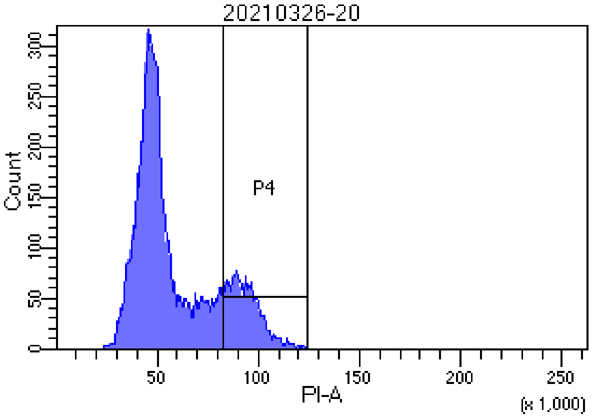

Tube: 20

Population	#Events	%Parent	%Total
All Events	11,832	####	100.0
P1	10,000	84.5	84.5
P2	7,146	71.5	60.4
P3	7,061	70.6	59.7
P4	1,344	19.0	11.4

BD FACSDiva 8.0.1

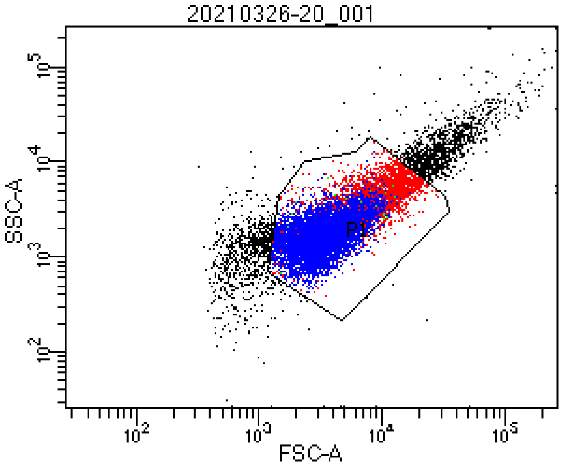

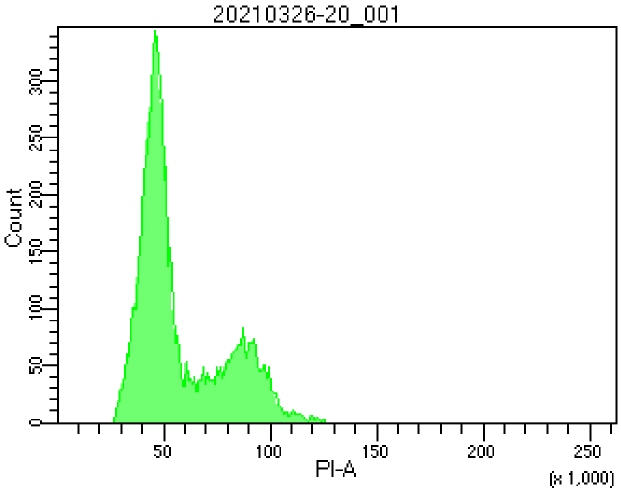

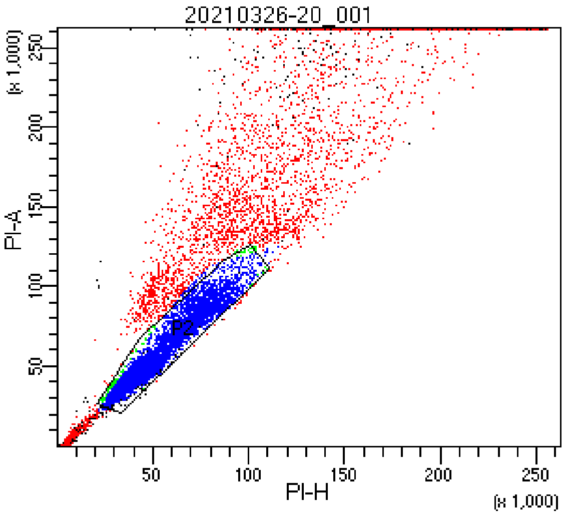

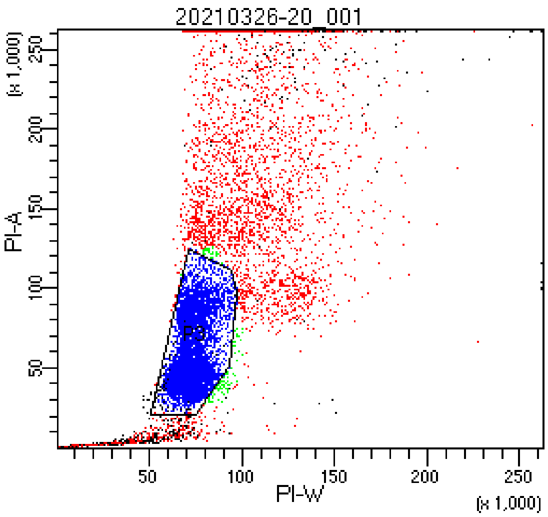

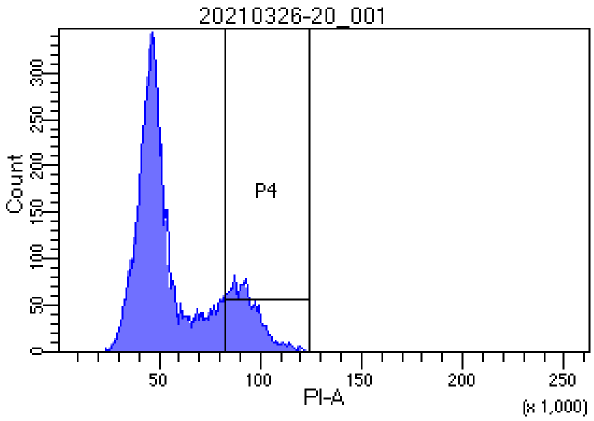

Tube: 20_001

Population	#Events	%Parent	%Total
All Events	12,162	####	100.0
P1	10,000	82.2	82.2
P2	7,120	71.2	58.5
P3	7,074	70.7	58.2
P4	1,300	18.4	10.7

BD FACSDiva 8.0.1

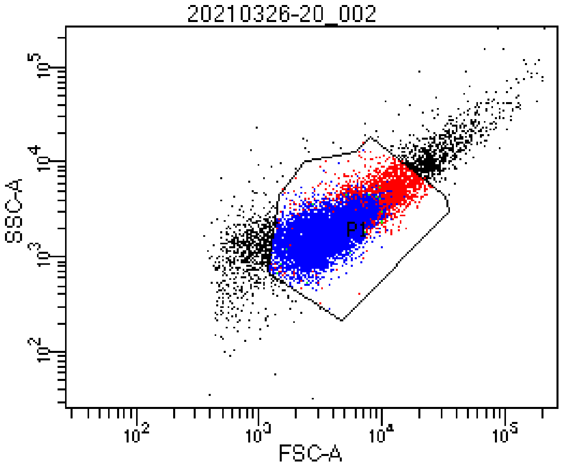

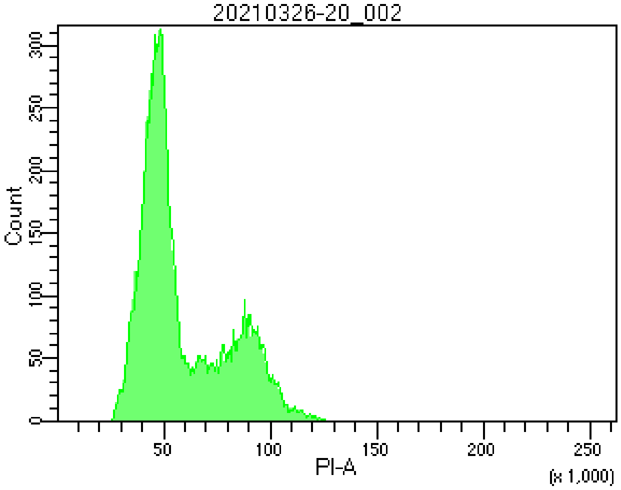

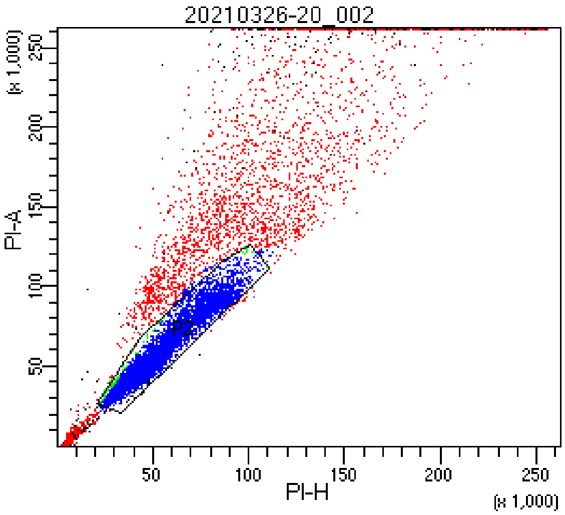

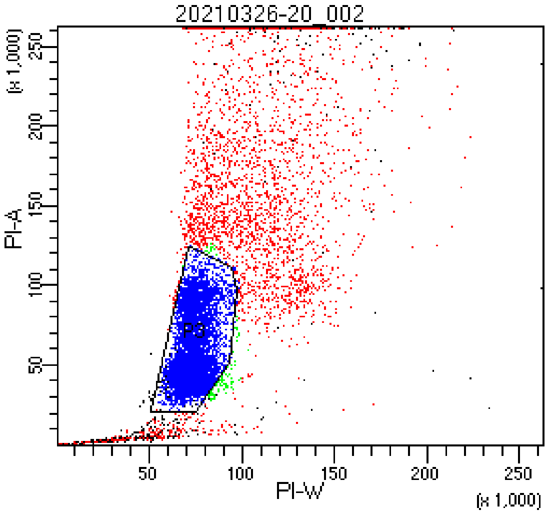

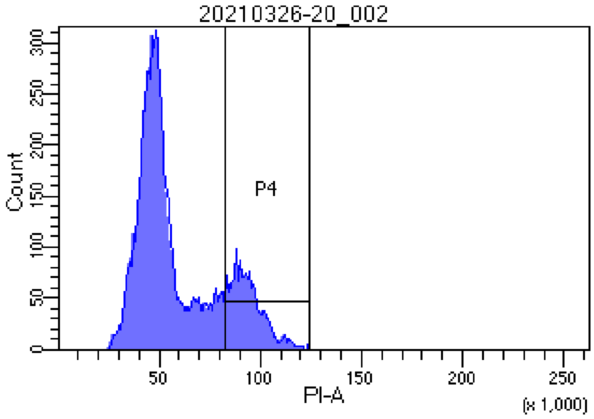

Tube: 20_002

Population	#Events	%Parent	%Total
All Events	11,650	####	100.0
P1	10,000	85.8	85.8
P2	7,369	73.7	63.3
P3	7,340	73.4	63.0
P4	1,430	19.5	12.3

BD FACSDiva 8.0.1

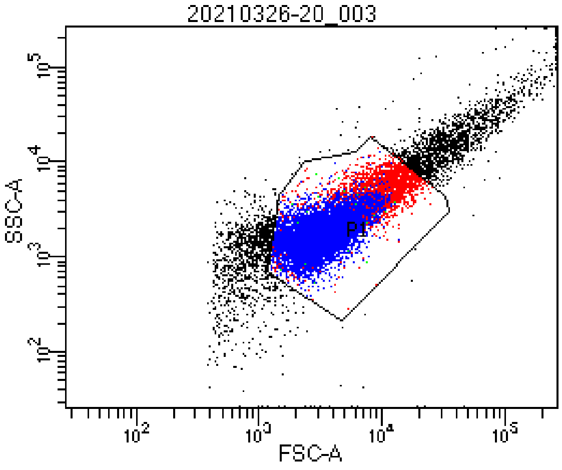

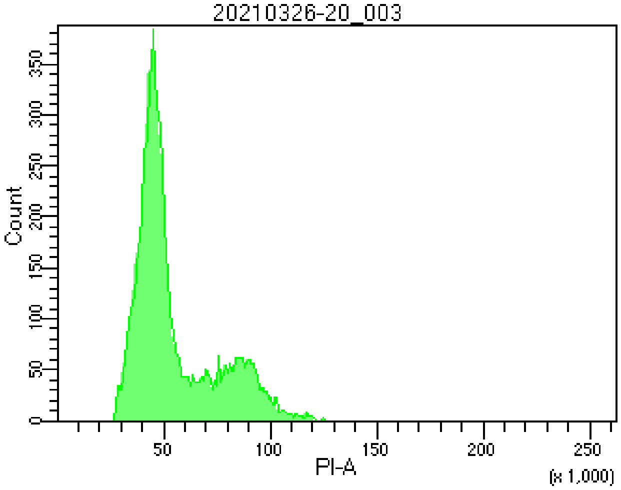

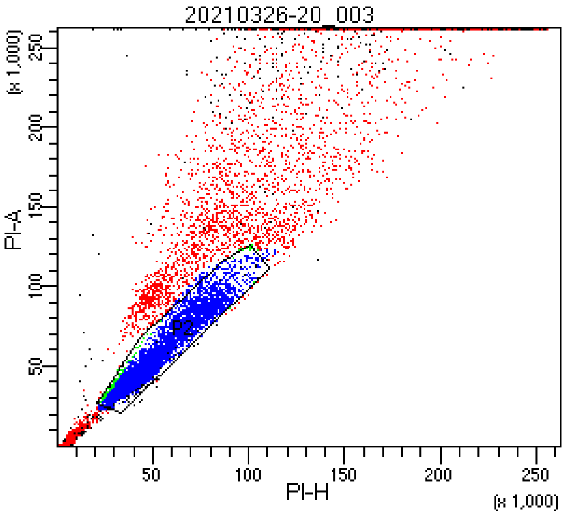

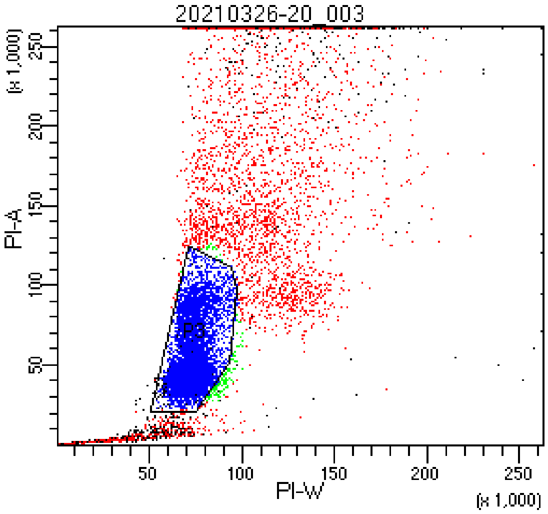

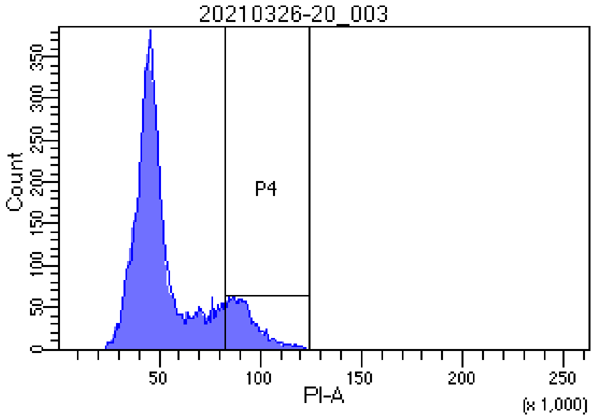

Tube: 20_003

Population	#Events	%Parent	%Total
All Events	12,759	####	100.0
P1	10,001	78.4	78.4
P2	7,261	72.6	56.9
P3	7,198	72.0	56.4
P4	1,062	14.8	8.3

BD FACSDiva 8.0.1

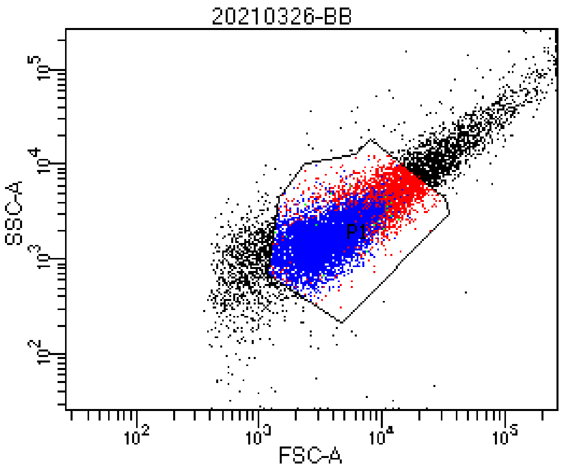

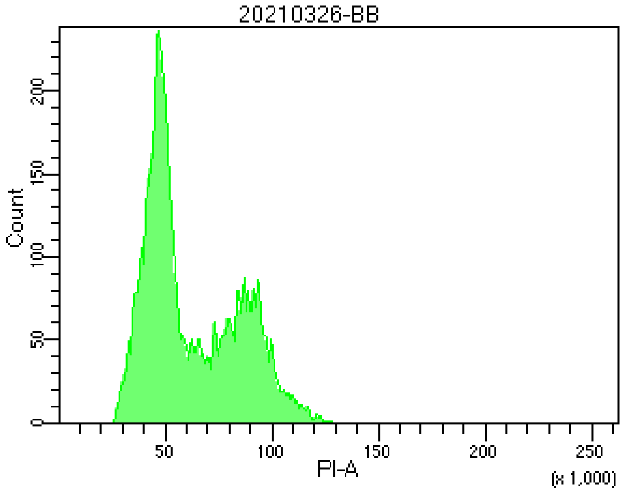

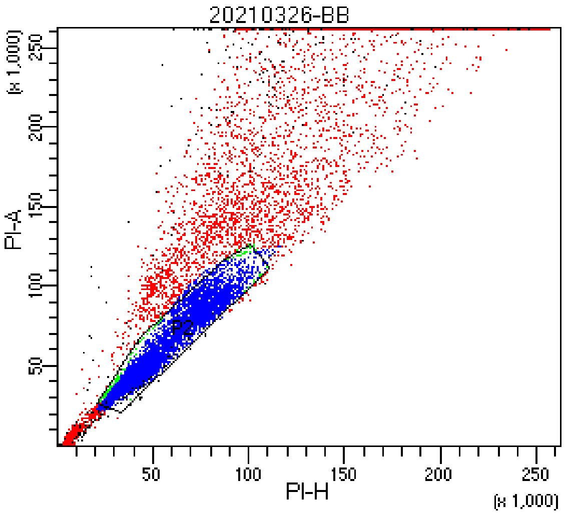

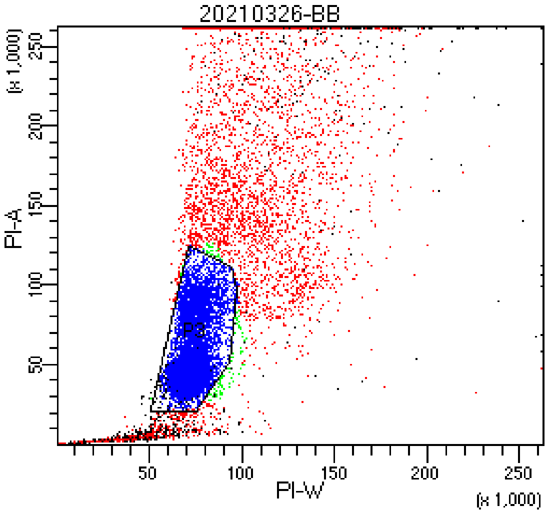

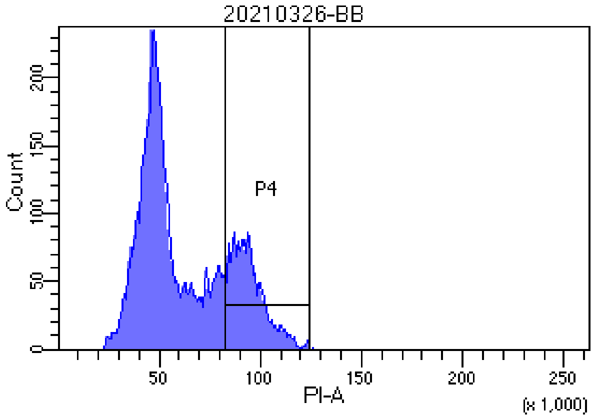

Tube: BB

Population	#Events	%Parent	%Total
All Events	12,300	####	100.0
P1	9,436	76.7	76.7
P2	6,066	64.3	49.3
P3	6,051	64.1	49.2
P4	1,504	24.9	12.2

BD FACSDiva 8.0.1

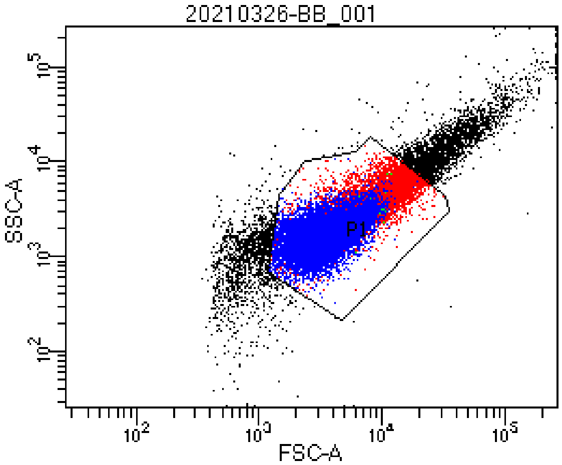

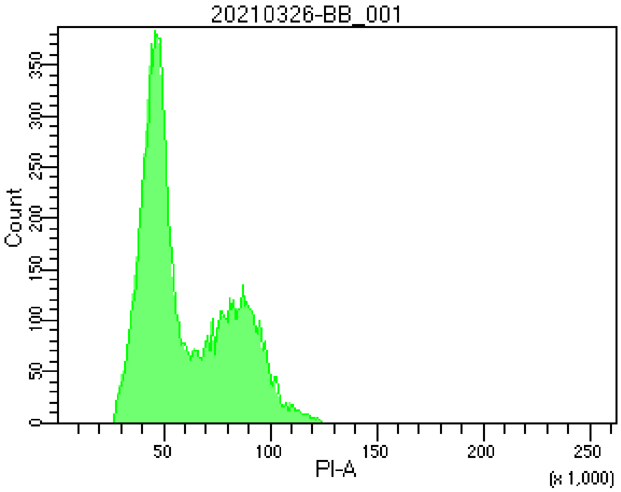

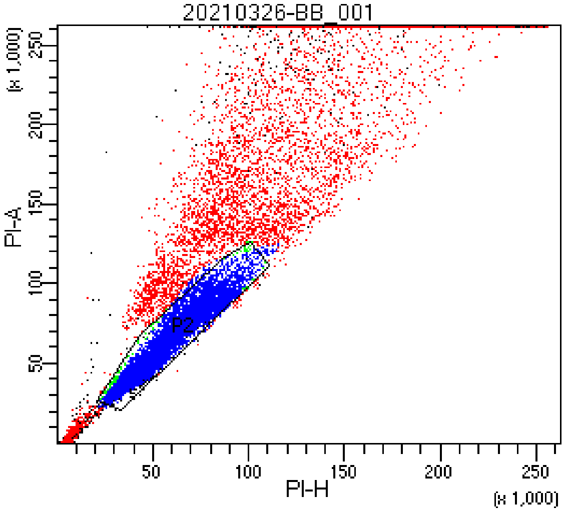

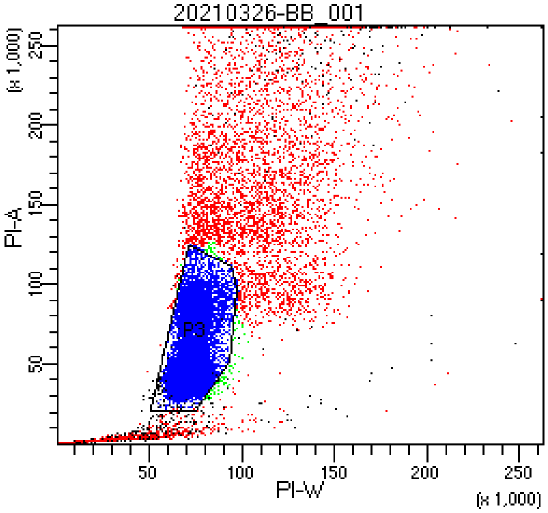

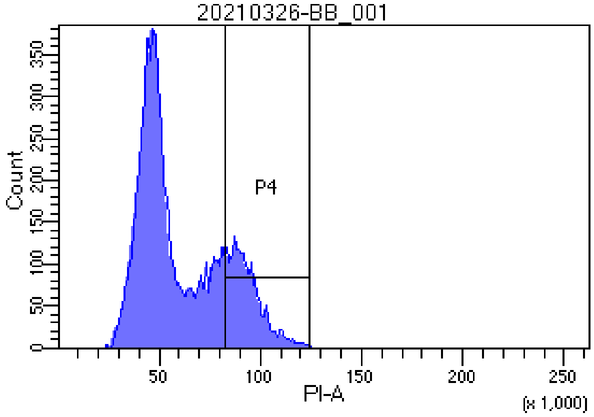

Tube: BB_001

Population	#Events	%Parent	%Total
All Events	18,594	####	100.0
P1	14,764	79.4	79.4
P2	9,988	67.7	53.7
P3	10,000	67.7	53.8
P4	2,053	20.5	11.0

BD FACSDiva 8.0.1

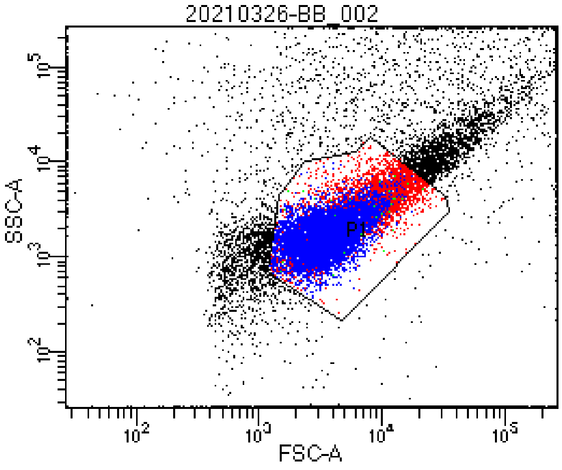

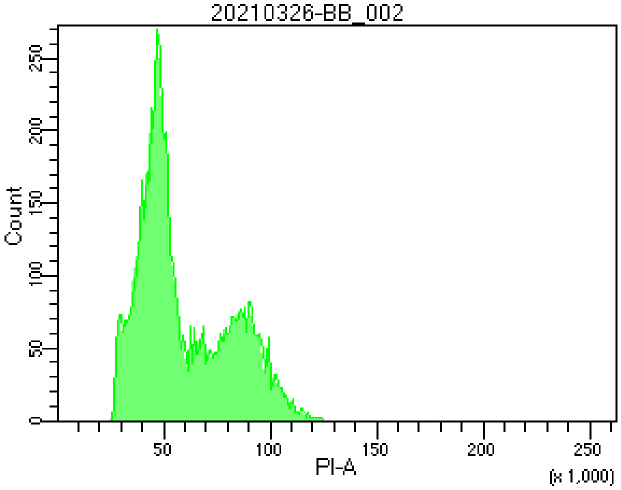

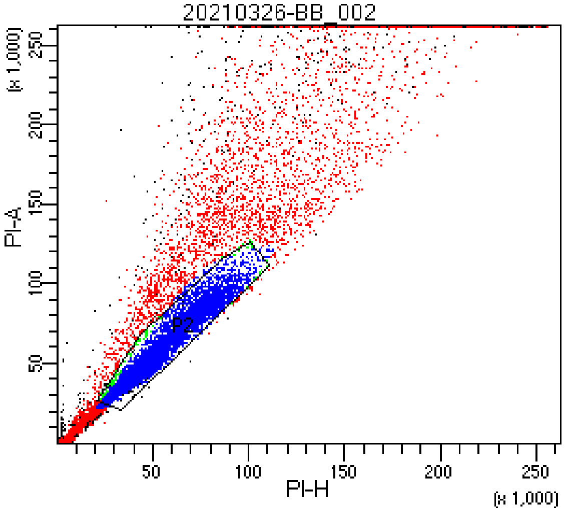

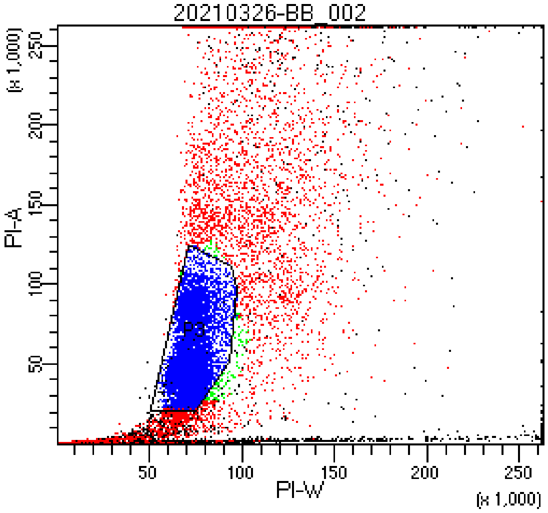

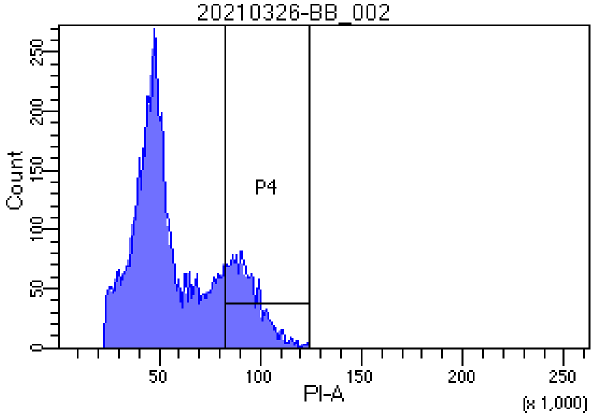

Tube: BB_002

Population	#Events	%Parent	%Total
All Events	17,491	####	100.0
P1	10,719	61.3	61.3
P2	6,829	63.7	39.0
P3	6,930	64.7	39.6
P4	1,364	19.7	7.8
