# Supplementary material for: Protein Lactylation Critically Regulates Energy Metabolism in the Protozoan Parasite Trypanosoma brucei
Source: Front Cell Dev Biol. 2021 Oct 14;9:719720. doi: 10.3389/fcell.2021.719720 (PMC8551762; doi:10.3389/fcell.2021.719720)
Supplement: Supplementary file 4 [file Data_Sheet_4.ZIP › Original Data 2-Flow Cytometry/20210330-ROS/Batch_Analysis_02042021154545.pdf]

BD FACSDiva 8.0.1

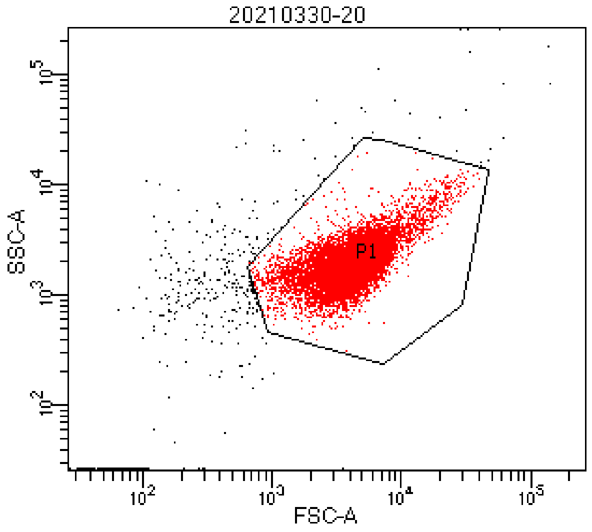


Tube: 20

| Population | #Events | %Parent | %Total |
|---|---|---|---|
| All Events | 10,817 | #### | 100.0 |
| P1 | 10,357 | 95.7 | 95.7 |

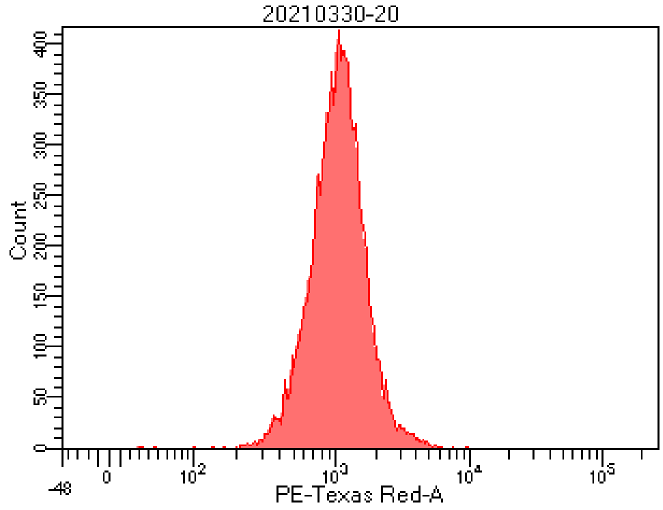


| | |
|---|---|
| Experiment Name: | Experiment-ROS |
| Specimen Name: | 20210330 |
| Tube Name: | 20 |
| Record Date: | Mar 30, 2021 4:31:39 PM |
| $OP: | Administrator |
| GUID: | df81d70d-5e6b-4646-9a25-8d6... |

| Population | #Events | %Parent | FSC-A Mean | SSC-A Mean | PE-Texa... Mean |
|---|---|---|---|---|---|
| All Events | 10,817 | #### | 4,613 | 2,345 | 1,082 |
| P1 | 10,357 | 95.7 | 4,718 | 2,186 | 1,113 |

20210330-20_001

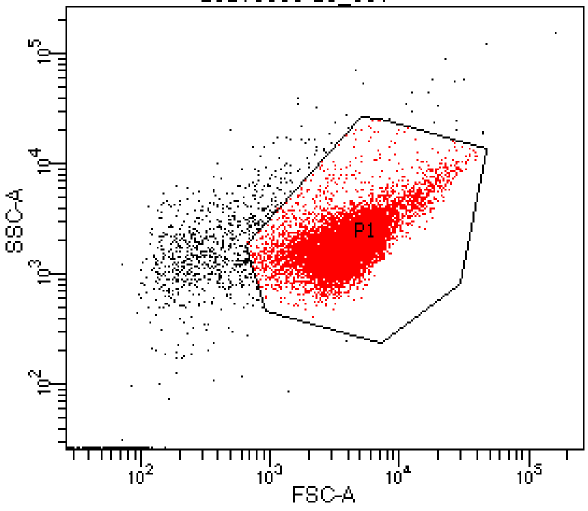


Tube: 20_001

| Population | #Events | %Parent | %Total |
|---|---|---|---|
| All Events | 11,461 | #### | 100.0 |
| P1 | 10,455 | 91.2 | 91.2 |

20210330-20_001

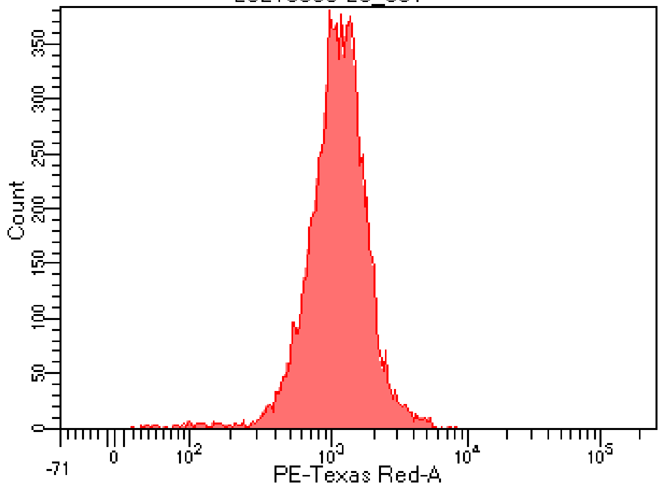


| | |
|---|---|
| Experiment Name: | Experiment-ROS |
| Specimen Name: | 20210330 |
| Tube Name: | 20_001 |
| Record Date: | Mar 30, 2021 4:34:48 PM |
| $OP: | Administrator |
| GUID: | 7fbd3b99-7793-4ec9-991c-c24... |

| Population | #Events | %Parent | FSC-A Mean | SSC-A Mean | PE-Texa... Mean |
|---|---|---|---|---|---|
| All Events | 11,461 | #### | 4,309 | 2,341 | 1,085 |
| P1 | 10,455 | 91.2 | 4,631 | 2,255 | 1,169 |

BD FACSDiva 8.0.1

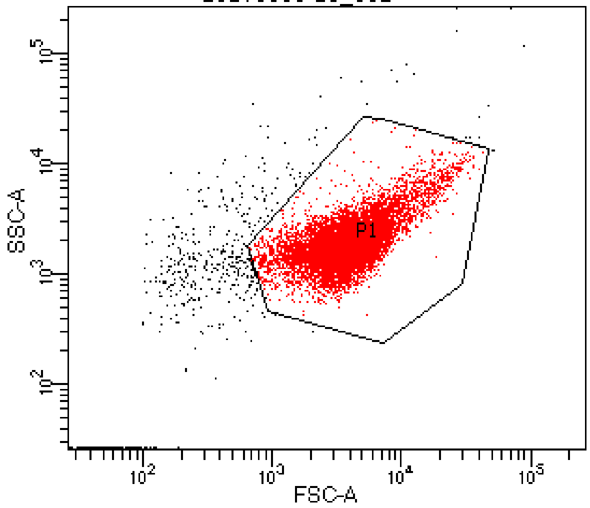


| Tube: 20_002 | | | |
|---|---|---|---|
| Population | #Events | %Parent | %Total |
| All Events | 11,071 | #### | 100.0 |
| P1 | 10,470 | 94.6 | 94.6 |

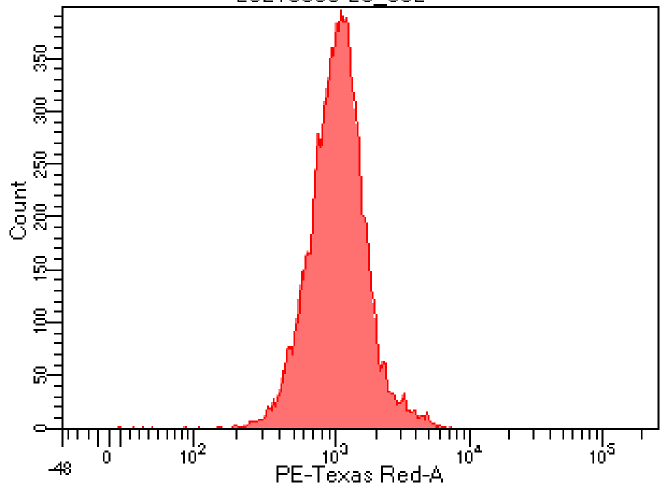


| | |
|---|---|
| Experiment Name: | Experiment-ROS |
| Specimen Name: | 20210330 |
| Tube Name: | 20_002 |
| Record Date: | Mar 30, 2021 4:38:27 PM |
| $OP: | Administrator |
| GUID: | 50a34326-b0df-48bd-95a3-57f1... |

| Population | #Events | %Parent | FSC-A Mean | SSC-A Mean | PE-Texa... Mean |
|---|---|---|---|---|---|
| All Events | 11,071 | #### | 4,624 | 2,352 | 1,078 |
| P1 | 10,470 | 94.6 | 4,807 | 2,251 | 1,118 |

## BD FACSDiva 8.0.1

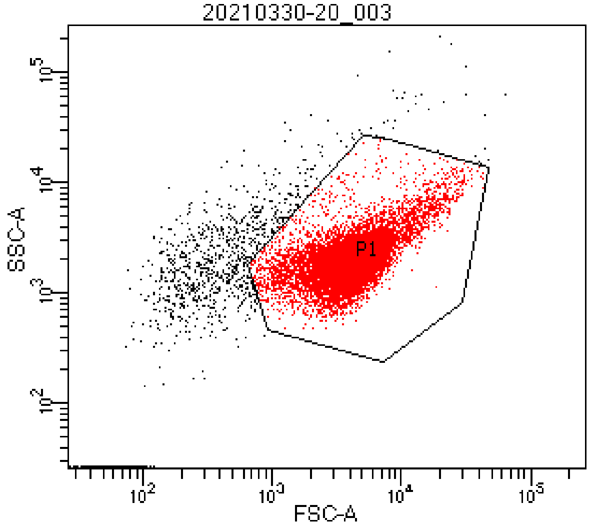


Tube: 20_003

| Population | #Events | %Parent | %Total |
|---|---|---|---|
| All Events | 11,607 | #### | 100.0 |
| P1 | 10,512 | 90.6 | 90.6 |

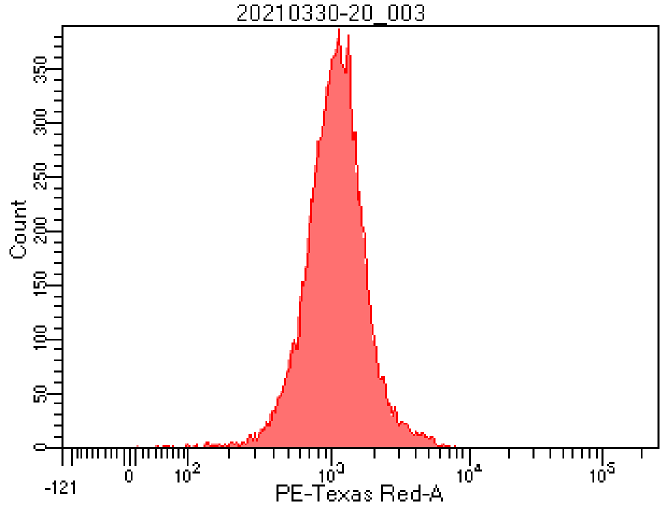


| | |
|---|---|
| Experiment Name: | Experiment-ROS |
| Specimen Name: | 20210330 |
| Tube Name: | 20_003 |
| Record Date: | Mar 30, 2021 4:42:05 PM |
| $OP: | Administrator |
| GUID: | 8f077e0b-d589-4a4c-adf5-00f8... |

| Population | #Events | %Parent | FSC-A Mean | SSC-A Mean | PE-Texa... Mean |
|---|---|---|---|---|---|
| All Events | 11,607 | #### | 4,496 | 2,529 | 1,068 |
| P1 | 10,512 | 90.6 | 4,860 | 2,351 | 1,141 |

BD FACSDiva 8.0.1

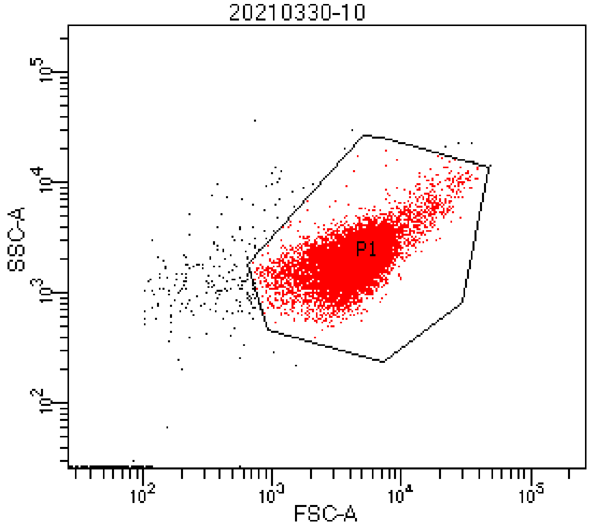


| Tube: 10 | | | |
|---|---|---|---|
| Population | #Events | %Parent | %Total |
| All Events | 10,718 | #### | 100.0 |
| P1 | 10,331 | 96.4 | 96.4 |

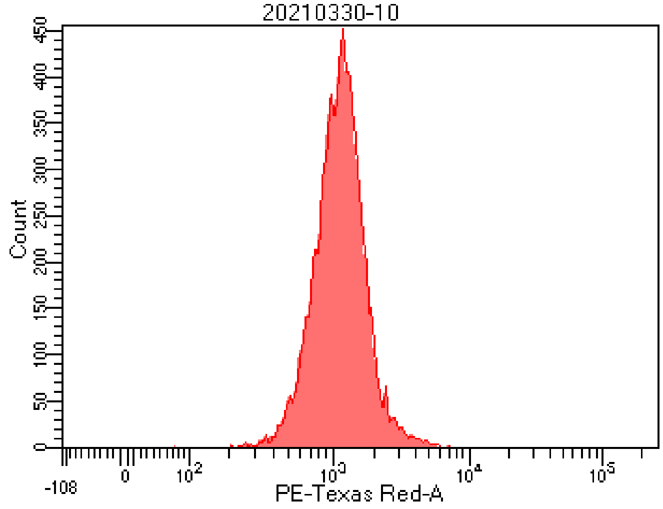


| | |
|---|---|
| Experiment Name: | Experiment-ROS |
| Specimen Name: | 20210330 |
| Tube Name: | 10 |
| Record Date: | Mar 30, 2021 4:45:17 PM |
| $OP: | Administrator |
| GUID: | 0715640b-b33e-40cb-aa24-f54... |

| Population | #Events | %Parent | FSC-A Mean | SSC-A Mean | PE-Texa... Mean |
|---|---|---|---|---|---|
| All Events | 10,718 | #### | 4,750 | 2,198 | 1,135 |
| P1 | 10,331 | 96.4 | 4,896 | 2,217 | 1,169 |

BD FACSDiva 8.0.1

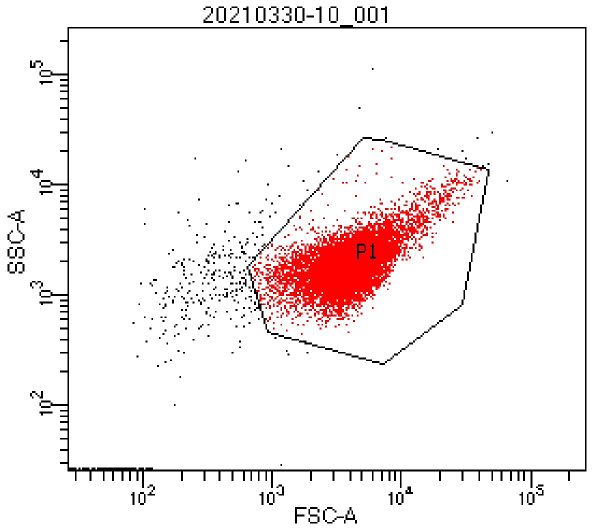


Tube: 10_001

| Population | #Events | %Parent | %Total |
|---|---|---|---|
| All Events | 10,911 | #### | 100.0 |
| P1 | 10,401 | 95.3 | 95.3 |

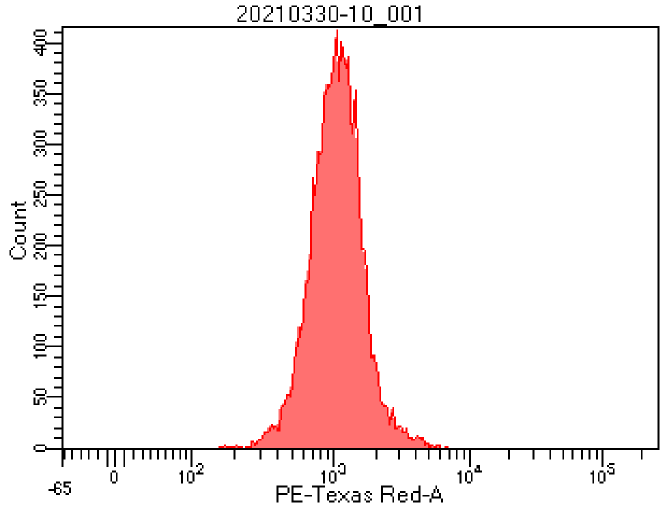


| | |
|---|---|
| Experiment Name: | Experiment-ROS |
| Specimen Name: | 20210330 |
| Tube Name: | 10_001 |
| Record Date: | Mar 30, 2021 4:49:07 PM |
| $OP: | Administrator |
| GUID: | 963ac5b5-d8de-417d-927b-948... |

| Population | #Events | %Parent | FSC-A Mean | SSC-A Mean | PE-Texa... Mean |
|---|---|---|---|---|---|
| All Events | 10,911 | #### | 4,619 | 2,203 | 1,049 |
| P1 | 10,401 | 95.3 | 4,794 | 2,213 | 1,090 |

BD FACSDiva 8.0.1

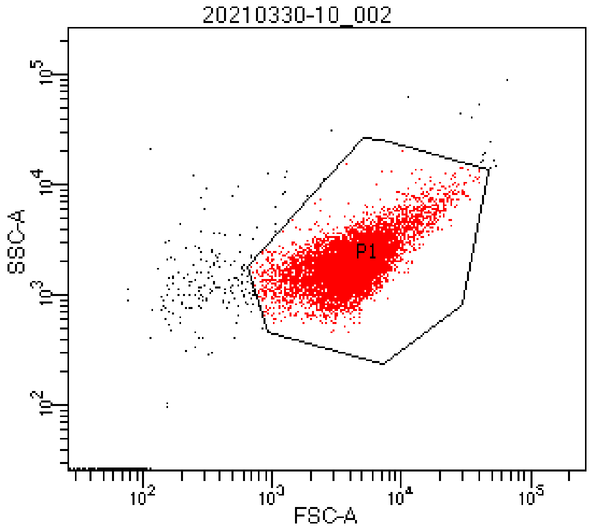


| Tube: 10_002 | | | |
|---|---|---|---|
| Population | #Events | %Parent | %Total |
| All Events | 10,647 | #### | 100.0 |
| P1 | 10,338 | 97.1 | 97.1 |

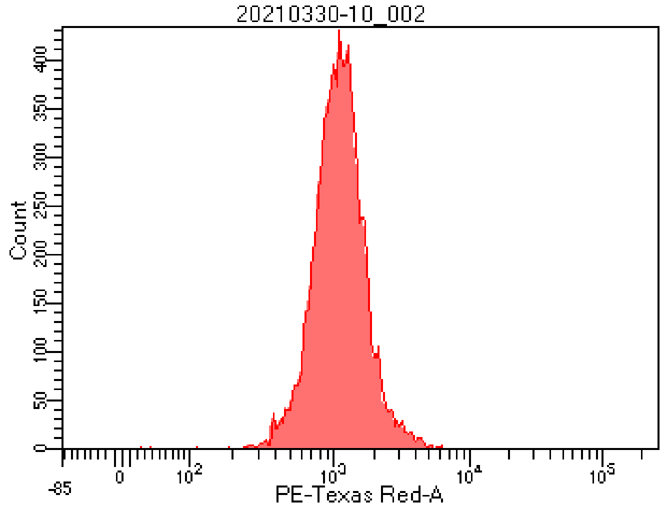


| | |
|---|---|
| Experiment Name: | Experiment-ROS |
| Specimen Name: | 20210330 |
| Tube Name: | 10_002 |
| Record Date: | Mar 30, 2021 4:51:18 PM |
| $OP: | Administrator |
| GUID: | 8a501462-9141-430e-89a4-47f... |

| Population | #Events | %Parent | FSC-A Mean | SSC-A Mean | PE-Texa... Mean |
|---|---|---|---|---|---|
| All Events | 10,647 | #### | 4,840 | 2,220 | 1,124 |
| P1 | 10,338 | 97.1 | 4,922 | 2,203 | 1,148 |

BD FACSDiva 8.0.1

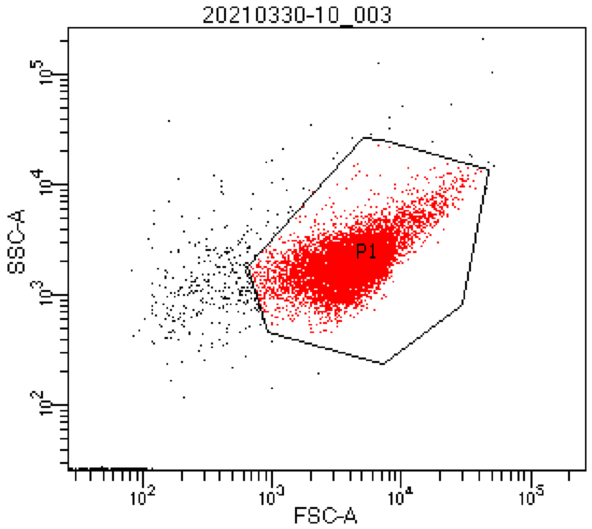


| Tube: 10_003 | | | |
|---|---|---|---|
| Population | #Events | %Parent | %Total |
| All Events | 9,734 | #### | 100.0 |
| P1 | 9,198 | 94.5 | 94.5 |

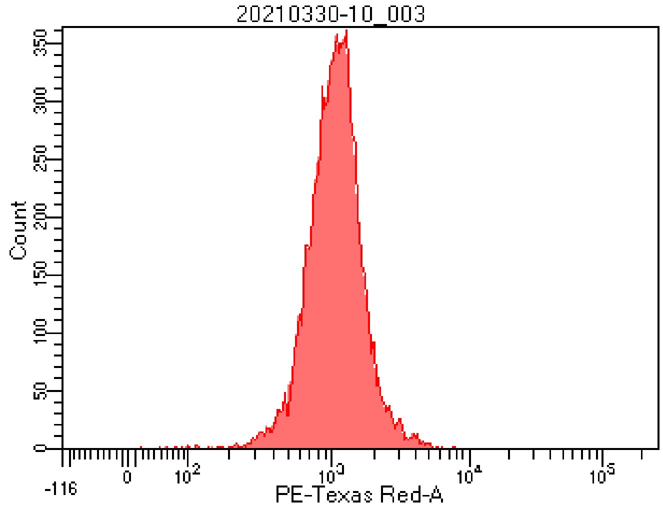


| | |
|---|---|
| Experiment Name: | Experiment-ROS |
| Specimen Name: | 20210330 |
| Tube Name: | 10_003 |
| Record Date: | Mar 30, 2021 4:55:30 PM |
| $OP: | Administrator |
| GUID: | 91b6b0c3-fe2f-451d-8a1a-3e9c... |

| Population | #Events | %Parent | FSC-A Mean | SSC-A Mean | PE-Texa... Mean |
|---|---|---|---|---|---|
| All Events | 9,734 | #### | 4,628 | 2,302 | 1,052 |
| P1 | 9,198 | 94.5 | 4,832 | 2,264 | 1,099 |

BD FACSDiva 8.0.1

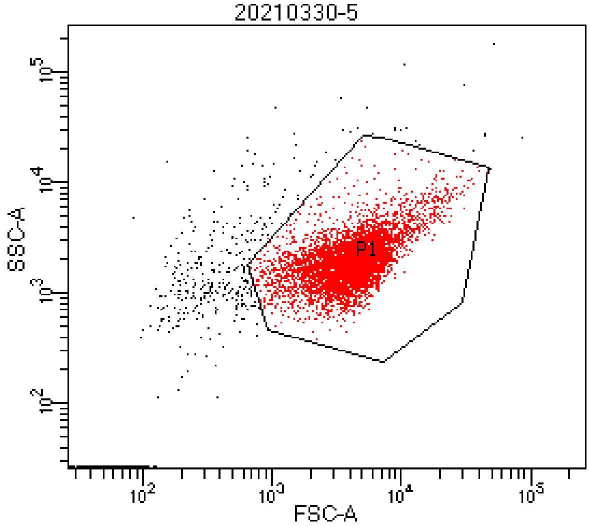


Tube: 5

| Population | #Events | %Parent | %Total |
|---|---|---|---|
| All Events | 6,411 | #### | 100.0 |
| P1 | 5,850 | 91.2 | 91.2 |

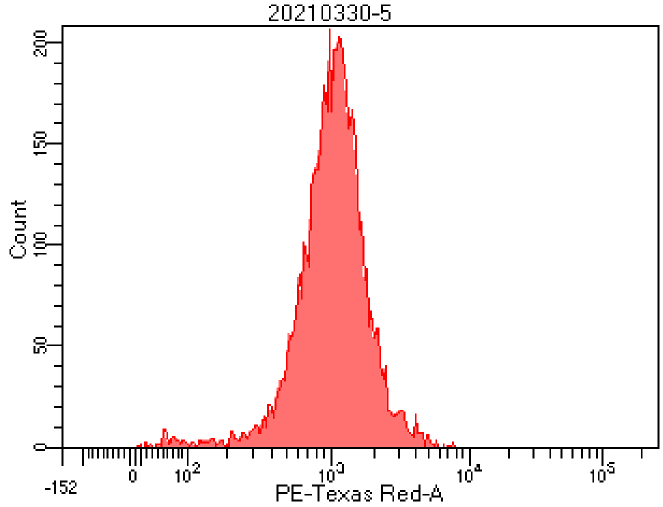


| | |
|---|---|
| Experiment Name: | Experiment-ROS |
| Specimen Name: | 20210330 |
| Tube Name: | 5 |
| Record Date: | Mar 30, 2021 4:59:15 PM |
| $OP: | Administrator |
| GUID: | 5b17a68c-cd38-4ef0-b177-fcbfc... |

| Population | #Events | %Parent | FSC-A Mean | SSC-A Mean | PE-Texa... Mean |
|---|---|---|---|---|---|
| All Events | 6,411 | #### | 4,473 | 2,381 | 1,048 |
| P1 | 5,850 | 91.2 | 4,794 | 2,281 | 1,120 |

BD FACSDiva 8.0.1

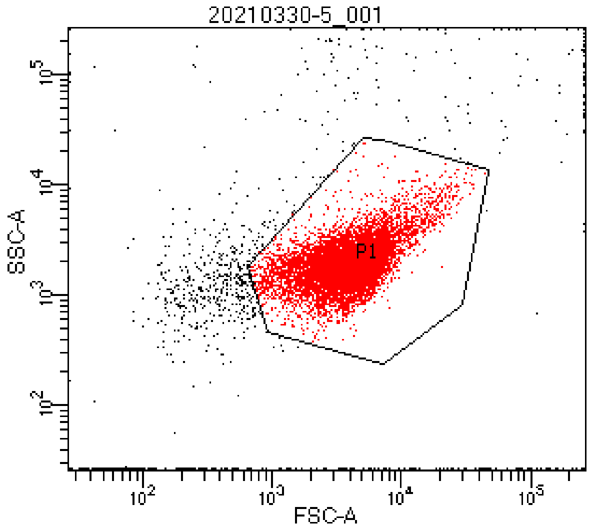


Tube: 5_001

| Population | #Events | %Parent | %Total |
|---|---|---|---|
| All Events | 10,375 | #### | 100.0 |
| P1 | 9,217 | 88.8 | 88.8 |

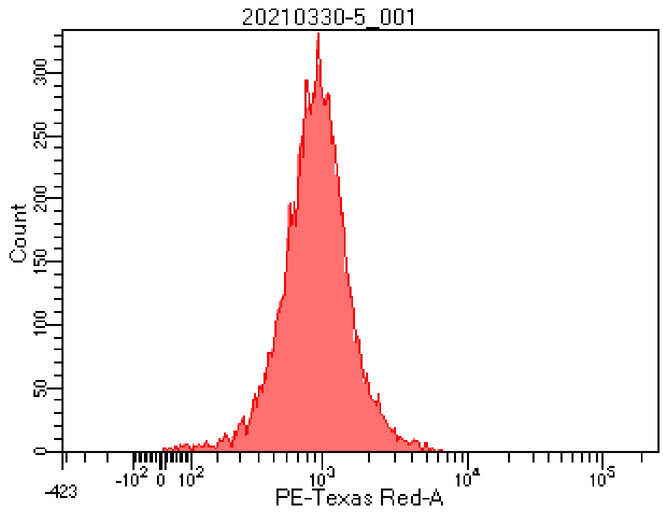


| | |
|---|---|
| Experiment Name: | Experiment-ROS |
| Specimen Name: | 20210330 |
| Tube Name: | 5_001 |
| Record Date: | Mar 30, 2021 5:02:40 PM |
| $OP: | Administrator |
| GUID: | 040ec225-18ec-4b53-9e00-0f4... |

| Population | #Events | %Parent | FSC-A Mean | SSC-A Mean | PE-Texa... Mean |
|---|---|---|---|---|---|
| All Events | 10,375 | #### | 9,399 | 6,700 | 903 |
| P1 | 9,217 | 88.8 | 4,672 | 2,269 | 987 |

BD FACSDiva 8.0.1

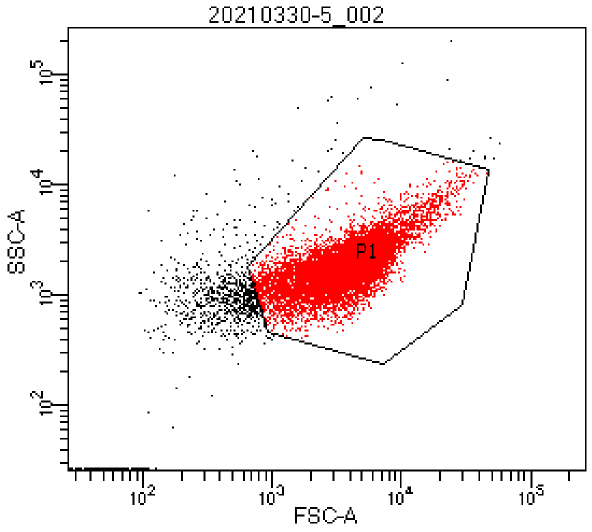


Tube: 5_002

| Population | #Events | %Parent | %Total |
|---|---|---|---|
| All Events | 12,094 | #### | 100.0 |
| P1 | 11,020 | 91.1 | 91.1 |

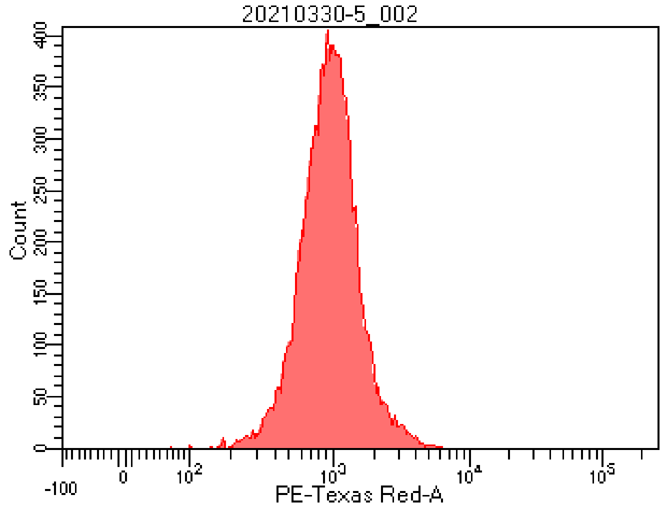


| | |
|---|---|
| Experiment Name: | Experiment-ROS |
| Specimen Name: | 20210330 |
| Tube Name: | 5_002 |
| Record Date: | Mar 30, 2021 5:06:27 PM |
| $OP: | Administrator |
| GUID: | 7b9fa2c5-c53f-417d-8fb9-5f734... |

| Population | #Events | %Parent | FSC-A Mean | SSC-A Mean | PE-Texa... Mean |
|---|---|---|---|---|---|
| All Events | 12,094 | #### | 4,223 | 2,118 | 961 |
| P1 | 11,020 | 91.1 | 4,550 | 2,117 | 1,011 |

BD FACSDiva 8.0.1

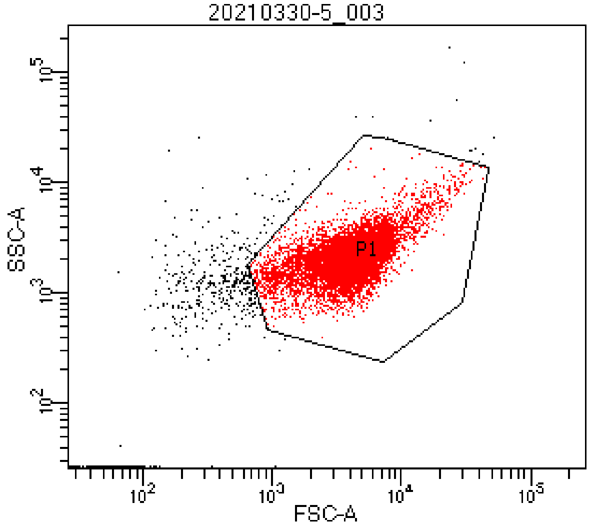


| Tube: 5_003 | | | |
|---|---|---|---|
| Population | #Events | %Parent | %Total |
| All Events | 9,002 | #### | 100.0 |
| P1 | 8,368 | 93.0 | 93.0 |

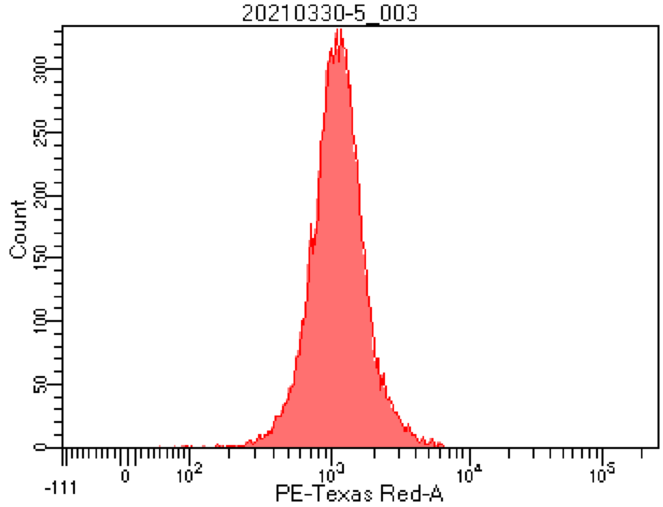


| | |
|---|---|
| Experiment Name: | Experiment-ROS |
| Specimen Name: | 20210330 |
| Tube Name: | 5_003 |
| Record Date: | Mar 30, 2021 5:10:18 PM |
| $OP: | Administrator |
| GUID: | 10eafb5e-7e1e-4ded-a9eb-667... |

| Population | #Events | %Parent | FSC-A Mean | SSC-A Mean | PE-Texa... Mean |
|---|---|---|---|---|---|
| All Events | 9,002 | #### | 4,443 | 2,355 | 1,096 |
| P1 | 8,368 | 93.0 | 4,713 | 2,371 | 1,151 |

# BD FACSDiva 8.0.1

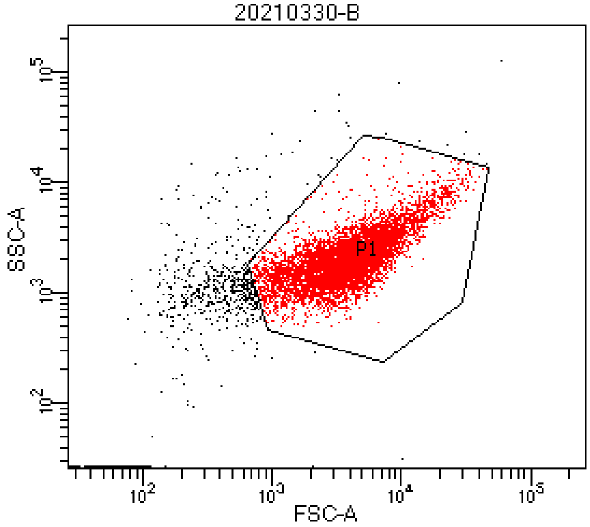


| Tube: B | | | |
|---|---|---|---|
| Population | #Events | %Parent | %Total |
| All Events | 8,708 | #### | 100.0 |
| P1 | 7,920 | 91.0 | 91.0 |

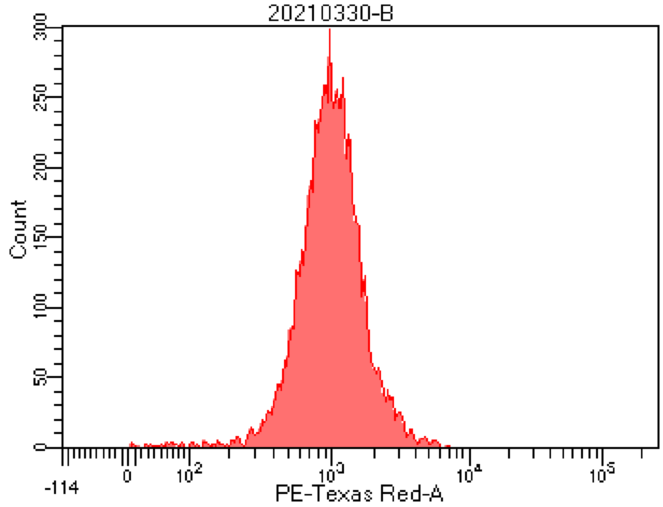


| | |
|---|---|
| Experiment Name: | Experiment-ROS |
| Specimen Name: | 20210330 |
| Tube Name: | B |
| Record Date: | Mar 30, 2021 5:13:49 PM |
| $OP: | Administrator |
| GUID: | 566f9a33-7e88-445f-924f-007f0... |

| Population | #Events | %Parent | FSC-A Mean | SSC-A Mean | PE-Texa... Mean |
|---|---|---|---|---|---|
| All Events | 8,708 | #### | 4,655 | 2,398 | 983 |
| P1 | 7,920 | 91.0 | 5,046 | 2,418 | 1,055 |

BD FACSDiva 8.0.1

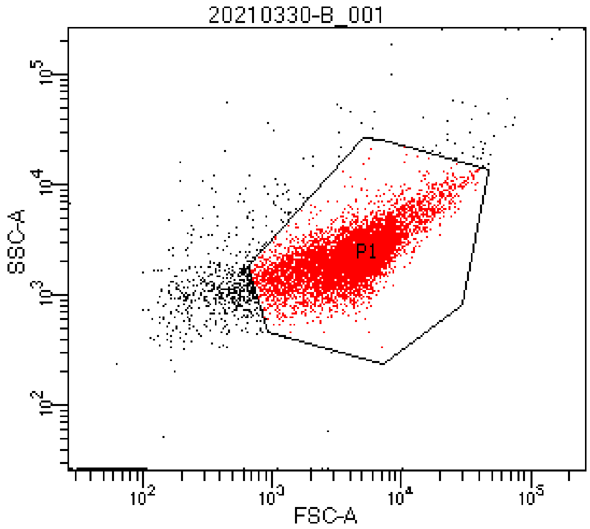


Tube: B_001

| Population | #Events | %Parent | %Total |
|---|---|---|---|
| All Events | 8,650 | #### | 100.0 |
| P1 | 7,781 | 90.0 | 90.0 |

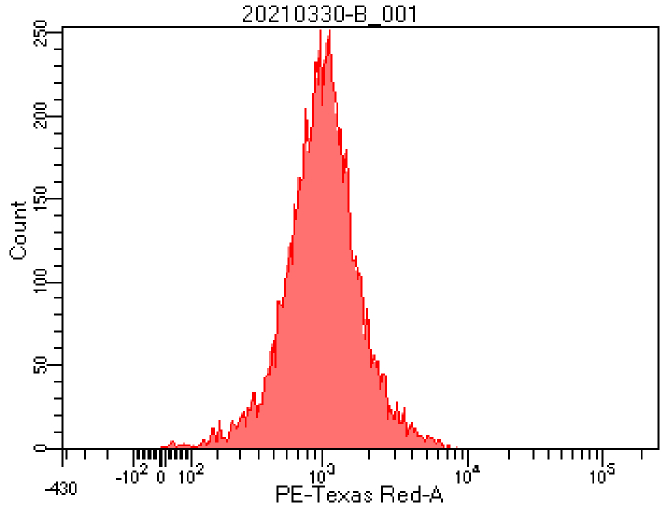


| | |
|---|---|
| Experiment Name: | Experiment-ROS |
| Specimen Name: | 20210330 |
| Tube Name: | B_001 |
| Record Date: | Mar 30, 2021 5:17:11 PM |
| $OP: | Administrator |
| GUID: | b10fecea-b6c4-43fa-aeb5-7952... |

| Population | #Events | %Parent | FSC-A Mean | SSC-A Mean | PE-Texa... Mean |
|---|---|---|---|---|---|
| All Events | 8,650 | #### | 4,885 | 2,877 | 1,102 |
| P1 | 7,781 | 90.0 | 5,118 | 2,627 | 1,118 |

BD FACSDiva 8.0.1

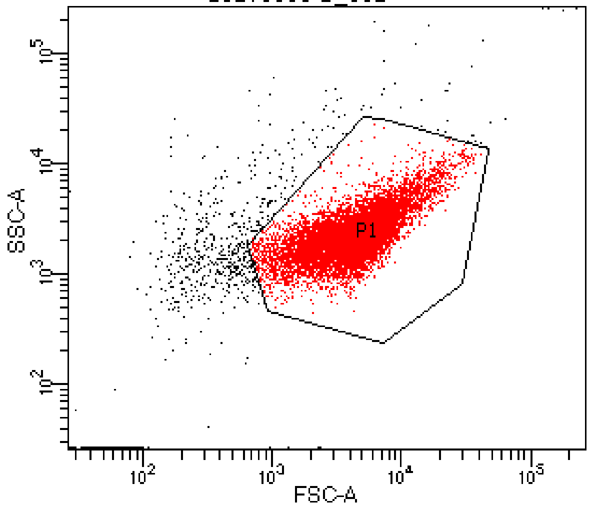


| Tube: B_002 | | | |
|---|---|---|---|
| Population | #Events | %Parent | %Total |
| All Events | 11,470 | #### | 100.0 |
| P1 | 10,657 | 92.9 | 92.9 |

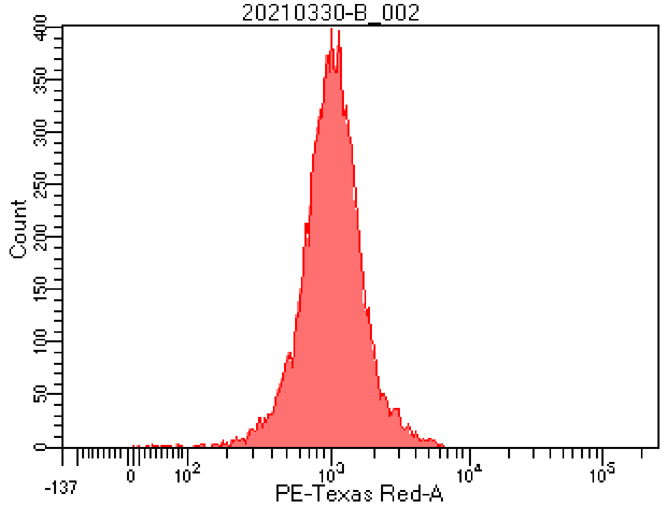


| | |
|---|---|
| Experiment Name: | Experiment-ROS |
| Specimen Name: | 20210330 |
| Tube Name: | B_002 |
| Record Date: | Mar 30, 2021 5:20:03 PM |
| $OP: | Administrator |
| GUID: | ab255743-ee8b-43de-b223-fdc... |

| Population | #Events | %Parent | FSC-A Mean | SSC-A Mean | PE-Texa... Mean |
|---|---|---|---|---|---|
| All Events | 11,470 | #### | 4,990 | 2,860 | 1,043 |
| P1 | 10,657 | 92.9 | 5,208 | 2,618 | 1,091 |

BD FACSDiva 8.0.1

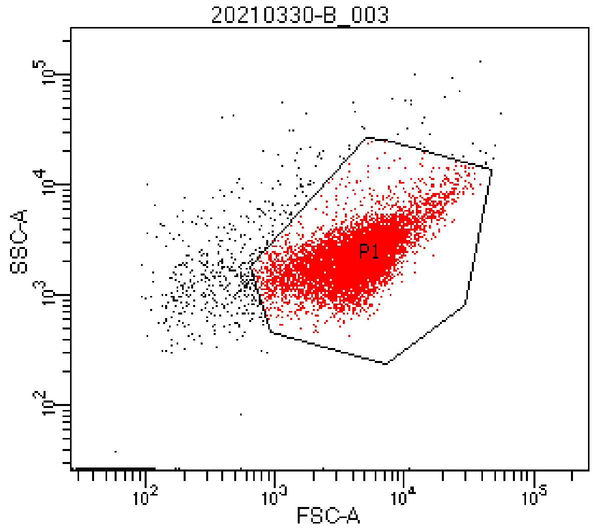


Tube: B_003

| Population | #Events | %Parent | %Total |
|---|---|---|---|
| All Events | 9,857 | #### | 100.0 |
| P1 | 9,109 | 92.4 | 92.4 |

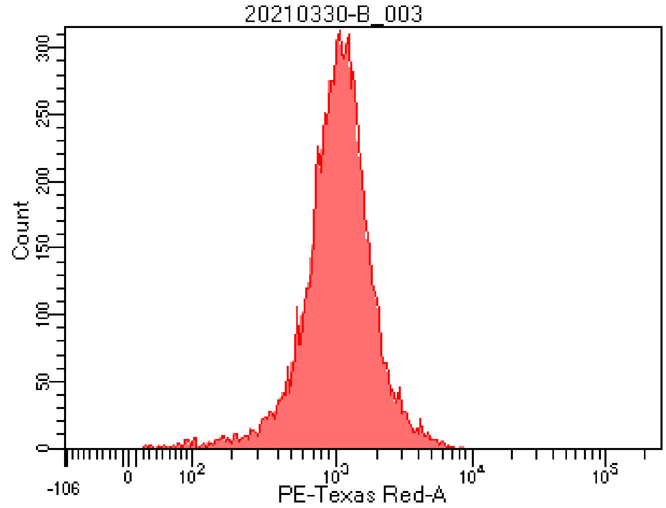


| | |
|---|---|
| Experiment Name: | Experiment-ROS |
| Specimen Name: | 20210330 |
| Tube Name: | B_003 |
| Record Date: | Mar 30, 2021 5:24:26 PM |
| $OP: | Administrator |
| GUID: | 3d4cfd79-dd23-44cf-8aa9-6e99... |

| Population | #Events | %Parent | FSC-A Mean | SSC-A Mean | PE-Texa... Mean |
|---|---|---|---|---|---|
| All Events | 9,857 | #### | 4,847 | 2,670 | 1,090 |
| P1 | 9,109 | 92.4 | 5,129 | 2,553 | 1,152 |
