# Supplementary figures and images for: Protein Lactylation Critically Regulates Energy Metabolism in the Protozoan Parasite Trypanosoma brucei (part 2 of 2)
Source: Front Cell Dev Biol. 2021 Oct 14;9:719720. doi: 10.3389/fcell.2021.719720 (PMC8551762; doi:10.3389/fcell.2021.719720)

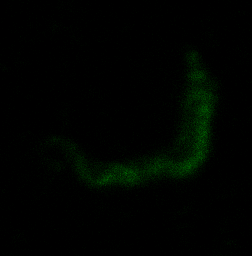

Supplement: Supplementary file 5 [file Data_Sheet_5.ZIP › Original Data 3-IFA/Fluorescence intensity statistics/Glucose 25 mM/2.tif]

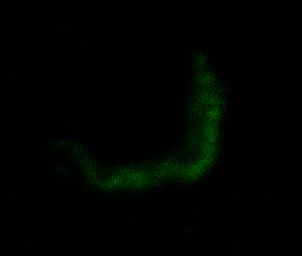

Supplement: Supplementary file 5 [file Data_Sheet_5.ZIP › Original Data 3-IFA/Fluorescence intensity statistics/Glucose 25 mM/1.tif]

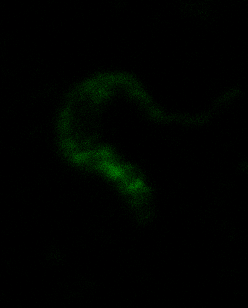

Supplement: Supplementary file 5 [file Data_Sheet_5.ZIP › Original Data 3-IFA/Fluorescence intensity statistics/Glucose 25 mM/5.tif]

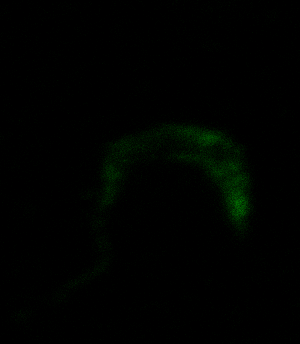

Supplement: Supplementary file 5 [file Data_Sheet_5.ZIP › Original Data 3-IFA/Fluorescence intensity statistics/Glucose 25 mM/4.tif]

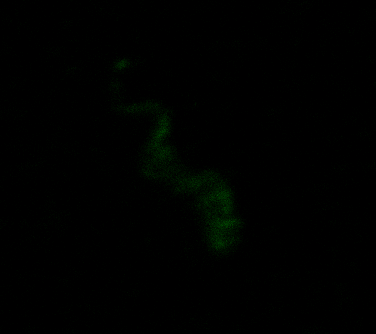

Supplement: Supplementary file 5 [file Data_Sheet_5.ZIP › Original Data 3-IFA/Fluorescence intensity statistics/Glucose 25 mM/6.tif]

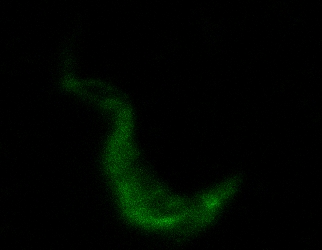

Supplement: Supplementary file 5 [file Data_Sheet_5.ZIP › Original Data 3-IFA/Fluorescence intensity statistics/Glucose 1 mM/8.tif]

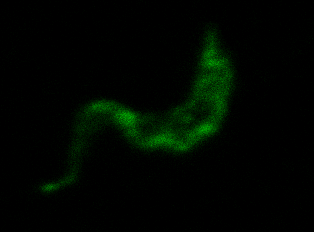

Supplement: Supplementary file 5 [file Data_Sheet_5.ZIP › Original Data 3-IFA/Fluorescence intensity statistics/Glucose 1 mM/3.tif]

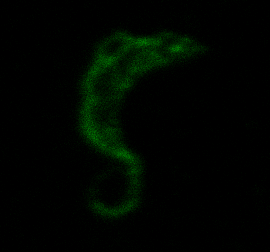

Supplement: Supplementary file 5 [file Data_Sheet_5.ZIP › Original Data 3-IFA/Fluorescence intensity statistics/Glucose 1 mM/2.tif]

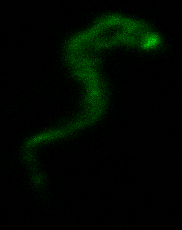

Supplement: Supplementary file 5 [file Data_Sheet_5.ZIP › Original Data 3-IFA/Fluorescence intensity statistics/Glucose 1 mM/1.tif]

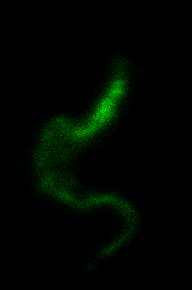

Supplement: Supplementary file 5 [file Data_Sheet_5.ZIP › Original Data 3-IFA/Fluorescence intensity statistics/Glucose 1 mM/5.tif]

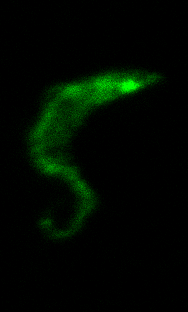

Supplement: Supplementary file 5 [file Data_Sheet_5.ZIP › Original Data 3-IFA/Fluorescence intensity statistics/Glucose 1 mM/4.tif]

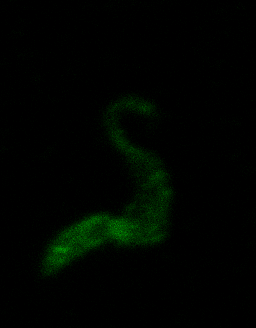

Supplement: Supplementary file 5 [file Data_Sheet_5.ZIP › Original Data 3-IFA/Fluorescence intensity statistics/Glucose 1 mM/6.tif]

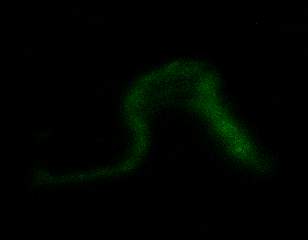

Supplement: Supplementary file 5 [file Data_Sheet_5.ZIP › Original Data 3-IFA/Fluorescence intensity statistics/Glucose 1 mM/7.tif]

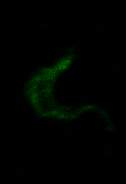

Supplement: Supplementary file 5 [file Data_Sheet_5.ZIP › Original Data 3-IFA/Fluorescence intensity statistics/Glucose 5 mM/9.tif]

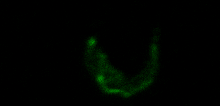

Supplement: Supplementary file 5 [file Data_Sheet_5.ZIP › Original Data 3-IFA/Fluorescence intensity statistics/Glucose 5 mM/8.tif]

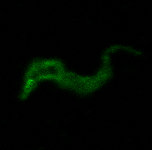

Supplement: Supplementary file 5 [file Data_Sheet_5.ZIP › Original Data 3-IFA/Fluorescence intensity statistics/Glucose 5 mM/3.tif]

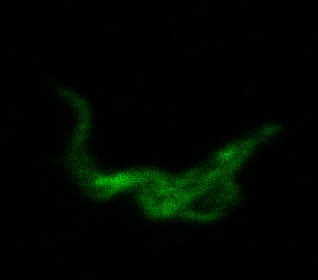

Supplement: Supplementary file 5 [file Data_Sheet_5.ZIP › Original Data 3-IFA/Fluorescence intensity statistics/Glucose 5 mM/2.tif]

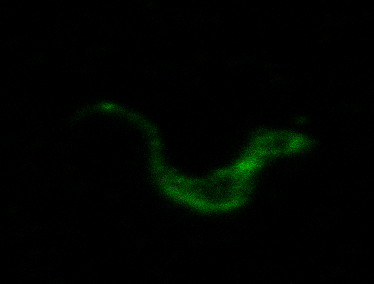

Supplement: Supplementary file 5 [file Data_Sheet_5.ZIP › Original Data 3-IFA/Fluorescence intensity statistics/Glucose 5 mM/1.tif]

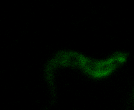

Supplement: Supplementary file 5 [file Data_Sheet_5.ZIP › Original Data 3-IFA/Fluorescence intensity statistics/Glucose 5 mM/5.tif]

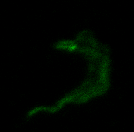

Supplement: Supplementary file 5 [file Data_Sheet_5.ZIP › Original Data 3-IFA/Fluorescence intensity statistics/Glucose 5 mM/4.tif]

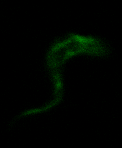

Supplement: Supplementary file 5 [file Data_Sheet_5.ZIP › Original Data 3-IFA/Fluorescence intensity statistics/Glucose 5 mM/6.tif]

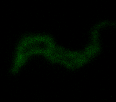

Supplement: Supplementary file 5 [file Data_Sheet_5.ZIP › Original Data 3-IFA/Fluorescence intensity statistics/Glucose 5 mM/7.tif]

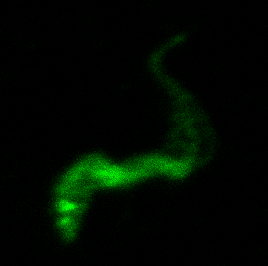

Supplement: Supplementary file 5 [file Data_Sheet_5.ZIP › Original Data 3-IFA/Fluorescence intensity statistics/Glucose 0 mM/3.tif]

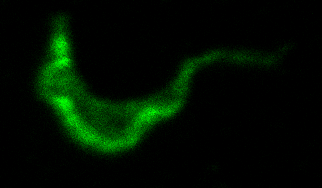

Supplement: Supplementary file 5 [file Data_Sheet_5.ZIP › Original Data 3-IFA/Fluorescence intensity statistics/Glucose 0 mM/2.tif]

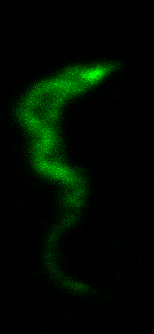

Supplement: Supplementary file 5 [file Data_Sheet_5.ZIP › Original Data 3-IFA/Fluorescence intensity statistics/Glucose 0 mM/1.tif]

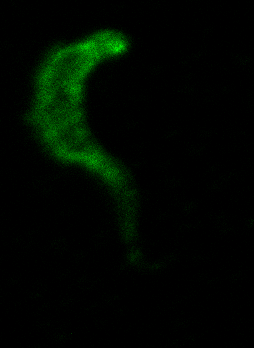

Supplement: Supplementary file 5 [file Data_Sheet_5.ZIP › Original Data 3-IFA/Fluorescence intensity statistics/Glucose 0 mM/5.tif]

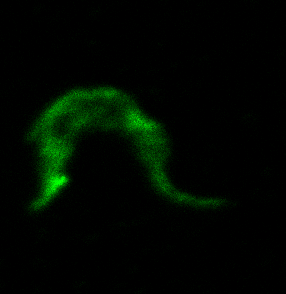

Supplement: Supplementary file 5 [file Data_Sheet_5.ZIP › Original Data 3-IFA/Fluorescence intensity statistics/Glucose 0 mM/4.tif]

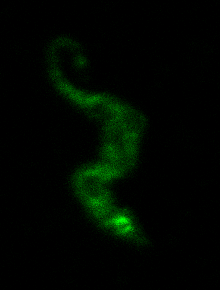

Supplement: Supplementary file 5 [file Data_Sheet_5.ZIP › Original Data 3-IFA/Fluorescence intensity statistics/Glucose 0 mM/6.tif]

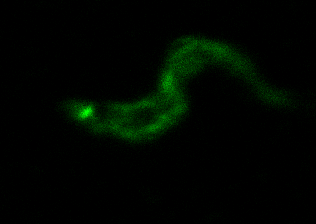

Supplement: Supplementary file 5 [file Data_Sheet_5.ZIP › Original Data 3-IFA/Fluorescence intensity statistics/Glucose 0 mM/7.tif]

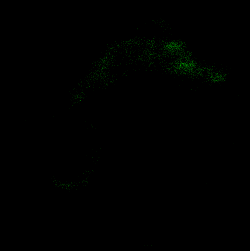

Supplement: Supplementary file 5 [file Data_Sheet_5.ZIP › Original Data 3-IFA/Fluorescence intensity statistics/2-DG 1 mM/10.tif]

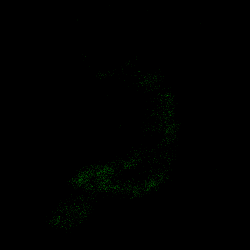

Supplement: Supplementary file 5 [file Data_Sheet_5.ZIP › Original Data 3-IFA/Fluorescence intensity statistics/2-DG 1 mM/9.tif]

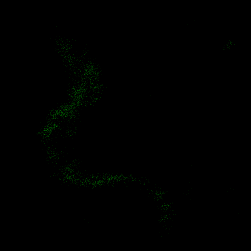

Supplement: Supplementary file 5 [file Data_Sheet_5.ZIP › Original Data 3-IFA/Fluorescence intensity statistics/2-DG 1 mM/8.tif]

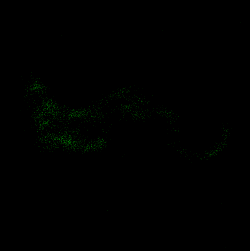

Supplement: Supplementary file 5 [file Data_Sheet_5.ZIP › Original Data 3-IFA/Fluorescence intensity statistics/2-DG 1 mM/3.tif]

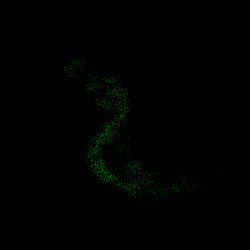

Supplement: Supplementary file 5 [file Data_Sheet_5.ZIP › Original Data 3-IFA/Fluorescence intensity statistics/2-DG 1 mM/2.tif]

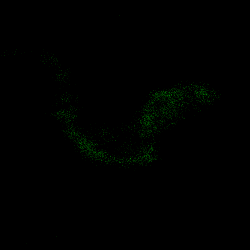

Supplement: Supplementary file 5 [file Data_Sheet_5.ZIP › Original Data 3-IFA/Fluorescence intensity statistics/2-DG 1 mM/1.tif]

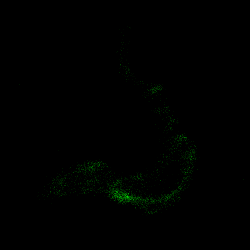

Supplement: Supplementary file 5 [file Data_Sheet_5.ZIP › Original Data 3-IFA/Fluorescence intensity statistics/2-DG 1 mM/5.tif]

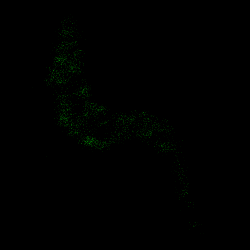

Supplement: Supplementary file 5 [file Data_Sheet_5.ZIP › Original Data 3-IFA/Fluorescence intensity statistics/2-DG 1 mM/4.tif]

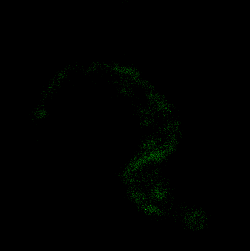

Supplement: Supplementary file 5 [file Data_Sheet_5.ZIP › Original Data 3-IFA/Fluorescence intensity statistics/2-DG 1 mM/6.tif]

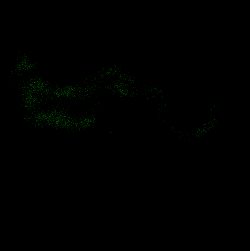

Supplement: Supplementary file 5 [file Data_Sheet_5.ZIP › Original Data 3-IFA/Fluorescence intensity statistics/2-DG 1 mM/7.tif]

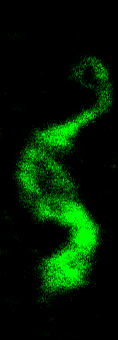

Supplement: Supplementary file 5 [file Data_Sheet_5.ZIP › Original Data 3-IFA/Fluorescence intensity statistics/Oxamate 5 mM/10.tif]

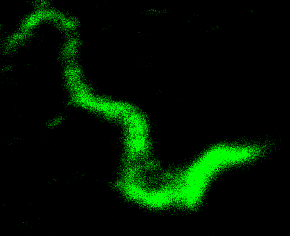

Supplement: Supplementary file 5 [file Data_Sheet_5.ZIP › Original Data 3-IFA/Fluorescence intensity statistics/Oxamate 5 mM/9.tif]

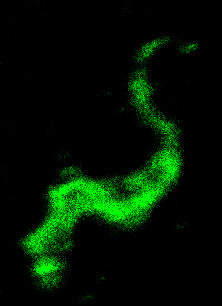

Supplement: Supplementary file 5 [file Data_Sheet_5.ZIP › Original Data 3-IFA/Fluorescence intensity statistics/Oxamate 5 mM/8.tif]

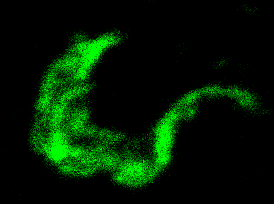

Supplement: Supplementary file 5 [file Data_Sheet_5.ZIP › Original Data 3-IFA/Fluorescence intensity statistics/Oxamate 5 mM/3.tif]

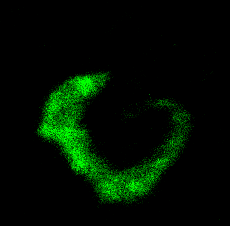

Supplement: Supplementary file 5 [file Data_Sheet_5.ZIP › Original Data 3-IFA/Fluorescence intensity statistics/Oxamate 5 mM/2.tif]

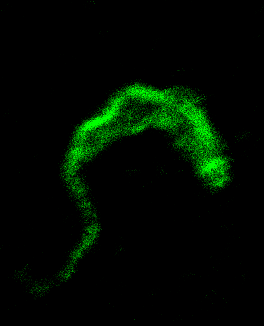

Supplement: Supplementary file 5 [file Data_Sheet_5.ZIP › Original Data 3-IFA/Fluorescence intensity statistics/Oxamate 5 mM/1.tif]

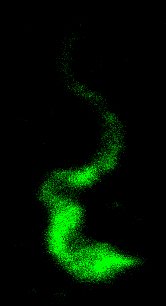

Supplement: Supplementary file 5 [file Data_Sheet_5.ZIP › Original Data 3-IFA/Fluorescence intensity statistics/Oxamate 5 mM/5.tif]

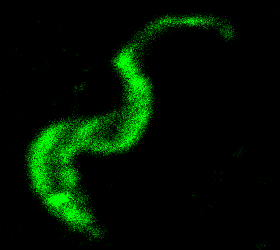

Supplement: Supplementary file 5 [file Data_Sheet_5.ZIP › Original Data 3-IFA/Fluorescence intensity statistics/Oxamate 5 mM/4.tif]

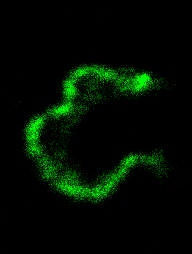

Supplement: Supplementary file 5 [file Data_Sheet_5.ZIP › Original Data 3-IFA/Fluorescence intensity statistics/Oxamate 5 mM/6.tif]

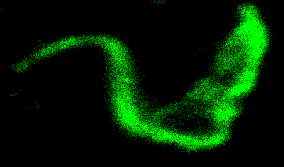

Supplement: Supplementary file 5 [file Data_Sheet_5.ZIP › Original Data 3-IFA/Fluorescence intensity statistics/Oxamate 5 mM/7.tif]

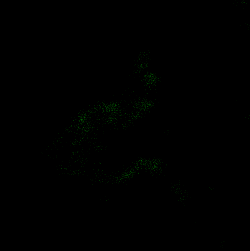

Supplement: Supplementary file 5 [file Data_Sheet_5.ZIP › Original Data 3-IFA/Fluorescence intensity statistics/2-DG 5 mM/10.tif]

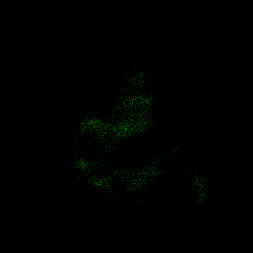

Supplement: Supplementary file 5 [file Data_Sheet_5.ZIP › Original Data 3-IFA/Fluorescence intensity statistics/2-DG 5 mM/9.tif]

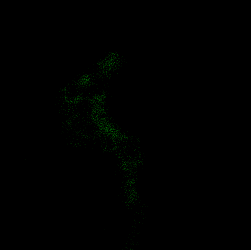

Supplement: Supplementary file 5 [file Data_Sheet_5.ZIP › Original Data 3-IFA/Fluorescence intensity statistics/2-DG 5 mM/8.tif]

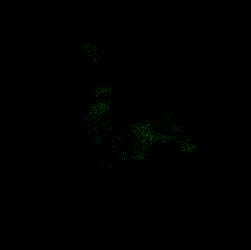

Supplement: Supplementary file 5 [file Data_Sheet_5.ZIP › Original Data 3-IFA/Fluorescence intensity statistics/2-DG 5 mM/3.tif]

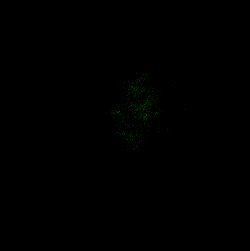

Supplement: Supplementary file 5 [file Data_Sheet_5.ZIP › Original Data 3-IFA/Fluorescence intensity statistics/2-DG 5 mM/2.tif]

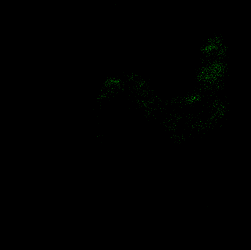

Supplement: Supplementary file 5 [file Data_Sheet_5.ZIP › Original Data 3-IFA/Fluorescence intensity statistics/2-DG 5 mM/1.tif]

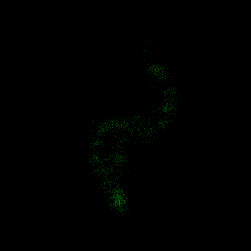

Supplement: Supplementary file 5 [file Data_Sheet_5.ZIP › Original Data 3-IFA/Fluorescence intensity statistics/2-DG 5 mM/5.tif]

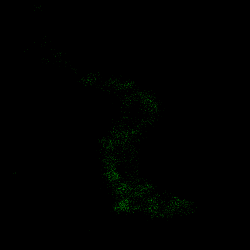

Supplement: Supplementary file 5 [file Data_Sheet_5.ZIP › Original Data 3-IFA/Fluorescence intensity statistics/2-DG 5 mM/4.tif]

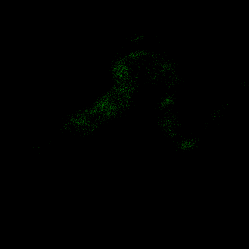

Supplement: Supplementary file 5 [file Data_Sheet_5.ZIP › Original Data 3-IFA/Fluorescence intensity statistics/2-DG 5 mM/6.tif]

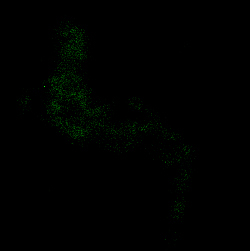

Supplement: Supplementary file 5 [file Data_Sheet_5.ZIP › Original Data 3-IFA/Fluorescence intensity statistics/2-DG 5 mM/7.tif]

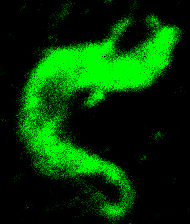

Supplement: Supplementary file 5 [file Data_Sheet_5.ZIP › Original Data 3-IFA/Fluorescence intensity statistics/Oxamate 0 mM/10.tif]

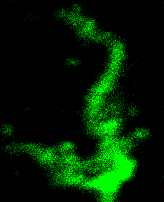

Supplement: Supplementary file 5 [file Data_Sheet_5.ZIP › Original Data 3-IFA/Fluorescence intensity statistics/Oxamate 0 mM/9.tif]

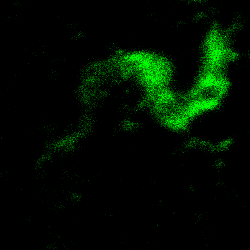

Supplement: Supplementary file 5 [file Data_Sheet_5.ZIP › Original Data 3-IFA/Fluorescence intensity statistics/Oxamate 0 mM/8.tif]

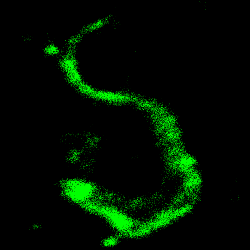

Supplement: Supplementary file 5 [file Data_Sheet_5.ZIP › Original Data 3-IFA/Fluorescence intensity statistics/Oxamate 0 mM/3.tif]

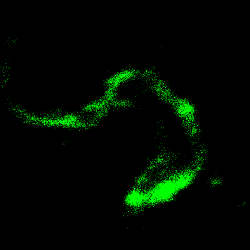

Supplement: Supplementary file 5 [file Data_Sheet_5.ZIP › Original Data 3-IFA/Fluorescence intensity statistics/Oxamate 0 mM/2.tif]

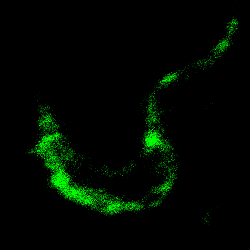

Supplement: Supplementary file 5 [file Data_Sheet_5.ZIP › Original Data 3-IFA/Fluorescence intensity statistics/Oxamate 0 mM/1.tif]

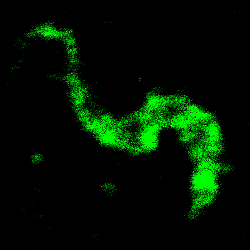

Supplement: Supplementary file 5 [file Data_Sheet_5.ZIP › Original Data 3-IFA/Fluorescence intensity statistics/Oxamate 0 mM/5.tif]

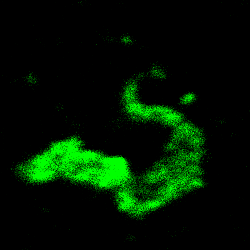

Supplement: Supplementary file 5 [file Data_Sheet_5.ZIP › Original Data 3-IFA/Fluorescence intensity statistics/Oxamate 0 mM/4.tif]

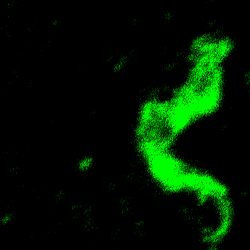

Supplement: Supplementary file 5 [file Data_Sheet_5.ZIP › Original Data 3-IFA/Fluorescence intensity statistics/Oxamate 0 mM/6.tif]

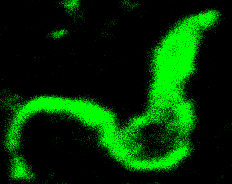

Supplement: Supplementary file 5 [file Data_Sheet_5.ZIP › Original Data 3-IFA/Fluorescence intensity statistics/Oxamate 0 mM/7.tif]

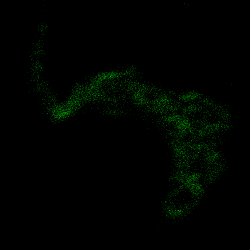

Supplement: Supplementary file 5 [file Data_Sheet_5.ZIP › Original Data 3-IFA/Fluorescence intensity statistics/2-DG 0 mM/10.tif]

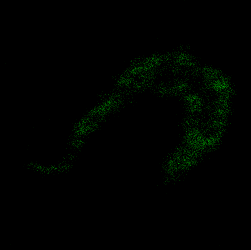

Supplement: Supplementary file 5 [file Data_Sheet_5.ZIP › Original Data 3-IFA/Fluorescence intensity statistics/2-DG 0 mM/9.tif]

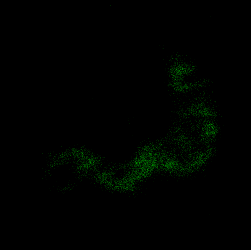

Supplement: Supplementary file 5 [file Data_Sheet_5.ZIP › Original Data 3-IFA/Fluorescence intensity statistics/2-DG 0 mM/8.tif]

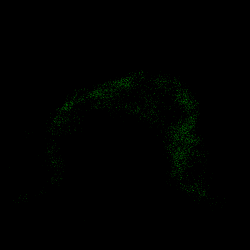

Supplement: Supplementary file 5 [file Data_Sheet_5.ZIP › Original Data 3-IFA/Fluorescence intensity statistics/2-DG 0 mM/3.tif]

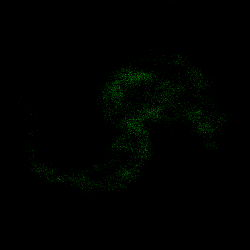

Supplement: Supplementary file 5 [file Data_Sheet_5.ZIP › Original Data 3-IFA/Fluorescence intensity statistics/2-DG 0 mM/2.tif]

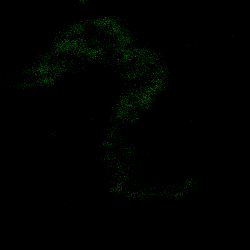

Supplement: Supplementary file 5 [file Data_Sheet_5.ZIP › Original Data 3-IFA/Fluorescence intensity statistics/2-DG 0 mM/1.tif]

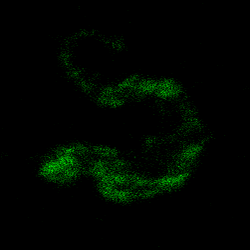

Supplement: Supplementary file 5 [file Data_Sheet_5.ZIP › Original Data 3-IFA/Fluorescence intensity statistics/2-DG 0 mM/5.tif]

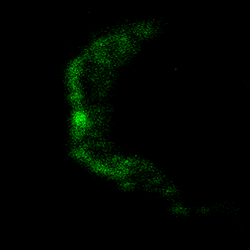

Supplement: Supplementary file 5 [file Data_Sheet_5.ZIP › Original Data 3-IFA/Fluorescence intensity statistics/2-DG 0 mM/4.tif]

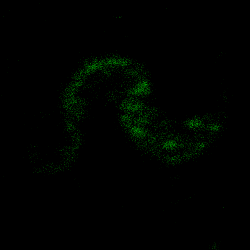

Supplement: Supplementary file 5 [file Data_Sheet_5.ZIP › Original Data 3-IFA/Fluorescence intensity statistics/2-DG 0 mM/6.tif]

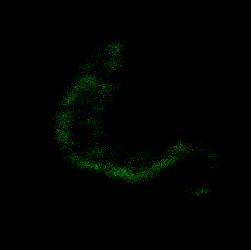

Supplement: Supplementary file 5 [file Data_Sheet_5.ZIP › Original Data 3-IFA/Fluorescence intensity statistics/2-DG 0 mM/7.tif]

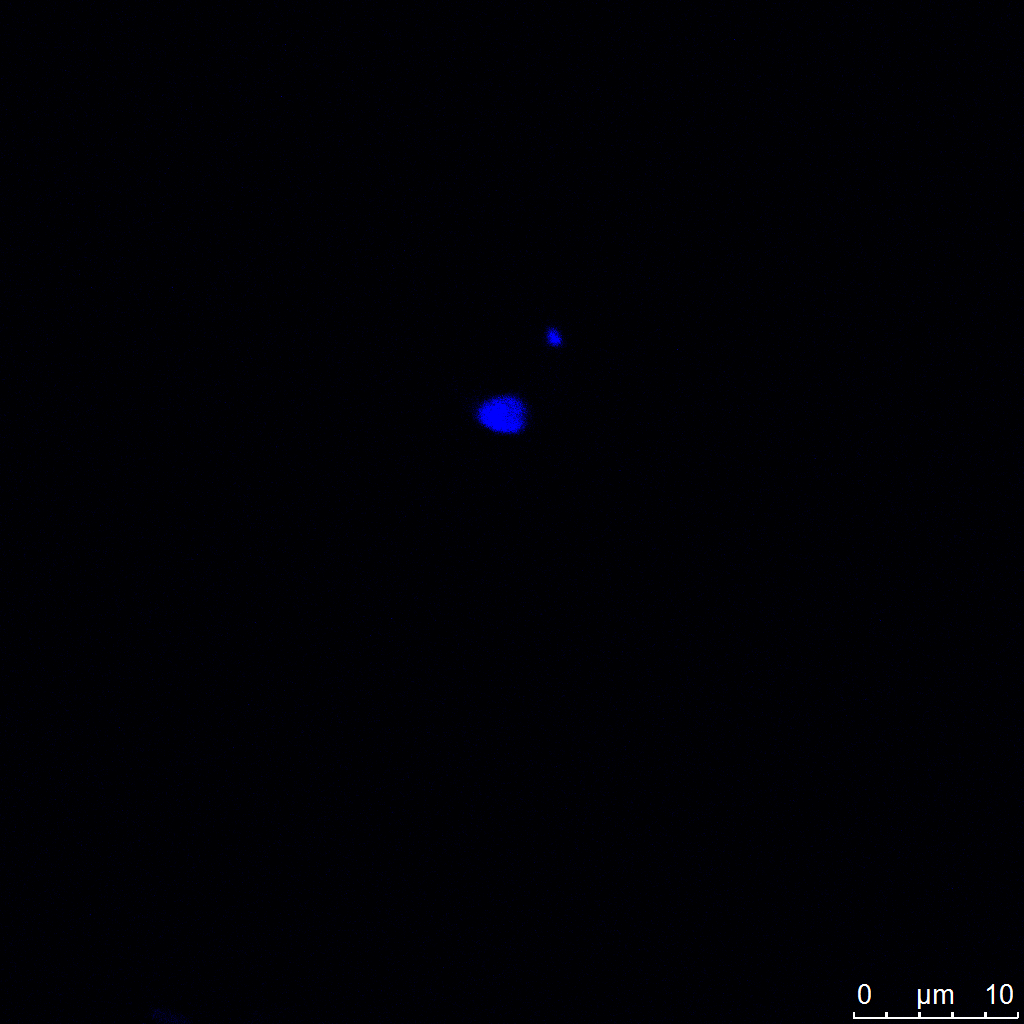

Supplement: Supplementary file 5 [file Data_Sheet_5.ZIP › Original Data 3-IFA/01 Glucose/1 mM/Project20200710_G1-3_z0_ch00.tif]

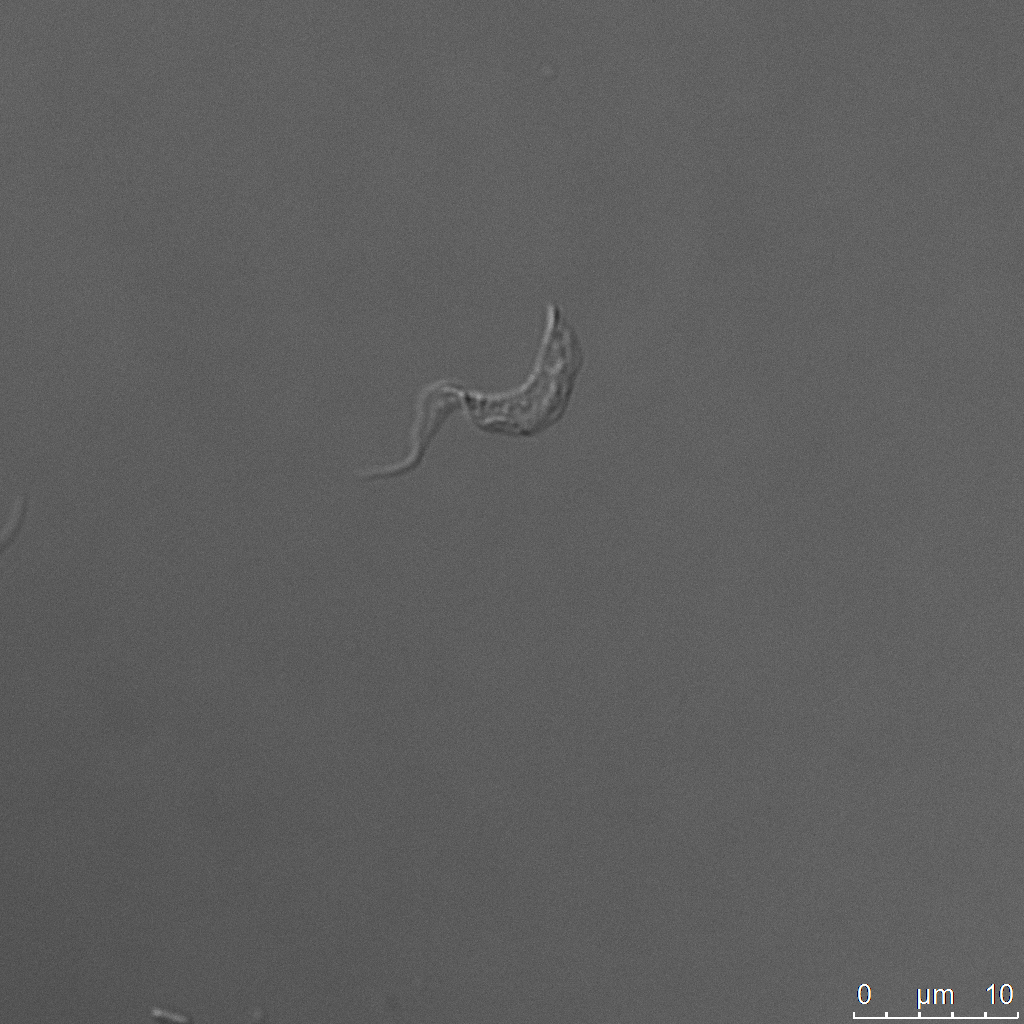

Supplement: Supplementary file 5 [file Data_Sheet_5.ZIP › Original Data 3-IFA/01 Glucose/1 mM/Project20200710_G1-3_z0_ch01.tif]

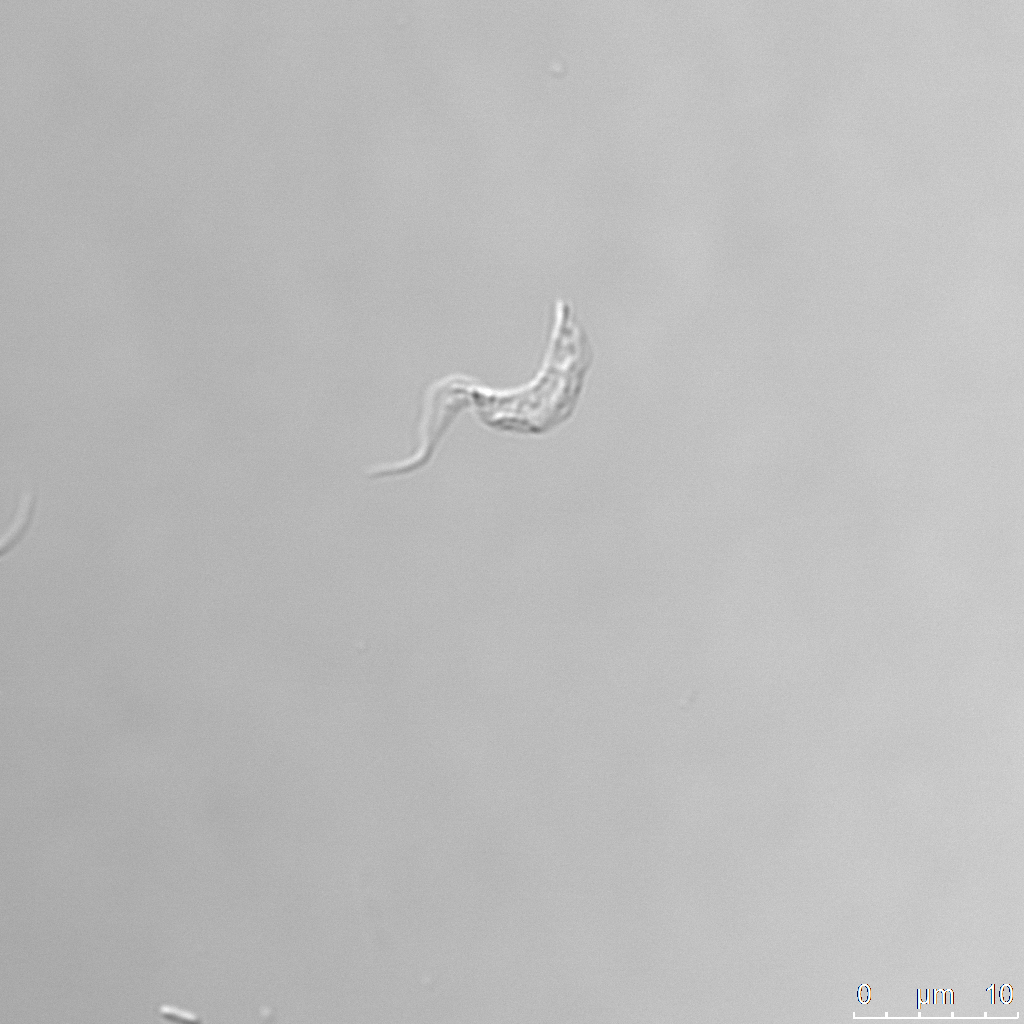

Supplement: Supplementary file 5 [file Data_Sheet_5.ZIP › Original Data 3-IFA/01 Glucose/1 mM/Project20200710_G1-3_z0_ch03.tif]

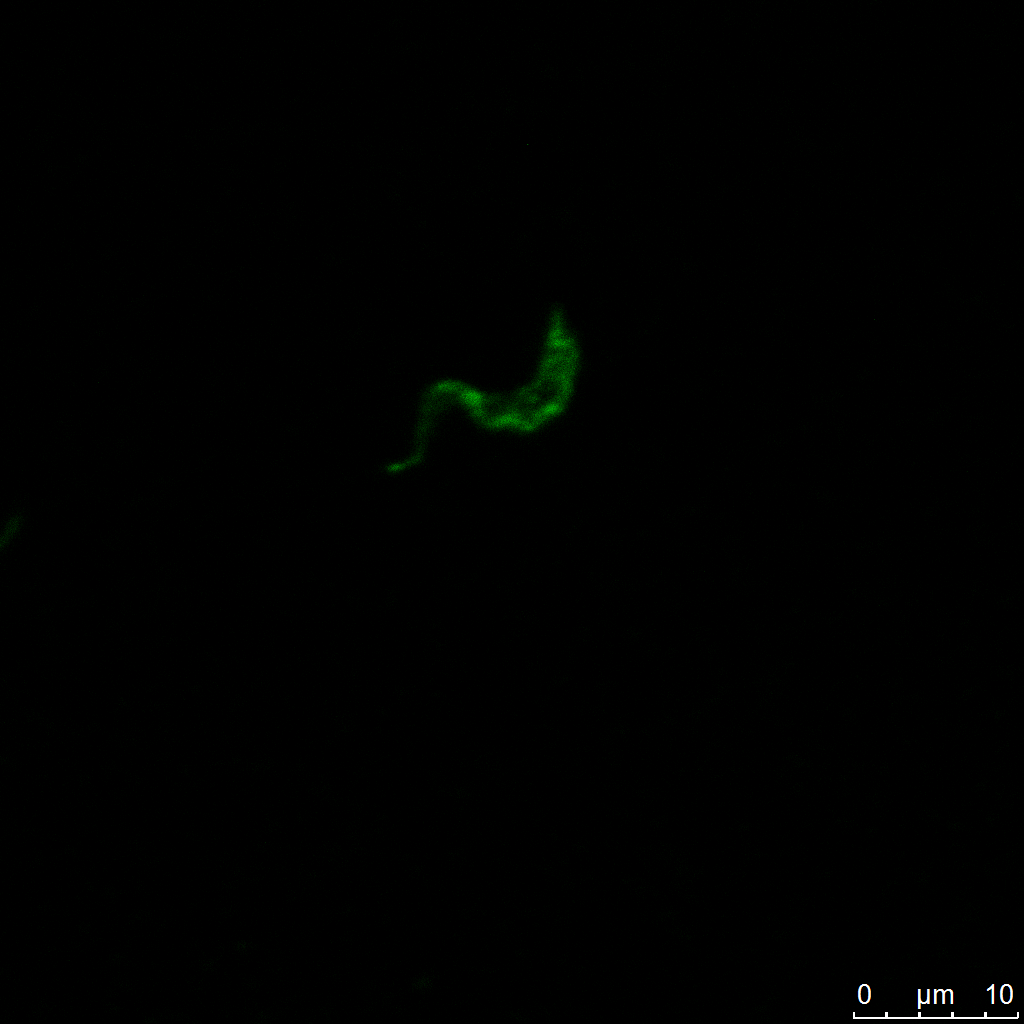

Supplement: Supplementary file 5 [file Data_Sheet_5.ZIP › Original Data 3-IFA/01 Glucose/1 mM/Project20200710_G1-3_z0_ch02.tif]

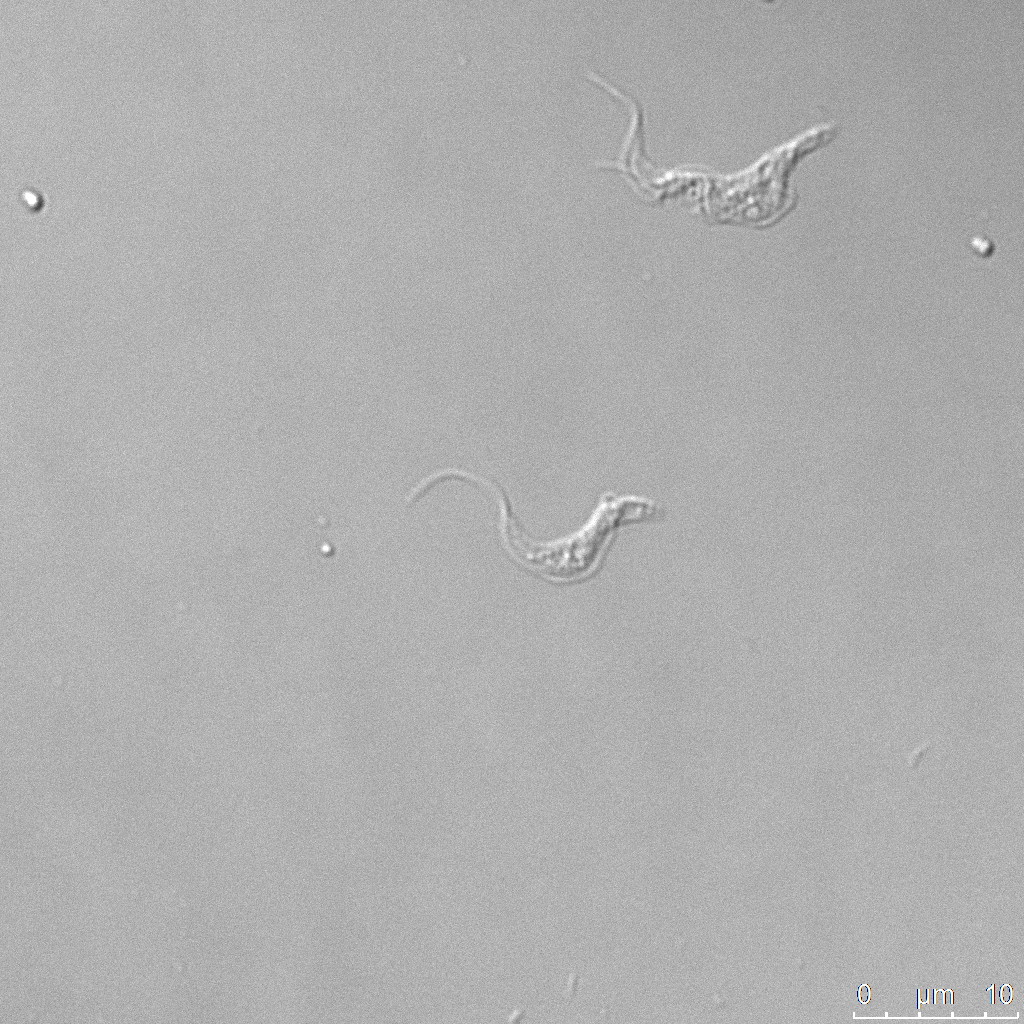

Supplement: Supplementary file 5 [file Data_Sheet_5.ZIP › Original Data 3-IFA/01 Glucose/5 mM/Project20200710_G5-1_z0_ch01.tif]

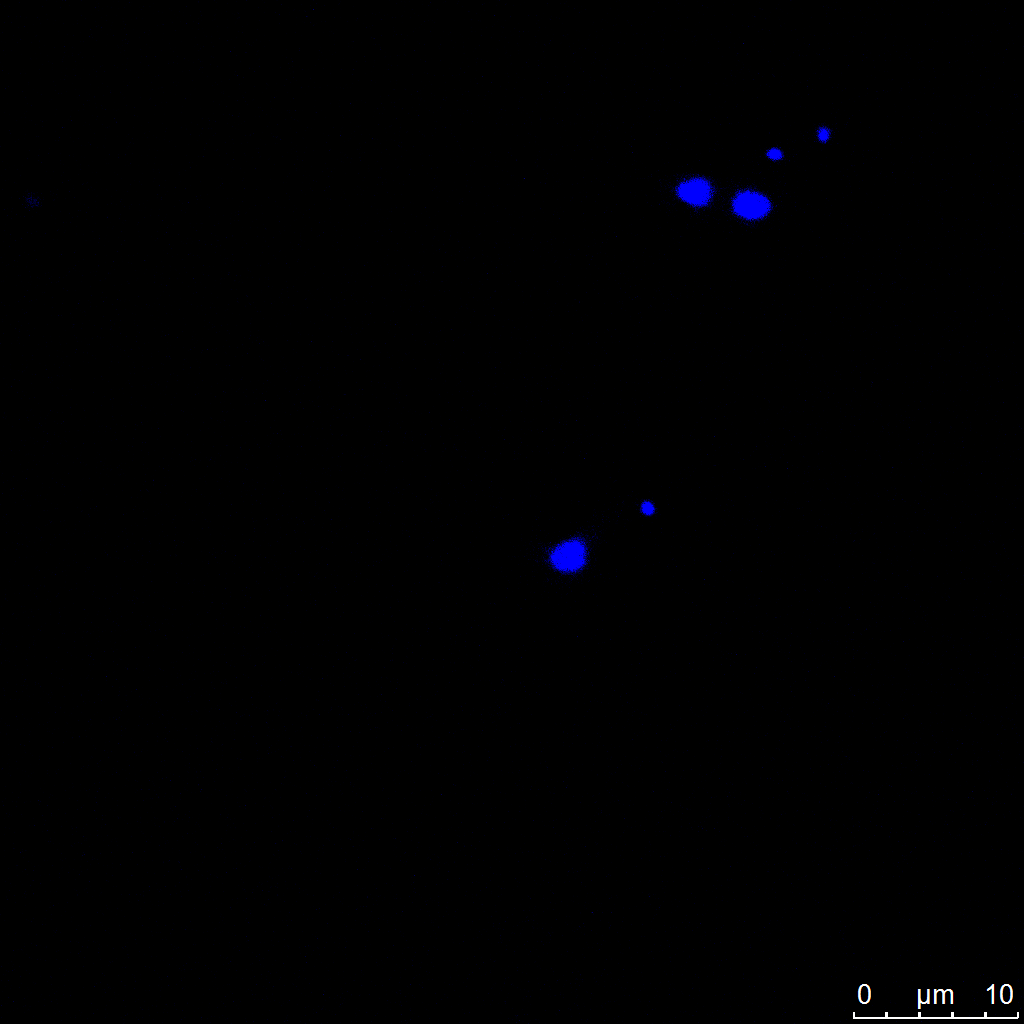

Supplement: Supplementary file 5 [file Data_Sheet_5.ZIP › Original Data 3-IFA/01 Glucose/5 mM/Project20200710_G5-1_z0_ch00.tif]

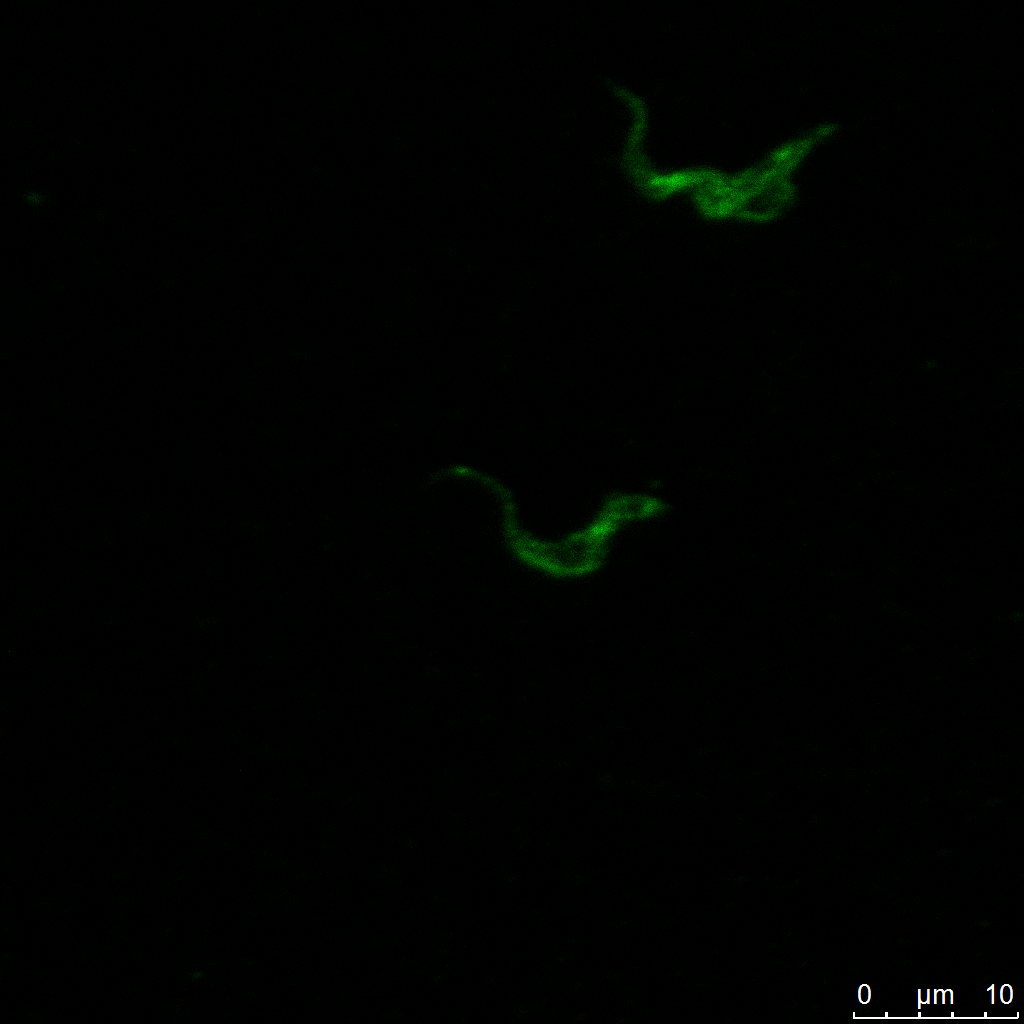

Supplement: Supplementary file 5 [file Data_Sheet_5.ZIP › Original Data 3-IFA/01 Glucose/5 mM/Project20200710_G5-1_z0_ch02.tif]

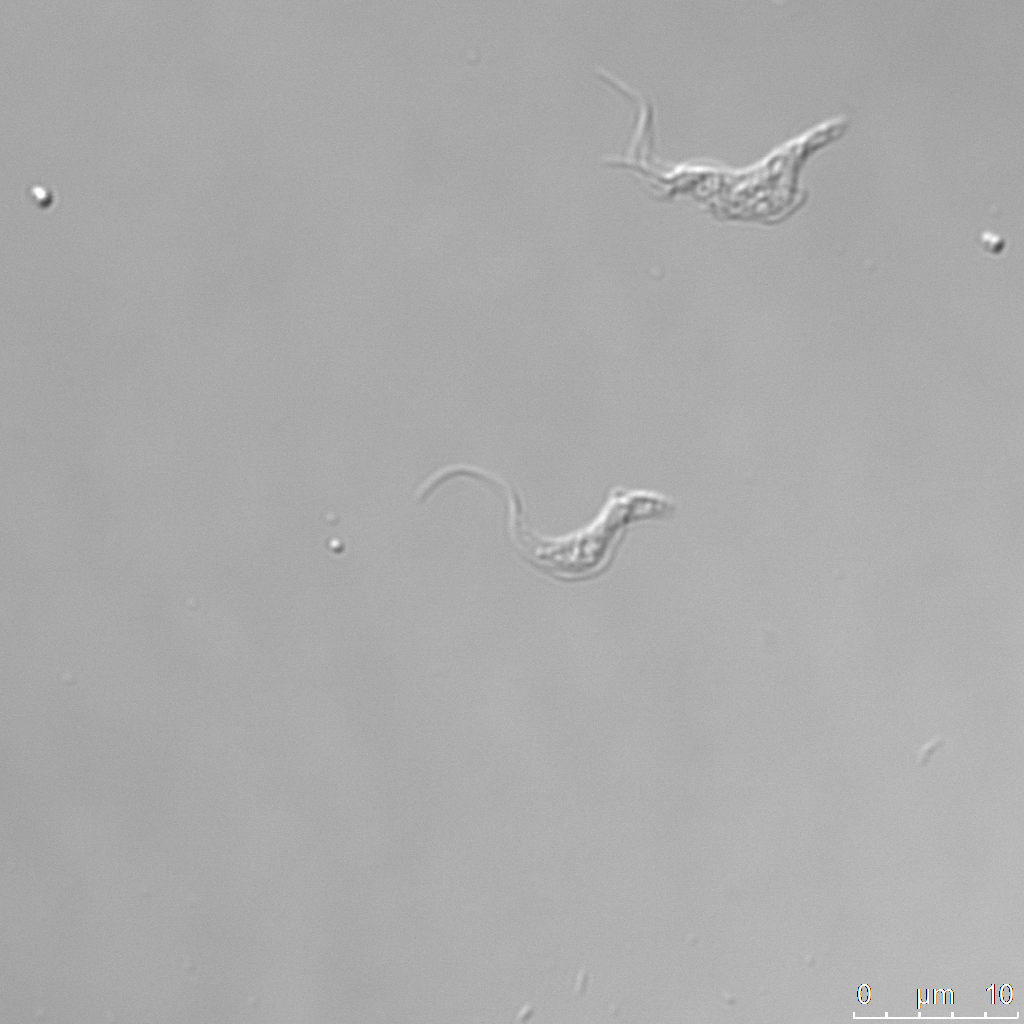

Supplement: Supplementary file 5 [file Data_Sheet_5.ZIP › Original Data 3-IFA/01 Glucose/5 mM/Project20200710_G5-1_z0_ch03.tif]

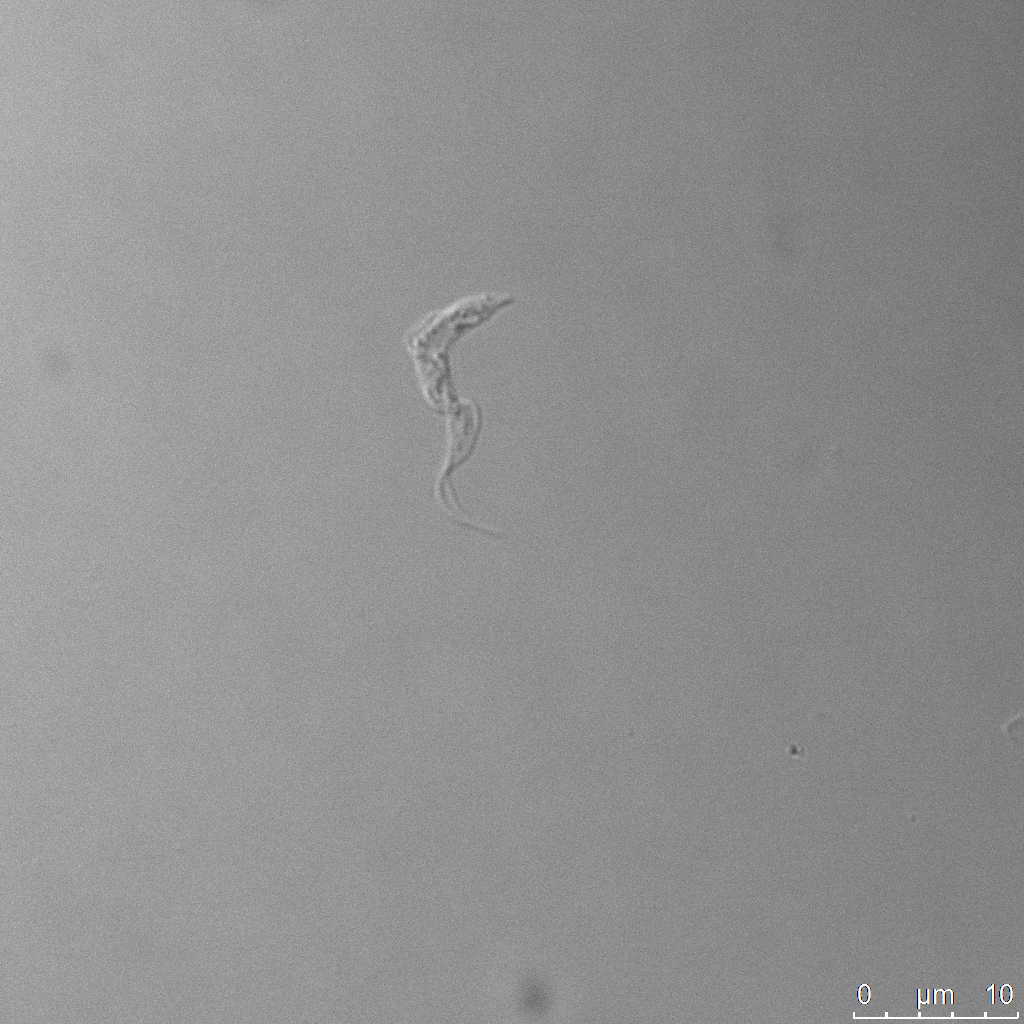

Supplement: Supplementary file 5 [file Data_Sheet_5.ZIP › Original Data 3-IFA/01 Glucose/0 mM/Project20200710_G0-1_z0_ch01.tif]

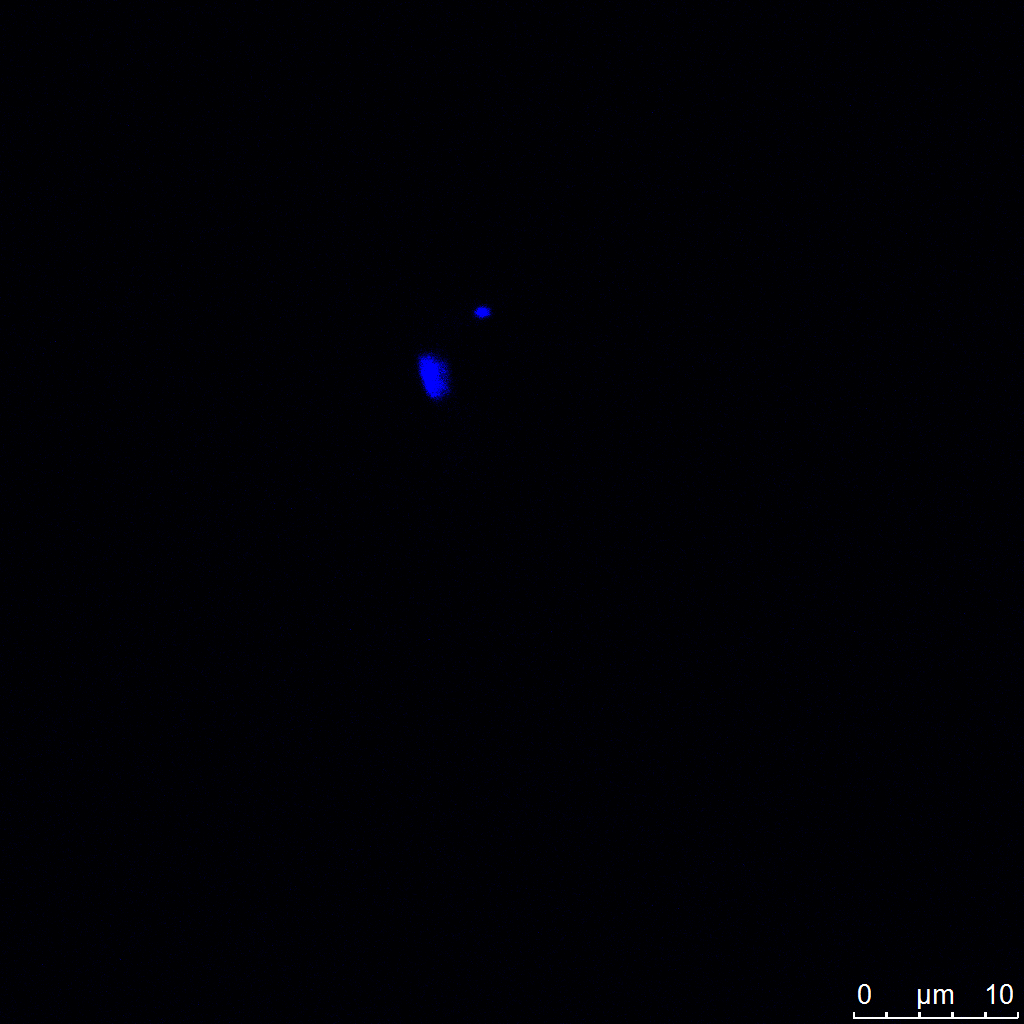

Supplement: Supplementary file 5 [file Data_Sheet_5.ZIP › Original Data 3-IFA/01 Glucose/0 mM/Project20200710_G0-1_z0_ch00.tif]

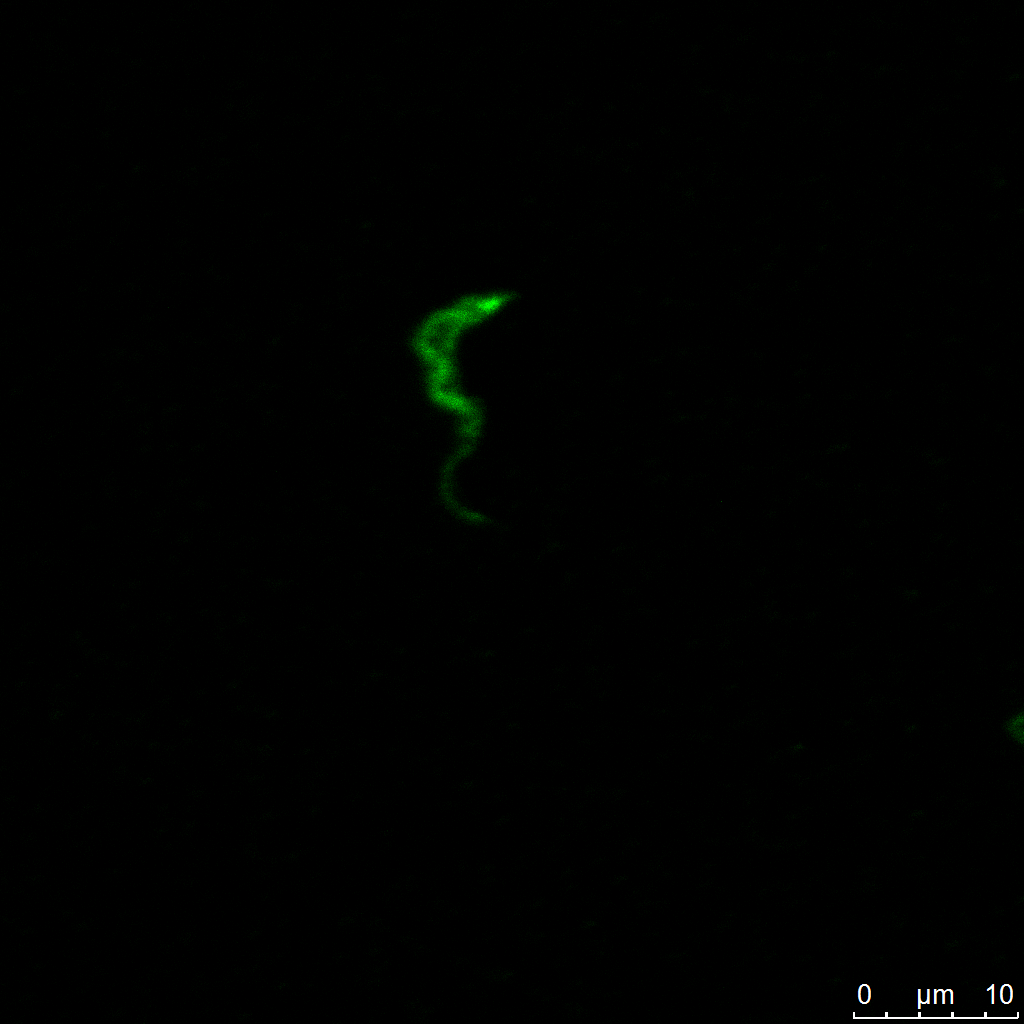

Supplement: Supplementary file 5 [file Data_Sheet_5.ZIP › Original Data 3-IFA/01 Glucose/0 mM/Project20200710_G0-1_z0_ch02.tif]

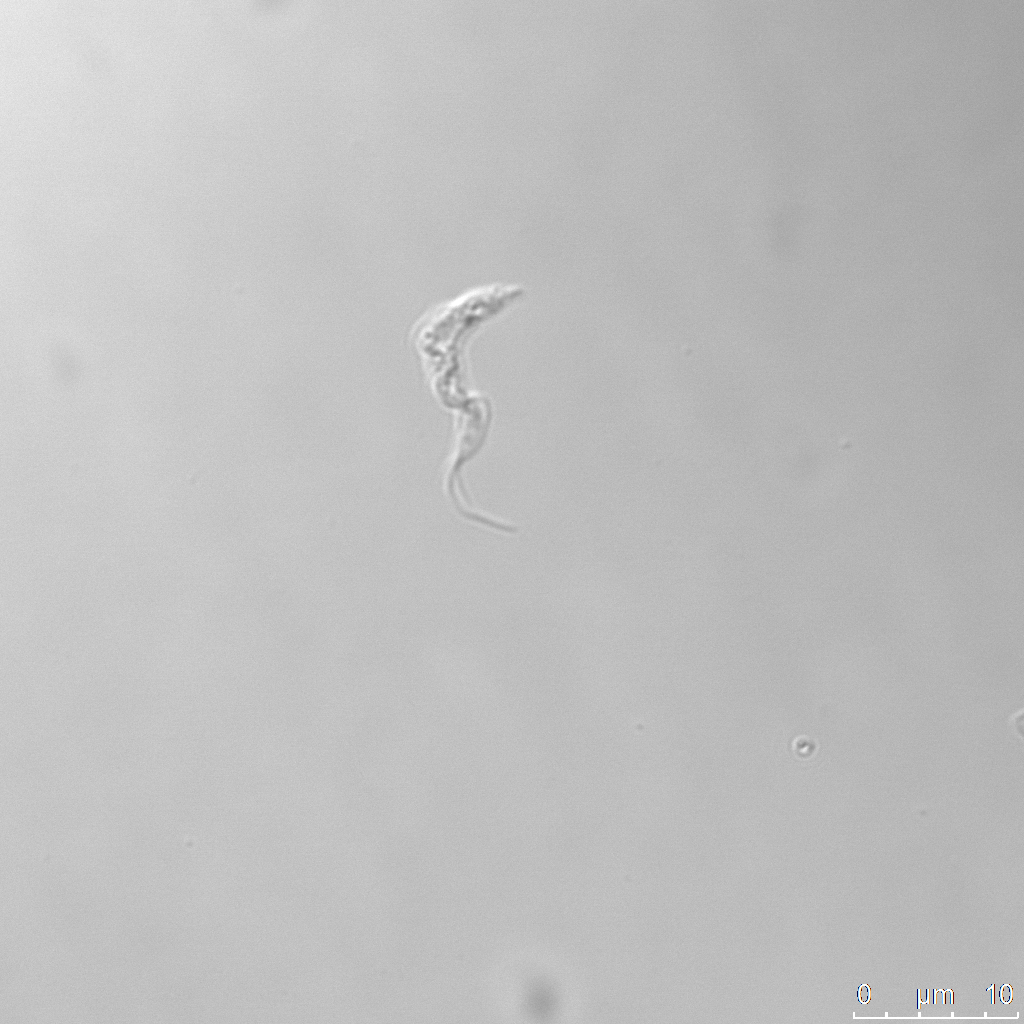

Supplement: Supplementary file 5 [file Data_Sheet_5.ZIP › Original Data 3-IFA/01 Glucose/0 mM/Project20200710_G0-1_z0_ch03.tif]

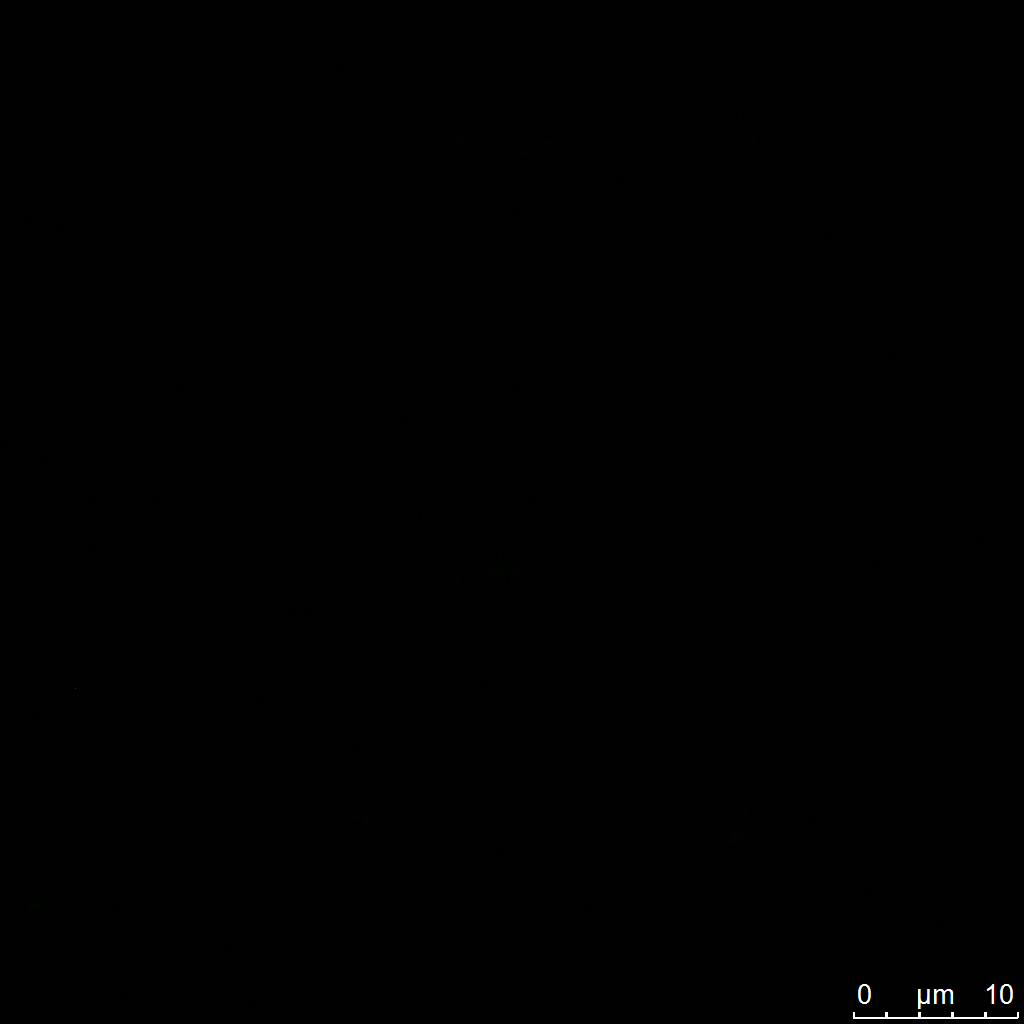

Supplement: Supplementary file 5 [file Data_Sheet_5.ZIP › Original Data 3-IFA/01 Glucose/Negative/Project20200710_0711-N-2_z0_ch02.tif]

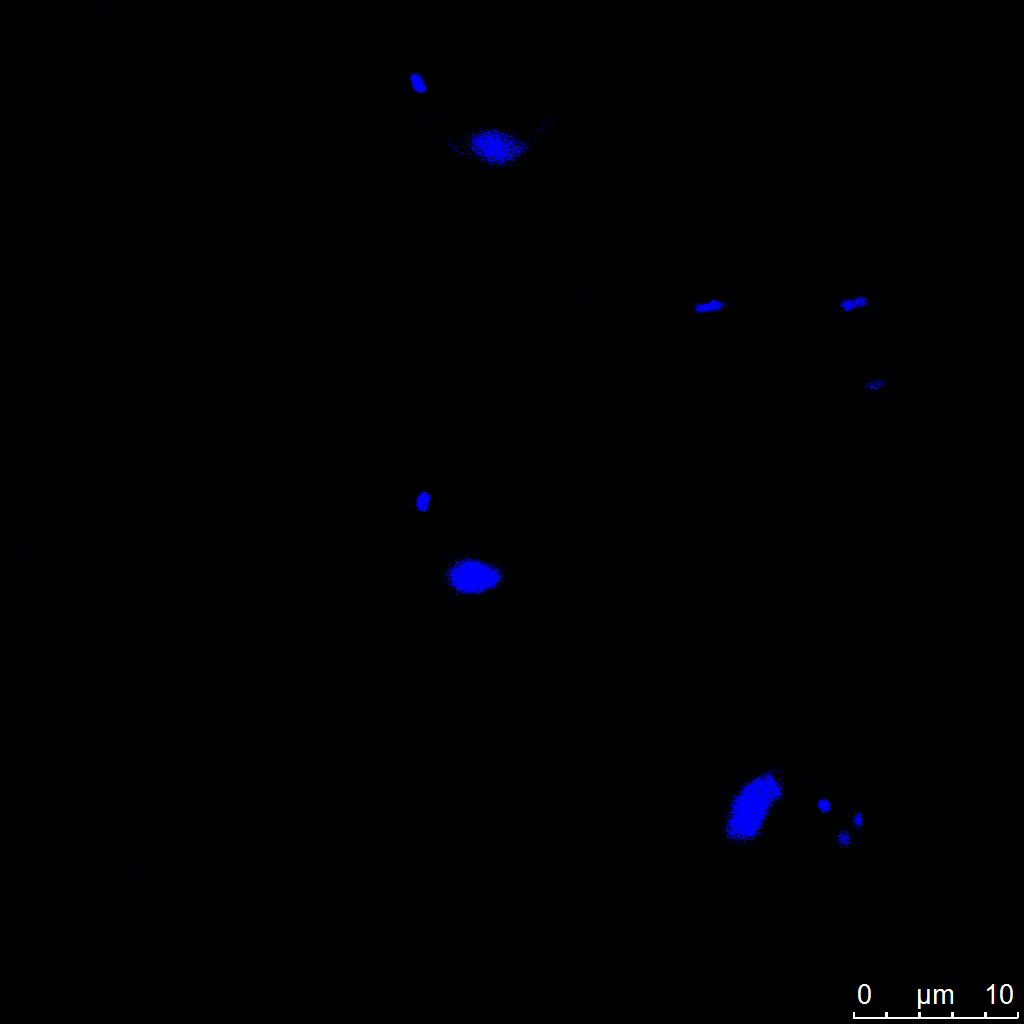

Supplement: Supplementary file 5 [file Data_Sheet_5.ZIP › Original Data 3-IFA/01 Glucose/Negative/Project20200710_0711-B-2_z0_ch00.tif]

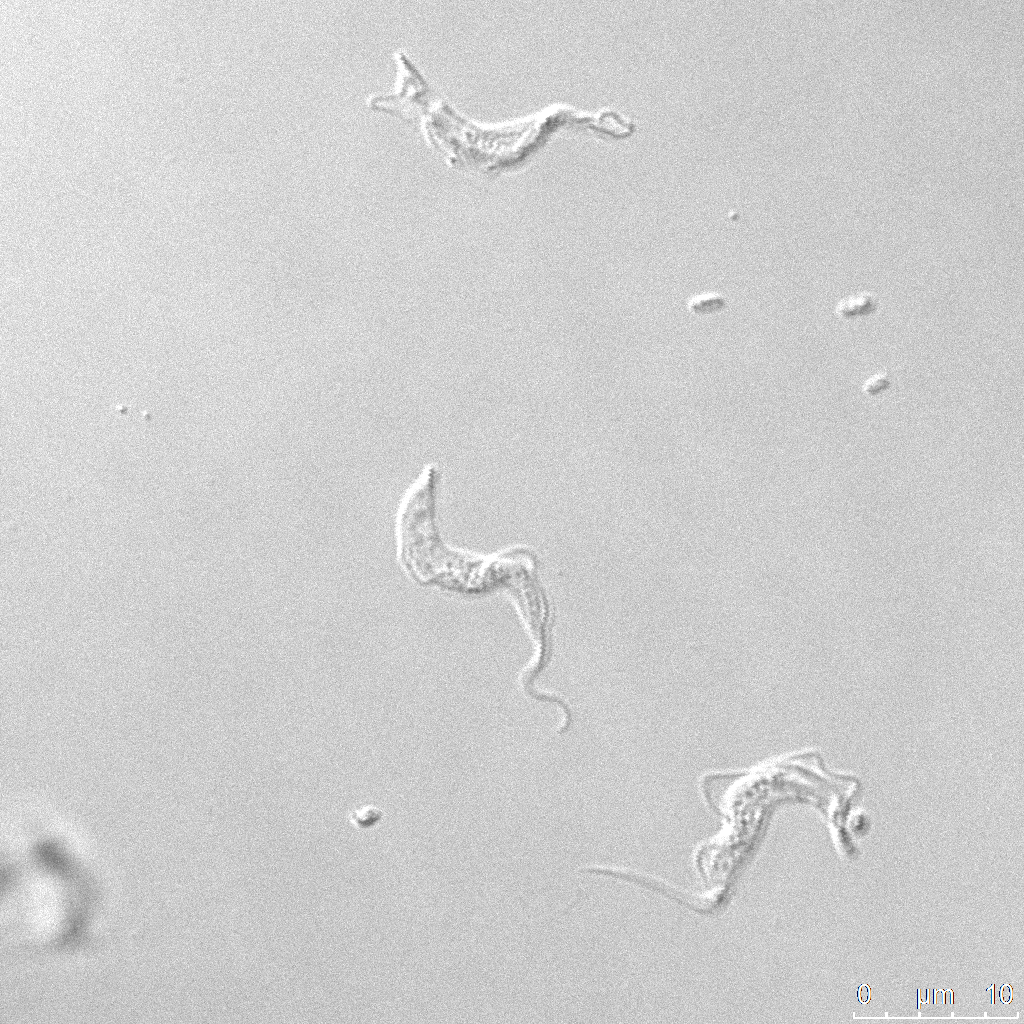

Supplement: Supplementary file 5 [file Data_Sheet_5.ZIP › Original Data 3-IFA/01 Glucose/Negative/Project20200710_0711-B-2_z0_ch01.tif]

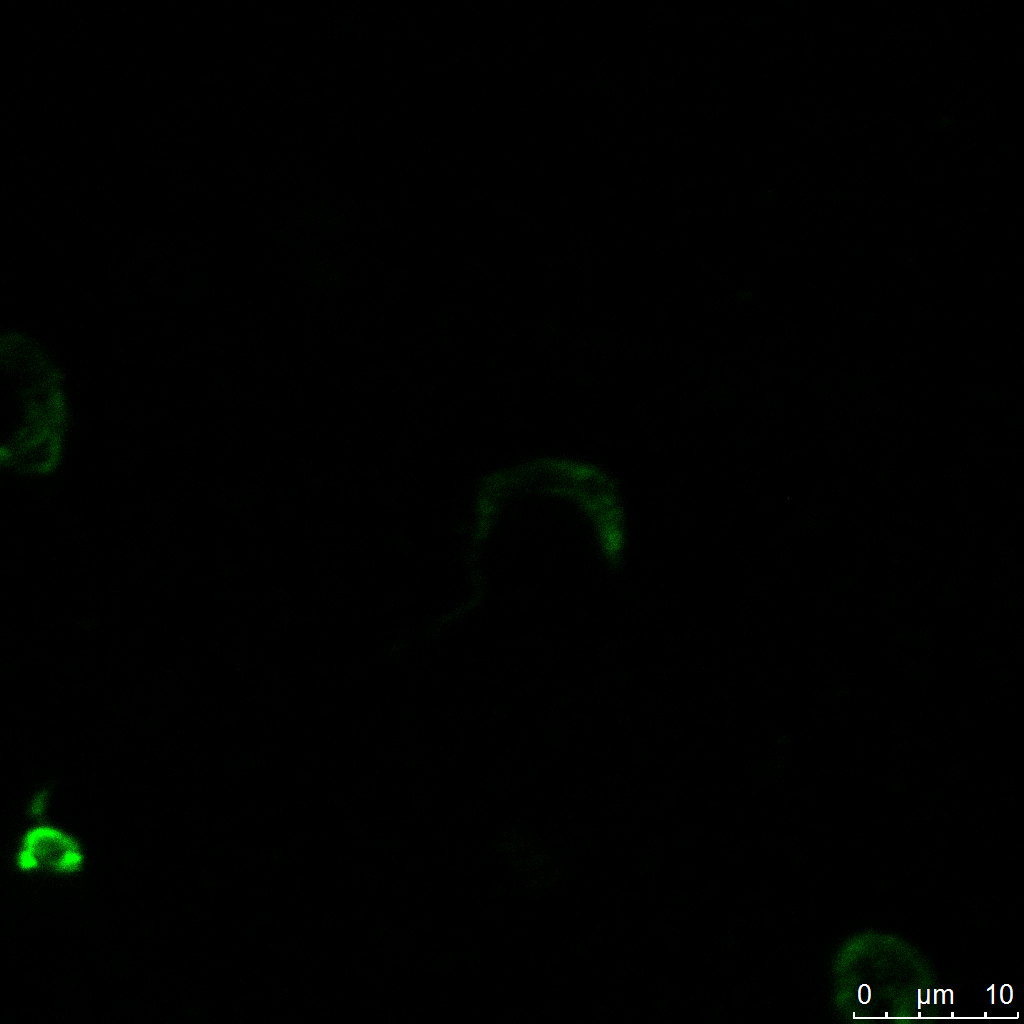

Supplement: Supplementary file 5 [file Data_Sheet_5.ZIP › Original Data 3-IFA/01 Glucose/25 mM/Project20200710_G25-4_z0_ch02.tif]

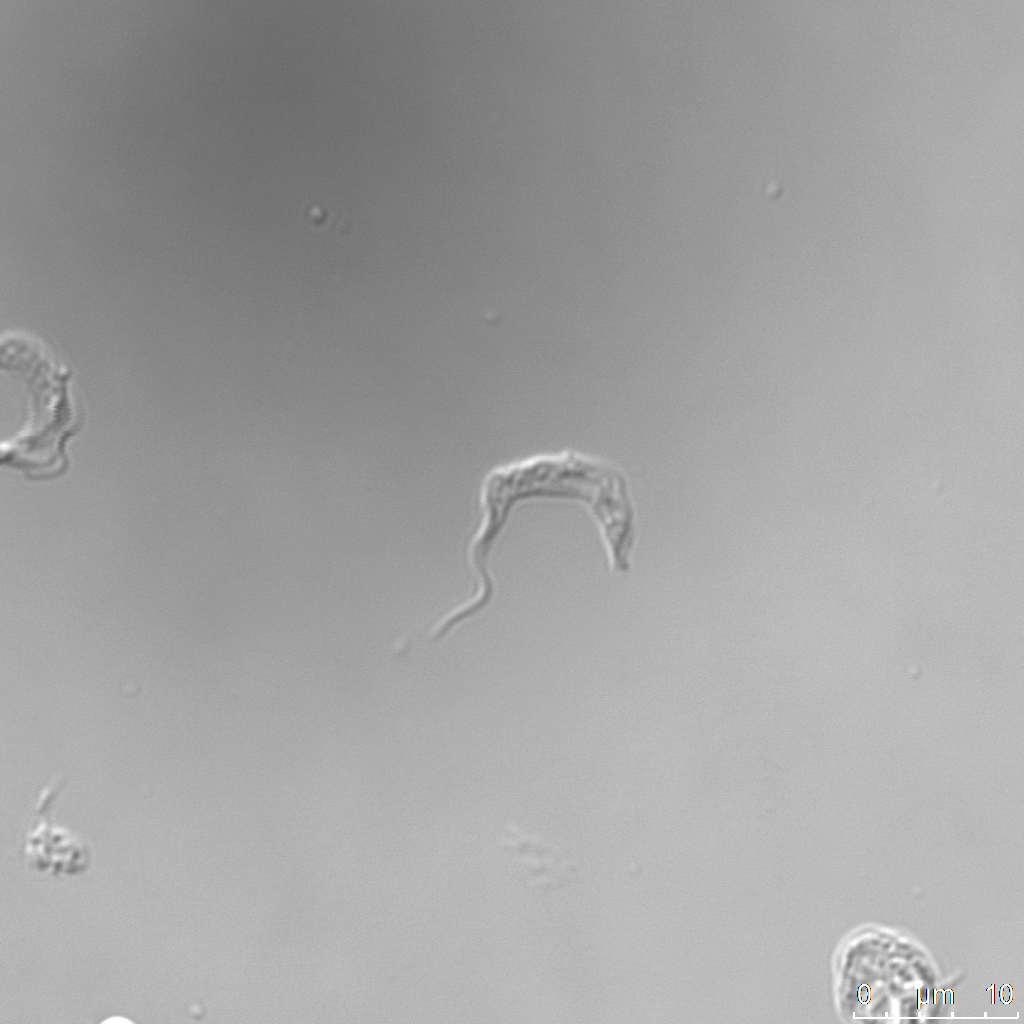

Supplement: Supplementary file 5 [file Data_Sheet_5.ZIP › Original Data 3-IFA/01 Glucose/25 mM/Project20200710_G25-4_z0_ch03.tif]

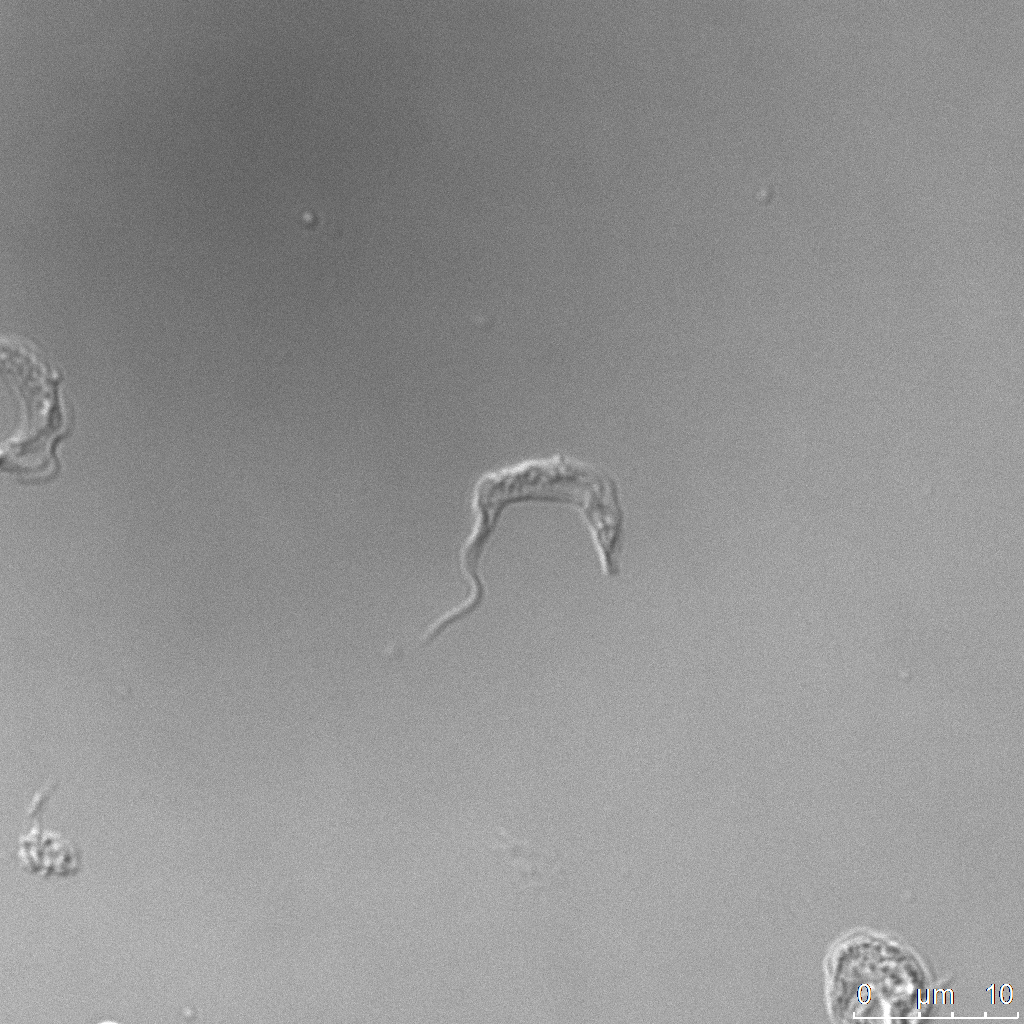

Supplement: Supplementary file 5 [file Data_Sheet_5.ZIP › Original Data 3-IFA/01 Glucose/25 mM/Project20200710_G25-4_z0_ch01.tif]

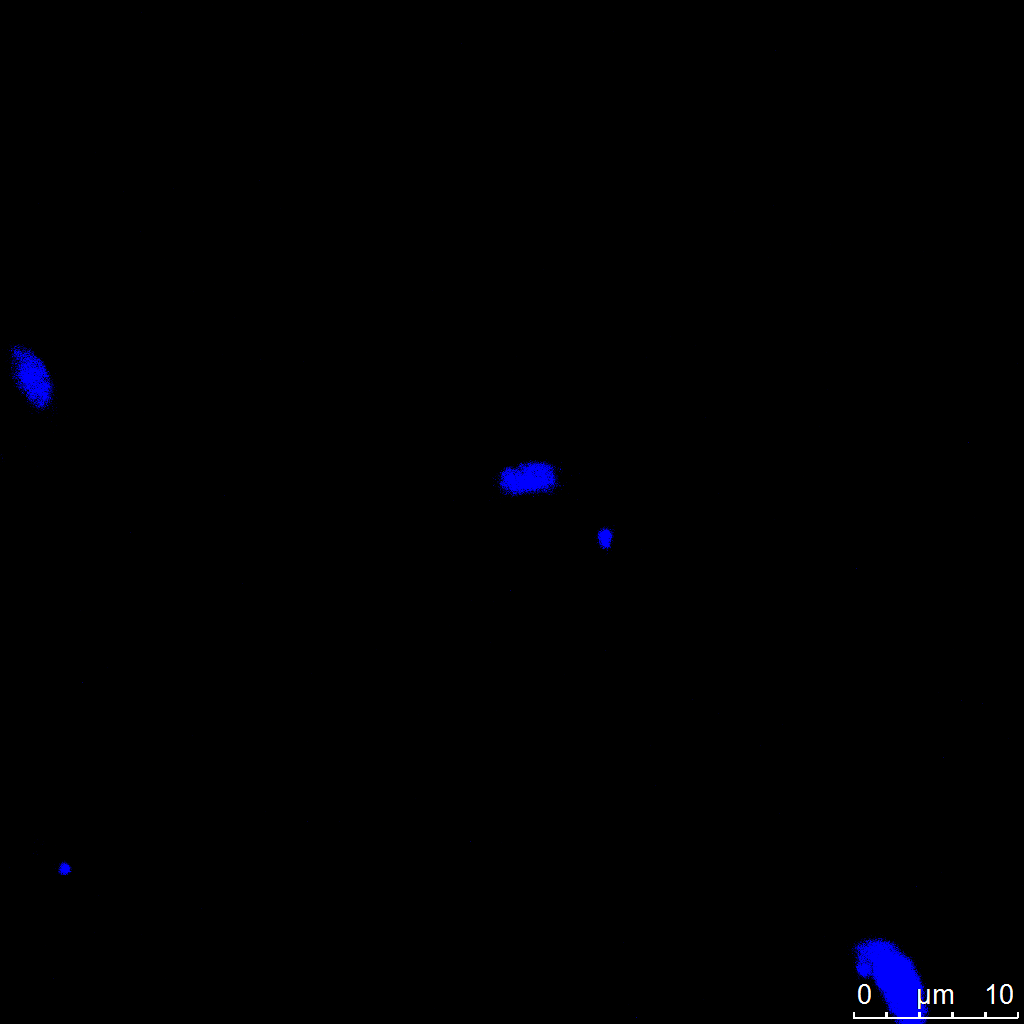

Supplement: Supplementary file 5 [file Data_Sheet_5.ZIP › Original Data 3-IFA/01 Glucose/25 mM/Project20200710_G25-4_z0_ch00.tif]
