# Supplementary material for: Protein Lactylation Critically Regulates Energy Metabolism in the Protozoan Parasite Trypanosoma brucei
Source: Front Cell Dev Biol. 2021 Oct 14;9:719720. doi: 10.3389/fcell.2021.719720 (PMC8551762; doi:10.3389/fcell.2021.719720)
Supplement: Supplementary file 6 [file Data_Sheet_6.PDF]

Supplementary Material

1 Supplementary Figures and Tables

1.1 Supplementary Figures

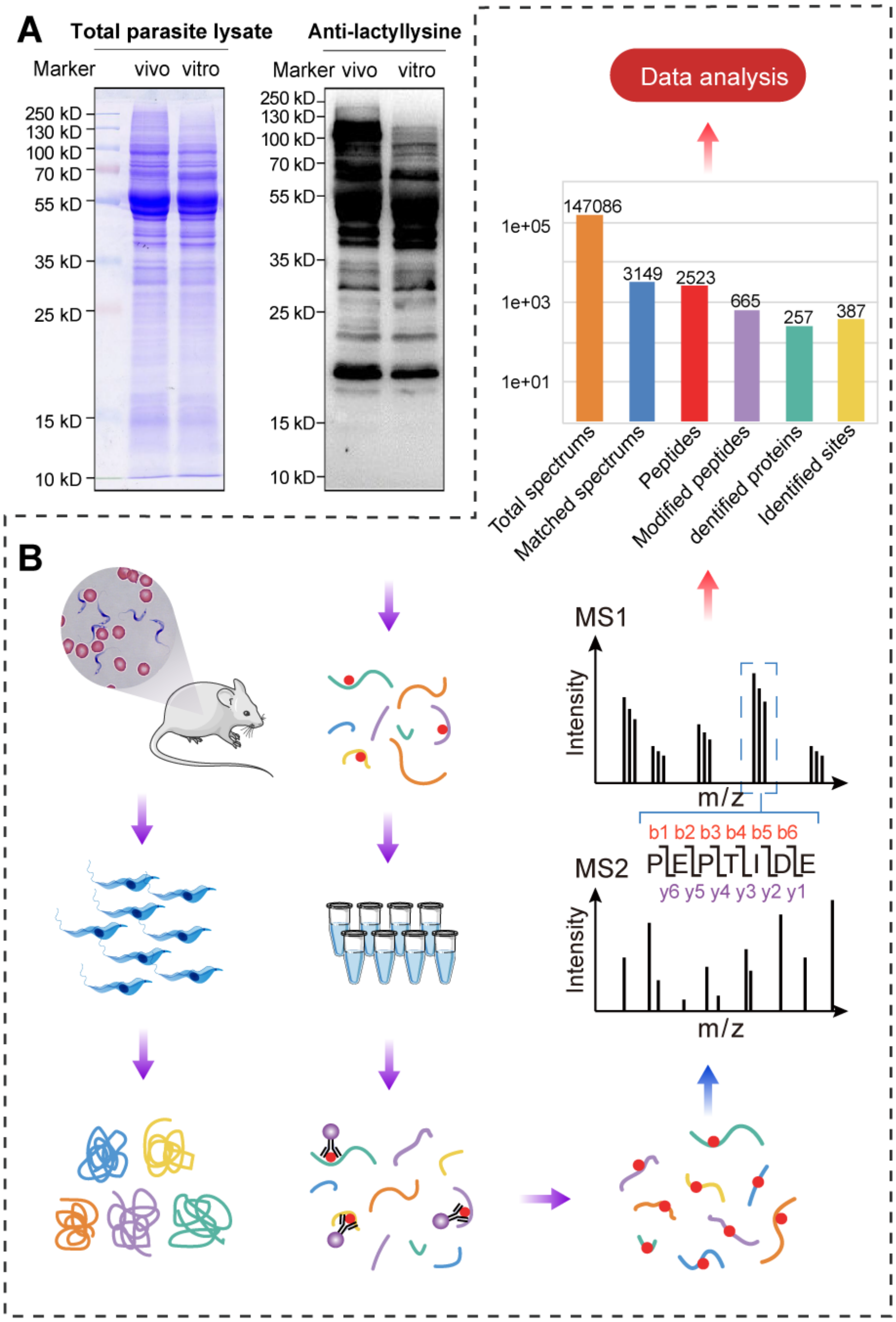

Supplementary Figure 1. Lactylation identification of BSF T. brucei. (A) Coomassie blue staining of 20 μg BSF trypanosome lysates showed equal loading amounts. Western blot was probed with a

monoclonal anti-lactyllysine antibody. (C) Flowchart illustrating proteomic procedures for lactylated protein identification. Parasite proteins were extracted and trypsinized and peptides were separated by HPLC and subjected to monoclonal pan-antibody affinity enrichment and PTM identification. The bar chart indicating basic MS statistics. The data obtained was then used for bioinformatics analysis. The purple, blue, and red arrows represent the stages of sample preparation, mass spectrometry and data analysis, respectively.

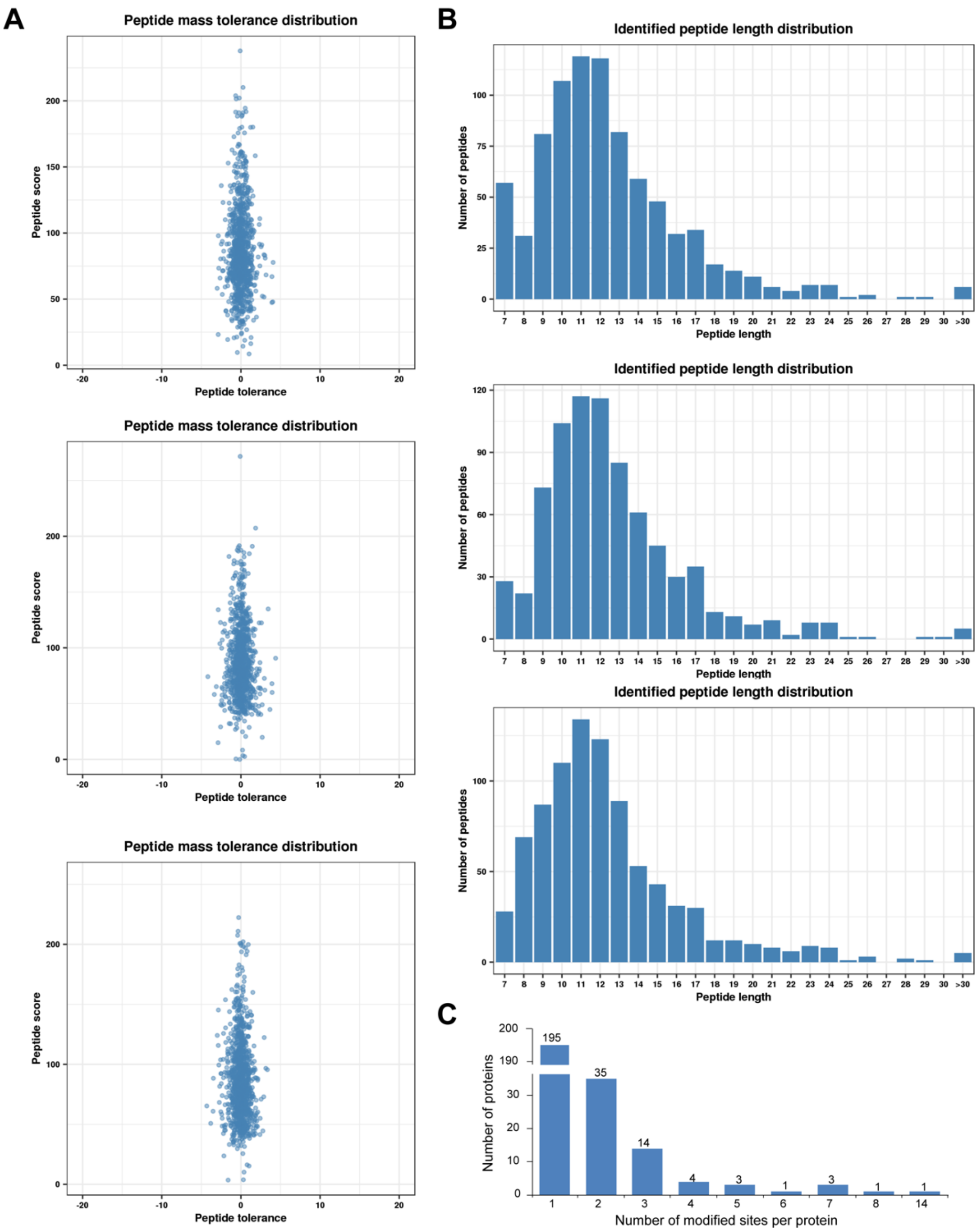

Supplementary Figure 2. Quality control of lactylated proteins in *T. brucei*. (A) Scatter plot shows the peptide mass tolerance distribution of peptides identified by mass spectrometry in three

replicates. (B) Bar plots displaying the length distribution of peptides identified by mass spectrometry in three replicates. (C) The number of proteins with different number of lactylated sites.

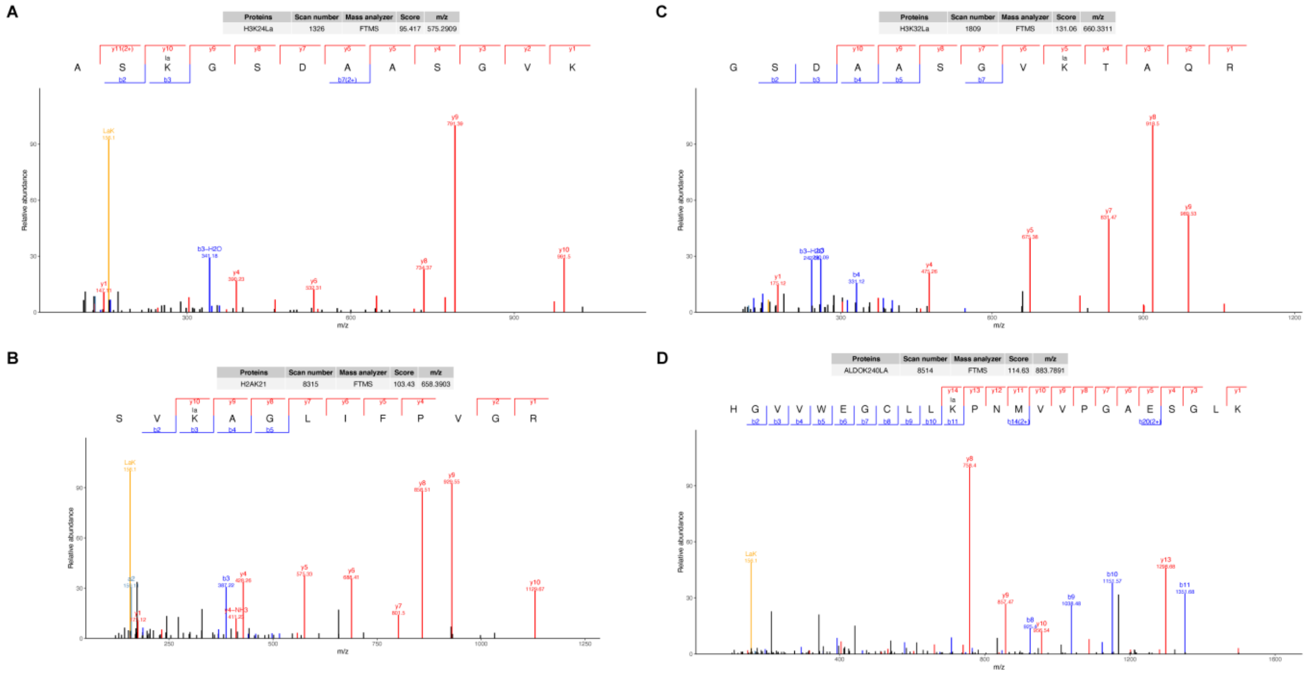

Supplementary Figure 3. Typical Lysine lactylation spectra. MS/MS spectra of lactylated peptides ((A)H3K23, (B)H2AK20, (C) H3K32, (D) ALDO K240). The b ion refers to the N-terminal parts of the peptide, and the y ion refers to the C-terminal parts of the peptide.

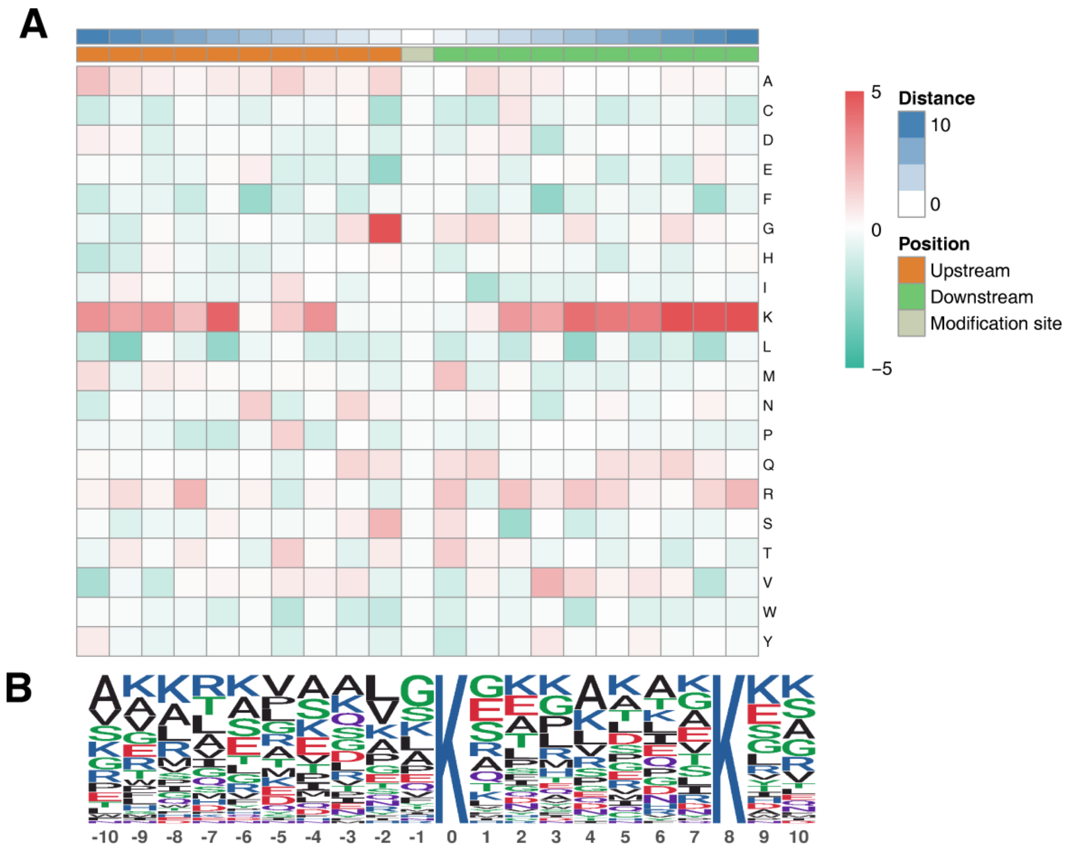

Motif	Motif Score	Foreground		Background		Fold Increase
		Matches	Size	Matches	Size	
xxxxxxxxxx_K_xxxxxxxKxx	7.09	51	382	10753	180301	2.2

Supplementary Figure 4. Lysine lactylation motif identification. (A) Heat map showing enrichment (red) or depletion (green) of amino acids in specific positions flanking the lactylated lysine. (B) Probability sequence motifs of lactylated sites consisting of 20 residues surrounding the targeted lysine residue produced using Motif-x. The size of each letter correlates to the frequency of that

amino acid residue occurring in that position. The table shows this modified site feature sequence and its enrichment statistics from MoMo software.

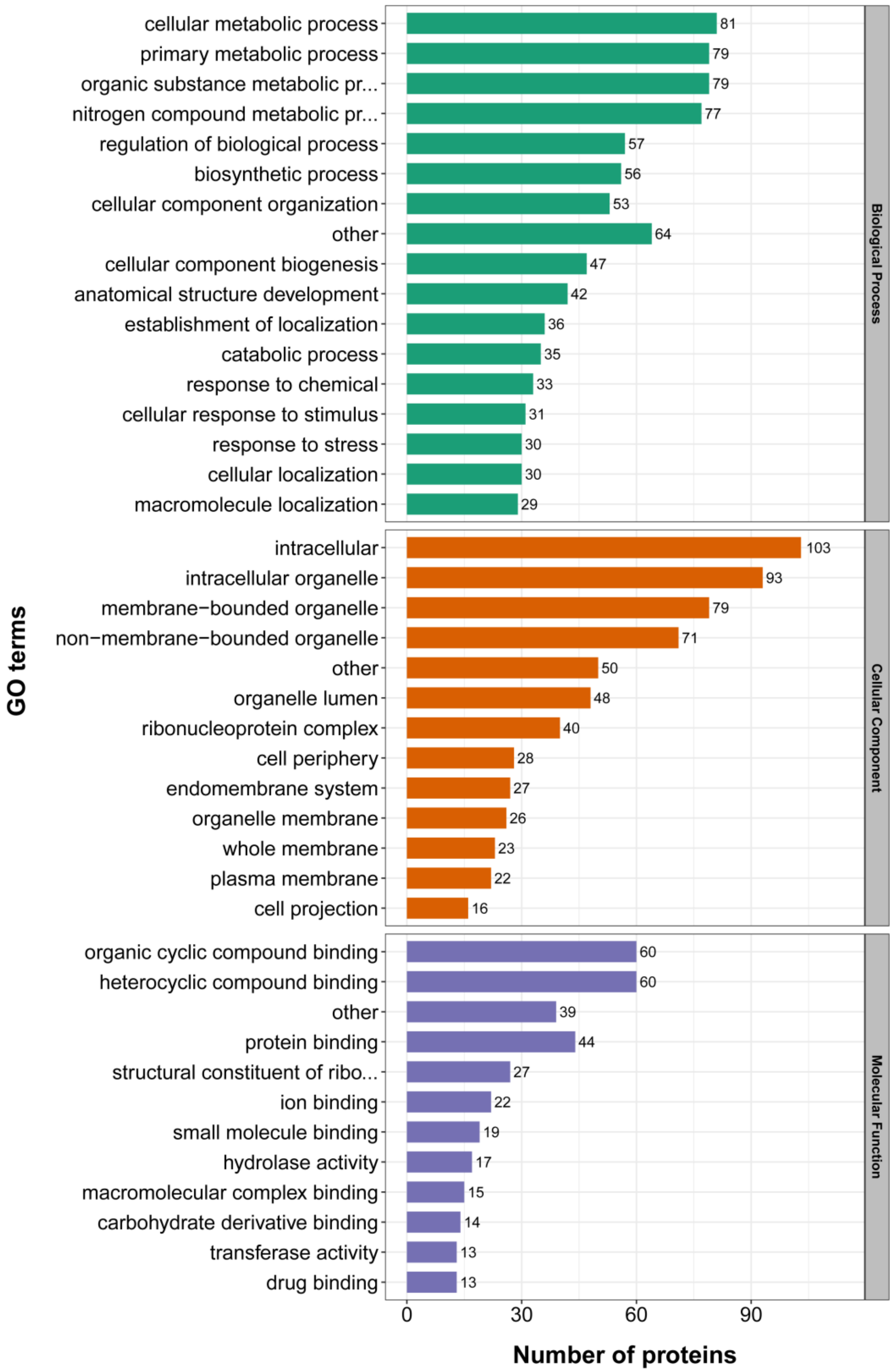

Supplementary Figure 5. *T. brucei* lysine lactylome characterization. Statistical distribution chart of proteins corresponding to modification sites under each GO category (2nd Level). GO functional

classification of all lactylated proteins was predicted based on three major categories: Biological Process, Cell component, and Molecular Function.

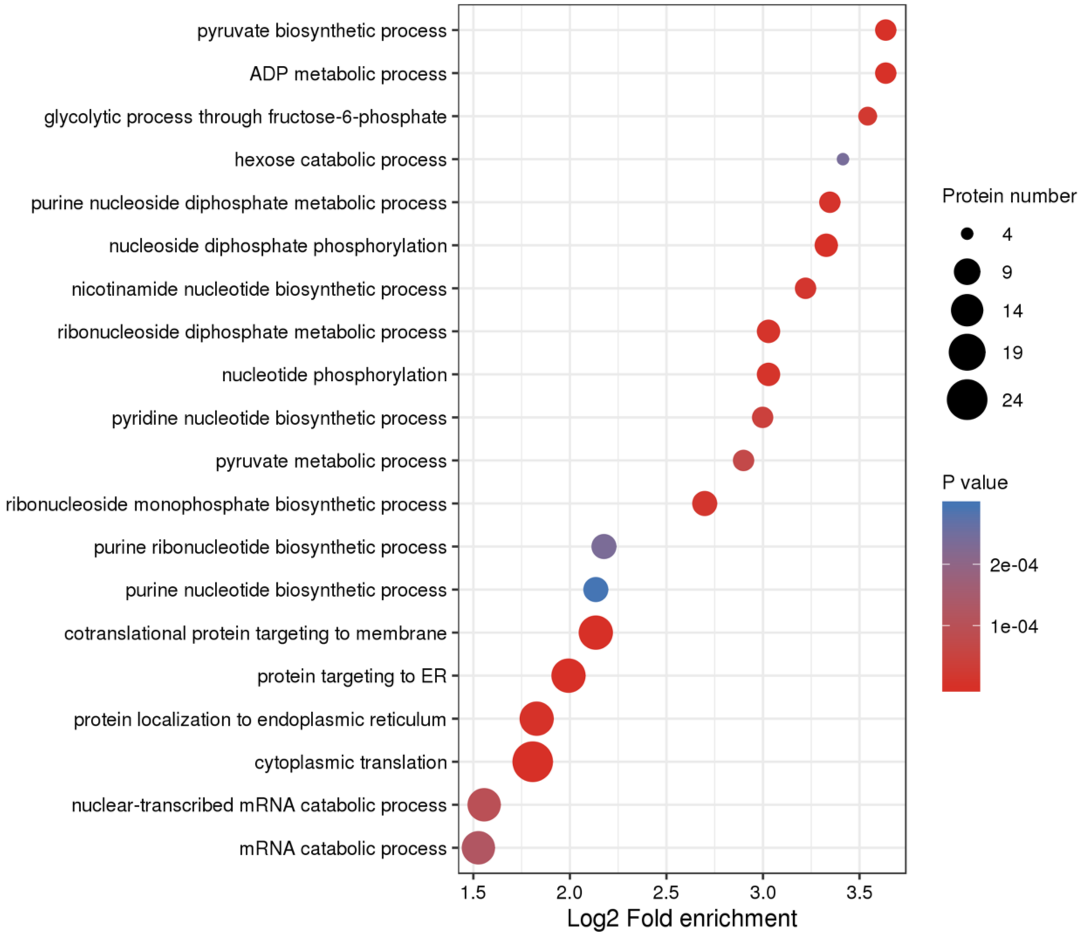

Supplementary Figure 6. Functional enrichment analysis of Biological Process. Bubble chart of enrichment distribution of proteins corresponding to lactylated sites in Biological Process (Fisher's exact test, $p < 0.05$).

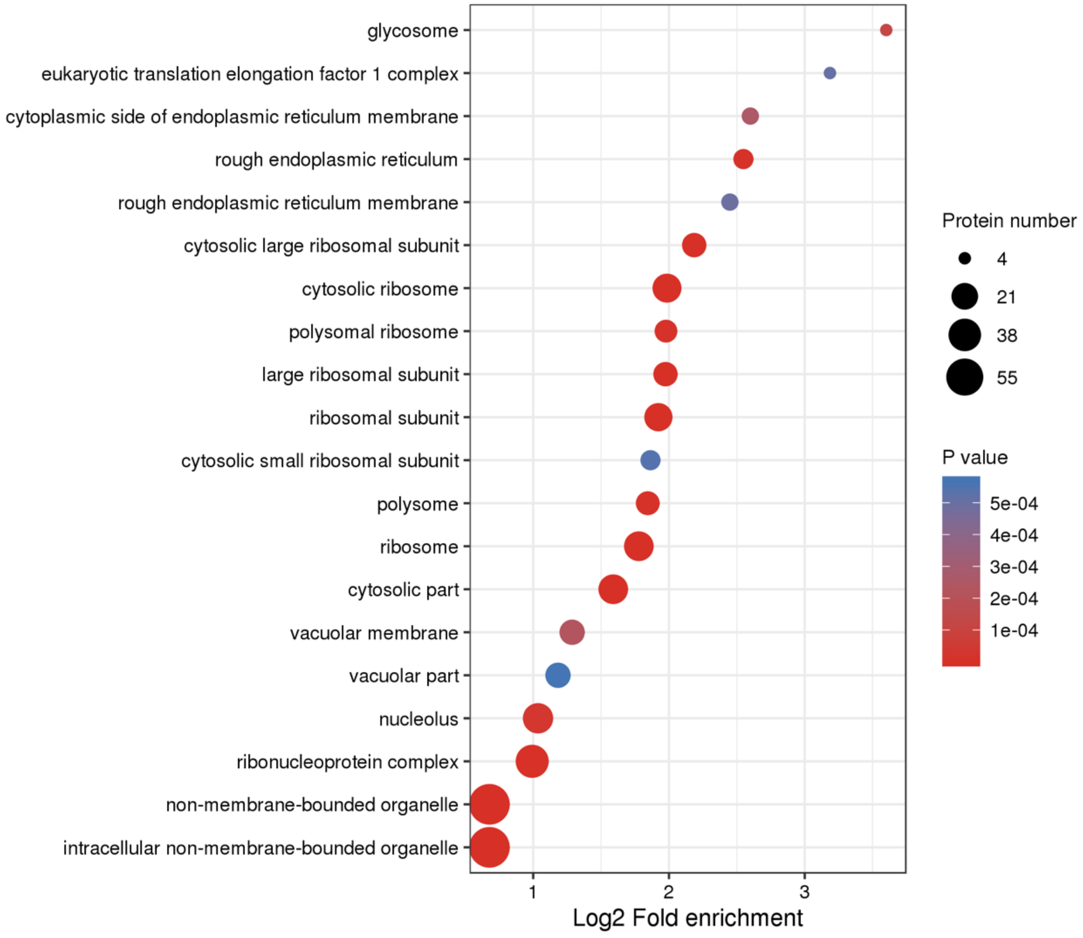

Supplementary Figure 7. Functional enrichment analysis of Cellular Component. Bubble chart of enrichment distribution of proteins corresponding to lactylated sites in Cellular Component (Fisher's exact test, $p < 0.05$).

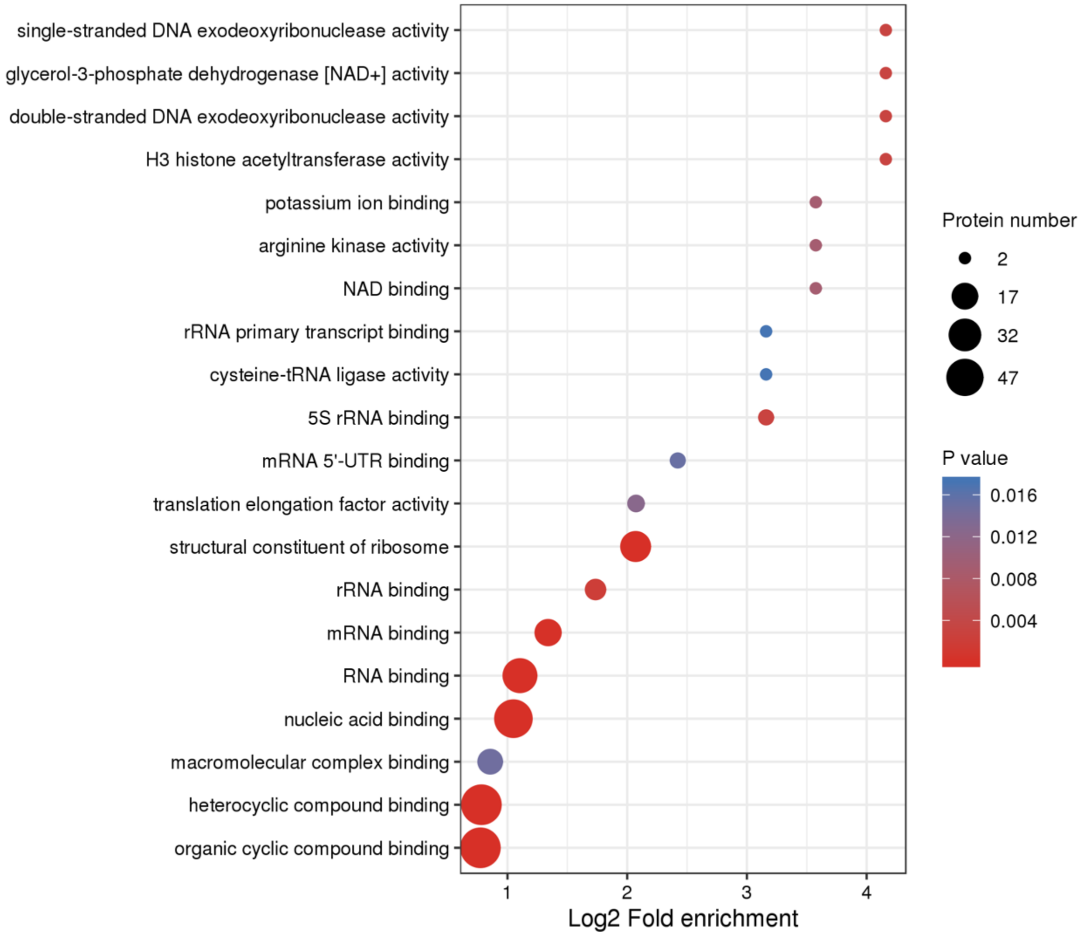

Supplementary Figure 8. Functional enrichment analysis of Molecular Function. Bubble chart of enrichment distribution of proteins corresponding to lactylated sites in Molecular Function (Fisher's exact test, $p < 0.05$).

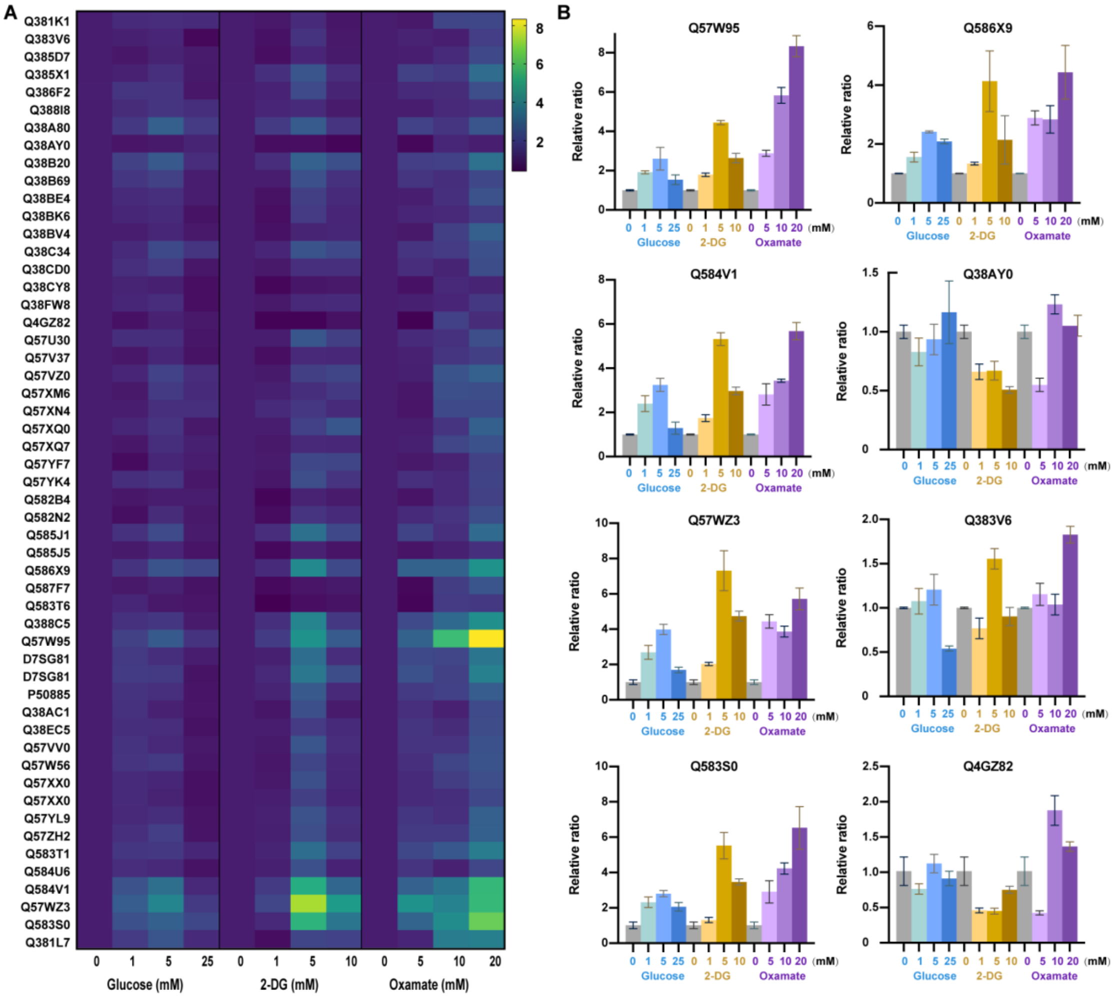

Supplementary Figure 9. Protein lactylation participates in gene expression. (A) Heatmap display quantitative analysis of RNA expression of gene regulators in T. brucei treated with glucose, 2-DG or oxamate for 24 h by real-time PCR. (B) Bar plots select the more significant groups to be shown more clearly from Figure A.

1.2 Legends of Supplementary Tables

Supplementary Table 1. Basic statistical data of MS results.

Supplementary Table 2. Protein annotation of lactylated proteins.

Supplementary Table 3. GO enrichment of lactylated proteins.

Supplementary Table 4. Lactylation of histones and their variants.

Supplementary Table 5. Lactylation of gene regulators.

Supplementary Table 6. Lactylation of glycolytic enzymes.
